# Systems Analysis of Insulin and IGF1 Receptors Networks in Breast Cancer Cells Identifies Commonalities and Divergences in Expression Patterns

**DOI:** 10.3389/fendo.2020.00435

**Published:** 2020-07-07

**Authors:** Rive Sarfstein, Adva Yeheskel, Tali Sinai-Livne, Metsada Pasmanik-Chor, Haim Werner

**Affiliations:** ^1^Department of Human Molecular Genetics and Biochemistry, Sackler School of Medicine, Tel Aviv University, Tel Aviv, Israel; ^2^Bioinformatics Unit, George Wise Faculty of Life Sciences, Tel Aviv University, Tel Aviv, Israel; ^3^Yoran Institute for Human Genome Research, Tel Aviv University, Tel Aviv, Israel

**Keywords:** insulin-like growth factor-1 (IGF1), IGF1 receptor (IGF1R), insulin receptor (INSR), systems analysis, BioNSi, network simulation

## Abstract

Insulin and insulin-like growth factor-1 (IGF1), acting respectively via the insulin (INSR) and IGF1 (IGF1R) receptors, play key developmental and metabolic roles throughout life. In addition, both signaling pathways fulfill important roles in cancer initiation and progression. The present study was aimed at identifying mechanistic differences between INSR and IGF1R using a recently developed bioinformatics tool, the Biological Network Simulator (BioNSi). This application allows to import and merge multiple pathways and interaction information from the KEGG database into a single network representation. The BioNsi network simulation tool allowed us to exploit the availability of gene expression data derived from breast cancer cell lines with specific disruptions of the INSR or IGF1R genes in order to investigate potential differences in protein expression that might be linked to biological attributes of the specific receptor networks. Modeling-generated information was corroborated by experimental and biological assays. BioNSi analyses revealed that the expression of 75 and 71 genes changed during simulation of IGF1R-KD and INSR-KD, compared to control cells, respectively. Out of 16 proteins that BioNSi analysis was based on, validated by Western blotting, nine were shown to be involved in DNA repair, eight in cell cycle checkpoints, six in proliferation, eight in apoptosis, seven in oxidative stress, six in cell migration, two in energy homeostasis, and three in senescence. Taken together, analyses identified a number of commonalities and, most importantly, dissimilarities between the IGF1R and INSR pathways that might help explain the basis for the biological differences between these networks.

## Introduction

The insulin-like growth factors (IGFs) create a complex network of ligands (insulin, IGF1, and IGF2), cell-surface transmembrane receptors [insulin receptor (INSR), IGF1 receptor (IGF1R), and IGF2 receptor (IGF2R)], and IGF binding proteins (IGFBPs) that, in a highly orchestrated manner, govern many physiological events, including endocrine, metabolic, nutritional, body growth, and aging processes (1–3). INSR and IGF1R display a remarkable similarity in their genomic organization as well as in the overall structures of the mature, tyrosine kinase-containing heterotetrameric cell-surface receptors (4, 5).

Insulin and IGF1, acting respectively *via* the INSR and IGF1R, display important metabolic and developmental roles at each period of life (6–8). While INSR and IGF1R share the majority of their downstream cytoplasmic mediators, clinical, and experimental data indicates that INSR activation leads predominantly to metabolic activities. On the other hand, IGF1R activation precedes growth events (9, 10). The IGF2R resembles the mannose 6-phosphate receptor, a membrane protein involved in the recycling of lysosomal enzymes (11). The ability of insulin and IGF1 to bind and activate the opposite receptor has been widely reported in the scientific literature (12). Cross-activation usually takes place at elevated concentrations of the hormone (at least one order of magnitude higher doses than those required by the high affinity-binding ligand to activate its cognate receptor) (13). Thus, high ambient values of insulin can activate the IGF1R, leading to proliferative activities, while excessive IGF1 can stimulate the INSR, leading to metabolic actions (5, 9). Pathological dysregulation of the IGF system is linked to a number of conditions, ranging from growth deficits to cancer development (14–16). The involvement of the INSR and IGF1R in breast cancer has been extensively reported (17–20). However, the complexity of this hormonal network led to conflicting results regarding the relative impact of the various players (i.e., ligands, receptors, etc.) in malignancy (21–23). Furthermore, this complexity translated into a disappointingly slow pace in the development of INSR/IGF1R-directed therapies (24–26). Genome-wide analyses of the mechanisms of action of insulin and IGF1 in breast cancer, as well as identification of the signaling pathways involved, is expected to be of major translational relevance.

Modeling and simulation of regulatory networks became an integral part of biological research (27). Network simulation tools are aimed at elucidating the interactions between genes, proteins and signaling pathways and, furthermore, are designed to provide new insights into complex biological questions. Simulation tools allow the visualization and analysis of the dynamics of local biological networks. The Biological Network Simulator (BioNSi) tool is a computational application that allows to import and merge multiple pathways and interaction information from the KEGG database into a single network representation (http://bionsi.wix.com/bionsi) (28, 29). Moreover, BioNSi enables the upload of expression values from high-throughput experiments and simulate the gene or protein interactions acquired from KEGG accordingly. Data generated may facilitate the investigation of the entire dynamic network under different biological conditions (30, 31). Given the vital roles of the INSR and IGF1R signaling pathways in breast cancer, and to gain new insight into potential commonalities and divergences in expression patterns between both receptors and their downstream mediators, we employed the BioNSi bioinformatics tool for network simulation. MCF7 breast cancer-derived cells with specific disruption of the INSR or IGF1R were used to study gene expression. BioNSi bioinformatics analyses integrated and simulated nine KEGG pathways, including apoptosis, cell cycle regulation, PI3K/Akt signaling, senescence and others, to a single network, based on specific gene expression. Our focus on these biological pathways stems from the fact that an important body of work over the past years revealed critical differences between the INSR and IGF1R pathways in these paths. Modeling-generated information was corroborated by experimental analyses.

## Materials and Methods

### MCF7 Breast Cancer-Derived INSR-KD and IGF1R-KD Cell Lines

The generation of MCF7-derived INSR-knock down (KD) and IGF1R-KD cell lines has been recently described (32). Briefly, GIPZ plasmids encoding the following microRNA-adapted short hairpin RNAs (shRNA): TGACTGTGAAATCTTCGGC (human IGF1R) and CTTACCAAGGCCTGTCTAA3 (human INSR), packed in lentiviral particles, were obtained from Open Biosystems (Huntsville, AL, USA). Plasmids were transfected into breast carcinoma-derived estrogen receptor-positive MCF7 cells (American Type Culture Collection, Manassas, VA, USA). In addition, a plasmid containing a non-coding shRNA sequence (control shRNA) was transfected into the same cells. Cells were maintained in DMEM supplemented with 10% fetal bovine serum, 100 units/ml penicillin, 100 μg/ml streptomycin, 5.6 μg/ml amphotericin B, and 1 μg/ml puromycin. MCF7-derived cell lines were a gift of Drs. Derek LeRoith and Ran Rostoker (Technion, Haifa, Israel).

### Microarray Experiment

MCF7 cells were seeded in 10-cm Petri dishes until reaching 100% confluence. The cells were washed with phosphate-buffered saline (PBS) and total RNA was isolated using the TRIzol reagent (InVitrogen, Waltham, MA, USA), according to the RNA extraction guidelines for Affimetrix GeneChip Assays. Affymetrix® Human Gene 2.1 ST Array Strip (#902114, Santa Clara, CA, USA) was used for gene expression analysis according to manufacturer's instruction manual (www.affymetrix.com). Partek Genomics Suite was used for analysis (www.partek.com/partek-genomics-suite/). The untreated MCF7 cells gene expression data is available as Supplementary Data Sheet 1.

### Protein Analysis and Immunoblotting

Cells were harvested and lysed in a buffer containing proteases and phosphatases inhibitors. Samples were electrophoresed through 5, 10, 12, or 15% SDS–PAGE, followed by protein transfer onto nitrocellulose membranes. Blots were blocked with 5% skim milk and incubated overnight with antibodies against Cyclin D1 (#DCS6), ATM (#D2E2), JAK1 (#3332), STAT3 (#79D7), Caspase-3 (#8G10), AKT (#9272), mTOR (#7C10), and Ampk (#2532). These antibodies were obtained from Cell Signaling Technology (Danvers, MA, USA). An antibody against SOD2 was obtained from Enzo Life Sciences (Farmingdale, NY, USA). Antibodies against Chek2 (#A-11), p53 (mixture of DO-1 and 1801), and Hsp70 (#B-6) were obtained from Santa Cruz Biotechnology (Dallas, TX, USA). An antibody against Ras (#Ab-3) was purchased from Oncogene Research Products (La Jolla, CA, USA). Blots were washed and incubated with the corresponding horseradish peroxidase-conjugated secondary antibody. Proteins were detected using the enhanced chemiluminiscence reaction (Westar Supernova, Cyanagen, Bologna, Italy). Hsp70 was used as a loading control.

### Cell Cycle Analysis

Cells were seeded in duplicate onto 6-well-plates (10^4^ cells/well) for 24 h. Cells were then serum-starved for an additional 24 h and incubated in the presence or absence of IGF1 or insulin (50 ng/ml) for 72 h. After incubation, cells were washed with PBS, trypsinized, and pelleted by centrifugation. The cells were permeabilized with Triton-X100, after which propidium iodide was added. Stained cells were analyzed using a FacsCalibur system (Cytek Development Inc., Fremont, CA, USA).

### Cell Senescence Assays

Cells were seeded in 24-well-plates (5 × 10^4^ cells/well) for 24 h. Cells were then serum-starved for an additional 24 h and incubated in the presence or absence of etoposide (10 μM) for 48 h. After incubation, cells were washed with PBS, fixed with 2% formaldehyde/0.2% glutaraldehyde for 5 min and incubated overnight at 37°C in staining solution [40 mM sodium citrate, pH 6.0, 1% 5-bromo-4-chloro-3-indolyl-β-D-galactopyranoside (X-gal), 5 mM potassium ferrocyanide, 5 mM ferricyanide, 150 mM sodium chloride, and 2 mM magnesium chloride] (33). Cultures were examined under phase-contrast microscopy. The development of a blue color in the cytoplasm was detected and photographed using an inverted microscope (Nikon Instruments Inc., Lewisville, TX, USA). Four pictures were taken of each well. β-galactosidase-stained and unstained cells were counted and used to calculate a final average ratio for each well from quadruplicate samples.

### Bioinformatics Network Analyses

Nine human KEGG pathways (based on 16 key genes) (Table 1) were imported to BioNSi tool to form a single network for simulation analysis. The rationale for this choice of genes is the fact that they are regarded as prototypical targets of insulin and/or IGF1 action (5, 9, 10, 16).

**Table 1:** List of KEGG pathways analyzed by BioNSi (including 16 selected key genes).

	**EGFR tyrosine kinase inhibitor resistance**	**AMPK signaling pathway**	**Insulin signaling pathway**	**p53 signaling pathway**	**Cell cycle**	**FoXO signaling pathway**	**PI3K-Akt signaling pathway**	**Apoptosis**	**Cellular senescence**
ATM									
SOD2									
STAT3									
CHEK2									
TP53									
IGF1R									
INSR									
AKT3									
CCND1									
CASP3									
HRAS									
CDKN1A									
MTOR									
JAK1									
PRKAA1 (AMPK)									
CDKN2A									

*Nine pathways were selected for BioNSi network analysis [EGFR tyrosine kinase inhibitor resistance (hsa01521), AMPK signaling pathway (hsa04152), Insulin signaling pathway (hsa04910), P53 signaling pathway (hsa04115), Cell cycle (hsa04110), FoxO signaling pathway (hsa04068), PI3K-Akt signaling pathway (hsa04151), Apoptosis (hsa04210), cellular senescence (hsa04218)]. The 16 selected genes that participate in specific KEGG pathways (gray boxes) were tested by immunoblotting assays*.

**Figure 1:**
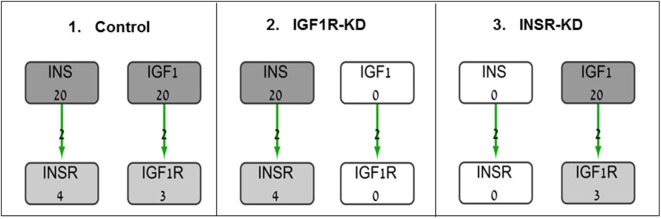
Diagram of BioNSi simulations experimental design. Network nodes are shown as rectangles with gene name and an initial normalized expression value below it. Grayscale coloring of nodes represents gene expression of control MCF7 cells. Green edges are shown as directed arrows, representing gene activation (with level 2, as indicated in section Materials and Methods). Initial expression values for the key genes affecting gene expression [INSR, insulin (INS), IGF1R, and IGF1] are presented (simulations: 1, control; 2, INSR-KD; and 3, IGF1R-KD). Initial expression of INS and IGF1 were set manually to 0 (KD) or 20 (very high).

The BioNSi global network that includes all of these nine pathways contains 385 nodes (genes and small molecules) that are connected by 631 edges, in addition to gene self-inhibition edges that represent biological degradation (30). KEGG pathway information includes activation and inhibition edges. BioNSi settings were: activation edges were set to +2, inhibition to −1, phosphorylation to +2, dephosphorylation to −1, ubiquitination to −1, binding/association to +2, dissociation to −1, and self-inhibition to −1. In order to simulate the effect of activation and inhibition between nodes over time, untreated MCF7 gene expression microarray results were imported to BioNSi analysis as baseline (GSE145787; described above). The average expression values for replicate control cells were used as initial values for the nodes in the network and were normalized by the BioNSi tool to be between 0 and 9 (grayscale). Three different BioNSi simulation analyses were compared as follows (all simulations were run for 100 steps as shown in Figure 1):

1. Control simulation, in which IGF1 and insulin initial expression values were manually set to 20 (very high), instead of 2 and 0, respectively. In order to observe the maximal changes in gene expression along the complete interaction network during simulation, expression values of insulin and IGF1 were increased.
2. IGF1R-KD, in which IGF1R and its ligand, IGF1, initial expression values were manually set to 0 (not detectable), but insulin and INSR expression values are intact.
3. INSR-KD, in which INSR and its ligand, insulin, initial expression values were manually set to 0 (not detectable), but IGF1 and IGF1R expression values are intact.

The complete network is available as a *Cytoscape* session file (Supplementary Data Sheet 2).

### Statistical Analyses

The statistical significance of the differences between groups was assessed by Student's *t*-test (two samples, equal variance). Scanning densitometry analyses were evaluated using the TINA imaging analysis software (http://biochemlabsolutions.com/GelQuantNET.html). Signal intensities of proteins were normalized to the corresponding Hsp70 signals. Data are presented as mean ± SEM of three independent experiments. *P* < 0.05 or 0.01 were considered statistically significant.

## Results

### BioNSi Analysis of MCF7-Derived INSR-KD and IGF1R-KD Breast Cancer Cell Lines

**Figure 2:**
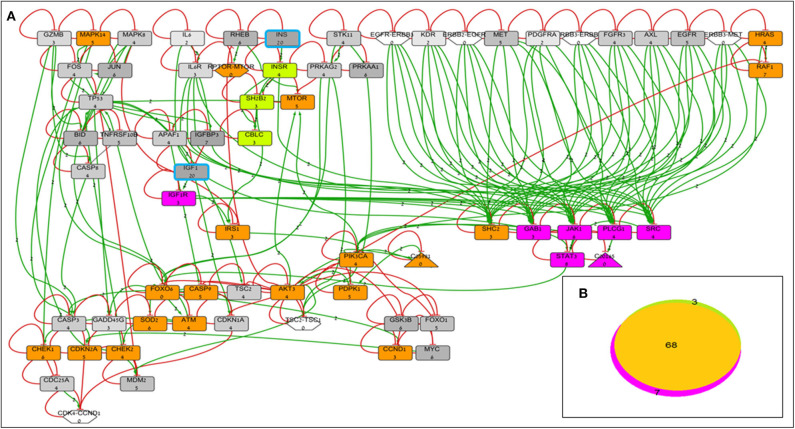
BioNSi network and simulation analyses of MCF7-derived breast cancer cell lines. **(A)** Reduced network of 68 genes (only biologically tested genes and their neighbors), colored according to their initial expression value (grayscale). The initial expression value is written underneath each gene name. INS and IGF1 expression was manually set as very high (20), and genes are highlighted in turquoise frame. Complexes initial expression is zero by BioNSi default. Activation arrow edges are colored green. Inhibition edges are colored red with flat heads. Simulation analyses were performed on the complete network (385 genes). Node's background was changed after simulations: genes whose expression changed during simulation in both KD cells are colored orange; genes whose expression changed during simulation only in IGF1R-KD cells are colored purple; and genes whose expression changed during simulation only in INSR-KD cells are colored green. **(B)** Venn diagram of numbers of genes whose expression changed during simulation (specific KD vs. control). Nodes whose expression changed only in IGF1R-KD cells are colored purple (IGF1R, STAT3, GAB1, JAK1, PLCG1, SRC, and diacylglycerol). Genes whose expression changed during simulation only in INSR-KD are colored green (INSR, SH2B2, and CBLC). Overlapping genes whose expression changed during simulation in both KD cells are colored orange.

**Figure 3:**
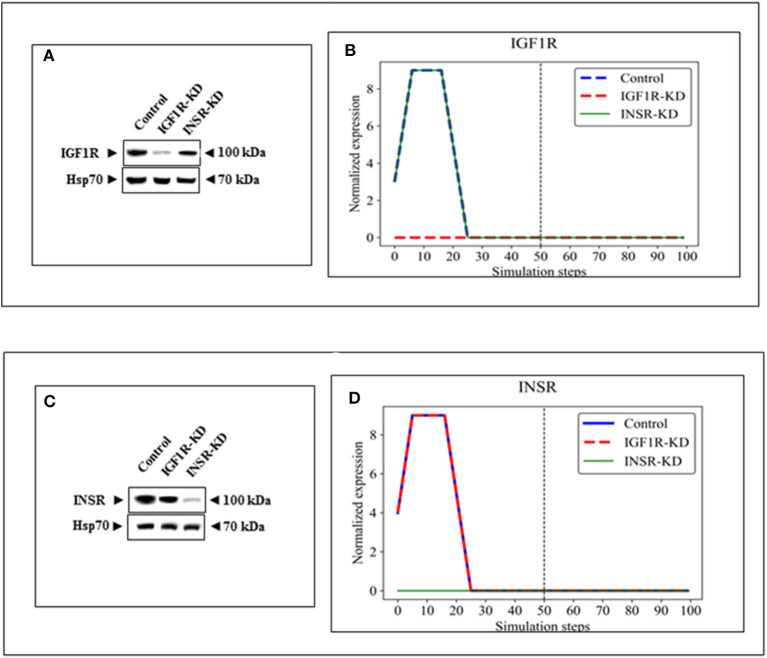
Western blots and BioNSi simulation analyses of IGF1R and INSR expression after KD. Western blots of IGF1R **(A)** and INSR **(C)** were conducted using total lysates of Control, IGF1R-KD and INSR-KD cells. One-hundred simulation steps were performed as described in Figure 2. A dashed vertical line indicates 50 steps of simulation. BioNSi plots of expression values against simulation steps are shown **(B,D)**. Control (blue), IGF1R-KD (red), and INSR-KD (green). Splicing has occurred in the blot figures and full scans of the entire original (unprocessed) gels are presented in Supplementary Material. Squares in the uncropped films denote bands shown in the final figures.

**Figure 4:**
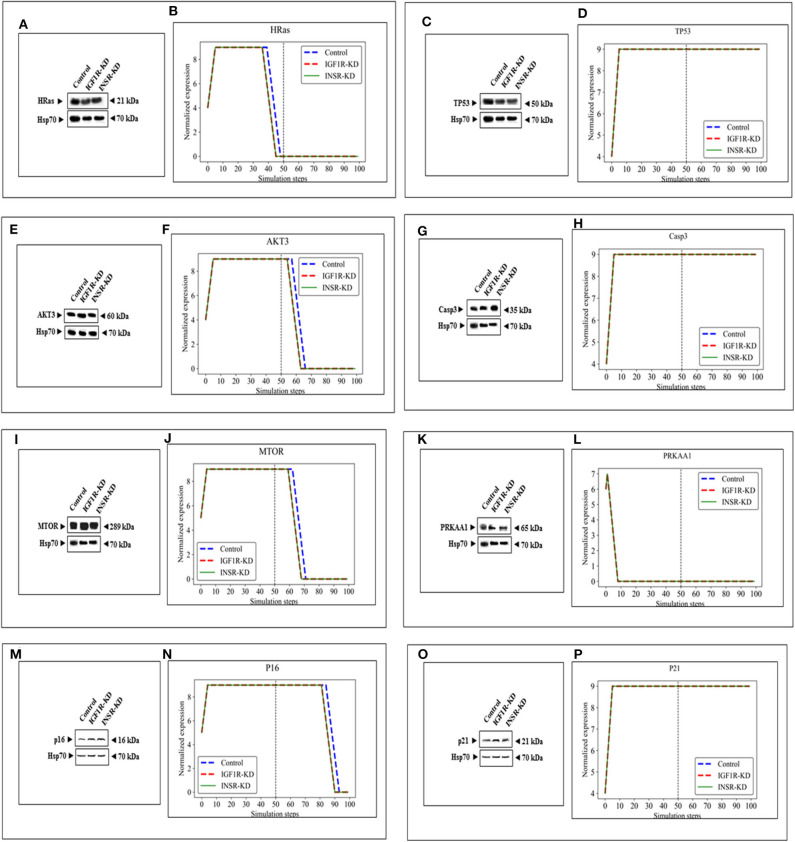
Eight selected genes that do not change between control and KDs: BioNSi simulations validated by Western blots. Western blot analyses showing expression of HRas **(A)**, TP53 **(C)**, AKT3 **(E)**, CASP3 **(G)**, MTOR **(I)**, PRKAA1 **(K)**, p16 **(M)**, and p21 **(O)** proteins were conducted on whole cell lysates of Control, IGF1R-KD, and INSR-KD cells. Hsp70 was used as a loading control. Data are representative of two independent experiments. **(B,D,F,H,J,L,N,P)** BioNSi plots of normalized expression values against 100 simulation steps in Control (blue), IGF1R-KD (red), and INSR-KD (green) cells. A dashed vertical line indicates 50 steps of simulation. Splicing has occurred in the blot figures and full scans of the entire original (unprocessed) gels are presented in Supplementary Material. Squares in the uncropped films denote bands shown in the final figures.

**Figure 5:**
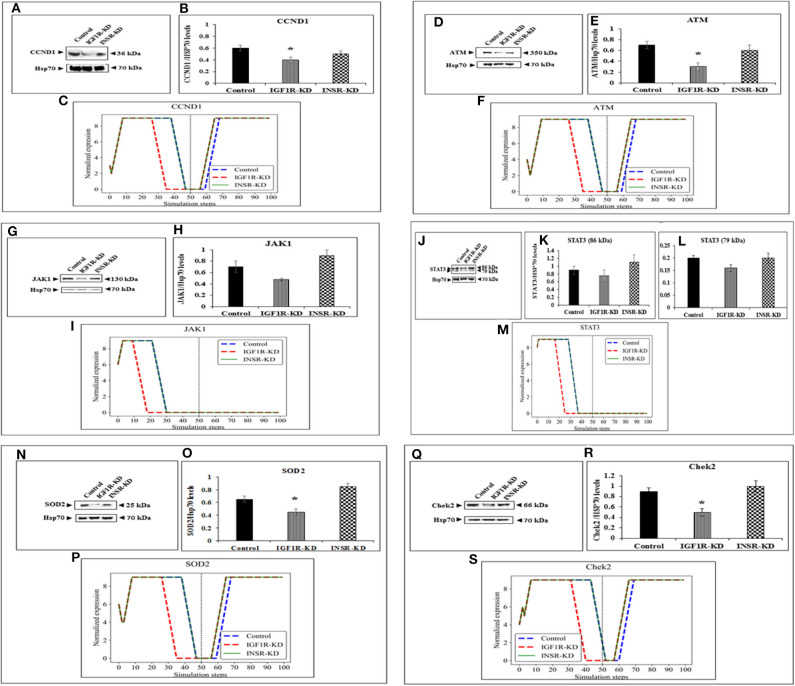
Six selected genes exhibiting a change between control and IGF1R-KD, validated by Western blots. Western blot analysis showing expression of CCND1 **(A)**, ATM **(D)**, JAK1 **(G)**, STAT3 **(J)**, SOD2 **(N)**, and Chek2 **(Q)** proteins levels were performed as described above. **(B,E,H,K,L,O,R)** Scanning densitometry analysis of basal proteins levels. Bars represent protein values (AU, arbitrary units), normalized to the corresponding Hsp70 levels. Results of an illustrative experiment, repeated twice with similar results, are shown. *p < 0.01 vs. control cells. **(C,F,I,M,P,S)** BioNSi simulation plots of normalized expression values against 100 simulation steps are shown. Control (blue), IGF1R-KD (red), and INSR-KD (green). A dashed vertical line indicates 50 steps of simulation. Splicing has occurred in the blot figures and full scans of the entire original (unprocessed) gels are presented in Supplementary Material. Squares in the uncropped films denote bands shown in the final figures.

BioNSi analyses revealed that the expression of 75 and 71 genes changed during simulation of IGF1R-KD (simulation 2) and INSR-KD (simulation 3), compared to control cells (simulation 1), respectively. As it is confusing to observe the complete network of 385 genes (see cytoscape file in Supplementary Data Sheet 2), Figure 2A shows a reduced network of 68 genes. However, the simulation analyses were performed for the complete network of 385 genes. The reduced network (68 genes) presents 16 genes that were biologically tested (Figures 3–5, detailed below) and their direct interactors. Sixty-eight out of the 385 genes in the original network changed in both types of KD cells (19 of them are presented in Figure 2A, colored orange). A Venn diagram indicating the number of genes that changed during simulations is presented in Figure 2B. The specific changes in simulations of the biologically tested genes are plotted in Figures 3–5. Figure 3 [Western blots (Figures 3A,C) and simulations (Figures 3B,D)] shows that KD of the two main effectors tested, IGF1R and INSR, resulted in distinct reduction of these specific genes. IGF1R-KD did not change the behavior of INSR, and, INSR-KD did not change IGF1R expression, as expected due to their different functions.

The expression of four genes changed in both types of disrupted cells in a similar manner during simulation [HRas (Figure 4B); AKT3 (Figure 4F); MTOR (Figure 4J); CDKN2A (P16; Figure 4N)]. The expression of four genes was unaffected by neither INSR-KD nor IGF1R-KD disruption, and this was validated by Western blotting (see below) [TP53 (Figure 4D); CASP3 (Figure 4H); PRKAA1 (AMPK; Figure 4L); CDKN1A (P21; Figure 4P)]. The expression of three genes changed only in IGF1R-KD cells [(IGF1R, Figure 3B); JAK1 (Figure 5I); STAT3 (Figure 5M)] (validated by Western blots, see below). The expression of four genes changed in both types of disrupted cells but in different directions in each simulation [CCND1 (Cyclin D1; Figure 5C); ATM (Figure 5F); SOD2 (Figure 5P); CHEK2 (Figure 5S)].

### Western Immunoblotting Validation of BioNSi Simulations

In order to validate the BioNSi simulation results, changes in protein expression levels in IGF1R-KD and INSR-KD cells were measured by Western blots and quantified by TINA software from new independent protein samples. IGF1R-KD reduced protein expression of six genes (CCND1, ATM, JAK1, STAT3, SOD2, and CHEK2), compared to INSR-KD and control cells (Figures 5A,D,G,J,N,Q, respectively). However, protein expression of eight genes [HRas, TP53, AKT3, CASP3, MTOR, PPKAA1 (AmpK3), p16, and p21] was not markedly changed (Figures 4A,C,E,G,I,K,O, respectively). These results emphasize the different molecular functions of INSR and IGF1R in breast cancer cells.

Out of 16 proteins that BioNSi analysis was based on, validated by Western blotting, nine were shown to be involved in DNA repair, eight in cell cycle checkpoints, six in proliferation, eight in apoptosis, seven in oxidative stress, six in cell migration, two in energy homeostasis, and three in senescence (Table 2).

**Table 2:** List of 11 proteins that BioNSi analysis is based on, validated by Western blotting, according to their biological functions (gray boxes).

	**DNA repair**	**Cell cycle**	**Proliferation**	**Apoptosis**	**Oxidative stress**	**Cell migration**	**Energy homeostasis**	**Senescence**
TP53								
CASP3								
AKT3								
MTOR								
ATM								
STAT3								
JAK1								
p16								
p21								
PRKAA1								
SOD2								

### Cell Cycle Analysis

**Figure 6:**
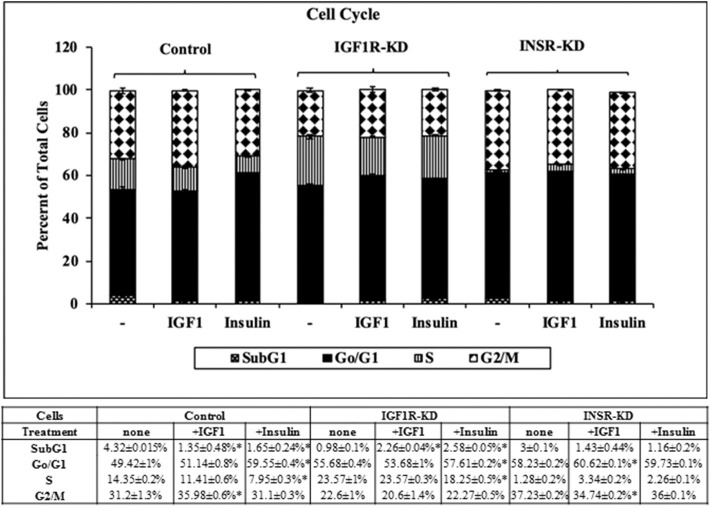
Cell cycle stages distribution in Control, IGF1R-KD, and INSR-KD cells. Cells were seeded in quadruplicate dishes, and treated with IGF1 or insulin (or left untreated, controls) for 72 h. The bars represent mean ± SEM) of three independent experiments, performed each in duplicates samples. Cell cycle distribution was measured as described in section Materials and Methods. *p = 0.05 vs. untreated cells.

Next, experiments were carried out to characterize IGF1 or insulin effects on cell cycle progression. To this end, IGF1R-KD, INSR-KD and control MCF7 cells were treated with IGF1 or insulin for 72 h, after which flow cytometry was performed on propidium iodide-stained cells. In control cells, both IGF1 and insulin decreased the proportion of cells at the SubG1 phase (from 4.3 to 1.35% or 1.65%, respectively) (Figure 6). In addition, insulin induced a decrease in the portion of cells at S phase (from 14 to 7.95%) and an increase in the portion of cells at the G0/G1 phase (from 49 to 59.55%). In contrast, in IGF1R-KD cells both IGF1 and insulin led to increases in the proportion of cells at the SubG1 phase (from 0.98 to 2.26 or 2.58%, respectively). In addition, insulin elicited a mild increase in the portion of IGF1R-KD cells at G0/G1 phase (from 55.7 to 57.6%) and a decrease in the portion of cells at S phase (from 23.6 to 18.2%). As expected, IGF1 led to a small but significant increase in the portion of cells at G0/G1 phase (from 58.2 to 60.6%) and S phase (from 1.3 to 3.3%) in INSR-KD cells. A decreased portion of cells was observed at G2/M (from 37.2 to 34.7%) in IGF1-treated INSR-KD. Insulin had no effect on cell cycle progression in INSR-KD cells. Reduction in expression levels of a number of cell cycle-associated genes [e.g., CCND1, ATM, CHEK2 (Figure 5), and TP53 (Figure 4)] in IGF1R-KD cells might, at least partly, explain the different cell cycle dynamics of IGF1R-KD and INSR-KD cells. Of interest, the simulated expression behaviors of CCND1, ATM, and CHEK2 was similar between INSR-KD and control cells (Figure 5). Taken together, results suggest that IGF1R, which is expressed by both INSR-KD and control, but not IGF1R-KD, cells has a critical role in cell cycle progression. Finally, the distinct representation of both receptors in these cells is reflected in the different proportions of cells at S phase under basal conditions.

### Senescence Assays

**Figure 7:**
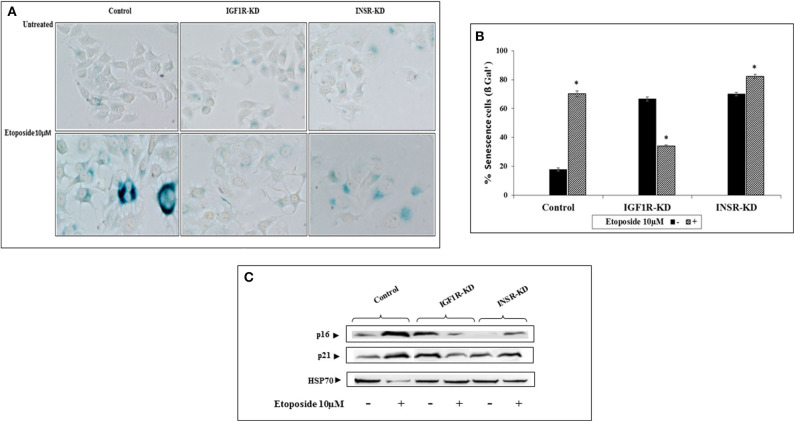
Effect of INSR or IGF1R disruption (KD) on etoposide-induced senescence. **(A)** Senescence-associated beta-galactosidase (SA-β-gal) staining (blue/green) in MCF7 stable cells after treatment with etoposide (10 μM) for 48 h. **(B)** Percentage of SA-β-gal positive cells was counted in at least three random fields from quadruplicates samples. Graph represents means ± SEM (*n* = 4). *Statistically significantly different from controls, *p* < 0.01. **(C)** Western blotting of p16 and p21 protein expression in the presence (+) or absence (–) of etoposide in MCF7 stable cells. Hsp70 was used as a loading control. Splicing has occurred in the blot figures and full scans of the entire original gels is presented in Supplementary Material. Squares denote bands shown in the final figures.

To examine the differential involvement of INSR and IGF1R in acquisition of a senescence phenotype, the ability of etoposide (a DNA-damaging anticancer drug) treatment to activate such a response was examined. Etoposide-treated control and INSR-KD cultures exhibited changes in morphology that are typically associated with senescence. Specifically, cells displayed a flattened aspect, increased size and an altered ratio of nucleus to cytoplasm, with higher granularity compared to untreated cells (Figure 7A). Interestingly, etoposide treatment of IGF1R-KD cells for 48 h led to a marked decrease in the portion of senescent, compared to control, cells (Figures 7A,B). Thus, etoposide led to a ~5-fold increase (from 18 to 90%) in β-gal-stained control cells, a ~1.17-fold increase (from 70 to 82%) in β-gal-stained INSR-KD cells, and a 48% decrease (from 68 to 36%) in β-gal-stained IGF1R-KD cells (Figure 7B). Furthermore, levels of the p16 and p21 senescence protein markers were enhanced in etoposide-treated control and INSR-KD cells (Figure 7C). In contrast, the levels of both proteins were diminished in etoposide-treated IGF1R-KD cells. Taken together, data suggest that IGF1R has a critical role in the attainment of senescence. The simulated expression of p16 was minimally changed in both INSR-KD and IGF1R-KD cells with respect to controls (Figure 4N), validated by Western blots (Figure 4M). On the other hand, p21 simulation analysis was not affected by the disruption (Figure 4P), validated by Western blots (Figure 4O).

## Discussion

A fundamental question in the area of insulin and IGF1 biology concerns the fact that INSR and IGF1R, even though they share the majority of their downstream cytoplasmic targets and signaling pathways, are yet responsible for mediating distinct physiological roles. Numerous theories have been formulated to explain these discrepancies, including a different tissue distribution of INSR and IGF1R (4), divergent internalization kinetics and subcellular localization of the hormone-receptor complex (34), and dissimilar hormone-receptor affinities (35). Furthermore, a number of substrates that are preferentially activated either by insulin or IGF1 have been identified (36). The present study was aimed at identifying mechanistic differences between INSR and IGF1R using BioNSi, a recently developed bioinformatics tool. This simulation tool allowed us to exploit expression data of breast cancer-derived cell lines with specific disruptions of the INSR or IGF1R genes and their targets in an extended network based on nine KEGG pathways. We thus investigated the differences in protein expression that might be linked to biological attributes of these specific receptor networks.

Using the BioNSi tool, we found 78 genes that were changed between both KDs and control cells. Among them, 76 are downstream genes to INSR and IGF1R. One must note that the BioNSi simulation tool is quite simple in terms of input information available from KEGG. Thus, as the signal is farther down the network, the effect seen may be lost. Therefore, we manually increased the initial expression values of the signal start points (IGF1 and insulin) to 20. At such conditions, the most downstream gene that exhibits a change in expression value during simulations is CHEK2, with 12 edges between it and insulin or IGF1 (the signal molecules) and 11 nodes. During IGF1R-KD simulation, CHEK2 keeps its simulated expression value the same as in the control simulation, up to step 32. Then, its expression value drops to zero and rises up to nine again at step 58. This pattern is a result of the complicated gene network it belongs to. If the initial expression value of IGF1 was lower, such changes at the later steps of the simulation may not have been observed, as the strength of the effect is deteriorating with steps.

In addition to signal expression, one must note that although nine KEGG pathways were selected for this analysis, based on 16 key genes, these represent only a small part of the cellular proteins actually involved in the biological processes investigated. Moreover, BioNSi analysis is based on proteins that are all affecting in a similar manner. As a matter of fact, we know that individual proteins act differently on each other. Hence, it is important to emphasize that the *in silico* BioNSi simulation is based only on partial available molecular information that might mask some of the *in vivo* results. Taking into account these limitations, the simulation analysis investigated 16 genes that were validated correctly by Western blotting and biological assays. Not surprising, but a necessary validation, was the observation that KD of both IGF1R and INSR genes resulted in a sharp reduction in the expression of the selected KD gene in both simulation and Western blots (Figure 3). The expression of an additional 14 genes (by Western blots) further validated simulation results [Figure 4 (eight genes; no change after KD of both genes) and Figure 5 (six genes; reduced after IGF1R KD)]. The reduction in IGF1R may be linked to the key role of this gene in cell cycle and senescence, differently from INSR actions.

Data indicate that a number of genes exhibit the same pattern in Western blot experiments and BioNSi simulation analyses (e.g., STAT3, P53, CASP3, and AMPK). Other genes, however, display different behaviors upon experimental or simulation analyses. Thus, for genes CCND1, ATM, SOD2, CHEK2, HRAS, MTOR, and AKT the differences in simulated expression behavior between KD and control simulations are complex. For example, SOD2 simulated expression values are different between IGF1R-KD and control cells during the first 50 steps of the simulation, but INSR-KD cells show no difference from control. After 50 steps, differences can be seen between control and both disrupted cell types. This phenomenon can be explained by the differences in time frames in which simulation and biological assays are being measured. Simulation starts from a static point of gene expression measurement and expression values are calculated at each step, until 100 steps. At this time, all expression levels have reached a steady-state [either the maximal (9) or minimal (0) expression level]. At the steady-state level, no additional changes are being made. On the other hand, the biological assays take place at a specific point during the lifetime of the cell and cannot reflect all changes in expression as predicted in simulation. Therefore, the results of simulations and Western blotting must be compared in a careful manner. Thus, Western blot results of a specific protein may reflect simulated expression value seen earlier in simulation for upstream genes and/or later for downstream genes (compare CHEK2 and ATM).

Additional differences between results of simulations as compared to Western blots may be due to the selection of specific pathways. In other words, we did not aim to simulate the entire cell, but rather selected important pathways that are responsible for IGF1R and/or INSR downstream responses. However, the fact that many of the interactors in the network are players in multiple cellular pathways, some of which were not the focus of our simulation, might eventually lead to divergent results between both types of analysis. TP53 is the classical example of this situation. Finally, it is important to emphasize that the present study was conducted on MCF7-derived cells, an estrogen receptor positive/progesterone receptor positive/human epidermal growth factor receptor-2 negative adenocarcinoma line derived from a metastatic site. Caution must be exerted when extrapolating data reported here to other cell types, particularly cells with a negative steroid hormone status.

IGF1 has been identified as a key player in cell cycle progression (37–40). Our analysis revealed a number of differences in the proportion of cells at the different stages between IGF1R-KD and INSR-KD cells. For example, the portion of cells at G2M was 37.2% in untreated INSR-KD cells (expressing predominantly an IGF1R) in comparison to 22.6% in IGF1R-KD cells (expressing mainly an INSR). The fact that IGF1 was able to increase the proportion of cells at SubG1 in IGF1R-KD cells (from 0.98 to 2.26%) may suggest that IGF1 is able to cross-activate the INSR, which is the main receptor in these cells. Alternatively, results may indicate the presence of residual IGF1R in IGF1R-KD cells.

The involvement of the insulin-like axis on senescence (at both cellular and organism levels) has been widely reported in recent years (41–45). Our results provide evidence that etoposide, a DNA damaging agent, leads to a significant decrease in the proportion of senescent cells in IGF1R-KD, compared to INSR-KD and control, cells. Levels of the p16 and p21 senescence markers were enhanced in etoposide-treated control and INSR-KD cells but markedly decreased in IGF1R-KD cells. These findings suggest that IGF1R expression and action are critical for progression of senescence. Furthermore, results are in agreement with reports showing that IGF1R can induce senescence in fibroblast (46), keratinocytes (47), and endothelial (45) cells. The biological role of the IGF1R in senescence is reflected in the well-documented involvement of the growth hormone-IGF1 axis in longevity. Evidence has accumulated showing that disruption of this hormonal system is correlated with extended lifespan in various animal species, including nematodes, flies, and mouse (48–50). Female “*Laron*” mice (*GHR/BP*^−/−^) have a 38% longer lifespan than wild-type animals (51). While the impact of specific mutations on human lifespan are difficult to evaluate, studies have identified functionally significant *IGF1R* mutations in centenarians (52).

In summary, despite the limitations of the bioinformatics method and its inability to take all known interactions into account, BioNSi simulations constitute an important addition in insulin/IGF1 research. The tool is aimed at predicting protein interaction effects *in silico* in order to suggest biologically plausible models for experimental evaluation. Our computational analyses, validated by Western blot analyses and biological studies, have identified a number of commonalities and, most importantly, dissimilarities between the IGF1R and INSR pathways that might help explain biological differences between these networks. We believe that comprehensive investigation of the INSR and IGF1R pathways, aided by modern experimental and bioinformatics technologies, might have a translational impact in the area of INSR/IGF1R therapeutic targeting.

## Data Availability Statement

The article contains previously unpublished data. Gene expression data has been deposited at the GEO repository. Accession number is GSE145787.

## Author Contributions

RS, AY, MP-C, and HW conceived of and designed the study. The experimental procedures were performed by RS. The simulation analyses were conducted by AY. The microarray experiments were conducted by TS-L. Additional computational analyses were conducted by AY and MP-C. Statistical analyses were performed by RS. HW and MP-C prepared the manuscript. All authors approved the manuscript.

## Conflict of Interest

The authors declare that the research was conducted in the absence of any commercial or financial relationships that could be construed as a potential conflict of interest.

## Supplementary Material

Supplementary Data Sheet 1 Gene expression file from microarray analysis (MCF7 cells, untreated) used as initial expression values for BioNSi simulations (CSV). Gene expression data has been deposited at the GEO repository. Accession number is GSE145787.

Supplementary Data Sheet 2 Complete network file containing 385 nodes and edges from nine KEGG pathways with normalized expression values from the gene expression file (22,148 genes). The file (CYS) can be opened in cytoscape (https://cytoscape.org/download.html). A pdf file is provided.

Supplementary Data Sheet 3 Unprocessed original scans for all of the figures containing blots.
