# Supplementary material for: Systems Analysis of Insulin and IGF1 Receptors Networks in Breast Cancer Cells Identifies Commonalities and Divergences in Expression Patterns
Source: Front Endocrinol (Lausanne). 2020 Jul 7;11:435. doi: 10.3389/fendo.2020.00435 (PMC7359857; doi:10.3389/fendo.2020.00435)
Supplement: Supplementary Data Sheet 1 — Gene expression file from microarray analysis (MCF7 cells, untreated) used as initial expression values for BioNSi simulations (CSV). Gene expression data has been deposited at the GEO repository. Accession number is GSE145787. [file Data_Sheet_1.PDF]

Gene Symbo	mean expression
A1BG	4.22398
A1CF	3.299304
A2LD1	5.078018
A2M	3.298256
A2ML1	4.952062
A4GALT	5.417778
A4GNT	2.641148
AAA1	2.728034
AAAS	7.696204
AACS	7.333822
AADAC	2.210732
AADACL2	2.516172
AADACL3	3.027668
AADACL4	2.518544
AADAT	3.962876
AAGAB	7.719898
AAK1	6.234414
AAK1	7.50973
AAMP	7.966858
AANAT	4.56886
AARS	10.240344
AARS2	5.51362
AARSD1	6.560062
AASDH	6.067718
AASDHPPT	7.224908
AASS	3.118196
AATF	8.873314
AATK	4.5403
ABAT	6.293326
ABCA1	4.11889
ABCA10	2.025658
ABCA10	2.67258
ABCA11P	2.822934
ABCA12	5.396774
ABCA13	2.970548
ABCA2	5.785908
ABCA3	4.478988
ABCA4	3.159066
ABCA5	5.100754
ABCA6	2.362792
ABCA7	4.963924
ABCA8	2.52348

ABCA9	2.6824
ABCB1	2.914526
ABCB10	5.855198
ABCB11	2.722786
ABCB4	2.867008
ABCB5	2.294098
ABCB6	6.000518
ABCB7	6.088416
ABCB8	6.141244
ABCB8	6.412576
ABCB8	4.951044
ABCB8	4.466754
ABCB9	5.082968
ABCC1	7.048596
ABCC10	6.166118
ABCC11	4.697612
ABCC12	2.540602
ABCC13	2.42448
ABCC2	5.342254
ABCC3	7.364186
ABCC4	6.053034
ABCC5	7.353506
ABCC6	4.276838
ABCC6P2	5.82384
ABCC8	4.036926
ABCC9	2.925878
ABCD1	5.89938
ABCD2	2.459642
ABCD3	7.890234
ABCD4	5.83124
ABCE1	7.847154
ABCF1	8.43882
ABCF1	8.43882
ABCF1	8.43882
ABCF2	7.786348
ABCF3	8.14571
ABCG1	3.868704
ABCG2	4.109452
ABCG4	4.30991
ABCG5	3.168334
ABCG8	3.474062
ABHD1	3.008212
ABHD10	7.397554

ABHD11	6.119662
ABHD12	7.565948
ABHD12B	3.145872
ABHD13	6.556448
ABHD14A	4.67586
ABHD14B	5.053788
ABHD15	5.866858
ABHD2	6.013514
ABHD3	7.261798
ABHD4	6.737108
ABHD4	3.51985
ABHD5	6.518546
ABHD6	5.977224
ABHD8	6.277478
ABI1	8.237864
ABI2	7.367068
ABI3	4.004874
ABI3BP	2.673918
ABL1	7.169922
ABL2	7.658332
ABLIM1	7.458332
ABLIM2	4.842014
ABLIM3	6.595504
ABO	4.412186
ABP1	3.859498
ABR	6.595452
ABRA	2.802956
ABT1	6.55066
ABTB1	5.330326
ABTB2	5.147036
ACAA1	6.874652
ACAA1	2.363648
ACAA2	6.342572
ACACA	8.04873
ACACB	4.437046
ACAD10	5.504382
ACAD11	5.681034
ACAD8	6.157934
ACAD9	9.310592
ACAD9	2.971036
ACADL	2.645278
ACADM	8.6472
ACADS	5.777612

ACADSB	5.726558
ACADVL	9.149692
ACAN	3.670574
ACAP1	4.37501
ACAP2	8.23834
ACAP3	5.656298
ACAT1	6.860926
ACAT2	8.454004
ACBD3	8.839374
ACBD4	6.025892
ACBD5	7.3999
ACBD6	6.569424
ACBD7	5.376232
ACCN1	3.532212
ACCN2	3.903796
ACCN3	4.740962
ACCN4	2.838286
ACCN5	2.498358
ACCS	5.00927
ACCSL	3.31808
ACD	7.250798
ACE	4.452708
ACE2	6.473694
ACER1	3.859918
ACER2	5.441196
ACER3	5.343348
ACHE	4.715788
ACIN1	7.77077
ACIN1	7.526978
ACLY	9.548372
ACMSD	3.121308
ACN9	4.751634
ACO1	7.617062
ACO2	8.920626
ACOT1	6.800536
ACOT11	6.040142
ACOT11	3.689884
ACOT12	2.82703
ACOT13	8.000942
ACOT2	6.569564
ACOT4	6.261224
ACOT6	2.46167
ACOT7	7.165414

ACOT7	7.791206
ACOT8	6.26823
ACOT9	6.423082
ACOX1	6.79131
ACOX2	4.199696
ACOX3	6.73267
ACOXL	4.53183
ACP1	9.014698
ACP2	7.691258
ACP5	3.80091
ACP6	7.359006
ACPL2	4.779022
ACPP	5.0267
ACPT	5.055272
ACR	3.74044
ACR	4.080604
ACRBP	4.284202
ACRC	3.056044
ACRV1	2.616474
ACSBG1	3.562046
ACSBG2	2.883534
ACSF2	6.895876
ACSF3	6.06613
ACSL1	8.928002
ACSL3	9.14689
ACSL4	4.906022
ACSL5	7.704078
ACSL6	3.205038
ACSM1	2.958312
ACSM2A	2.725972
ACSM2A	2.764162
ACSM2B	2.73721
ACSM3	2.863482
ACSM5	3.500776
ACSS1	4.068696
ACSS2	7.87377
ACSS3	2.610584
ACTA1	6.021244
ACTA2	3.111114
ACTA2	4.504056
ACTB	12.38596
ACTBL2	2.649654
ACTC1	3.937498

ACTG1	12.18626
ACTG2	3.465488
ACTL6A	8.527954
ACTL6B	3.3792
ACTL7A	2.86243
ACTL7B	4.480704
ACTL8	4.01429
ACTL9	5.43939
ACTN1	9.454472
ACTN2	3.621022
ACTN3	3.279656
ACTN4	11.07726
ACTR10	7.35432
ACTR1A	7.446186
ACTR1B	7.36582
ACTR2	10.59582
ACTR3	10.299254
ACTR3B	6.294894
ACTR3BP2	2.284388
ACTR3BP2	2.261132
ACTR3BP2	2.311066
ACTR3BP2	2.389972
ACTR3C	7.140908
ACTR5	5.955712
ACTR6	5.685162
ACTR8	6.578246
ACTRT1	2.783892
ACTRT2	4.604154
ACVR1	5.696344
ACVR1B	6.371626
ACVR1C	3.637702
ACVR2A	5.45506
ACVR2B	4.53782
ACVRL1	4.990062
ACY1	6.121684
ACY3	4.542684
ACYP1	5.121452
ACYP2	5.429316
ADA	6.558922
ADAD1	2.335776
ADAD2	5.630502
ADAD2	4.337812
ADAL	6.225726

ADAM10	9.501796
ADAM11	4.417522
ADAM12	5.629802
ADAM15	7.409108
ADAM17	8.399514
ADAM18	2.57556
ADAM19	3.702606
ADAM2	2.503482
ADAM20	3.559784
ADAM21	2.890656
ADAM21	2.881214
ADAM22	4.630166
ADAM23	2.515944
ADAM28	2.791944
ADAM29	2.565044
ADAM30	2.440784
ADAM32	2.866158
ADAM33	4.99569
ADAM3A	2.43054
ADAM5P	2.150768
ADAM6	2.672676
ADAM7	2.576084
ADAM8	5.003614
ADAM9	9.372592
ADAMDEC1	2.511808
ADAMTS1	3.199454
ADAMTS10	3.996914
ADAMTS12	4.521592
ADAMTS13	4.686762
ADAMTS14	3.819448
ADAMTS15	4.364014
ADAMTS16	4.146476
ADAMTS17	4.387928
ADAMTS18	3.04574
ADAMTS19	3.04529
ADAMTS2	4.56565
ADAMTS20	2.78112
ADAMTS3	3.369484
ADAMTS4	3.860912
ADAMTS5	2.95943
ADAMTS6	2.991364
ADAMTS7	6.041016
ADAMTS8	4.346486

ADAMTS9	4.431014
ADAMTSL1	2.911516
ADAMTSL1	3.496396
ADAMTSL2	4.515326
ADAMTSL3	4.212784
ADAMTSL4	4.190708
ADAMTSL4	3.611156
ADAMTSL5	4.980248
ADAP1	7.338512
ADAP2	4.863984
ADAR	9.38
ADARB1	5.589848
ADARB2	4.579478
ADAT1	8.044482
ADAT2	6.021854
ADAT3	4.984618
ADC	4.629158
ADCK1	5.592652
ADCK2	5.351178
ADCK3	5.67341
ADCK4	6.10593
ADCK5	6.048822
ADCY1	4.448018
ADCY10	2.637694
ADCY2	3.814434
ADCY3	7.165668
ADCY4	3.898324
ADCY5	6.640882
ADCY6	7.452404
ADCY7	5.390212
ADCY8	3.174774
ADCY9	5.302028
ADCYAP1	3.340482
ADCYAP1R1	3.056804
ADD1	7.819186
ADD2	4.098344
ADD3	6.129274
ADH1A	3.465776
ADH1B	2.662706
ADH1C	2.760016
ADH4	2.5294
ADH5	7.531466
ADH6	2.383848

ADH7	2.351206
ADHFE1	3.150306
ADI1	8.65045
ADIG	3.811386
ADIPOQ	3.392656
ADIPOR1	9.318206
ADIPOR2	7.849926
ADK	7.00993
ADM	7.968162
ADM2	5.861242
ADNP	7.622032
ADNP2	5.604736
ADO	5.742602
ADORA1	5.924988
ADORA2A	4.870886
ADORA2B	8.259268
ADORA3	2.892396
ADPGK	6.511854
ADPRH	3.421476
ADPRHL1	5.124762
ADPRHL2	6.37357
ADRA1A	2.490232
ADRA1B	4.652048
ADRA1D	5.121288
ADRA2A	5.010546
ADRA2B	4.539652
ADRA2C	6.277064
ADRB1	5.482932
ADRB2	3.570982
ADRB3	3.37913
ADRBK1	6.902444
ADRBK2	4.887932
ADRM1	9.099586
ADSL	7.606678
ADSS	7.617174
ADSSL1	6.372578
AEBP1	4.874262
AEBP2	8.40865
AEN	5.662728
AES	8.196556
AFAP1	4.990686
AFAP1	8.184692
AFAP1-AS	7.33394

AFAP1L1	5.483298
AFAP1L2	4.907362
AFF1	7.635716
AFF2	3.083662
AFF3	3.22521
AFF4	8.481764
AFG3L1P	5.353544
AFG3L2	8.790242
AFM	2.260888
AFMID	5.804676
AFP	2.6829
AFTPH	7.493722
AGA	6.685044
AGAP1	4.473002
AGAP11	5.654364
AGAP2	3.942414
AGAP3	6.360212
AGAP4	6.118782
AGAP4	6.574734
AGAP4	6.456456
AGAP5	5.978704
AGAP5	6.583838
AGAP5	6.328028
AGAP6	6.280364
AGAP7	6.250034
AGAP9	6.645474
AGAP9	6.645474
AGBL1	3.232146
AGBL2	3.796922
AGBL3	2.832882
AGBL4	2.996668
AGBL5	6.043664
AGER	4.7734
AGER	4.802954
AGER	4.778474
AGFG1	9.480008
AGFG2	5.493216
AGGF1	6.060732
AGK	6.680886
AGL	5.363436
AGMAT	4.894108
AGPAT1	7.41226
AGPAT1	7.41226

AGPAT1	7.41226
AGPAT2	8.51761
AGPAT3	6.600602
AGPAT4	3.918446
AGPAT5	7.086818
AGPAT6	8.77696
AGPAT9	8.45908
AGPHD1	5.445846
AGPS	7.992092
AGR2	8.884474
AGR3	2.306014
AGRN	7.107656
AGRP	5.402186
AGT	3.1543
AGTPBP1	4.942152
AGTR1	2.898492
AGTR2	2.607834
AGTRAP	7.166742
AGXT	4.328992
AGXT2	3.418436
AGXT2L1	2.55186
AGXT2L2	5.899596
AHCTF1	7.312364
AHCTF1	7.676614
AHCY	8.230302
AHCYL1	8.791432
AHCYL2	6.379378
AHDC1	5.582502
AHI1	4.903488
AHNAK	9.5666
AHNAK2	6.486704
AHR	8.15494
AHRR	5.530466
AHSA1	8.707422
AHSA2	6.240142
AHSG	2.81714
AHSP	2.941954
AICDA	3.413146
AIDA	8.094106
AIDA	4.46336
AIF1	2.94533
AIF1	2.94533
AIF1	2.94533

AIF1L	6.909844
AIFM1	9.514606
AIFM2	6.472674
AIFM3	4.84312
AIG1	7.796588
AIM1	9.020286
AIM1L	6.446888
AIM2	2.474402
AIMP1	5.87341
AIMP2	8.818
AIP	7.382012
AIPL1	4.607898
AIRE	5.277098
AJAP1	3.128362
AK1	8.102978
AK2	7.633312
AK2	10.59106
AK3	6.878956
AK4	5.457384
AK4	6.932226
AK5	2.570192
AK7	2.905042
AKAP1	8.060518
AKAP10	7.183664
AKAP11	5.864488
AKAP12	3.093312
AKAP13	5.889762
AKAP14	2.305048
AKAP3	3.71591
AKAP4	2.914412
AKAP5	2.963682
AKAP6	3.918052
AKAP7	3.191104
AKAP8	8.267146
AKAP8L	8.272778
AKAP9	4.099866
AKD1	2.541386
AKD1	2.40878
AKD1	3.760928
AKIRIN1	8.378648
AKIRIN2	8.000138
AKNA	4.928188
AKNAD1	3.1079

AKR1A1	8.509936
AKR1B1	9.770596
AKR1B10	5.898286
AKR1C1	6.090242
AKR1C2	10.33438
AKR1C3	9.400754
AKR1C4	2.622482
AKR1CL1	3.211478
AKR1D1	2.982372
AKR1E2	7.163926
AKR7A2	7.916174
AKR7A2P1	3.88122
AKR7A3	5.344706
AKR7L	4.09159
AKT1	7.618932
AKT1S1	6.317226
AKT2	8.39807
AKT3	6.35535
AKTIP	7.19404
ALAD	6.481286
ALAS1	8.8374
ALAS2	3.506666
ALB	2.62262
ALCAM	6.744922
ALDH16A1	6.334988
ALDH18A1	8.630692
ALDH1A1	4.127704
ALDH1A2	2.959744
ALDH1A3	9.464516
ALDH1B1	7.766086
ALDH1L1	4.098452
ALDH1L2	3.907024
ALDH2	4.31676
ALDH3A1	7.552474
ALDH3A2	8.982328
ALDH3B1	7.330752
ALDH3B2	5.002598
ALDH4A1	5.174674
ALDH5A1	5.650742
ALDH6A1	7.577778
ALDH7A1	9.46749
ALDH8A1	2.75559
ALDH9A1	2.85124

ALDH9A1	9.026892
ALDOA	8.74088
ALDOAP2	4.887332
ALDOB	3.09157
ALDOC	5.22023
ALG1	7.385654
ALG10	5.947374
ALG10B	5.936756
ALG11	6.20255
ALG12	6.788932
ALG13	3.886644
ALG13	3.0236
ALG14	5.502694
ALG1L	5.364852
ALG2	7.906262
ALG3	10.024726
ALG5	6.784406
ALG6	6.334646
ALG8	7.948748
ALG9	7.601642
ALK	2.98966
ALKBH1	6.533522
ALKBH2	6.438992
ALKBH3	7.323586
ALKBH4	5.359434
ALKBH5	8.342952
ALKBH6	7.915418
ALKBH7	5.74315
ALKBH8	4.877442
ALLC	2.719794
ALMS1	5.287736
ALMS1P	3.147362
ALOX12	3.691604
ALOX12B	4.484548
ALOX12P2	5.529624
ALOX15	4.90758
ALOX15B	4.432966
ALOX5	8.38366
ALOX5AP	3.975692
ALOXE3	3.733598
ALPI	5.254394
ALPK1	6.176088
ALPK2	2.82188

ALPK3	4.06351
ALPL	3.440556
ALPP	6.003382
ALPPL2	7.198214
ALS2	5.676344
ALS2CL	6.18419
ALS2CR11	2.358712
ALS2CR12	2.793648
ALS2CR4	5.7983
ALS2CR8	3.016826
ALX1	3.024264
ALX3	4.643244
ALX4	4.108306
AMAC1	4.589928
AMAC1L2	5.253028
AMAC1L2	2.026832
AMAC1L3	4.738538
AMACR	6.360198
AMBN	2.435772
AMBP	3.846302
AMBRA1	7.189866
AMD1	8.475102
AMDHD1	5.06881
AMDHD2	5.9071
AMELX	2.699644
AMELY	2.642272
AMFR	7.966166
AMH	6.05288
AMHR2	3.715296
AMICA1	2.727184
AMIGO1	5.33525
AMIGO2	5.624074
AMIGO3	4.631528
AMMECR1	7.60513
AMMECR1L	7.314198
AMN	6.354036
AMN1	4.807356
AMOT	3.243012
AMOTL1	7.808158
AMOTL2	6.695414
AMPD1	2.61583
AMPD2	5.272274
AMPD3	4.910056

AMPH	2.458596
AMT	4.05157
AMTN	2.706144
AMY1A	2.488634
AMY1A	2.488634
AMY1A	2.488634
AMY2A	2.314338
AMY2B	2.838668
AMZ1	5.734764
AMZ2	8.948276
AMZ2P1	5.818976
ANAPC1	6.474962
ANAPC1	6.81907
ANAPC1	8.27252
ANAPC1	6.217846
ANAPC1	7.796052
ANAPC10	5.612916
ANAPC11	6.009542
ANAPC11	5.86544
ANAPC13	7.343082
ANAPC16	8.09491
ANAPC2	6.273764
ANAPC4	6.197526
ANAPC5	8.078902
ANAPC7	8.225174
ANG	4.045486
ANGEL1	6.183176
ANGEL2	6.624536
ANGPT1	2.709944
ANGPT2	2.835114
ANGPT4	3.623938
ANGPTL1	2.267628
ANGPTL2	3.439146
ANGPTL3	2.162674
ANGPTL4	4.535078
ANGPTL5	2.047168
ANGPTL6	4.805636
ANGPTL7	2.92656
ANK1	4.049278
ANK2	2.734272
ANK3	4.786928
ANKAR	2.998206
ANKDD1A	4.912558

ANKFN1	2.519956
ANKFY1	7.692356
ANKH	5.46776
ANKH	7.391094
ANKHD1-EIF4	7.176678
ANKIB1	7.891516
ANKK1	4.125292
ANKLE1	4.525302
ANKLE2	7.236298
ANKMY1	4.23051
ANKMY2	6.990542
ANKRA2	4.116972
ANKRD1	5.226664
ANKRD10	7.67449
ANKRD11	6.540856
ANKRD11	4.671916
ANKRD12	4.442892
ANKRD13A	8.631092
ANKRD13B	4.986678
ANKRD13C	6.83188
ANKRD13D	5.526322
ANKRD16	3.678138
ANKRD17	7.55858
ANKRD18A	3.226668
ANKRD18B	3.46209
ANKRD19	2.555606
ANKRD2	5.518296
ANKRD20A1	3.08619
ANKRD20A2	2.94148
ANKRD20A3	3.170312
ANKRD20A3	3.170312
ANKRD20A5	2.440796
ANKRD20B	2.254828
ANKRD20B	2.828832
ANKRD20B	2.639466
ANKRD20B	2.810922
ANKRD20B	3.20119
ANKRD20B	4.260012
ANKRD20B	2.311016
ANKRD20B	2.324916
ANKRD22	6.16409
ANKRD23	4.655976
ANKRD24	4.376518

ANKRD26	4.590482
ANKRD26P1	2.623506
ANKRD27	8.192322
ANKRD28	7.686458
ANKRD29	3.515396
ANKRD30A	2.21871
ANKRD30B	2.044838
ANKRD30B	2.078062
ANKRD30BL	4.7691
ANKRD30BP2	2.783512
ANKRD31	2.937504
ANKRD32	3.244714
ANKRD32	2.27107
ANKRD33	3.337212
ANKRD33B	5.957462
ANKRD34A	4.183604
ANKRD34B	2.550538
ANKRD34C	2.95749
ANKRD35	3.620316
ANKRD36	4.204114
ANKRD36B	5.495082
ANKRD36B	6.28012
ANKRD36B	6.298364
ANKRD36B	5.817808
ANKRD36B	4.426542
ANKRD36BP1	2.869242
ANKRD37	4.220406
ANKRD39	6.406896
ANKRD40	7.42578
ANKRD42	3.99134
ANKRD42	7.297134
ANKRD43	5.352424
ANKRD44	4.17333
ANKRD45	2.619534
ANKRD46	5.44034
ANKRD49	5.162548
ANKRD5	5.627776
ANKRD50	7.52041
ANKRD52	6.654188
ANKRD53	4.188882
ANKRD54	5.976628
ANKRD55	2.72089
ANKRD56	4.333788

ANKRD57	6.498274
ANKRD58	4.013916
ANKRD6	3.961566
ANKRD60	5.221168
ANKRD61	3.309032
ANKRD62	2.598268
ANKRD62	2.253514
ANKRD7	2.205426
ANKRD9	5.109628
ANKS1A	6.627258
ANKS1B	2.876028
ANKS3	5.20954
ANKS4B	3.454702
ANKS6	5.837102
ANKZF1	5.06634
ANLN	9.184094
ANO1	7.088068
ANO10	7.388986
ANO2	3.809812
ANO3	2.471394
ANO4	2.721222
ANO5	2.297526
ANO6	7.895154
ANO7	4.56818
ANO8	4.855838
ANO9	6.147932
ANP32A	9.172818
ANP32A	8.020692
ANP32B	9.357516
ANP32C	6.336098
ANP32D	2.439158
ANP32E	6.91867
ANPEP	9.581434
ANTXR1	6.747722
ANTXR2	5.81509
ANTXRL	2.528218
ANUBL1	4.737416
ANXA1	10.79534
ANXA10	2.695248
ANXA11	8.735044
ANXA13	2.727606
ANXA2	11.58492
ANXA2P1	6.77006

ANXA2P2	11.3934
ANXA2P3	4.33499
ANXA3	8.70232
ANXA4	8.680024
ANXA5	10.22118
ANXA6	5.90794
ANXA7	8.167386
ANXA8	7.315832
ANXA8L1	8.802572
ANXA8L2	7.294286
ANXA9	6.66012
AOAH	2.605192
AOC2	4.301734
AOC3	4.581214
AOC3	5.992626
AOX1	3.493632
AP1AR	6.065914
AP1B1	7.853634
AP1G1	8.852766
AP1G2	8.307878
AP1M1	6.827982
AP1M2	8.755078
AP1S1	8.038616
AP1S2	5.054504
AP1S2	3.341226
AP1S3	6.185312
AP2A1	7.092362
AP2A2	6.388576
AP2B1	9.38293
AP2M1	10.4586
AP2S1	8.853672
AP3B1	7.385754
AP3B2	3.220222
AP3D1	8.18184
AP3M1	7.497768
AP3M2	7.243368
AP3S1	6.460976
AP3S2	8.512864
AP4B1	5.62164
AP4E1	6.190066
AP4M1	6.056174
AP4S1	4.55728
APAF1	6.404222

APBA1	4.02229
APBA2	5.947466
APBA3	4.546784
APBB1	3.046882
APBB1IP	2.721536
APBB2	7.93927
APBB3	6.023784
APC	4.09949
APC2	4.507706
APCDD1	5.175198
APCDD1L	4.946312
APCS	3.031904
APEH	8.230014
APEX1	9.838196
APEX2	6.44512
APH1A	7.90247
APH1B	4.73764
API5	8.538278
APIP	6.153564
APITD1-COR	4.625298
APLF	2.505278
APLN	4.536708
APLNR	3.512052
APLP1	5.30014
APLP2	10.63288
APOA1	4.742892
APOA1	4.827042
APOA1BP	9.520576
APOA2	3.547146
APOA4	3.890466
APOA4	4.502958
APOA5	4.049626
APOB	2.910542
APOB48R	3.99975
APOBEC1	3.791124
APOBEC2	2.872542
APOBEC3A	4.06736
APOBEC3B	9.426674
APOBEC3C	6.7948
APOBEC3D	5.45509
APOBEC3F	6.17701
APOBEC3G	3.14152
APOBEC3H	3.627694

APOBEC4	2.696766
APOC1	3.734822
APOC2	4.753246
APOC3	3.894444
APOC3	3.86026
APOC4	4.13598
APOC4	2.897082
APOD	8.59205
APOE	4.60977
APOF	2.855574
APOH	2.360944
APOL1	7.411818
APOL2	6.198176
APOL3	5.90772
APOL4	3.331578
APOL5	3.900202
APOL6	7.915506
APOLD1	6.433958
APOM	4.29613
APOM	4.29613
APOM	4.29613
APOO	6.856172
APOOL	4.934904
APP	11.0786
APPBP2	7.678974
APPL1	7.169984
APPL2	6.109894
APRT	6.778456
APTX	5.591128
AQP1	4.868384
AQP10	3.537218
AQP11	3.31754
AQP12A	5.016596
AQP12A	4.855396
AQP2	3.955178
AQP3	8.69551
AQP4	2.471426
AQP5	4.234114
AQP6	3.669858
AQP7	5.262504
AQP7P1	4.146852
AQP7P1	4.152664
AQP7P1	4.093648

AQP7P1	4.073684
AQP8	3.811268
AQP9	3.240588
AQPEP	2.740058
AQPEP	3.265294
AQR	8.193352
AR	3.24766
ARAF	7.05028
ARAF2P	3.706236
ARAF2P	3.706236
ARAP1	6.670762
ARAP2	5.147562
ARAP3	4.88694
ARC	5.505356
ARCN1	8.598484
AREG	7.357232
AREG	7.86122
ARF1	10.30974
ARF3	9.806498
ARF4	10.30058
ARF5	7.139846
ARF6	9.203158
ARFGAP1	6.43435
ARFGAP2	8.563978
ARFGAP3	6.758444
ARFGEF1	6.70264
ARFGEF2	6.31285
ARFIP1	7.893266
ARFIP2	7.446646
ARFRP1	6.301972
ARG1	2.636984
ARG2	5.088632
ARGFX	2.740732
ARGLU1	8.59226
ARHGAP1	8.55707
ARHGAP10	5.66575
ARHGAP10	4.46801
ARHGAP11A	8.012298
ARHGAP11B	6.884196
ARHGAP12	7.610148
ARHGAP15	2.489714
ARHGAP17	5.490746
ARHGAP18	7.056264

ARHGAP19	5.786608
ARHGAP20	2.850478
ARHGAP21	7.597574
ARHGAP22	3.34598
ARHGAP23	7.714852
ARHGAP23	6.557992
ARHGAP24	2.828888
ARHGAP25	2.958194
ARHGAP26	8.009298
ARHGAP27	5.952762
ARHGAP28	2.380774
ARHGAP29	8.302314
ARHGAP30	3.941574
ARHGAP31	5.760948
ARHGAP32	7.63102
ARHGAP33	5.06613
ARHGAP36	3.178324
ARHGAP39	5.288466
ARHGAP4	4.906628
ARHGAP40	3.88298
ARHGAP42	5.062408
ARHGAP44	3.66841
ARHGAP5	6.969528
ARHGAP6	2.956722
ARHGAP9	3.840396
ARHGDIA	7.552444
ARHGDIA	7.309874
ARHGDIB	9.859964
ARHGDIG	3.318426
ARHGDIG	4.201056
ARHGEF1	6.141316
ARHGEF10	5.63689
ARHGEF10	4.482788
ARHGEF10L	5.82071
ARHGEF11	6.54921
ARHGEF12	8.322408
ARHGEF15	4.78176
ARHGEF16	5.675364
ARHGEF17	5.08935
ARHGEF18	6.965028
ARHGEF19	5.393292
ARHGEF2	7.340586
ARHGEF25	4.444522

ARHGEF26	2.36913
ARHGEF3	5.497836
ARHGEF37	6.535758
ARHGEF38	5.873924
ARHGEF4	5.08554
ARHGEF40	4.649608
ARHGEF5	7.176244
ARHGEF5	7.130526
ARHGEF6	3.046968
ARHGEF7	7.078236
ARHGEF9	5.800318
ARID1A	6.491572
ARID1B	5.423958
ARID2	6.559408
ARID3A	5.547752
ARID3B	4.590622
ARID3B	3.72545
ARID3C	3.937316
ARID4A	5.375534
ARID4B	5.889998
ARID5A	4.28759
ARID5A	4.13231
ARID5B	7.079128
ARIH1	8.23425
ARIH2	7.422338
ARIH2	5.486184
ARL1	7.148162
ARL10	3.898422
ARL11	3.372268
ARL13A	2.332362
ARL13B	5.86351
ARL14	4.528096
ARL15	3.111308
ARL17A	7.161072
ARL17A	7.16291
ARL17A	7.13484
ARL2	8.647226
ARL2BP	8.160202
ARL3	4.973496
ARL4A	4.395266
ARL4C	7.17359
ARL4D	6.706114
ARL5A	6.99872

ARL5B	7.240826
ARL5C	3.032212
ARL6	3.198036
ARL6IP1	11.90868
ARL6IP4	7.225388
ARL6IP5	9.64181
ARL6IP6	7.477088
ARL8A	7.458434
ARL8B	8.255374
ARL9	2.65952
ARMC1	7.896088
ARMC10	7.181892
ARMC10	6.97349
ARMC2	3.757736
ARMC3	2.60284
ARMC4	3.108768
ARMC5	4.531716
ARMC6	7.255622
ARMC7	6.402662
ARMC8	7.68403
ARMC9	5.212988
ARMCX1	3.005936
ARMCX2	3.461246
ARMCX3	5.695686
ARMCX4	3.05105
ARMCX5	3.944238
ARMCX6	6.040274
ARMCX6	6.234436
ARNT	7.114554
ARNT2	3.63062
ARNTL	4.949928
ARNTL2	7.530184
ARPC1A	9.954298
ARPC1B	9.954358
ARPC2	9.68113
ARPC3	9.555386
ARPC4	9.337772
ARPC5	9.469218
ARPC5L	7.355344
ARPM1	4.469446
ARPP19	7.929126
ARPP19	7.076046
ARPP21	2.776734

ARR3	3.055346
ARRB1	7.090216
ARRB2	6.459558
ARRDC1	7.94318
ARRDC2	6.653194
ARRDC3	7.608286
ARRDC4	5.689878
ARSA	4.921568
ARSB	4.557368
ARSD	5.397388
ARSE	2.919616
ARSF	3.050172
ARSG	6.14609
ARSH	3.732052
ARSI	3.806468
ARSJ	5.515116
ARSK	4.21885
ART1	4.454698
ART3	2.335214
ART4	2.738066
ART5	4.592178
ARTN	5.013002
ARV1	7.402062
ARVCF	5.59859
ARVP6125	2.70587
ARX	5.616792
AS3MT	4.827214
ASAH1	8.586588
ASAH2	4.430252
ASAH2	3.49134
ASAH2	3.350332
ASAM	8.831098
ASAP1	8.40608
ASAP2	7.308522
ASAP3	5.792626
ASB1	4.492082
ASB10	3.874856
ASB11	2.689878
ASB12	3.042464
ASB13	6.547948
ASB14	2.644366
ASB15	2.531472
ASB16	5.016138

ASB17	2.277634
ASB18	3.559756
ASB2	2.838494
ASB3	7.96165
ASB4	2.76308
ASB5	2.797154
ASB6	6.67822
ASB7	6.300706
ASB8	6.784752
ASB9	5.40243
ASCC1	7.525464
ASCC2	7.661064
ASCC3	7.192464
ASCL1	3.35017
ASCL2	3.721638
ASCL3	2.654276
ASCL4	2.774572
ASCL5	3.897466
ASF1A	6.504362
ASF1B	9.713624
ASGR1	4.127454
ASGR2	3.532118
ASH1L	7.469206
ASH2L	8.994286
ASIP	4.138554
ASL	7.353324
ASMT	5.054378
ASMT	5.054378
ASMTL	6.596164
ASMTL	6.540014
ASMTL-AS	4.175618
ASMTL-AS	4.1061
ASNA1	9.036856
ASNS	7.539228
ASNSD1	6.014404
ASPA	2.056938
ASPDH	4.321072
ASPG	5.2334
ASPH	7.319994
ASPHD1	5.550266
ASPHD2	3.827874
ASPM	6.755706
ASPN	2.17035

ASPRV1	4.389294
ASPSCR1	6.277122
ASRGL1	5.704948
ASS1	10.70164
ASTE1	4.520116
ASTL	4.286562
ASTN1	4.169076
ASTN2	4.13141
ASXL1	6.825766
ASXL2	6.332408
ASXL3	2.809816
ASZ1	2.46966
ATAD1	2.39648
ATAD2	8.449318
ATAD2B	4.695962
ATAD3A	6.958236
ATAD3B	6.885322
ATAD3C	4.88184
ATAD5	5.89978
ATAT1	5.019924
ATAT1	4.932726
ATAT1	5.019924
ATCAY	3.728458
ATE1	8.302952
ATF1	7.892762
ATF2	8.03667
ATF3	4.499678
ATF4	7.075866
ATF5	5.725622
ATF6	8.330106
ATF6B	8.016592
ATF6B	7.822332
ATF7	6.514042
ATF7IP	8.24256
ATF7IP2	2.345848
ATG10	5.48007
ATG12	5.511716
ATG13	8.012532
ATG14	6.715646
ATG16L1	6.875084
ATG16L2	4.684326
ATG2A	5.885714
ATG2B	5.062302

ATG2B	4.099688
ATG3	9.180716
ATG4A	5.58256
ATG4A	2.973232
ATG4B	6.063172
ATG4B	6.101204
ATG4C	4.930012
ATG4D	6.142836
ATG5	7.348388
ATG7	8.713514
ATG9A	5.968364
ATG9B	4.57663
ATHL1	4.30611
ATIC	8.576308
ATL1	4.273034
ATL2	8.148698
ATL3	8.222698
ATL3	8.035788
ATM	5.101078
ATMIN	7.050782
ATMIN	7.456588
ATN1	6.737366
ATOH1	4.118414
ATOH7	3.55485
ATOH8	3.917386
ATOX1	7.575906
ATP10A	5.347552
ATP10B	3.436608
ATP10D	6.99752
ATP11A	6.079144
ATP11B	7.20742
ATP11C	5.766138
ATP12A	3.01126
ATP13A1	6.985654
ATP13A2	5.778822
ATP13A3	9.270146
ATP13A4	2.602016
ATP13A5	2.617436
ATP1A1	10.53826
ATP1A2	3.972566
ATP1A3	5.898006
ATP1A4	3.268478
ATP1B1	10.21931

ATP1B2	4.25073
ATP1B3	8.215002
ATP1B4	3.2454
ATP2A1	3.32334
ATP2A2	9.21986
ATP2A3	4.504634
ATP2B1	7.553662
ATP2B2	4.213798
ATP2B3	3.855882
ATP2B4	8.054634
ATP2C1	7.880418
ATP2C2	6.3957
ATP4A	3.652642
ATP4B	3.758508
ATP5A1	8.937988
ATP5B	11.7631
ATP5C1	10.34162
ATP5D	7.893156
ATP5E	7.464248
ATP5EP2	7.896038
ATP5F1	5.339614
ATP5G1	8.079084
ATP5G2	7.520562
ATP5G3	4.393754
ATP5H	10.117218
ATP5I	7.801682
ATP5J	6.915422
ATP5J2	8.89211
ATP5L	9.732078
ATP5L2	3.86592
ATP5O	9.258126
ATP5S	5.31458
ATP5SL	7.697162
ATP6AP1	8.88926
ATP6AP1L	4.20828
ATP6AP2	8.534838
ATP6V0A1	8.682526
ATP6V0A2	6.287614
ATP6V0A4	3.622464
ATP6V0B	8.638998
ATP6V0C	8.943912
ATP6V0D1	8.62442
ATP6V0D2	2.880886

ATP6V0E1	10.33722
ATP6V0E2	5.958768
ATP6V0E2	5.134336
ATP6V1A	9.07171
ATP6V1B1	7.460642
ATP6V1B2	8.962796
ATP6V1C1	7.669478
ATP6V1D	8.013614
ATP6V1E1	8.799492
ATP6V1E2	4.356682
ATP6V1F	7.687868
ATP6V1G1	8.437704
ATP6V1G2	2.774036
ATP6V1G2	3.251202
ATP6V1G2	2.774036
ATP6V1G3	2.738912
ATP6V1H	7.638408
ATP7A	4.474672
ATP7B	3.608194
ATP8A1	2.553826
ATP8A2	3.000812
ATP8B1	3.955416
ATP8B1	6.224826
ATP8B2	4.370334
ATP8B3	4.070828
ATP8B4	2.505302
ATP8B5P	2.556398
ATP9A	7.846356
ATP9B	4.847612
ATPAF1	5.713908
ATPAF2	6.790268
ATPBD4	5.686136
ATPIF1	6.941116
ATR	6.11979
ATRIP	5.836476
ATRN	7.259688
ATRNL1	2.655164
ATRX	4.918666
ATRX	4.460592
ATXN1	5.381066
ATXN10	8.072484
ATXN2	8.366368
ATXN2L	7.330402

ATXN3	6.329106
ATXN3L	2.354302
ATXN7	6.821708
ATXN7L1	4.120116
ATXN7L1	5.154432
ATXN7L1	4.451394
ATXN7L1	4.698576
ATXN7L1	4.400906
ATXN7L1	4.784962
ATXN7L2	4.397812
ATXN7L3	6.399036
ATXN7L3B	8.304538
AUH	6.451446
AUP1	8.009876
AURKA	9.227136
AURKAIP1	8.232966
AURKAIP1	8.29832
AURKB	8.241036
AURKC	4.532774
AUTS2	3.673122
AVEN	5.999528
AVIL	3.73081
AVIL	4.707634
AVL9	6.544358
AVP	4.514588
AVPI1	7.097786
AVPR1A	3.13574
AVPR1B	2.921252
AVPR2	3.899118
AWAT1	3.392574
AWAT2	3.386318
AXIN1	5.969864
AXIN2	4.013978
AXL	6.537128
AZGP1	7.08489
AZI1	5.173672
AZI2	5.843256
AZIN1	9.37828
AZU1	4.461566
B2M	11.01058
B3GALNT1	4.90536
B3GALNT2	8.114102
B3GALT1	2.53638

B3GALT2	2.303434
B3GALT4	4.024412
B3GALT4	4.024412
B3GALT4	4.024412
B3GALT5	3.954414
B3GALT6	6.171146
B3GALTL	6.10472
B3GAT1	3.759528
B3GAT2	3.988588
B3GAT3	6.625604
B3GNT1	4.935714
B3GNT2	7.35272
B3GNT3	7.638216
B3GNT4	4.53597
B3GNT5	6.780074
B3GNT6	5.34101
B3GNT7	5.446162
B3GNT8	3.887496
B3GNT9	4.614974
B3GNTL1	4.47265
B3GNTL1	4.330192
B4GALNT1	3.439018
B4GALNT2	3.659542
B4GALNT3	6.665274
B4GALNT4	5.226632
B4GALT1	8.44845
B4GALT2	7.422858
B4GALT3	7.392432
B4GALT4	7.591452
B4GALT5	9.741956
B4GALT6	3.856182
B4GALT7	6.361422
B9D1	5.443478
B9D2	4.93045
BAALC	3.780656
BAAT	2.718468
BACE1	5.100318
BACE2	7.250024
BACH1	5.540754
BACH2	2.976738
BAD	6.614798
BAG1	7.101144
BAG2	6.57322

BAG3	7.566354
BAG4	6.086216
BAG5	6.769978
BAGE2	2.114032
BAHCC1	5.245156
BAHD1	5.977934
BAI1	5.055432
BAI2	5.078908
BAI3	2.854776
BAIAP2	6.838182
BAIAP2L1	8.823
BAIAP2L2	4.790364
BAIAP3	4.435452
BAK1	7.616426
BAMBI	10.31851
BANF1	9.086274
BANF2	3.02573
BANK1	2.717764
BANP	5.44725
BAP1	7.004688
BARD1	7.012438
BARHL1	5.27641
BARHL2	4.883444
BARX1	5.439092
BARX2	6.920904
BASE	2.850776
BASP1	6.07833
BAT1	10.55258
BAT1	10.61542
BAT1	10.60222
BAT2	7.745912
BAT2	7.627162
BAT2L1	9.532518
BAT2L1	8.307072
BAT2L1	2.952608
BAT2L2	8.02127
BAT3	8.01347
BAT3	8.050144
BAT3	8.050144
BAT4	5.517472
BAT5	6.058592
BAT5	6.060678
BATF	5.733986

BATF2	5.28603
BATF3	4.00674
BAX	7.22337
BAZ1A	6.601272
BAZ1B	8.756898
BAZ2A	7.501834
BAZ2B	5.222344
BBC3	5.75515
BBOX1	2.370628
BBS1	5.4005
BBS10	4.17344
BBS12	4.587024
BBS2	7.51243
BBS4	5.386724
BBS5	2.862614
BBS7	4.50323
BBS9	4.429084
BBX	7.131206
BCAM	6.963652
BCAN	4.615612
BCAP29	5.480588
BCAP31	8.964178
BCAR1	6.545114
BCAR3	6.565318
BCAS1	5.027484
BCAS2	7.490528
BCAS3	6.36838
BCAS4	6.330628
BCAT1	2.601348
BCAT2	6.633166
BCCIP	7.606644
BCDIN3D	5.416836
BCHE	2.02253
BCKDHA	8.493358
BCKDHB	7.945958
BCKDK	7.38099
BCL10	6.314282
BCL11A	3.000006
BCL11B	4.4493
BCL2	4.5673
BCL2A1	2.943056
BCL2L1	8.706046
BCL2L10	3.476888

BCL2L11	6.043788
BCL2L12	6.587342
BCL2L13	8.199658
BCL2L14	4.720272
BCL2L15	2.596466
BCL2L2	6.306348
BCL3	6.054002
BCL6	7.496656
BCL6B	3.45287
BCL7A	5.834484
BCL7B	8.598516
BCL7C	7.20983
BCL9	5.586578
BCL9L	6.393376
BCLAF1	7.823744
BCMO1	5.593558
BCO2	7.125982
BCOR	5.996672
BCORL1	5.230392
BCORP1	2.84258
BCR	6.09255
BCR	7.312154
BCR	5.965822
BCS1L	6.165486
BDH1	6.134416
BDH2	3.539926
BDKRB1	5.814296
BDKRB2	4.256412
BDNF	3.61364
BDP1	5.608708
BDP1	5.625746
BEAN1	4.637978
BECN1	7.645256
BEGAIN	3.52115
BEND2	2.576798
BEND3	5.009294
BEND4	3.628814
BEND5	3.177656
BEND6	3.111754
BEND7	4.436958
BEST1	3.707406
BEST2	3.513734
BEST3	2.481754

BEST4	4.070026
BET1	6.273306
BET1L	6.164088
BET3L	2.869154
BEX1	3.74737
BEX2	3.706688
BEX4	4.02426
BEX5	2.671146
BFAR	8.500856
BFSP1	4.61849
BFSP2	3.385752
BGLAP	5.716494
BGN	4.543276
BHLHA15	5.521746
BHLHA9	5.991318
BHLHB9	2.780216
BHLHE22	4.461774
BHLHE23	5.442976
BHLHE40	8.365624
BHLHE41	8.211508
BHMT	3.224036
BHMT2	4.008696
BICC1	2.34488
BICD1	5.647616
BICD2	6.65961
BID	8.109294
BIK	5.506124
BIN1	6.623664
BIN2	2.556438
BIN3	6.216734
BIRC2	7.128072
BIRC3	7.346712
BIRC5	8.142108
BIRC5	3.204562
BIRC6	7.313562
BIRC7	4.620774
BIRC8	3.24683
BIVM	5.387422
BIVM	5.505382
BLCAP	8.497552
BLID	2.701044
BLK	3.70499
BLM	6.034614

BLMH	5.5631
BLNK	4.331004
BLOC1S1	6.305474
BLOC1S2	6.787814
BLOC1S3	5.87425
BLVRA	8.169284
BLVRB	7.699364
BLZF1	7.644322
BMF	5.27762
BMI1	7.31017
BMP1	6.322188
BMP10	2.72155
BMP15	2.880142
BMP2	6.035834
BMP2K	5.1518
BMP3	4.434548
BMP4	5.445338
BMP5	2.697966
BMP6	5.454138
BMP7	4.481286
BMP8A	4.303784
BMP8B	5.067142
BMPER	3.203186
BMPR1A	7.128982
BMPR1A	7.171164
BMPR1B	4.134958
BMPR2	6.527234
BMS1	8.40122
BMS1	8.958692
BMS1P1	7.455056
BMS1P1	7.14578
BMX	2.49258
BNC1	3.70269
BNC2	3.794032
BNIP1	5.13253
BNIP2	7.31
BNIP3	7.197224
BNIP3	1.973336
BNIP3L	7.313562
BNIPL	3.658014
BOC	3.010146
BOD1	7.78423
BOD1L	5.114006

BOD1P	5.921126
BOK	5.909082
BOLA1	5.569234
BOLA2	10.48214
BOLA3	8.985104
BOLL	2.641332
BOP1	7.05234
BOP1	7.476806
BPESC1	3.516642
BPGM	6.07117
BPHL	5.603376
BPI	2.8471
BPIL1	3.765298
BPIL2	2.532098
BPIL3	4.013274
BPNT1	8.593238
BPTF	6.282158
BPY2	2.588184
BPY2	2.588184
BPY2	2.588184
BRAF	6.708598
BRAP	7.4384
BRCA1	6.902586
BRCA2	6.40313
BRCC3	7.55284
BRD1	6.44473
BRD2	8.709768
BRD2	8.88287
BRD3	6.98497
BRD4	6.755368
BRD7	7.462846
BRD7	4.914104
BRD7P3	3.367622
BRD7P3	2.8369
BRD8	6.905652
BRD9	6.678178
BRDT	3.142866
BRE	8.528688
BREA2	4.006242
BRF1	5.951386
BRF2	5.836982
BRI3	7.388196
BRI3BP	7.60974

BRIP1	6.50108
BRIX1	8.18099
BRMS1	7.256432
BRMS1L	5.567776
BRP44	8.45061
BRP44L	7.290042
BRPF1	6.550722
BRPF3	6.444022
BRS3	2.66696
BRSK1	4.246192
BRSK2	4.550622
BRWD1	5.601428
BRWD3	5.236134
BSCL2	6.428682
BSDC1	7.2873
BSG	10.114756
BSN	3.353002
BSND	3.705834
BSPH1	2.14521
BSPRY	7.575696
BST1	4.421
BST2	9.848494
BSX	4.626392
BTAF1	6.247454
BTBD1	6.634102
BTBD10	6.404598
BTBD11	4.444794
BTBD11	3.001104
BTBD16	4.808134
BTBD2	7.089056
BTBD3	7.768538
BTBD6	5.877202
BTBD7	5.96017
BTBD8	3.023008
BTBD9	5.948188
BTC	4.767198
BTD	5.806244
BTF3	7.880478
BTF3L4	8.655826
BTF3L4	8.231096
BTF3P11	3.431892
BTF3P9	4.174584
BTG1	10.118398

BTG2	5.693992
BTG3	6.969094
BTG4	2.330258
BTK	2.945372
BTLA	2.891818
BTN1A1	2.740154
BTN2A1	6.561686
BTN2A2	6.041944
BTN2A3	3.729662
BTN3A1	6.464716
BTN3A2	6.727562
BTN3A3	5.777856
BTNL2	3.897418
BTNL2	3.897418
BTNL2	3.897418
BTNL3	2.41009
BTNL8	2.985728
BTNL8	2.97316
BTNL9	4.570734
BTRC	5.626074
BUB1	8.317214
BUB1B	7.633486
BUB3	10.59676
BUD13	6.167464
BUD31	7.204484
BVES	5.993762
BYSL	8.208502
BZRAP1	3.995848
BZW1	10.076684
BZW2	8.199278
C10orf10	6.997706
C10orf107	2.220844
C10orf108	3.63793
C10orf11	3.950242
C10orf110	3.483896
C10orf111	4.016994
C10orf113	2.62205
C10orf114	5.504506
C10orf116	4.621858
C10orf118	4.969804
C10orf119	8.43228
C10orf12	4.804656
C10orf120	2.578368

C10orf122	2.337602
C10orf125	5.541686
C10orf126	3.317798
C10orf129	3.043642
C10orf131	2.059464
C10orf137	7.214778
C10orf140	5.015318
C10orf18	6.55802
C10orf2	5.762526
C10orf25	3.13569
C10orf26	6.54016
C10orf27	3.026934
C10orf28	5.457706
C10orf31	3.498544
C10orf32	3.857892
C10orf35	5.951286
C10orf46	7.65731
C10orf47	6.671096
C10orf53	3.332944
C10orf54	6.00234
C10orf55	3.005736
C10orf57	6.20777
C10orf58	8.09642
C10orf62	3.37585
C10orf67	4.271658
C10orf68	3.271188
C10orf71	3.573032
C10orf72	3.385138
C10orf76	6.591082
C10orf78	4.066316
C10orf79	2.6517
C10orf81	3.377018
C10orf82	3.938674
C10orf84	6.526664
C10orf85	3.99668
C10orf88	6.39322
C10orf90	2.671436
C10orf91	2.983768
C10orf92	3.63029
C10orf93	2.54521
C10orf95	4.819444
C10orf96	2.101228
C10orf99	4.046026

C11orf1	6.837304
C11orf10	9.842744
C11orf16	3.96613
C11orf17	6.816594
C11orf2	7.138282
C11orf21	4.14571
C11orf24	6.180984
C11orf30	5.765156
C11orf31	6.430336
C11orf34	3.083012
C11orf35	3.538512
C11orf35	5.66415
C11orf36	3.13619
C11orf40	4.335322
C11orf41	3.309978
C11orf41	2.818398
C11orf42	3.331154
C11orf44	3.321688
C11orf45	4.876146
C11orf46	5.239236
C11orf48	8.163566
C11orf49	6.683764
C11orf51	6.877006
C11orf52	6.525188
C11orf53	2.656186
C11orf54	7.645722
C11orf57	6.374396
C11orf58	9.101746
C11orf58	10.084486
C11orf59	6.94271
C11orf61	6.11449
C11orf63	3.598218
C11orf64	2.246412
C11orf65	2.766612
C11orf66	4.61076
C11orf67	5.715434
C11orf68	6.432286
C11orf70	2.47729
C11orf71	4.405422
C11orf72	3.615152
C11orf73	6.31122
C11orf74	2.63491
C11orf75	4.952368

C11orf76	5.269198
C11orf80	7.001818
C11orf82	4.797704
C11orf83	5.905456
C11orf84	5.712916
C11orf85	3.216018
C11orf86	4.622142
C11orf87	3.862076
C11orf88	3.111188
C11orf89	5.13318
C11orf9	3.818514
C11orf92	3.892858
C11orf95	6.24356
C11orf96	4.818822
C12orf10	6.850038
C12orf11	7.744908
C12orf12	3.083108
C12orf23	6.039826
C12orf24	3.652706
C12orf26	4.060716
C12orf28	3.27421
C12orf29	5.915426
C12orf32	8.139136
C12orf33	2.468692
C12orf34	5.508798
C12orf35	4.920382
C12orf36	5.533894
C12orf37	2.69203
C12orf39	2.312484
C12orf4	6.275592
C12orf40	2.130532
C12orf41	6.933464
C12orf42	2.664746
C12orf43	6.329298
C12orf44	6.701632
C12orf45	6.73134
C12orf47	4.85321
C12orf48	4.376548
C12orf49	7.343532
C12orf5	6.880058
C12orf50	2.600892
C12orf51	5.706494
C12orf51	6.453096

C12orf52	6.841772
C12orf53	4.653116
C12orf54	2.77852
C12orf55	2.958582
C12orf56	2.34461
C12orf57	8.847466
C12orf59	4.795114
C12orf60	3.956886
C12orf61	4.008654
C12orf62	6.474608
C12orf63	2.622088
C12orf64	2.267376
C12orf65	6.401744
C12orf66	5.572504
C12orf67	3.407806
C12orf68	4.095956
C12orf69	2.252496
C12orf70	3.114282
C12orf72	3.010912
C12orf74	3.004992
C12orf75	6.38524
C12orf76	4.294158
C13orf1	7.339464
C13orf15	6.901488
C13orf16	3.66643
C13orf18	2.862834
C13orf23	7.770384
C13orf26	2.28816
C13orf27	4.40566
C13orf28	2.765468
C13orf29	5.474164
C13orf30	2.56587
C13orf31	4.527734
C13orf33	3.705568
C13orf34	6.464082
C13orf35	2.973832
C13orf36	3.933346
C13orf38	3.7764
C13orf39	2.56153
C13orf40	2.655328
C13orf40	2.715516
C14orf1	8.992346
C14orf101	5.925584

C14orf102	5.31397
C14orf104	5.144664
C14orf105	2.390302
C14orf106	5.498464
C14orf109	7.019628
C14orf118	6.864848
C14orf126	6.54486
C14orf128	4.963032
C14orf129	5.722918
C14orf132	4.079076
C14orf135	5.794246
C14orf138	5.285618
C14orf139	5.17558
C14orf142	6.491184
C14orf143	5.402804
C14orf145	5.80282
C14orf147	6.471804
C14orf148	2.91112
C14orf149	5.445074
C14orf153	6.187658
C14orf156	11.19216
C14orf159	5.89769
C14orf162	4.169098
C14orf165	2.671916
C14orf166	8.505152
C14orf166B	3.061298
C14orf167	2.42384
C14orf169	6.950408
C14orf177	3.573274
C14orf178	3.595484
C14orf179	6.31832
C14orf180	5.81355
C14orf181	5.593338
C14orf182	4.626314
C14orf183	3.181856
C14orf184	4.540118
C14orf19	3.45301
C14orf2	6.821814
C14orf21	7.38276
C14orf23	2.919704
C14orf28	4.53284
C14orf33	4.854064
C14orf37	2.942426

C14orf38	2.08292
C14orf39	2.138718
C14orf4	6.881438
C14orf43	4.27542
C14orf43	6.033568
C14orf45	2.830356
C14orf48	2.828174
C14orf49	4.716888
C14orf50	3.208622
C14orf56	2.702496
C14orf70	3.03056
C14orf79	4.495258
C14orf80	5.45119
C14orf93	6.075958
C15orf17	4.819476
C15orf2	4.344466
C15orf21	4.05825
C15orf23	9.50852
C15orf24	8.823332
C15orf26	3.518428
C15orf27	3.718894
C15orf28	3.98562
C15orf29	5.512032
C15orf29	6.067422
C15orf32	2.869686
C15orf32	6.496118
C15orf33	2.436586
C15orf34	3.273332
C15orf37	4.44306
C15orf38	6.618806
C15orf39	6.430898
C15orf40	3.551682
C15orf41	7.15118
C15orf42	7.177484
C15orf43	2.33894
C15orf44	8.253472
C15orf48	6.226736
C15orf5	2.385918
C15orf51	3.556172
C15orf51	4.390044
C15orf51	4.574846
C15orf51	4.574846
C15orf51	4.868456

C15orf51	5.04939
C15orf51	4.70366
C15orf51	5.04939
C15orf51	4.868456
C15orf51	3.896784
C15orf52	6.706806
C15orf53	3.906978
C15orf54	2.235012
C15orf55	2.937512
C15orf56	5.25721
C15orf57	5.36407
C15orf58	5.220944
C15orf59	5.144912
C15orf60	2.840832
C15orf61	5.18292
C15orf62	5.048376
C15orf63	6.14068
C16orf11	5.720862
C16orf13	5.673274
C16orf3	3.64656
C16orf42	7.247074
C16orf45	3.73289
C16orf46	3.53804
C16orf47	3.657742
C16orf48	5.22537
C16orf5	4.238214
C16orf52	5.408934
C16orf53	8.932138
C16orf54	3.616164
C16orf54	3.616164
C16orf55	4.6199
C16orf57	5.330406
C16orf58	6.537594
C16orf59	5.729062
C16orf61	8.811812
C16orf62	6.314
C16orf63	8.11706
C16orf68	5.365036
C16orf7	5.278442
C16orf70	6.717078
C16orf71	3.59047
C16orf72	7.19807
C16orf73	2.308692

C16orf74	6.805688
C16orf75	5.74278
C16orf78	2.85861
C16orf79	5.19896
C16orf80	8.297642
C16orf81	4.146696
C16orf82	4.249332
C16orf85	3.707446
C16orf86	5.046936
C16orf87	4.88562
C16orf88	7.902844
C16orf89	3.830114
C16orf91	6.12443
C16orf93	5.495404
C17orf101	5.269094
C17orf102	2.626818
C17orf103	4.80111
C17orf104	2.266876
C17orf105	2.328904
C17orf106	5.800162
C17orf106	5.624968
C17orf108	3.279024
C17orf28	7.124978
C17orf37	7.768226
C17orf39	6.051712
C17orf42	5.75808
C17orf44	3.089028
C17orf46	3.640094
C17orf47	2.756414
C17orf48	3.642892
C17orf49	7.812128
C17orf50	5.50567
C17orf51	3.38544
C17orf53	5.44914
C17orf54	3.132288
C17orf55	5.049666
C17orf56	4.861174
C17orf57	2.931556
C17orf58	5.15332
C17orf58	4.395548
C17orf59	3.70819
C17orf59	5.502458
C17orf60	4.026714

C17orf61	5.891396
C17orf62	6.360384
C17orf63	6.77417
C17orf64	3.12474
C17orf65	6.650566
C17orf66	3.318674
C17orf68	6.954286
C17orf69	5.02244
C17orf70	5.886584
C17orf71	5.560582
C17orf73	3.103292
C17orf74	4.07497
C17orf75	6.85763
C17orf76	5.018286
C17orf77	3.001108
C17orf78	2.567384
C17orf79	7.472752
C17orf80	6.12972
C17orf81	7.530256
C17orf82	4.237234
C17orf85	7.613446
C17orf87	2.8825
C17orf88	5.114282
C17orf90	6.227294
C17orf91	4.721592
C17orf95	7.168824
C17orf96	4.634658
C17orf97	4.095764
C17orf99	5.07887
C18orf1	3.751522
C18orf10	6.818046
C18orf12	2.65922
C18orf16	2.661468
C18orf18	3.66524
C18orf19	7.262122
C18orf2	2.076046
C18orf20	2.6353
C18orf21	3.673228
C18orf23	3.497666
C18orf25	5.04888
C18orf26	2.98267
C18orf32	5.690358
C18orf34	2.317646

C18orf45	6.704156
C18orf54	2.605704
C18orf55	5.317536
C18orf56	5.957422
C18orf62	2.697164
C18orf8	7.535826
C19orf10	7.829724
C19orf12	6.077228
C19orf18	2.399394
C19orf2	7.358908
C19orf20	6.112324
C19orf21	6.975978
C19orf22	7.108718
C19orf23	3.608158
C19orf24	7.3233
C19orf25	5.717228
C19orf26	6.09407
C19orf28	6.708008
C19orf29	4.243562
C19orf29	5.819106
C19orf30	3.771252
C19orf33	8.241088
C19orf34	4.43802
C19orf35	4.55869
C19orf39	5.630532
C19orf40	6.180954
C19orf42	8.730916
C19orf43	9.919646
C19orf44	4.863744
C19orf45	3.655686
C19orf46	5.235278
C19orf47	5.733264
C19orf48	7.717478
C19orf50	7.891708
C19orf51	6.425286
C19orf52	6.206866
C19orf53	9.449484
C19orf54	5.893162
C19orf55	5.662494
C19orf56	8.342698
C19orf57	5.250674
C19orf59	4.219182
C19orf6	7.855344

C19orf60	5.825136
C19orf61	6.39949
C19orf62	8.612818
C19orf63	7.595336
C19orf63	4.692262
C19orf66	6.333264
C19orf67	4.474002
C19orf68	5.570542
C19orf70	6.488702
C19orf71	5.90322
C19orf73	4.875336
C19orf75	2.512476
C19orf77	4.94112
C1D	7.495296
C1D	6.373702
C1D	6.953526
C1D	6.995572
C1GALT1	6.434632
C1GALT1C1	4.166966
C1orf100	3.054956
C1orf101	3.047684
C1orf103	6.07319
C1orf105	2.677058
C1orf106	5.976866
C1orf107	5.852114
C1orf109	5.363616
C1orf110	2.836978
C1orf111	4.110188
C1orf113	5.975072
C1orf113	6.04264
C1orf114	2.468184
C1orf115	5.095514
C1orf116	7.14445
C1orf118	3.83199
C1orf122	6.013366
C1orf124	6.918242
C1orf125	2.30118
C1orf126	4.083556
C1orf127	4.238976
C1orf128	7.131016
C1orf129	2.251068
C1orf130	3.439674
C1orf131	4.56352

C1orf135	6.367532
C1orf137	2.308682
C1orf14	2.205052
C1orf141	2.250668
C1orf144	8.652602
C1orf146	1.99776
C1orf150	2.441144
C1orf151	10.16904
C1orf152	9.511824
C1orf156	5.88789
C1orf157	3.195254
C1orf158	3.000614
C1orf159	4.84796
C1orf161	2.567812
C1orf162	3.448432
C1orf163	6.624004
C1orf167	4.096474
C1orf168	2.267992
C1orf170	4.7222
C1orf172	6.515418
C1orf173	2.63375
C1orf174	7.76668
C1orf175	3.94023
C1orf177	3.153092
C1orf180	2.903278
C1orf182	3.467388
C1orf183	3.84108
C1orf186	3.498948
C1orf187	5.13413
C1orf189	3.128652
C1orf190	4.247996
C1orf191	3.365036
C1orf192	2.991576
C1orf194	2.89363
C1orf198	6.885842
C1orf200	3.661434
C1orf201	5.592048
C1orf21	6.866964
C1orf210	6.005244
C1orf211	3.974006
C1orf212	5.39312
C1orf213	4.152126
C1orf216	4.902574

C1orf220	4.429362
C1orf222	3.104018
C1orf223	3.07353
C1orf227	2.473182
C1orf228	3.949052
C1orf229	4.74242
C1orf230	3.024342
C1orf25	6.82937
C1orf26	2.92122
C1orf27	6.778318
C1orf31	5.975944
C1orf35	6.251604
C1orf38	5.083852
C1orf43	10.218746
C1orf46	2.6021
C1orf49	2.450594
C1orf50	5.686034
C1orf50	3.452342
C1orf51	3.142694
C1orf52	5.12177
C1orf53	6.35964
C1orf54	3.117352
C1orf55	6.638032
C1orf56	6.072466
C1orf57	6.987628
C1orf58	8.609204
C1orf59	6.457836
C1orf61	3.219012
C1orf63	6.764696
C1orf64	3.904178
C1orf65	4.999652
C1orf66	6.263126
C1orf68	4.756438
C1orf69	5.032456
C1orf74	5.400904
C1orf77	8.30499
C1orf83	5.783512
C1orf85	6.774784
C1orf86	5.516008
C1orf87	2.604852
C1orf88	3.16352
C1orf89	5.487926
C1orf9	5.835282

C1orf91	4.136286
C1orf92	3.283654
C1orf93	6.692378
C1orf94	3.069444
C1orf95	4.10248
C1orf96	4.941942
C1orf97	4.402524
C1orf99	2.83077
C1QA	3.377222
C1QB	3.975928
C1QBP	8.904642
C1QC	4.296674
C1QL1	6.241742
C1QL2	3.308518
C1QL3	5.343848
C1QL4	4.24284
C1QTNF1	5.209154
C1QTNF2	4.308434
C1QTNF3	3.026908
C1QTNF4	5.555044
C1QTNF5	4.384608
C1QTNF5	5.270358
C1QTNF6	5.884176
C1QTNF7	2.598094
C1QTNF8	4.011432
C1QTNF9	2.246402
C1QTNF9B	2.884798
C1R	6.77267
C1RL	6.567638
C1S	7.962598
C2	3.708014
C2	3.723852
C2	3.723852
C20orf103	2.74163
C20orf106	4.648984
C20orf107	4.236624
C20orf108	6.270244
C20orf11	8.538056
C20orf111	7.04413
C20orf112	5.666114
C20orf114	8.766776
C20orf117	5.17856
C20orf12	4.512982

C20orf123	3.821942
C20orf132	2.876212
C20orf134	5.058328
C20orf135	4.067952
C20orf141	4.022482
C20orf144	6.100392
C20orf151	5.287162
C20orf152	3.312254
C20orf160	4.034016
C20orf165	4.4247
C20orf166	5.198188
C20orf173	3.691864
C20orf177	5.489544
C20orf181	4.982488
C20orf185	4.989484
C20orf186	3.085364
C20orf187	2.195168
C20orf191	6.766032
C20orf191	4.7964
C20orf194	5.820602
C20orf195	4.923444
C20orf196	5.550094
C20orf197	2.821678
C20orf20	7.310952
C20orf200	5.119284
C20orf201	5.559318
C20orf203	2.727138
C20orf24	6.884874
C20orf26	3.059702
C20orf27	5.387882
C20orf29	6.350728
C20orf3	7.589068
C20orf30	8.905774
C20orf4	7.37184
C20orf43	8.369436
C20orf46	2.842322
C20orf54	6.854612
C20orf61	3.195002
C20orf62	3.133802
C20orf7	4.393914
C20orf70	3.56808
C20orf71	3.864842
C20orf72	7.253566

C20orf79	2.459892
C20orf85	3.438468
C20orf90	5.069858
C20orf94	6.75529
C20orf96	3.31687
C21orf104	3.488058
C21orf105	3.25038
C21orf118	3.065058
C21orf119	6.181788
C21orf121	2.645588
C21orf122	3.913966
C21orf125	3.267098
C21orf128	3.516328
C21orf129	3.690904
C21orf15	3.903088
C21orf2	4.976242
C21orf2	4.876618
C21orf29	4.397452
C21orf30	3.78525
C21orf33	7.24212
C21orf34	2.345112
C21orf45	7.5409
C21orf49	2.82109
C21orf56	6.601206
C21orf57	5.795474
C21orf58	4.893532
C21orf59	7.510264
C21orf62	2.576496
C21orf63	6.529284
C21orf67	5.541612
C21orf67	5.564762
C21orf7	4.496368
C21orf70	6.541146
C21orf74	2.354112
C21orf81	4.507812
C21orf82	3.161008
C21orf84	4.025732
C21orf88	4.228882
C21orf89	3.598162
C21orf90	4.54425
C21orf91	6.362776
C21orf93	3.65914
C21orf94	2.109236

C21orf94	2.052374
C21orf94	2.21571
C22orf13	8.000672
C22orf15	3.76656
C22orf23	3.88689
C22orf24	4.011402
C22orf25	6.377134
C22orf26	4.371682
C22orf27	3.038702
C22orf28	8.99988
C22orf30	6.244466
C22orf31	2.143418
C22orf32	7.124766
C22orf33	2.793172
C22orf34	3.636382
C22orf36	4.679436
C22orf37	3.027836
C22orf39	6.547534
C22orf40	5.591376
C22orf42	2.835856
C22orf43	3.392064
C22orf45	3.952934
C22orf46	6.564038
C22orf9	6.40823
C2CD2	6.049692
C2CD2L	5.030234
C2CD3	5.307964
C2CD3	6.368262
C2CD4A	4.595162
C2CD4B	4.92212
C2CD4C	5.297464
C2orf14	2.803812
C2orf14	3.080582
C2orf15	5.28666
C2orf16	2.588102
C2orf18	7.489754
C2orf19	3.114382
C2orf24	5.732976
C2orf27A	5.241084
C2orf27B	6.563064
C2orf28	8.59575
C2orf29	8.533354
C2orf3	4.83706

C2orf34	5.125508
C2orf39	2.717696
C2orf40	3.70573
C2orf42	6.0127
C2orf43	5.535152
C2orf44	5.688764
C2orf46	2.681658
C2orf47	6.069382
C2orf48	4.760266
C2orf49	6.53816
C2orf50	3.709024
C2orf51	3.53711
C2orf52	4.338752
C2orf53	3.652956
C2orf54	4.636474
C2orf55	6.20916
C2orf56	6.290118
C2orf57	3.688268
C2orf58	3.238014
C2orf60	5.07138
C2orf61	2.747694
C2orf62	4.208212
C2orf63	3.323556
C2orf64	6.061556
C2orf65	3.702796
C2orf66	2.932538
C2orf67	3.222578
C2orf68	7.228576
C2orf69	5.354586
C2orf7	5.379024
C2orf70	4.210316
C2orf71	3.4772
C2orf73	2.522586
C2orf76	3.80527
C2orf77	2.265304
C2orf78	3.395584
C2orf80	2.792156
C2orf81	5.035298
C2orf82	5.832604
C2orf83	3.631288
C2orf84	2.767506
C2orf85	4.759374
C2orf86	5.170084

C2orf86	3.927892
C2orf88	4.104988
C2orf89	4.410466
C3	9.23678
C3AR1	2.682294
C3orf1	10.90136
C3orf10	9.21959
C3orf10	3.410628
C3orf14	6.794182
C3orf15	2.35675
C3orf17	6.964594
C3orf18	4.875966
C3orf19	6.135642
C3orf20	3.497932
C3orf21	5.935524
C3orf22	3.52605
C3orf23	5.663464
C3orf24	3.774526
C3orf25	3.37827
C3orf26	6.429652
C3orf27	4.844798
C3orf30	2.503644
C3orf31	6.266348
C3orf32	4.760272
C3orf33	3.902532
C3orf34	5.704486
C3orf35	2.966614
C3orf36	3.249426
C3orf37	8.885704
C3orf38	6.253202
C3orf39	5.11743
C3orf42	5.273904
C3orf43	2.56541
C3orf46	4.86315
C3orf47	4.546062
C3orf48	2.410294
C3orf49	2.638096
C3orf51	2.708836
C3orf52	5.011642
C3orf54	3.561274
C3orf55	3.037344
C3orf56	2.71813
C3orf57	5.380262

C3orf58	4.07404
C3orf59	7.569294
C3orf62	5.858602
C3orf63	6.372518
C3orf64	5.051604
C3orf65	2.354816
C3orf67	3.273638
C3orf70	3.846492
C3orf75	4.56375
C3orf77	5.780922
C3orf78	5.719764
C3orf79	2.62752
C3P1	3.282046
C4A	5.940632
C4A	5.940632
C4A	5.940632
C4BPA	2.569742
C4BPB	2.734082
C4orf11	2.908946
C4orf14	7.029838
C4orf17	2.241558
C4orf19	3.203756
C4orf21	4.49759
C4orf21	4.414172
C4orf22	2.489724
C4orf23	5.561614
C4orf26	2.47361
C4orf27	5.410588
C4orf29	6.07308
C4orf3	6.89812
C4orf31	2.871026
C4orf32	6.74849
C4orf33	4.974722
C4orf34	6.924982
C4orf35	2.402616
C4orf36	4.672506
C4orf37	2.601474
C4orf38	3.005784
C4orf39	3.292242
C4orf40	2.674504
C4orf41	6.769878
C4orf42	4.936706
C4orf43	6.062142

C4orf44	3.778232
C4orf45	2.250878
C4orf46	6.833066
C4orf46	5.8965
C4orf49	4.342984
C4orf50	3.261286
C4orf52	5.37298
C4orf6	2.8984
C4orf7	2.455602
C5	4.209274
C5AR1	4.711118
C5orf13	6.02503
C5orf15	7.079464
C5orf17	3.833222
C5orf20	3.40554
C5orf22	8.00023
C5orf23	2.67634
C5orf24	7.002742
C5orf25	6.157654
C5orf27	2.970222
C5orf28	6.583992
C5orf30	5.160382
C5orf32	8.215494
C5orf33	7.180084
C5orf34	6.170482
C5orf35	4.436202
C5orf36	3.218466
C5orf36	2.41116
C5orf36	2.113614
C5orf38	6.073788
C5orf39	4.494654
C5orf39	3.66916
C5orf4	5.196416
C5orf40	3.438652
C5orf41	5.23734
C5orf42	5.20624
C5orf43	6.697832
C5orf44	5.06538
C5orf44	2.714652
C5orf45	5.216324
C5orf46	2.75572
C5orf47	3.03056
C5orf48	3.24632

C5orf50	3.320838
C5orf51	8.36925
C5orf52	3.359798
C5orf53	4.120192
C5orf54	4.902444
C5orf55	5.352516
C5orf56	5.782914
C5orf60	3.262024
C5orf60	3.163244
C5orf62	5.093578
C6	2.358952
C6orf1	5.01566
C6orf10	2.48663
C6orf100	3.41388
C6orf103	2.15751
C6orf105	6.70986
C6orf106	8.29566
C6orf108	7.425984
C6orf114	4.987772
C6orf115	6.78767
C6orf118	2.903398
C6orf123	3.042092
C6orf123	2.928186
C6orf123	2.928186
C6orf124	4.201614
C6orf125	8.033452
C6orf126	4.110708
C6orf129	4.235354
C6orf130	6.96952
C6orf132	6.504122
C6orf132	6.883564
C6orf136	5.810408
C6orf136	5.585498
C6orf136	5.585498
C6orf138	2.678152
C6orf138	4.606634
C6orf141	5.983268
C6orf142	2.267682
C6orf145	5.807434
C6orf146	2.43447
C6orf15	4.42775
C6orf15	4.427642
C6orf15	4.466304

C6orf150	8.500324
C6orf154	3.509284
C6orf155	6.30434
C6orf162	6.184044
C6orf163	2.247726
C6orf165	2.644526
C6orf168	5.927366
C6orf170	3.754806
C6orf174	2.478128
C6orf186	2.128976
C6orf191	2.291354
C6orf192	6.520502
C6orf195	3.285846
C6orf201	3.198466
C6orf203	5.607
C6orf204	2.619592
C6orf208	3.721062
C6orf211	5.335014
C6orf218	2.774938
C6orf221	3.712136
C6orf222	3.45978
C6orf223	4.80752
C6orf225	4.51901
C6orf226	5.316636
C6orf227	4.39982
C6orf25	4.655712
C6orf25	4.042272
C6orf25	4.649624
C6orf26	5.310484
C6orf26	5.323756
C6orf27	4.952356
C6orf35	6.081424
C6orf35	5.996762
C6orf47	6.440638
C6orf47	6.398316
C6orf47	6.398316
C6orf48	7.945288
C6orf48	7.945288
C6orf52	5.779058
C6orf54	4.129814
C6orf57	5.718594
C6orf58	2.778482
C6orf62	9.176888

C6orf64	6.53373
C6orf70	5.903968
C6orf72	7.408796
C6orf81	3.70466
C6orf89	7.556376
C6orf94	2.467308
C6orf97	2.578756
C6orf99	4.502052
C7	2.42748
C7orf10	3.346612
C7orf11	10.32152
C7orf11	7.398778
C7orf11	11.09192
C7orf13	6.050402
C7orf16	3.048872
C7orf23	7.053856
C7orf25	4.930998
C7orf26	6.124176
C7orf27	6.54829
C7orf29	7.337386
C7orf31	4.55818
C7orf33	3.498444
C7orf34	3.449244
C7orf36	6.65185
C7orf41	4.13239
C7orf42	9.289432
C7orf43	5.504012
C7orf44	9.030062
C7orf45	2.2713
C7orf46	4.020584
C7orf47	6.596748
C7orf49	7.704072
C7orf50	8.102172
C7orf51	5.272598
C7orf52	3.268284
C7orf53	2.450958
C7orf54	3.938558
C7orf55	5.555568
C7orf57	3.394446
C7orf58	2.460126
C7orf59	6.389858
C7orf60	4.565624
C7orf61	5.355506

C7orf62	2.788896
C7orf63	2.67122
C7orf64	6.513302
C7orf66	2.689506
C7orf68	5.167488
C7orf69	2.59892
C7orf70	7.2747
C7orf70	7.339868
C7orf72	2.005236
C7orf72	2.60056
C8A	2.732402
C8B	3.041778
C8G	5.57298
C8orf12	2.497836
C8orf17	3.618962
C8orf22	2.480566
C8orf30A	7.000566
C8orf30A	6.907866
C8orf31	3.491668
C8orf33	8.437798
C8orf34	2.749988
C8orf37	6.020656
C8orf38	6.097772
C8orf4	3.383
C8orf40	6.841718
C8orf41	6.644236
C8orf42	5.613234
C8orf44	3.97082
C8orf45	3.70635
C8orf46	4.42719
C8orf47	4.152148
C8orf48	2.38177
C8orf49	2.952326
C8orf51	5.37875
C8orf54	2.19632
C8orf55	6.58044
C8orf56	3.274048
C8orf58	5.574378
C8orf59	6.38069
C8orf73	4.115892
C8orf73	5.461572
C8orf74	4.073682
C8orf76	6.528702

C8orf77	5.053958
C8orf79	3.57247
C8orf79	2.604188
C8orf8	2.15647
C8orf80	3.528168
C8orf84	5.40892
C8orf85	4.87552
C8orf86	2.819826
C9	2.636708
C9orf100	6.1195
C9orf102	5.915896
C9orf102	6.008248
C9orf102	5.507654
C9orf103	7.012856
C9orf106	3.775432
C9orf11	2.757308
C9orf114	6.794898
C9orf116	5.284164
C9orf119	6.358624
C9orf123	7.272934
C9orf125	3.01128
C9orf128	3.108356
C9orf130	4.16297
C9orf131	2.8674
C9orf131	5.044288
C9orf135	2.740544
C9orf140	6.261406
C9orf141	5.865492
C9orf142	6.95175
C9orf144	2.920972
C9orf144B	3.33202
C9orf150	7.573418
C9orf152	3.757106
C9orf153	2.47643
C9orf156	4.87912
C9orf16	6.759438
C9orf163	3.510504
C9orf167	5.041322
C9orf169	5.393764
C9orf169	5.45242
C9orf170	4.705634
C9orf171	3.790718
C9orf172	4.90428

C9orf173	4.684466
C9orf174	3.835908
C9orf21	5.76278
C9orf23	6.653464
C9orf24	3.747434
C9orf25	5.299746
C9orf27	2.476904
C9orf29	2.604232
C9orf3	6.183172
C9orf30	8.456894
C9orf31	2.815798
C9orf37	5.898268
C9orf38	2.314428
C9orf4	3.828946
C9orf40	6.911802
C9orf41	6.453074
C9orf43	4.036282
C9orf46	4.48804
C9orf5	9.031696
C9orf50	4.106102
C9orf53	3.373108
C9orf57	3.362394
C9orf6	5.615916
C9orf62	4.77784
C9orf64	7.546732
C9orf66	3.457582
C9orf68	4.541426
C9orf7	5.730302
C9orf70	3.616818
C9orf71	3.216454
C9orf72	3.660134
C9orf78	7.958598
C9orf79	3.248844
C9orf80	7.930384
C9orf82	6.16227
C9orf84	2.46292
C9orf85	5.190868
C9orf86	7.363448
C9orf89	7.094484
C9orf9	5.08279
C9orf91	6.016538
C9orf93	3.739486
C9orf95	5.328756

C9orf96	4.896972
C9orf98	3.838776
CA1	2.364546
CA10	3.212412
CA11	6.364812
CA12	6.332902
CA13	3.183434
CA14	3.058558
CA2	5.071784
CA3	3.542002
CA4	4.36428
CA5A	3.833154
CA5B	5.450642
CA5BP	5.790904
CA6	2.675064
CA7	3.655266
CA8	2.704164
CA9	4.653076
CAB39	9.02005
CAB39L	3.130604
CABIN1	6.581652
CABLES1	5.900406
CABLES2	5.294746
CABP1	3.253672
CABP2	6.04014
CABP4	4.592828
CABP5	4.102252
CABP7	4.261946
CABYR	4.987372
CACHD1	5.901118
CACNA1A	3.343782
CACNA1B	4.10856
CACNA1C	3.189834
CACNA1C	2.833946
CACNA1D	3.31484
CACNA1E	3.233348
CACNA1F	3.570988
CACNA1G	4.654508
CACNA1H	4.869684
CACNA1I	4.182412
CACNA1S	3.230088
CACNA2D1	2.474364
CACNA2D2	3.385372

CACNA2D3	2.933892
CACNA2D4	3.686676
CACNB1	4.893242
CACNB2	3.179712
CACNB3	6.352294
CACNB4	2.879994
CACNG1	3.527396
CACNG2	2.984018
CACNG3	2.746188
CACNG4	6.45237
CACNG5	3.576882
CACNG6	4.266056
CACNG7	4.625972
CACNG8	5.712016
CACYBP	7.053714
CACYBP	9.704984
CAD	6.639346
CADM1	3.164062
CADM2	2.648518
CADM3	3.919168
CADM4	6.327764
CADPS	2.757234
CADPS2	4.294978
CAGE1	2.804256
CALB1	9.668248
CALB2	7.791578
CALCA	3.698322
CALCB	3.269128
CALCOCO1	7.045784
CALCOCO2	7.803408
CALCR	2.531252
CALCRL	2.399654
CALD1	5.526772
CALHM1	3.594296
CALHM2	7.195978
CALHM3	5.123584
CALM1	8.21497
CALM2	7.4737
CALM3	9.808494
CALML3	4.104914
CALML4	5.877158
CALML5	4.705416
CALML6	3.865064

CALN1	3.01396
CALR	9.987934
CALR3	2.87321
CALU	8.285016
CALY	5.705932
CAMK1	4.510316
CAMK1D	6.979192
CAMK1G	3.218294
CAMK2A	4.5127
CAMK2B	2.70294
CAMK2D	7.062458
CAMK2G	6.83084
CAMK2N1	8.794236
CAMK2N2	6.658652
CAMK4	3.33474
CAMKK1	3.885048
CAMKK2	6.38894
CAMKV	2.952058
CAMLG	6.479102
CAMP	4.517452
CAMSAP1	6.944366
CAMSAP1L1	6.94662
CAMTA1	3.984484
CAMTA2	5.741466
CAND1	8.037578
CAND2	4.409982
CANT1	7.463666
CANX	11.08328
CAP1	10.49642
CAP2	6.940452
CAPG	8.42712
CAPN1	9.39495
CAPN10	4.24097
CAPN11	2.886994
CAPN12	3.92879
CAPN13	2.86472
CAPN2	9.271054
CAPN3	4.324528
CAPN5	5.350352
CAPN6	3.169214
CAPN7	7.340014
CAPN9	2.666376
CAPNS1	10.46634

CAPNS2	3.103876
CAPRIN1	9.918994
CAPRIN2	5.409476
CAPS	7.834544
CAPS2	3.08823
CAPSL	3.516034
CAPZA1	6.581326
CAPZA2	7.745438
CAPZA3	2.04415
CAPZB	9.650446
CARD10	6.503786
CARD11	4.117154
CARD14	3.94376
CARD16	2.719468
CARD17	2.701002
CARD18	2.762674
CARD6	6.05615
CARD8	5.28878
CARD9	5.157648
CARHSP1	7.344102
CARKD	7.807584
CARM1	6.82961
CARNS1	4.507106
CARS	7.09605
CARS2	7.06192
CARTPT	2.647464
CARTPT	2.647464
CASC1	2.421422
CASC2	3.385294
CASC3	8.384722
CASC4	7.146048
CASC5	6.335676
CASD1	6.41501
CASK	7.253838
CASKIN1	3.83703
CASKIN2	5.90648
CASP1	4.516182
CASP10	5.337932
CASP12	2.567938
CASP14	5.377726
CASP2	9.036668
CASP3	6.92431
CASP4	8.111014

CASP5	2.561942
CASP6	6.378274
CASP7	7.48587
CASP8	6.273852
CASP8AP2	6.146528
CASP9	7.026968
CASQ1	3.136646
CASQ2	2.529644
CASR	3.80073
CASS4	3.189042
CAST	8.223958
CASZ1	5.149732
CASZ1	6.087542
CASZ1	5.3983
CAT	6.929268
CATSPER1	3.967046
CATSPER2	4.144738
CATSPER2P1	3.617264
CATSPER3	3.586638
CATSPER4	2.976318
CATSPERB	2.481216
CATSPERG	4.15684
CAV1	8.95305
CAV2	8.565754
CAV3	3.70644
CBARA1	6.753664
CBFA2T2	5.402556
CBFA2T3	5.076412
CBFB	5.851158
CBL	7.409994
CBLB	4.71726
CBLC	4.670376
CBLL1	6.4571
CBLN1	3.524568
CBLN2	3.310504
CBLN3	3.679454
CBLN4	3.119646
CBR1	7.406278
CBR3	6.217012
CBR4	6.400272
CBS	7.729894
CBWD1	7.778172
CBWD1	7.585978

CBWD3	7.600664
CBWD3	7.747994
CBWD3	7.751074
CBWD5	6.89093
CBWD5	6.138856
CBX1	6.878862
CBX2	6.090196
CBX3	8.16557
CBX4	6.971436
CBX5	9.52108
CBX6	7.26075
CBX7	5.470038
CBX8	6.072584
CBY1	4.975708
CC2D1A	7.011178
CC2D1B	5.612776
CC2D2A	4.977948
CC2D2B	2.574304
CCAR1	7.32577
CCBE1	2.922446
CCBL1	5.273894
CCBL2	5.297732
CCBP2	3.316514
CCDC101	8.63578
CCDC102A	5.41713
CCDC102B	2.577802
CCDC103	4.75626
CCDC104	5.582616
CCDC105	4.311972
CCDC106	5.7255
CCDC107	5.786466
CCDC108	3.687694
CCDC109A	6.72898
CCDC109B	7.283882
CCDC11	3.479458
CCDC110	2.59182
CCDC111	5.505924
CCDC112	3.075512
CCDC113	5.274018
CCDC114	4.02651
CCDC115	6.658536
CCDC116	4.660638
CCDC117	7.342996

CCDC12	7.643228
CCDC120	5.256912
CCDC121	4.107856
CCDC122	3.285514
CCDC123	6.211736
CCDC124	7.66012
CCDC125	4.833022
CCDC125	4.786866
CCDC126	3.838158
CCDC127	8.064992
CCDC129	3.036546
CCDC13	4.449004
CCDC130	6.634582
CCDC132	4.396376
CCDC134	6.365736
CCDC135	4.21004
CCDC136	3.184178
CCDC137	5.80466
CCDC138	4.64723
CCDC14	5.486088
CCDC140	3.617828
CCDC141	2.559954
CCDC142	5.00633
CCDC144A	4.921668
CCDC144B	5.269516
CCDC144B	5.354042
CCDC144NL	4.741326
CCDC146	2.965578
CCDC147	2.48435
CCDC148	2.24153
CCDC149	4.96108
CCDC15	3.388622
CCDC150	3.702894
CCDC151	4.191674
CCDC153	3.424812
CCDC154	4.553998
CCDC155	5.247718
CCDC157	4.37463
CCDC158	2.360392
CCDC159	5.000086
CCDC17	4.3379
CCDC18	3.433696
CCDC19	3.775642

CCDC21	6.914878
CCDC22	5.12631
CCDC23	4.395706
CCDC24	5.347396
CCDC25	6.673816
CCDC26	2.397142
CCDC27	4.493426
CCDC28A	6.247034
CCDC28B	3.960854
CCDC3	2.686778
CCDC30	2.726902
CCDC33	4.374062
CCDC34	4.747632
CCDC36	2.37295
CCDC37	3.76231
CCDC38	2.315822
CCDC39	2.29862
CCDC40	3.878942
CCDC41	2.887212
CCDC42	3.044764
CCDC42B	5.212022
CCDC43	6.924258
CCDC45	7.051166
CCDC46	2.746404
CCDC47	8.634154
CCDC48	4.984268
CCDC50	8.209762
CCDC51	5.618862
CCDC53	6.030676
CCDC54	2.417468
CCDC55	6.865394
CCDC56	8.41811
CCDC57	4.924868
CCDC58	7.231572
CCDC59	6.993194
CCDC6	8.022632
CCDC60	3.427206
CCDC61	5.804722
CCDC62	3.12791
CCDC63	2.801868
CCDC64	7.098986
CCDC64B	6.260158
CCDC65	2.94037

CCDC66	3.025184
CCDC67	2.321266
CCDC68	2.369766
CCDC69	3.576028
CCDC70	3.26342
CCDC71	6.58342
CCDC72	7.27868
CCDC72	7.467992
CCDC73	2.215894
CCDC74A	4.238822
CCDC74A	4.14019
CCDC75	5.406534
CCDC76	4.21706
CCDC77	7.155968
CCDC78	4.294964
CCDC79	2.193634
CCDC8	4.718434
CCDC80	5.738372
CCDC81	3.061786
CCDC82	4.924334
CCDC82	5.203672
CCDC83	2.226528
CCDC84	6.702636
CCDC85A	2.835036
CCDC85A	4.29653
CCDC85B	6.48905
CCDC85C	5.997642
CCDC85C	5.307174
CCDC86	6.775574
CCDC87	3.415554
CCDC88A	4.94434
CCDC88B	4.573336
CCDC88C	5.41315
CCDC88C	4.74338
CCDC89	3.368944
CCDC9	6.76405
CCDC90A	7.113844
CCDC90B	5.825066
CCDC91	6.349894
CCDC92	4.717246
CCDC93	6.852242
CCDC94	6.137534
CCDC96	3.763256

CCDC97	6.032022
CCDC99	7.381272
CCHCR1	6.029152
CCHCR1	5.908966
CCIN	3.010366
CCK	4.129946
CCKAR	3.750044
CCKBR	4.87194
CCL1	3.31478
CCL11	3.647664
CCL13	2.901556
CCL14-CCL15	3.172308
CCL16	3.039138
CCL17	4.81478
CCL18	2.780762
CCL19	3.674406
CCL2	2.855368
CCL20	4.59712
CCL21	3.80699
CCL22	5.220082
CCL23	2.69748
CCL24	3.629488
CCL25	4.269412
CCL26	3.099022
CCL27	4.409482
CCL28	5.291648
CCL3	4.745078
CCL3L1	5.896892
CCL3L1	5.896892
CCL3L1	5.896892
CCL4	2.320458
CCL4L1	3.040692
CCL4L1	3.124168
CCL4L1	3.124168
CCL5	10.001084
CCL7	2.433932
CCL8	2.460144
CCM2	6.822824
CCNA1	4.276226
CCNA2	8.451188
CCNB1	10.07349
CCNB1IP1	5.956024
CCNB2	9.115334

CCNB3	2.797952
CCNC	8.611936
CCND1	8.410416
CCND1	4.255748
CCND2	3.598512
CCND3	6.963454
CCNDBP1	6.339862
CCNE1	6.509578
CCNE2	7.798852
CCNF	6.975916
CCNG1	7.026474
CCNG2	6.395994
CCNH	4.605366
CCNI	9.498628
CCNI2	4.073608
CCNJ	4.688376
CCNJL	6.412044
CCNK	6.703454
CCNL1	7.851282
CCNL2	6.905752
CCNL2	6.827518
CCNO	5.112102
CCNT1	7.394322
CCNT2	6.028272
CCNY	8.605608
CCNYL1	7.498742
CCNYL2	1.99545
CCNYL3	2.10406
CCPG1	3.578618
CCR1	2.86976
CCR10	3.970786
CCR2	2.764778
CCR2	3.04109
CCR3	2.656882
CCR4	2.329412
CCR5	2.911024
CCR5	2.911024
CCR6	3.791806
CCR6	3.151288
CCR6	5.014356
CCR7	3.968838
CCR8	2.928588
CCR9	2.593632

CCRL1	2.45211
CCRL1	3.128142
CCRL2	2.702572
CCRL2	2.954016
CCRN4L	7.26733
CCS	6.242046
CCT2	9.73037
CCT3	10.219352
CCT4	9.328864
CCT5	10.83464
CCT6A	10.132446
CCT6B	4.904342
CCT7	10.58004
CCT8	7.535738
CCT8L2	4.387636
CCZ1	8.19691
CCZ1	8.762652
CD101	3.038052
CD109	7.973176
CD14	5.888864
CD151	8.529204
CD160	2.400278
CD163	2.498548
CD163L1	4.275996
CD164	10.075462
CD164L2	4.881396
CD177	4.4295
CD177	2.721042
CD180	2.61792
CD19	4.411258
CD1A	3.007712
CD1B	2.485844
CD1C	2.98112
CD1D	4.64428
CD1E	4.246578
CD2	3.158262
CD200	2.629614
CD200R1	2.546488
CD200R1L	2.731354
CD207	3.821458
CD209	3.640786
CD209	3.231534
CD22	5.846946

CD226	2.46342
CD24	9.377648
CD244	2.435498
CD247	3.51315
CD248	3.438294
CD27	3.699872
CD274	5.160146
CD276	7.53802
CD28	2.390032
CD2AP	7.787728
CD2BP2	7.770418
CD300A	3.770458
CD300C	4.552722
CD300E	4.54061
CD300LB	2.911934
CD300LD	2.856632
CD300LF	3.715374
CD300LG	4.555532
CD302	2.414394
CD320	6.448612
CD33	3.77348
CD34	3.152284
CD36	5.888936
CD37	3.668762
CD38	4.306328
CD3D	3.65679
CD3E	2.81826
CD3EAP	5.871204
CD3G	3.71127
CD4	3.73391
CD40	2.896878
CD40LG	2.18095
CD44	10.016504
CD46	7.608854
CD47	9.632134
CD48	3.80974
CD5	3.975446
CD52	4.323756
CD53	2.900306
CD55	7.870854
CD58	5.232268
CD59	9.136024
CD5L	3.52248

CD6	4.426032
CD63	9.939086
CD68	7.38834
CD69	2.485978
CD7	6.407846
CD70	4.867692
CD72	4.184204
CD74	5.499918
CD79A	4.137088
CD79B	4.11162
CD80	2.738592
CD81	9.263762
CD82	8.750494
CD83	5.892042
CD84	2.673808
CD86	2.508764
CD8A	4.540604
CD8B	3.66136
CD8B	2.352498
CD9	10.65118
CD93	3.153208
CD96	2.640666
CD97	8.457236
CD99	9.048284
CD99	9.048284
CD99L2	5.56924
CDA	8.04396
CDADC1	4.958786
CDAN1	5.42253
CDC123	8.881868
CDC14A	4.28426
CDC14B	5.64138
CDC14C	5.021956
CDC16	7.659192
CDC20	9.451024
CDC20B	2.721804
CDC23	7.9861
CDC25A	6.625292
CDC25B	8.027372
CDC25C	7.029238
CDC26	8.076584
CDC26	6.526854
CDC27	7.115418

CDC34	6.328936
CDC37	8.507142
CDC37L1	5.787262
CDC40	7.7265
CDC42	5.556
CDC42BPA	6.654942
CDC42BPB	7.150044
CDC42BPG	5.594162
CDC42EP1	7.17674
CDC42EP2	6.497256
CDC42EP3	6.756214
CDC42EP4	6.813944
CDC42EP5	5.847846
CDC42SE1	6.59422
CDC42SE2	9.458994
CDC45	8.55535
CDC5L	7.77385
CDC6	9.431754
CDC7	4.692016
CDC73	7.82334
CDCA2	7.80761
CDCA3	7.835418
CDCA4	7.975508
CDCA5	7.371892
CDCA7	6.886588
CDCA7L	8.429474
CDCA8	7.45068
CDCP1	8.865942
CDCP2	3.46459
CDH1	10.010388
CDH10	2.950646
CDH11	3.461636
CDH12	2.334704
CDH13	3.79668
CDH15	3.909988
CDH16	3.495926
CDH17	2.45329
CDH18	2.78965
CDH19	2.661444
CDH2	3.028732
CDH20	3.182134
CDH22	3.859608
CDH23	4.145688

CDH24	4.621876
CDH26	3.794888
CDH3	9.596276
CDH4	4.270146
CDH5	5.44235
CDH6	2.638418
CDH7	2.595986
CDH8	3.063918
CDH9	2.419768
CDHR1	3.070748
CDHR2	3.490734
CDHR3	2.858646
CDHR4	4.812686
CDHR5	3.987172
CDIPT	9.565294
CDK1	6.813356
CDK10	6.514094
CDK11A	6.505678
CDK12	8.425824
CDK13	7.819156
CDK14	7.070428
CDK15	2.800448
CDK16	7.41482
CDK17	5.22331
CDK18	5.42972
CDK19	6.339442
CDK2	9.528032
CDK20	4.888172
CDK2AP1	9.499918
CDK2AP2	6.426876
CDK3	4.299096
CDK4	9.157068
CDK5	8.35973
CDK5R1	5.320184
CDK5R2	5.807378
CDK5RAP1	6.775518
CDK5RAP2	7.413336
CDK5RAP3	8.20252
CDK6	6.382432
CDK7	6.007606
CDK7	6.007606
CDK8	8.238778
CDK9	9.272998

CDKAL1	7.900692
CDKL1	3.071168
CDKL2	2.59809
CDKL3	2.93742
CDKL4	2.268192
CDKL5	2.920874
CDKN1A	6.156576
CDKN1B	8.174808
CDKN1C	6.141542
CDKN2A	7.995882
CDKN2AIP	6.255532
CDKN2AIPNL	5.87369
CDKN2B	7.13925
CDKN2C	5.201346
CDKN2D	6.676462
CDKN3	8.178842
CDNF	3.779482
CDO1	3.279708
CDON	5.02031
CDR1	2.344096
CDR2	7.548294
CDR2L	6.032886
CDRT1	2.204524
CDRT1	5.76666
CDRT1	5.672206
CDRT1	6.664462
CDRT15	4.204004
CDRT4	4.329782
CDS1	7.882022
CDS2	8.300824
CDSN	4.808834
CDSN	4.794076
CDSN	4.751466
CDT1	7.161506
CDV3	9.515654
CDV3	9.7766
CDX1	5.169114
CDX2	4.559642
CDX4	2.850614
CDY1	2.830562
CDY1	2.568718
CDY1	2.730804
CDY1	2.568718

CDYL	7.226848
CDYL2	5.062472
CEACAM1	6.872592
CEACAM16	4.251222
CEACAM19	4.712028
CEACAM20	3.373904
CEACAM21	3.068544
CEACAM3	4.786236
CEACAM4	3.681468
CEACAM5	4.952088
CEACAM6	7.72152
CEACAM7	2.880506
CEACAM8	3.783622
CEBPA	5.426806
CEBPB	8.156024
CEBPD	6.802266
CEBPE	4.265704
CEBPG	6.792874
CEBPZ	5.016164
CECR1	3.072536
CECR2	3.579864
CECR4	4.308216
CECR5	6.111928
CECR6	5.252804
CEL	4.344454
CELA1	4.138018
CELA2A	3.265984
CELA2B	4.156866
CELA3A	4.402604
CELA3B	5.04984
CELF1	8.936904
CELF2	2.646774
CELF3	4.766118
CELF4	4.55006
CELF5	4.029426
CELF6	4.351764
CELP	4.671756
CELSR1	6.74668
CELSR2	5.712756
CELSR3	4.208424
CEMP1	4.23644
CEND1	5.468606
CENPA	7.69997

CENPB	7.00635
CENPBD1	5.291718
CENPC1	3.48468
CENPE	4.255296
CENPF	6.591048
CENPH	4.467042
CENPI	6.73352
CENPJ	6.255624
CENPK	6.610938
CENPL	7.3721
CENPM	7.407654
CENPN	9.023284
CENPO	7.75774
CENPO	7.377116
CENPP	5.384122
CENPQ	3.322998
CENPT	5.632438
CENPV	5.915016
CENPVL1	5.221126
CENPVL1	5.221126
CENPW	6.818216
CEP110	4.871286
CEP120	5.450072
CEP135	3.917192
CEP152	4.450954
CEP164	5.694092
CEP170	4.586112
CEP170P1	3.519604
CEP192	7.28052
CEP250	4.756436
CEP290	2.86
CEP350	6.380146
CEP55	7.358252
CEP57	6.645712
CEP57L1	4.835674
CEP63	4.74471
CEP68	4.946966
CEP70	6.364516
CEP72	6.830298
CEP76	5.640584
CEP78	6.508596
CEP97	6.027584
CEPT1	8.242042

CER1	2.886826
CERCAM	5.622488
CERK	6.804938
CERKL	3.147058
CES1	7.597412
CES1P1	4.277964
CES2	6.853612
CES3	3.374128
CES4A	3.433694
CES5A	2.932796
CETN1	3.732992
CETN2	7.174314
CETN3	3.413386
CETP	3.1759
CFB	8.841108
CFB	9.022752
CFB	9.456742
CFC1	4.625368
CFC1B	4.525566
CFD	5.613792
CFDP1	5.76809
CFH	2.951934
CFHR1	2.462276
CFHR2	2.522022
CFHR3	1.92957
CFHR4	2.316286
CFHR5	2.406476
CFI	2.697898
CFL1	10.73504
CFL2	6.551544
CFLAR	6.891902
CFP	4.534482
CFTR	2.345436
CGA	2.83923
CGB	4.830856
CGB1	4.431002
CGB2	4.588162
CGB5	4.69606
CGB5	4.780294
CGB7	4.549662
CGGBP1	7.309948
CGN	5.396026
CGNL1	3.64356

CGREF1	3.429018
CGRRF1	3.526676
CH25H	2.894958
CHAC1	6.264978
CHAC2	4.045126
CHAD	3.754024
CHADL	5.174938
CHAF1A	7.618572
CHAF1B	8.594442
CHAT	3.883582
CHCHD1	5.716922
CHCHD10	7.700736
CHCHD2	10.60228
CHCHD3	8.02694
CHCHD4	5.981894
CHCHD5	5.14662
CHCHD6	5.967388
CHCHD7	4.809488
CHCHD8	6.156074
CHD1	5.791762
CHD1L	5.136068
CHD2	8.250274
CHD3	7.536864
CHD4	8.921582
CHD5	4.304116
CHD6	6.270398
CHD7	7.425466
CHD8	7.701242
CHD9	6.38442
CHD9	2.272188
CHDH	4.32717
CHEK1	8.550382
CHEK2	6.997398
CHERP	7.086298
CHFR	6.718566
CHGA	3.336916
CHGB	3.245294
CHI3L1	5.335756
CHI3L2	4.585926
CHIA	2.975838
CHIC1	4.477668
CHIC2	5.478744
CHID1	6.676768

CHIT1	3.671416
CHKA	8.45238
CHKB-CPT1B	5.987302
CHL1	2.584716
CHM	4.214138
CHML	6.93449
CHMP1A	8.86735
CHMP1B	9.360576
CHMP2A	10.003626
CHMP2B	7.904164
CHMP4A	7.067798
CHMP4A	5.201624
CHMP4B	8.350114
CHMP4C	6.112744
CHMP5	7.87555
CHMP6	5.705596
CHMP7	6.388418
CHN1	3.65964
CHN2	4.553426
CHODL	3.22214
CHORDC1	6.662088
CHP	10.78744
CHP2	4.165696
CHPF	6.049204
CHPF2	5.965504
CHPT1	5.85825
CHRAC1	8.589728
CHRD	4.16439
CHRDL1	5.200268
CHRDL2	3.630224
CHRFAM7A	5.336642
CHRM1	4.090178
CHRM2	2.963382
CHRM3	2.295176
CHRM4	4.26161
CHRM5	3.329778
CHRNA1	3.700648
CHRNA10	4.414522
CHRNA2	3.969766
CHRNA3	3.29238
CHRNA4	4.023962
CHRNA5	7.665426
CHRNA6	2.341152

CHRNA7	4.11024
CHRNA9	2.7068
CHRNB1	5.829046
CHRNB2	4.925062
CHRNB3	2.64922
CHRNB4	4.116728
CHRND	3.753928
CHRNE	3.784904
CHRNG	4.999922
CHST1	4.006354
CHST10	4.452234
CHST11	7.358714
CHST12	6.929414
CHST13	5.002788
CHST14	6.201162
CHST15	7.768264
CHST2	4.974326
CHST3	6.054378
CHST4	3.253608
CHST5	4.348244
CHST6	3.778458
CHST7	4.825188
CHST8	5.383844
CHST9	2.22645
CHSY1	6.249648
CHSY3	2.43561
CHTF18	5.741298
CHTF8	8.573474
CHUK	6.176228
CHURC1	6.04063
CIAO1	7.495772
CIAPIN1	6.366372
CIB1	7.653212
CIB2	2.90871
CIB3	3.87059
CIB4	3.112582
CIC	5.80469
CIDEA	3.466134
CIDEB	3.664234
CIDEC	4.123928
CIDECP	6.166998
CIITA	4.217462
CILP	3.030528

CILP2	5.32633
CINP	6.339868
CIR1	3.25093
CIRBP	8.580272
CIRH1A	10.278686
CISD1	6.251838
CISD2	8.378096
CISH	4.70376
CIT	8.004378
CITED1	3.895306
CITED2	8.116308
CITED4	5.94374
CIZ1	8.13209
CKAP2	7.857926
CKAP2L	6.479886
CKAP4	6.235932
CKAP5	8.75515
CKB	5.269686
CKM	3.372946
CKMT1A	5.681216
CKMT1A	5.681216
CKMT2	3.092186
CKS1B	8.707048
CKS1B	10.249652
CKS1B	10.286832
CKS2	9.293002
CLASP1	7.076142
CLASP2	6.651926
CLASRP	5.872428
CLC	2.693428
CLCA1	2.611274
CLCA2	3.028382
CLCA3P	2.41913
CLCA4	2.818266
CLCC1	7.230536
CLCF1	7.157626
CLCN1	4.29895
CLCN2	5.730742
CLCN3	7.561508
CLCN4	5.157446
CLCN5	4.459924
CLCN6	6.389308
CLCN7	7.33253

CLCNKA	3.428362
CLCNKB	3.62727
CLDN1	9.533028
CLDN10	2.991852
CLDN11	5.654962
CLDN12	7.913156
CLDN14	2.939488
CLDN15	5.393078
CLDN16	3.813838
CLDN17	2.88191
CLDN18	3.485744
CLDN19	5.074562
CLDN2	3.301912
CLDN20	2.87894
CLDN22	2.72892
CLDN23	5.62074
CLDN24	2.271884
CLDN3	7.242566
CLDN4	7.862654
CLDN5	5.248184
CLDN6	2.812344
CLDN7	9.63202
CLDN8	3.538056
CLDN9	5.091528
CLDND1	8.310088
CLDND2	4.479756
CLEC10A	3.400904
CLEC11A	4.367172
CLEC12A	2.441516
CLEC12B	2.26708
CLEC14A	4.22418
CLEC16A	6.547358
CLEC17A	4.319004
CLEC18A	4.253602
CLEC18B	4.33707
CLEC18B	4.281846
CLEC1A	2.691194
CLEC1B	2.73382
CLEC2A	2.368852
CLEC2B	2.142988
CLEC2D	4.709144
CLEC3A	2.576352
CLEC3B	5.047684

CLEC4A	2.567172
CLEC4C	2.99032
CLEC4D	2.706334
CLEC4E	2.230248
CLEC4F	3.977758
CLEC4G	4.956422
CLEC4GP1	6.049364
CLEC4M	3.068704
CLEC5A	2.87114
CLEC6A	2.793406
CLEC7A	3.565588
CLEC9A	2.37761
CLECL1	3.057138
CLGN	2.53368
CLIC1	9.823394
CLIC1	9.883192
CLIC1	9.903818
CLIC2	2.22252
CLIC3	5.697
CLIC4	8.915902
CLIC5	2.858488
CLIC6	2.72806
CLINT1	9.397138
CLIP1	6.531456
CLIP2	5.371382
CLIP3	5.19051
CLIP4	8.58366
CLK1	6.762028
CLK2	7.358732
CLK2P	7.059336
CLK3	4.82687
CLK4	5.532952
CLK4	4.530094
CLLU1	2.542144
CLLU1OS	3.268634
CLMN	6.878932
CLN3	7.417834
CLN5	5.502688
CLN6	7.98772
CLN8	5.379328
CLN8	4.86393
CLNK	2.77477
CLNS1A	7.404794

CLOCK	5.871424
CLP1	6.591096
CLPB	6.507556
CLPP	7.151108
CLPS	4.091228
CLPTM1	8.924006
CLPTM1L	9.616134
CLPX	7.83163
CLRN1	2.510442
CLRN2	3.340588
CLRN3	2.750658
CLSPN	6.152422
CLSPN	7.193522
CLSTN1	8.347962
CLSTN2	3.767548
CLSTN3	7.093724
CLTA	7.526534
CLTB	8.424256
CLTC	9.505636
CLTCL1	5.688174
CLU	7.701026
CLUAP1	4.707586
CLUL1	2.847142
CLVS1	3.020172
CLVS2	2.582032
CLYBL	3.398486
CMA1	3.103926
CMAH	4.60446
CMAS	8.70219
CMBL	3.919576
CMC1	4.327422
CMIP	7.137158
CMKLR1	2.995632
CMPK1	7.695148
CMPK2	7.49945
CMTM1	5.858238
CMTM2	3.414608
CMTM3	4.723062
CMTM4	7.12645
CMTM5	3.875468
CMTM6	7.728624
CMTM7	7.935182
CMTM8	6.43751

CMYA5	5.922082
CNBD1	2.38915
CNBP	10.25486
CNDP1	3.47258
CNDP2	7.203544
CNFN	4.499976
CNGA1	2.61536
CNGA1	2.771874
CNGA2	3.10121
CNGA3	3.90273
CNGA4	3.638754
CNGB1	4.626474
CNGB3	2.789452
CNIH	8.339176
CNIH2	5.08402
CNIH3	3.149558
CNIH4	8.839386
CNKSR1	6.516422
CNKSR2	3.431884
CNKSR3	4.20836
CNN1	3.912048
CNN2	7.742094
CNN3	7.398348
CNNM1	4.367644
CNNM2	5.452048
CNNM3	6.15946
CNNM4	7.098978
CNO	5.891564
CNOT1	9.454512
CNOT10	7.79217
CNOT2	7.95492
CNOT3	7.029774
CNOT4	6.322544
CNOT6	8.019942
CNOT6L	6.221832
CNOT6L	5.145762
CNOT7	8.527378
CNOT8	8.779528
CNP	8.203246
CNPY1	2.672124
CNPY2	7.316764
CNPY3	7.965766
CNPY4	4.17754

CNR1	2.59649
CNR2	3.48328
CNRIP1	3.269624
CNST	4.350442
CNTD1	2.819732
CNTD2	5.04917
CNTFR	4.150172
CNTLN	4.268844
CNTN1	2.29973
CNTN2	4.106464
CNTN3	2.60165
CNTN4	2.433544
CNTN5	2.452082
CNTN6	2.607194
CNTNAP1	3.78983
CNTNAP2	2.941978
CNTNAP3	5.251722
CNTNAP3	5.338124
CNTNAP3	5.262926
CNTNAP3	5.915094
CNTNAP3	5.262926
CNTNAP4	2.887434
CNTNAP5	2.709138
CNTROB	6.67676
COASY	8.719852
COBL	5.189316
COBLL1	6.188522
COBRA1	7.583522
COCH	5.050804
COG1	6.800244
COG2	6.640206
COG3	6.851892
COG4	8.83972
COG5	7.406434
COG6	7.731618
COG7	7.22624
COG8	7.186746
COIL	8.172462
COL10A1	2.917964
COL11A1	3.227378
COL11A2	5.15048
COL11A2	3.504834
COL11A2	3.504834

COL12A1	6.856312
COL13A1	5.027158
COL14A1	3.162952
COL15A1	4.11225
COL16A1	4.990898
COL17A1	5.37801
COL18A1	6.214098
COL19A1	3.298406
COL1A1	5.711304
COL1A2	4.511008
COL20A1	4.85316
COL21A1	2.701496
COL22A1	4.214164
COL23A1	5.702802
COL24A1	2.826242
COL25A1	3.877496
COL27A1	4.798134
COL28A1	3.522994
COL2A1	4.707336
COL3A1	4.252936
COL4A1	4.768
COL4A2	5.503918
COL4A3	3.1471
COL4A3BP	6.568034
COL4A4	3.466888
COL4A5	4.645908
COL4A6	3.80597
COL5A1	5.491094
COL5A2	4.885756
COL5A3	5.333736
COL6A1	6.981906
COL6A2	6.9224
COL6A3	2.886598
COL6A5	3.043408
COL6A6	2.68494
COL7A1	4.779032
COL8A1	2.429518
COL8A2	4.462104
COL9A1	3.57661
COL9A2	4.820712
COL9A3	5.979454
COLEC10	3.63286
COLEC11	5.461174

COLEC12	3.648134
COLQ	3.878798
COMMD1	6.881492
COMMD10	5.653842
COMMD2	8.753344
COMMD3	6.875728
COMMD4	7.262266
COMMD5	6.506466
COMMD6	6.604718
COMMD7	7.660852
COMMD8	4.485728
COMMD9	3.14657
COMMD9	6.796246
COMP	3.743146
COMT	9.253298
COMTD1	6.081842
COPA	10.37198
COPB1	8.477264
COPB2	9.876432
COPE	8.086012
COPG	9.340488
COPG2	5.297248
COPS2	7.895184
COPS3	8.973366
COPS4	7.238852
COPS5	6.501722
COPS6	9.996462
COPS7A	9.049454
COPS7B	6.992686
COPS8	5.09946
COPZ1	10.019058
COPZ2	6.062886
COQ10A	5.664812
COQ10B	8.719274
COQ2	7.90379
COQ3	5.595668
COQ4	7.624364
COQ5	6.711976
COQ6	6.850534
COQ6	4.68521
COQ7	7.03666
COQ9	7.949314
CORIN	2.858944

CORO1A	5.29815
CORO1B	6.77483
CORO1C	10.25986
CORO2A	7.937528
CORO2B	3.17285
CORO6	4.342554
CORO7	5.288956
COTL1	8.414518
COX1	12.56632
COX10	7.0412
COX11	6.308726
COX11	5.863518
COX15	7.252022
COX16	8.167902
COX17	9.063106
COX17	9.581546
COX18	6.554702
COX19	6.582234
COX2	12.72416
COX3	12.29038
COX4I1	7.015786
COX4I2	4.057778
COX4NB	7.295636
COX5A	8.489446
COX5B	7.306692
COX6A1	10.226812
COX6A1	9.61793
COX6A2	5.299412
COX6B1	8.93504
COX6B2	4.020852
COX6C	9.798588
COX7A1	3.34193
COX7A2	9.120162
COX7A2L	8.365162
COX7B	7.073194
COX7B2	2.73945
COX7C	7.906788
COX8A	8.617708
COX8C	2.791058
CP	9.1496
CP110	5.447924
CPA1	3.92848
CPA2	2.444662

CPA3	2.441914
CPA4	5.40167
CPA5	2.719848
CPA6	2.400598
CPAMD8	5.785696
CPB1	2.638048
CPB2	2.459338
CPD	8.20149
CPE	2.905182
CPEB1	4.107488
CPEB2	5.188974
CPEB3	5.5902
CPEB4	6.225624
CPLX1	5.06643
CPLX2	3.953756
CPLX3	4.005656
CPLX3	3.511198
CPLX4	2.764964
CPM	5.781334
CPN1	3.125
CPN2	3.83074
CPNE1	9.437024
CPNE2	7.555322
CPNE3	8.676156
CPNE4	2.755718
CPNE5	3.62741
CPNE6	3.947532
CPNE7	5.038878
CPNE8	2.528554
CPNE9	3.330662
CPO	2.971172
CPOX	5.018792
CPPED1	6.141178
CPS1	2.993888
CPS1-IT	2.426812
CPSF1	7.437826
CPSF2	8.866164
CPSF3	8.163692
CPSF3L	7.20869
CPSF4	7.144364
CPSF4L	3.182104
CPSF6	8.088758
CPSF7	7.570808

CPT1A	7.700128
CPT1C	3.906456
CPT2	6.198932
CPT2	8.655834
CPVL	7.219368
CPXCR1	2.331228
CPXM1	3.007846
CPXM2	3.81713
CPZ	5.12505
CR1	2.56138
CR1L	2.246944
CR2	2.570922
CRABP1	4.194168
CRABP1	3.629144
CRABP2	9.614372
CRADD	4.361966
CRAMP1L	4.857294
CRAT	5.346472
CRB1	2.944108
CRB2	4.841842
CRB3	6.412028
CRBN	7.86287
CRCP	9.04983
CRCT1	3.180148
CREB1	8.094612
CREB3	7.250924
CREB3L1	3.761582
CREB3L2	5.610876
CREB3L3	4.25239
CREB3L4	5.912524
CREB5	3.262952
CREBBP	7.52654
CREBL2	7.471872
CREBZF	7.33816
CREG1	8.409184
CREG2	4.433196
CRELD1	5.508266
CRELD2	6.760274
CREM	4.796822
CRH	2.689044
CRHBP	3.665948
CRHR1	4.073722
CRHR2	3.712028

CRIM1	8.16367
CRIP1	4.533872
CRIP2	7.748876
CRIP3	4.771144
CRIPAK	7.138206
CRIPT	7.63197
CRISP1	3.149564
CRISP2	3.033878
CRISP3	2.640004
CRISPLD1	2.399292
CRISPLD2	5.028186
CRK	8.309594
CRKL	9.08623
CRLF1	6.023112
CRLF2	3.451362
CRLF3	7.634376
CRLS1	6.224348
CRMP1	3.848414
CRNKL1	6.21168
CRNN	3.354836
CROCC	4.85524
CROCCP2	4.475792
CROCCP3	4.3765
CROT	5.081108
CRP	2.77021
CRSP8P	7.776484
CRTAC1	3.119004
CRTAM	2.719124
CRTAP	7.472502
CRTC1	6.552224
CRTC2	5.873964
CRTC3	6.007376
CRX	3.16601
CRY1	5.428888
CRY2	5.098642
CRYAA	5.85768
CRYAB	6.513962
CRYBA1	2.981738
CRYBA2	3.842006
CRYBA4	4.07097
CRYBB1	4.172998
CRYBB2	4.704182
CRYBB2P1	6.307566

CRYBB3	5.579594
CRYGA	2.828196
CRYGB	2.333616
CRYGC	2.942766
CRYGD	2.784676
CRYGN	5.042874
CRYGS	4.58625
CRYL1	6.007522
CRYM	4.076152
CRYZ	3.754774
CRYZL1	5.178292
CS	8.558306
CSAD	6.045924
CSAG1	2.838106
CSAG2	5.557178
CSAG2	5.59169
CSDA	9.545348
CSDC2	4.932536
CSDE1	10.317094
CSE1L	9.363954
CSF1	6.24081
CSF1R	3.918106
CSF2	3.544398
CSF2RA	3.515754
CSF2RA	3.515754
CSF2RB	3.750528
CSF3	6.158504
CSF3R	3.220586
CSGALNACT1	5.891598
CSGALNACT2	6.877962
CSH1	4.629232
CSH2	4.427314
CSHL1	4.986416
CSK	6.771994
CSMD1	3.274874
CSMD2	3.492078
CSMD3	2.665958
CSN1S1	2.292106
CSN1S2AP	2.602716
CSN2	2.7813
CSN3	2.870236
CSNK1A1	10.48084
CSNK1A1L	4.493918

CSNK1A1P1	4.793198
CSNK1D	4.469048
CSNK1D	9.066248
CSNK1E	7.653474
CSNK1G1	5.54199
CSNK1G1	4.933174
CSNK1G2	6.70086
CSNK1G3	5.026252
CSNK2A1	9.767982
CSNK2A1P	8.021604
CSNK2A2	7.41096
CSNK2B	11.10998
CSNK2B	11.19832
CSNK2B	10.99934
CSPG4	3.86719
CSPG4P1Y	3.428086
CSPG4P1Y	3.428086
CSPG4P5	3.086234
CSPG5	3.658956
CSPP1	5.36853
CSRNP1	5.39441
CSRNP2	6.59027
CSRNP3	3.138566
CSRP1	9.457898
CSRP2	5.432298
CSRP2BP	5.515894
CSRP3	6.428396
CST1	2.665392
CST11	2.703482
CST2	4.226758
CST3	5.880542
CST4	5.211064
CST5	3.57631
CST6	5.555264
CST7	4.591006
CST8	2.89354
CST9	3.216708
CST9L	2.578952
CSTA	4.003982
CSTB	11.27698
CSTF1	7.086986
CSTF2	6.402862
CSTF2T	6.144606

CSTF3	6.963176
CSTL1	2.599228
CSTT	2.95859
CT45A1	2.669302
CT45A2	2.745946
CT45A3	2.728726
CT45A4	2.725222
CT45A5	2.719922
CT45A6	2.665152
CT47A1	4.306946
CT47A1	4.306946
CT47A1	4.306946
CT47A1	4.306946
CT47A1	4.44936
CT47A1	4.306946
CT47A1	4.306946
CT47A1	4.306946
CT47A1	4.306946
CT47A10	4.306946
CT47B1	4.30002
CTAG1A	5.44277
CTAG1A	5.44277
CTAG2	6.419516
CTAGE1	3.087052
CTAGE10P	2.613158
CTAGE15P	4.843374
CTAGE3P	2.350306
CTAGE4	4.834472
CTAGE4	4.049102
CTAGE5	5.752364
CTAGE6P	3.50262
CTAGE7P	2.58092
CTBP1	8.102346
CTBP2	6.221622
CTBS	6.279588
CTCF	8.520868
CTCFL	7.303192
CTCFL	3.26013
CTDNEP1	7.476334
CTDP1	5.69304
CTDSP1	6.289462
CTDSP2	8.03849
CTDSPL	7.448818

CTDSPL2	7.923012
CTF1	5.188568
CTGF	5.238334
CTH	5.172508
CTHRC1	4.97098
CTLA4	2.7722
CTNNA1	9.972028
CTNNA2	2.814064
CTNNA3	2.972416
CTNNAL1	6.247344
CTNNB1	9.826328
CTNNBIP1	6.880428
CTNNBL1	7.009854
CTNND1	9.50233
CTNND2	4.048086
CTNS	5.948084
CTPS	8.603008
CTPS2	6.837552
CTR9	6.95285
CTRB1	5.765402
CTRB2	5.218338
CTRC	3.60375
CTRL	4.426386
CTSA	9.235694
CTSB	9.141426
CTSB	4.937984
CTSC	8.34601
CTSD	10.027814
CTSE	3.862882
CTSF	4.126884
CTSG	3.222614
CTSH	7.740294
CTSK	3.146816
CTSL1	8.83357
CTSL2	8.815092
CTSL3	3.649524
CTSO	3.77046
CTSS	5.357402
CTSW	4.055122
CTSZ	8.216534
CTTN	9.517888
CTTNBP2	2.943482
CTTNBP2NL	5.723894

CTU1	5.990004
CTU2	5.724544
CTXN1	5.934084
CTXN3	2.71154
CUBN	2.63422
CUEDC1	6.595018
CUEDC2	6.336564
CUL1	8.760712
CUL2	7.346912
CUL3	8.018968
CUL4A	7.896594
CUL4B	6.202218
CUL5	5.896282
CUL7	5.684872
CUL9	5.829208
CUTA	8.845224
CUTC	6.589036
CUX1	6.734878
CUX2	4.004712
CUZD1	4.074774
CWC15	5.014138
CWC22	5.905504
CWC25	6.52942
CWC27	4.823218
CWF19L1	7.47462
CWF19L2	4.490318
CWH43	3.099488
CX3CL1	4.90242
CX3CR1	2.63303
CXADR	7.440474
CXCL1	5.071956
CXCL10	4.264404
CXCL11	2.519416
CXCL12	3.128142
CXCL13	2.414538
CXCL14	4.503518
CXCL16	8.089154
CXCL17	4.112406
CXCL2	3.688172
CXCL3	4.166208
CXCL5	4.420438
CXCL6	3.090086
CXCL9	2.713216

CXCR1	2.499792
CXCR2	2.575674
CXCR3	5.019226
CXCR4	2.78463
CXCR5	2.766324
CXCR6	2.898896
CXCR7	2.629002
CXorf1	2.528114
CXorf18	4.971752
CXorf18	6.662098
CXorf19	2.643794
CXorf21	2.132454
CXorf22	2.182854
CXorf23	3.08249
CXorf24	4.719834
CXorf26	2.623176
CXorf27	3.223848
CXorf36	2.847556
CXorf38	6.751958
CXorf40A	5.60789
CXorf40B	6.462232
CXorf41	2.285168
CXorf48	3.240408
CXorf56	5.510944
CXorf57	2.35139
CXorf58	2.359492
CXorf59	2.422572
CXorf61	5.26056
CXorf64	2.792666
CXorf65	2.991332
CXorf66	2.583282
CXorf67	4.54132
CXXC1	5.115744
CXXC1P1	2.780746
CXXC4	2.949726
CXXC5	8.63914
CYB561	6.513774
CYB561D1	5.427984
CYB561D2	5.237464
CYB5A	7.9158
CYB5B	9.985008
CYB5D1	6.139894
CYB5D2	6.78477

CYB5R1	7.377984
CYB5R2	3.187174
CYB5R3	7.669744
CYB5R4	5.975432
CYB5RL	5.318768
CYBA	7.70366
CYBASC3	7.518528
CYBB	3.047964
CYBRD1	8.17128
CYC1	9.884576
CYCS	7.650454
CYFIP1	8.775942
CYFIP2	3.307954
CYGB	4.650582
CYHR1	8.10953
CYHR1	6.74339
CYLC1	2.585014
CYLC2	2.42143
CYLD	6.356654
CYorf15A	3.341308
CYorf15B	2.5028
CYP11A1	4.061652
CYP11B1	4.216376
CYP11B2	3.373156
CYP17A1	2.663544
CYP19A1	2.771498
CYP1A1	3.16337
CYP1A2	4.04148
CYP1B1	7.707042
CYP20A1	6.0099
CYP21A2	3.866188
CYP21A2	3.802174
CYP21A2	3.802174
CYP24A1	2.624246
CYP26A1	5.155134
CYP26B1	4.637476
CYP26C1	4.943678
CYP27A1	6.813838
CYP27B1	4.126302
CYP27C1	4.318976
CYP2A13	3.706018
CYP2A6	3.648208
CYP2A7	3.740464

CYP2B6	2.683434
CYP2B7P1	3.552666
CYP2C18	2.628088
CYP2C19	3.27854
CYP2C8	2.555136
CYP2C9	3.155596
CYP2D6	4.594202
CYP2D7P1	4.74236
CYP2E1	4.207914
CYP2F1	4.536338
CYP2G1P	3.165622
CYP2J2	3.983916
CYP2R1	4.965146
CYP2S1	5.03269
CYP2U1	3.442258
CYP2W1	4.79431
CYP39A1	2.638874
CYP3A4	2.447216
CYP3A43	2.50432
CYP3A5	3.10491
CYP3A7	2.610066
CYP46A1	4.25722
CYP4A11	3.23721
CYP4A22	3.21406
CYP4B1	5.884294
CYP4F11	6.779718
CYP4F12	6.036404
CYP4F2	3.868346
CYP4F22	3.752148
CYP4F3	7.306174
CYP4F8	3.526898
CYP4V2	5.228442
CYP4X1	3.29103
CYP4Z1	2.763524
CYP4Z2P	2.5227
CYP51A1	7.10189
CYP7A1	2.31651
CYP7B1	3.082502
CYP8B1	2.84347
CYR61	5.498192
CYS1	6.363084
CYSLTR1	2.162106
CYSLTR2	2.463334

CYTB	12.4525
CYTH1	6.671266
CYTH2	8.560354
CYTH2	5.893122
CYTH3	7.692882
CYTH4	4.054008
CYTIP	2.838206
CYTL1	4.50492
CYYR1	2.80289
D2HGDH	5.13537
D2HGDH	5.121884
D4S234E	3.409496
DAAM1	5.912022
DAAM2	3.147892
DAB1	3.170716
DAB1	2.334374
DAB1	3.835322
DAB2	2.90305
DAB2IP	6.516654
DACH1	2.70554
DACH2	3.318872
DACT1	3.623708
DACT2	3.897686
DACT3	5.509348
DAD1	11.49504
DAG1	7.677302
DAGLA	4.984108
DAGLB	7.47923
DAK	6.988738
DALRD3	5.80504
DAND5	4.055132
DAO	2.27727
DAOA	2.388378
DAOA	3.186462
DAP	9.37576
DAP3	8.383006
DAPK1	8.977798
DAPK2	6.194268
DAPK3	5.818364
DAPL1	2.981278
DAPP1	5.171796
DARC	3.537156
DARS	8.629844

DARS2	8.62839
DAXX	6.619654
DAXX	6.848898
DAXX	6.708254
DAZ1	2.649918
DAZ1	2.649918
DAZ2	2.649918
DAZ2	2.817648
DAZAP1	7.457876
DAZAP2	9.984472
DAZL	3.974778
DBC1	3.201444
DBF4	3.499334
DBF4B	5.204342
DBF4B	5.523084
DBH	4.18926
DBI	8.773634
DBN1	6.412064
DBNDD1	5.998528
DBNDD2	6.589076
DBNL	7.898212
DBP	6.64858
DBR1	6.528504
DBT	6.19558
DBX1	4.914774
DBX2	2.735276
DCAF10	6.85905
DCAF11	7.626432
DCAF12	8.72365
DCAF12L1	3.348046
DCAF12L2	4.439268
DCAF13	6.452834
DCAF15	7.731952
DCAF16	7.789324
DCAF17	5.158038
DCAF4	6.726936
DCAF4L1	2.881794
DCAF4L2	3.831344
DCAF5	7.764082
DCAF6	7.747334
DCAF7	8.432956
DCAF8	7.31953
DCAF8L1	3.624576

DCAKD	7.12929
DCBLD1	7.387148
DCBLD2	9.370558
DCC	2.736696
DCD	3.581602
DCDC1	2.408754
DCDC2	3.20188
DCDC5	2.87855
DCHS1	3.887012
DCHS2	2.76409
DCI	8.119684
DCK	5.983846
DCLK1	2.98487
DCLK2	3.803382
DCLK3	2.694216
DCLRE1A	5.515094
DCLRE1B	5.390066
DCLRE1C	5.130904
DCN	2.362338
DCP1A	6.736688
DCP1B	5.95604
DCP2	6.028646
DCPS	7.418544
DCST1	4.132596
DCST2	3.977996
DCT	2.436312
DCTD	9.460374
DCTN1	7.967448
DCTN2	8.192394
DCTN3	8.263904
DCTN4	8.630574
DCTN5	9.825624
DCTN6	5.765418
DCTPP1	10.7549
DCUN1D1	9.01155
DCUN1D2	6.493034
DCUN1D3	6.856748
DCUN1D4	6.451666
DCUN1D5	8.385806
DCX	2.937172
DCXR	8.785482
DDA1	8.82152
DDAH1	6.135802

DDAH2	7.073734
DDAH2	7.10575
DDAH2	7.10575
DDB1	9.481178
DDB2	7.274984
DDC	4.231236
DDC	6.433966
DDHD1	5.30391
DDHD2	6.858454
DDI1	2.659138
DDI2	6.7738
DDIT3	7.360066
DDIT4	9.47459
DDIT4L	5.22614
DDN	4.33015
DDO	3.890254
DDOST	9.010948
DDR1	8.1801
DDR1	8.242108
DDR1	8.195592
DDR2	3.387112
DDR2	2.805238
DDRGK1	8.189378
DDT	8.020554
DDT	8.37757
DDX1	9.515966
DDX10	5.441512
DDX11	7.494284
DDX11L2	4.999042
DDX12	7.466536
DDX12	7.463686
DDX17	9.906308
DDX18	8.188518
DDX19A	7.146752
DDX19B	7.992746
DDX20	5.796646
DDX21	8.448688
DDX23	8.882886
DDX24	8.37438
DDX25	2.7625
DDX26B	2.74173
DDX27	8.680834
DDX28	5.314094

DDX31	6.60145
DDX39	8.57435
DDX3X	9.882964
DDX3Y	2.815996
DDX4	2.739826
DDX41	9.28135
DDX42	8.765436
DDX43	2.632506
DDX46	7.205154
DDX47	8.579872
DDX49	7.27422
DDX5	9.906614
DDX50	7.22417
DDX51	5.035218
DDX52	7.608394
DDX53	2.906616
DDX54	5.42248
DDX54	7.485306
DDX55	7.182486
DDX56	8.592042
DDX58	8.328736
DDX59	5.275678
DDX6	9.28891
DDX60	8.20388
DDX60L	8.307926
DEAF1	5.92719
1-Dec	2.796096
DECR1	8.050278
DECR2	5.890368
DEDD	7.919582
DEDD2	6.809562
DEF6	5.484552
DEF8	5.27914
DEFA3	2.897598
DEFA3	2.897598
DEFA3	2.897598
DEFA4	3.253768
DEFA5	3.34131
DEFA6	2.846096
DEFB1	5.227562
DEFB103A	2.823492
DEFB103A	2.823492
DEFB104A	2.553604

DEFB104A	2.553604
DEFB105A	2.538706
DEFB105A	2.538706
DEFB106A	2.070652
DEFB106A	2.070652
DEFB107A	2.163254
DEFB107A	2.163254
DEFB108B	2.571488
DEFB108B	2.571488
DEFB109P1B	2.586692
DEFB109P1B	2.586692
DEFB109P1B	2.987662
DEFB109P1B	2.665672
DEFB110	2.426364
DEFB112	2.29195
DEFB113	3.377708
DEFB114	2.176672
DEFB115	2.862238
DEFB116	2.586114
DEFB118	3.02271
DEFB119	2.79038
DEFB121	2.377664
DEFB123	3.539744
DEFB124	4.24413
DEFB125	2.754812
DEFB126	2.57929
DEFB127	2.378582
DEFB128	2.125138
DEFB129	2.403596
DEFB130	2.232466
DEFB130	2.232466
DEFB131	2.245084
DEFB132	4.2933
DEFB133	2.30827
DEFB134	2.38268
DEFB135	2.328418
DEFB136	2.295288
DEFB4A	2.977214
DEFB4A	2.977214
DEFT1P	4.254484
DEFT1P	4.254484
DEGS1	7.937328
DEGS2	6.439756

DEK	9.12479
DEM1	5.992084
DENND1A	7.010754
DENND1B	5.837018
DENND1B	6.231666
DENND1C	3.838624
DENND2A	3.910038
DENND2C	2.87414
DENND2C	5.260142
DENND2C	2.751644
DENND2C	2.400896
DENND2C	3.173424
DENND2C	2.696774
DENND2C	3.766632
DENND2C	4.158382
DENND2D	7.520512
DENND3	8.051248
DENND4A	5.413072
DENND4B	6.138578
DENND4C	7.271548
DENND5A	6.5701
DENND5B	4.473642
DENR	7.261944
DEPDC1	6.628876
DEPDC1B	6.52838
DEPDC4	2.895932
DEPDC5	5.59809
DEPDC6	5.782306
DEPDC7	3.17565
DERA	8.433936
DERL1	9.482896
DERL2	8.483942
DERL3	4.581592
DES	5.212974
DET1	3.829832
DEXI	7.535378
DEXI	7.535378
DEXI	5.151448
DFFA	7.784336
DFFB	5.07494
DFNA5	2.946288
DFNB31	4.816124
DGAT1	7.296344

DGAT1	7.296344
DGAT2	5.816862
DGAT2L6	2.447054
DGCR14	5.225258
DGCR14	6.341104
DGCR2	8.089132
DGCR5	4.436408
DGCR6	5.944278
DGCR6L	6.056848
DGCR6L	4.313254
DGCR8	7.533
DGKA	8.419254
DGKB	2.527132
DGKD	5.993634
DGKE	4.981396
DGKG	4.04197
DGKH	6.530034
DGKI	2.830786
DGKK	2.984278
DGKQ	5.168612
DGKZ	5.75001
DGKZ	6.33071
DGUOK	6.92862
DHCR24	10.237026
DHCR7	9.799246
DHDDS	8.584074
DHDH	4.891642
DHDPSL	4.92167
DHFR	8.6671
DHFR	8.626268
DHFRL1	5.322034
DHH	3.67718
DHODH	5.707978
DHPS	8.210664
DHRS1	8.339966
DHRS11	7.673454
DHRS12	4.76825
DHRS13	7.311336
DHRS2	4.102616
DHRS3	8.660332
DHRS4	7.229174
DHRS4L2	7.12804
DHRS7	7.16625

DHRS7B	6.506322
DHRS7C	3.71442
DHRS9	4.575012
DHTKD1	8.449694
DHX15	9.309622
DHX16	7.466752
DHX16	7.48119
DHX29	6.078504
DHX30	6.914092
DHX32	8.225404
DHX33	6.819492
DHX34	5.694768
DHX35	6.142512
DHX36	7.74856
DHX37	6.2555
DHX37	11.58314
DHX38	7.057994
DHX40	5.730514
DHX57	5.637024
DHX58	7.441078
DHX8	8.450328
DHX9	8.63977
DIABLO	6.517738
DIAPH1	8.722662
DIAPH2	4.340172
DIAPH3	7.634276
DICER1	6.592484
DIDO1	7.33344
DIMT1L	6.992906
DIO1	3.115232
DIO2	4.630746
DIO3	4.409358
DIP2A	5.808396
DIP2B	7.77187
DIP2C	5.292784
DIRAS1	5.411504
DIRAS2	3.296444
DIRAS3	3.328052
DIRC1	3.012988
DIRC2	7.924772
DIS3	6.463394
DIS3L	7.004838
DIS3L2	6.41627

DISC1	3.031026
DISP1	4.450658
DISP2	4.829198
DIXDC1	4.60791
DKC1	8.473806
DKFZP434F14	4.79208
DKFZP434I07	5.194984
DKFZP434K0	4.183034
DKFZP434L18	3.09144
DKFZp451A2	4.132692
DKFZP686I15	4.23021
DKFZp686O2	5.374894
DKFZp761E19	5.94143
DKFZp761P0	4.62467
DKFZP779L18	3.120212
DKFZp779M0	5.298434
DKK1	7.763848
DKK2	2.95405
DKK3	2.795594
DKK3	4.788012
DKK4	2.984512
DKKL1	3.586886
DLAT	8.413978
DLC1	3.405124
DLD	8.19468
DLEC1	3.855356
DLEU1	5.683104
DLEU2	6.507218
DLEU2L	2.178456
DLEU7	5.634356
DLG1	8.889754
DLG2	2.68706
DLG3	6.303104
DLG4	3.60822
DLG5	6.574994
DLGAP1	4.368646
DLGAP2	3.46063
DLGAP3	3.794396
DLGAP4	5.805202
DLGAP5	7.781228
DLK1	4.8041
DLK2	4.948258
DLL1	4.50158

DLL3	3.814622
DLL4	4.614552
DLST	8.183566
DLX1	5.042174
DLX2	4.229946
DLX3	4.489316
DLX4	5.05897
DLX5	3.60209
DLX6	5.794596
DMAP1	6.289682
DMBT1	3.65082
DMBX1	5.219238
DMC1	3.094588
DMD	2.690798
DMGDH	2.924934
DMKN	7.168706
DMP1	2.719492
DMPK	6.786914
DMRT1	3.658212
DMRT2	2.960548
DMRT3	3.499906
DMRTA1	5.346488
DMRTA2	6.158446
DMRTB1	5.168938
DMRTC1	4.295142
DMRTC1	3.967448
DMRTC2	3.75642
DMTF1	6.187732
DMWD	6.272768
DMXL1	4.688972
DMXL2	5.199272
DNA2	5.579468
DNAH1	3.768838
DNAH10	3.00281
DNAH10	3.657448
DNAH11	3.79756
DNAH12	2.473964
DNAH12	2.469058
DNAH12	2.409472
DNAH14	3.58213
DNAH14	2.186072
DNAH14	2.43428
DNAH17	4.095728

DNAH17	3.216768
DNAH17	2.603392
DNAH2	4.073478
DNAH3	2.791288
DNAH5	2.939274
DNAH6	2.452926
DNAH6	3.46035
DNAH6	2.553338
DNAH6	2.404574
DNAH7	2.772042
DNAH8	2.404412
DNAH9	3.033478
DNAI1	3.537052
DNAI2	4.072268
DNAJA1	8.274634
DNAJA2	2.674616
DNAJA2	7.94337
DNAJA3	8.049816
DNAJA4	3.732316
DNAJB1	10.79142
DNAJB11	10.134486
DNAJB12	6.30625
DNAJB13	3.449236
DNAJB13	3.565216
DNAJB14	5.284304
DNAJB14	5.292914
DNAJB2	5.651282
DNAJB3	3.769372
DNAJB4	5.765252
DNAJB5	5.270464
DNAJB6	8.204574
DNAJB7	2.529896
DNAJB8	3.590168
DNAJB9	5.035458
DNAJC1	7.768568
DNAJC10	7.02606
DNAJC11	7.684988
DNAJC12	4.660884
DNAJC13	7.857136
DNAJC14	6.652754
DNAJC15	4.877596
DNAJC16	7.075054
DNAJC17	5.453138

DNAJC18	4.340708
DNAJC19	8.671526
DNAJC19	6.789486
DNAJC2	5.777512
DNAJC21	7.376576
DNAJC22	4.774432
DNAJC24	4.506262
DNAJC25	6.937896
DNAJC27	4.226982
DNAJC28	2.833086
DNAJC3	7.789024
DNAJC30	6.9298
DNAJC4	6.539868
DNAJC4	3.97672
DNAJC5	7.774398
DNAJC5B	2.854394
DNAJC5G	3.043076
DNAJC6	2.990216
DNAJC7	8.611998
DNAJC8	7.749018
DNAJC9	5.447576
DNAJC9	7.21562
DNAL1	3.789578
DNAL4	5.387504
DNALI1	3.424346
DNASE1	4.783842
DNASE1L1	5.466136
DNASE1L2	3.907184
DNASE1L3	2.590278
DNASE2	7.907802
DNASE2B	2.616146
DND1	6.666494
DNER	5.498494
DNHD1	4.723708
DNHD1	4.76442
DNHD1	4.393114
DNHD1	4.117526
DNM1	5.429618
DNM1L	7.128742
DNM1P35	5.160704
DNM1P41	4.374002
DNM1P41	4.374002
DNM1P41	4.374002

DNM2	8.329958
DNM3	3.049472
DNMBP	4.805366
DNMT1	8.49352
DNMT3A	5.943726
DNMT3B	4.818674
DNMT3L	3.139098
DNPEP	7.060396
DNTT	2.78244
DNTTIP1	7.738856
DNTTIP2	5.669382
DOC2A	4.555882
DOC2B	4.396542
DOCK1	6.945118
DOCK10	2.475596
DOCK11	2.962872
DOCK2	2.869984
DOCK3	3.4595
DOCK4	4.056008
DOCK5	7.001762
DOCK6	4.05254
DOCK6	5.14055
DOCK7	6.05345
DOCK8	7.110658
DOCK9	7.404268
DOHH	6.603724
DOK1	5.158266
DOK2	3.941972
DOK3	4.650148
DOK4	5.562174
DOK5	2.568118
DOK6	3.117584
DOK7	5.557388
DOLK	6.708166
DOLPP1	7.293622
DOM3Z	5.105002
DOM3Z	5.105002
DOM3Z	5.350846
DONSON	7.94662
DOPEY1	5.067378
DOPEY2	5.584272
DOT1L	6.342664
DPAGT1	8.10634

DPCD	5.80236
DPCR1	3.442062
DPCR1	3.52101
DPEP1	4.442606
DPEP2	4.060824
DPEP3	4.074086
DPF1	4.068338
DPF2	9.25421
DPF3	3.655308
DPH1	6.73348
DPH2	5.93243
DPH3	6.214792
DPH3P1	5.02827
DPH5	6.940834
DPM1	9.188462
DPM2	7.098352
DPM3	7.351636
DPP10	2.630728
DPP3	8.576068
DPP4	3.740756
DPP6	2.932906
DPP7	7.339908
DPP8	7.07963
DPP9	6.312684
DPPA2	2.933778
DPPA3	2.965326
DPPA4	2.651154
DPPA5	3.37585
DPRX	3.095322
DPT	2.735364
DPY19L1	6.257814
DPY19L2	3.093762
DPY19L2P1	3.648206
DPY19L2P2	2.860358
DPY19L2P4	2.851404
DPY19L3	3.816222
DPY19L4	7.48709
DPY30	9.913382
DPYD	7.901046
DPYS	2.75463
DPYSL2	7.327552
DPYSL3	3.837022
DPYSL4	5.385308

DPYSL5	5.484054
DQX1	3.647116
DR1	7.525132
DRAM1	6.59911
DRAM2	7.847364
DRAP1	9.016106
DRD1	2.679188
DRD2	4.929874
DRD3	2.858832
DRD4	5.743114
DRD5	4.265602
DRD5	3.657248
DRD5	3.54567
DRD5	4.76814
DRD5	4.039298
DRG1	8.253978
DRG2	7.810472
DRGX	4.481404
DROSHA	7.391988
DRP2	3.190474
DSC1	3.577142
DSC2	9.628636
DSC3	6.286128
DSCAM	3.257268
DSCAML1	4.09023
DSCC1	6.38391
DSCR10	2.928092
DSCR3	8.305244
DSCR4	3.251316
DSCR6	5.390548
DSCR8	2.308622
DSCR9	3.281602
DSE	5.095898
DSEL	2.765486
DSERG1	2.142394
DSG1	2.65503
DSG2	7.441522
DSG3	4.598558
DSG4	3.157598
DSN1	7.9593
DSP	8.414184
DSPP	2.460976
DST	6.23404

DSTN	7.550098
DSTNP2	6.796296
DSTYK	4.839628
DTD1	6.799904
DTHD1	2.507458
DTL	8.845886
DTNA	3.087164
DTNB	6.236616
DTNBP1	6.70304
DTWD1	2.914308
DTWD2	5.545146
DTX1	4.28455
DTX2	6.323884
DTX3	4.443568
DTX3L	9.22263
DTX4	7.281804
DTYMK	4.98126
DTYMK	4.98126
DUOX1	3.179638
DUOX2	2.961598
DUOXA1	5.188986
DUOXA2	3.822984
DUPD1	4.025302
DUS1L	7.088606
DUS2L	7.146158
DUS3L	5.896596
DUS4L	5.237312
DUSP1	8.002502
DUSP10	5.37253
DUSP11	8.443156
DUSP12	6.997942
DUSP13	4.870108
DUSP14	7.269916
DUSP15	4.606138
DUSP16	8.309784
DUSP18	5.155238
DUSP19	3.35558
DUSP2	4.815956
DUSP21	2.791282
DUSP22	5.285696
DUSP23	7.044242
DUSP26	3.387494
DUSP27	3.558202

DUSP28	4.497146
DUSP3	7.04507
DUSP4	6.0488
DUSP5	7.47001
DUSP5P	2.893504
DUSP5P	3.498638
DUSP6	6.547412
DUSP7	6.228764
DUSP8	5.711348
DUSP9	7.095952
DUT	7.206882
DUX4	5.40354
DUX4	5.996502
DUX4	5.40354
DUX4	5.821808
DUX4L4	5.196032
DUX4L4	5.196032
DUX4L4	5.196032
DUX4L4	5.186618
DUX4L4	5.196032
DUX4L4	5.196032
DUX4L4	5.617638
DUX4L4	5.92215
DUX4L4	5.059252
DUXA	2.441694
DVL1	6.908094
DVL2	6.951222
DVL3	7.91986
DYDC1	2.316108
DYDC2	2.693976
DYM	5.86749
DYNC1H1	8.735952
DYNC1I1	3.412944
DYNC1I2	4.932408
DYNC1LI1	7.021028
DYNC1LI2	8.41101
DYNC2H1	2.398448
DYNC2H1	2.48074
DYNC2LI1	3.561408
DYNLL1	9.52275
DYNLL1	5.921528
DYNLL1	10.2189
DYNLL2	8.006628

DYNLRB1	7.3845
DYNLRB2	2.395922
DYNLT1	7.654638
DYNLT3	6.436954
DYRK1A	7.280182
DYRK1B	5.418166
DYRK2	6.689608
DYRK3	3.678826
DYRK4	3.801768
DYSF	3.558764
DYSFIP1	3.863472
DYX1C1	3.163708
DZIP1	6.688856
DZIP1L	3.616376
DZIP3	5.996922
E2F1	7.044806
E2F2	6.499406
E2F3	6.424824
E2F4	7.709932
E2F5	3.525584
E2F6	7.086722
E2F7	6.769834
E2F8	3.2259
E4F1	6.05679
EAF1	7.378732
EAF2	4.25164
EAPP	7.253672
EARS2	6.441798
EBAG9	6.633516
EBF1	3.148814
EBF2	2.869038
EBF3	3.697734
EBF4	5.115458
EBI3	4.130088
EBLN2	3.27104
EBNA1BP2	8.820348
EBP	8.333694
EBPL	8.849314
ECD	7.465904
ECE1	8.143706
ECE2	5.659516
ECEL1	4.164582
ECEL1P2	3.879578

ECH1	10.7954
ECHDC1	7.863658
ECHDC2	5.701972
ECHDC3	3.719388
ECHS1	10.020486
ECM1	4.750794
ECM2	3.209894
ECRP	3.659472
ECSIT	6.73982
ECT2	9.148396
EDA	3.12783
EDA2R	2.958106
EDAR	3.60079
EDARADD	4.390562
EDC3	7.39144
EDC4	6.936114
EDDM3A	2.363006
EDDM3B	2.428532
EDEM1	7.672758
EDEM1	4.36742
EDEM2	5.980502
EDEM3	6.013982
EDF1	9.444636
EDIL3	2.476538
EDN1	7.354382
EDN2	5.170698
EDN3	3.895116
EDNRA	2.405186
EDNRB	5.609688
EEA1	4.321166
EED	7.523664
EEF1A1	7.201592
EEF1A1	12.55586
EEF1A1	12.54608
EEF1A2	8.340308
EEF1B2	5.953528
EEF1D	11.06
EEF1D	5.706784
EEF1DP3	5.639338
EEF1E1	5.49874
EEF1G	11.904
EEF2	10.30745
EEF2K	5.99725

EEFSEC	7.319858
EEPD1	4.470616
EFCAB1	2.270722
EFCAB2	4.271124
EFCAB2	2.911018
EFCAB2	4.735134
EFCAB3	2.439208
EFCAB4A	5.855512
EFCAB4B	2.872904
EFCAB4B	3.830302
EFCAB4B	2.911386
EFCAB5	2.504152
EFCAB6	3.363584
EFCAB7	3.896948
EFEMP1	8.097106
EFEMP2	4.233108
EFHA1	6.52138
EFHA2	3.190088
EFHB	2.42668
EFHC1	5.213568
EFHC2	2.48175
EFHD1	4.991074
EFHD2	7.696452
EFNA1	6.151524
EFNA2	5.988482
EFNA3	5.635404
EFNA4	5.917592
EFNA5	6.677304
EFNB1	4.740636
EFNB2	6.042694
EFNB3	4.028002
EFR3A	8.927958
EFR3B	3.88164
EFS	4.474588
EFTUD1	6.184158
EFTUD1	4.449302
EFTUD2	9.068062
EGF	5.304062
EGFL6	2.938626
EGFL7	5.596054
EGFLAM	2.925752
EGFR	7.784882
EGLN1	7.011618

EGLN2	5.724314
EGLN3	5.12283
EGR1	6.834294
EGR2	3.978794
EGR3	3.790616
EGR4	6.01873
EHBP1	6.087142
EHBP1L1	4.659188
EHD1	6.649244
EHD2	6.244886
EHD3	4.42466
EHD4	8.173524
EHF	7.173486
EHHADH	6.335396
EHMT1	7.137564
EHMT2	7.060334
EHMT2	7.060334
EI24	9.27605
EID1	7.867368
EID2	6.794092
EID2B	4.704784
EID3	5.681928
EIF1	11.92238
EIF1AD	7.153022
EIF1AX	9.523778
EIF1AX	8.47199
EIF1AY	2.390442
EIF1B	5.374118
EIF2A	7.814294
EIF2AK1	10.20614
EIF2AK2	9.736094
EIF2AK3	6.277462
EIF2AK4	6.387684
EIF2B1	8.722928
EIF2B2	8.58528
EIF2B3	6.981134
EIF2B4	6.29909
EIF2B5	6.85536
EIF2C1	7.078674
EIF2C2	7.824016
EIF2C3	6.489586
EIF2C4	5.658342
EIF2S1	9.43866

EIF2S2	7.924998
EIF2S3	9.765702
EIF3A	8.84257
EIF3B	9.293636
EIF3C	10.88354
EIF3C	10.88354
EIF3D	10.082716
EIF3E	8.376648
EIF3F	5.51587
EIF3G	8.669166
EIF3H	9.786004
EIF3I	9.31375
EIF3J	7.648614
EIF3K	9.386562
EIF3L	9.788798
EIF3M	9.516054
EIF4A1	9.871294
EIF4A2	9.684308
EIF4A2	7.406606
EIF4A3	8.981046
EIF4B	9.664052
EIF4B	5.80119
EIF4B	8.793964
EIF4E	3.440426
EIF4E1B	5.853758
EIF4E2	7.792928
EIF4E3	4.815134
EIF4EBP1	6.938058
EIF4EBP2	8.325104
EIF4ENIF1	6.793122
EIF4G1	9.340082
EIF4G2	10.43408
EIF4G3	7.549144
EIF4H	10.035744
EIF4H	10.089122
EIF5	8.464492
EIF5A	4.83311
EIF5A2	5.714112
EIF5AL1	11.43174
EIF5AL1	11.4292
EIF5AL1	11.42108
EIF5B	7.732698
EIF6	9.271678

ELAC1	4.733288
ELAC2	8.373638
ELANE	5.438808
ELAVL1	6.78471
ELAVL2	3.362408
ELAVL3	3.32706
ELAVL4	2.741336
ELF1	9.54403
ELF2	6.884286
ELF3	8.393934
ELF4	7.279276
ELF5	3.600772
ELFN1	3.941604
ELFN2	5.222602
ELK1	6.144898
ELK3	5.42687
ELK4	6.136502
ELL	5.659816
ELL2	5.897732
ELL2	6.370008
ELL3	4.483894
ELMO1	2.863422
ELMO2	6.82159
ELMO3	6.234964
ELMOD1	2.573002
ELMOD2	5.208066
ELMOD3	5.369516
ELN	5.921066
ELOF1	8.32447
ELOVL1	8.798386
ELOVL2	2.944236
ELOVL3	3.521168
ELOVL4	2.531676
ELOVL5	9.52268
ELOVL6	6.816644
ELOVL7	7.077378
ELP1P	2.834844
ELP2	6.147072
ELP2P	4.10222
ELP3	8.137408
ELP4	6.441608
ELSPBP1	2.831372
ELTD1	2.222302

EMB	3.075536
EMCN	2.42151
EMD	8.050034
EME1	5.846334
EME2	5.985298
EMG1	8.218692
EMID1	5.065898
EMID2	4.607034
EMILIN1	4.6693
EMILIN2	4.083072
EMILIN3	3.468922
EML1	4.296232
EML2	7.131542
EML3	5.866856
EML4	7.885186
EML5	3.150292
EML6	4.379154
EMP1	10.6481
EMP2	9.97964
EMP3	6.814166
EMR1	3.20498
EMR2	4.271556
EMR3	2.781472
EMR4P	3.035332
EMX1	5.759994
EMX2	3.044724
EN1	4.852368
EN2	5.536236
ENAH	7.547386
ENAM	2.677334
ENC1	6.474288
ENDOD1	5.377922
ENDOG	6.239416
ENDOU	2.93346
ENG	6.412134
ENGASE	5.080836
ENHO	4.14197
ENKUR	2.179654
ENO1	12.26016
ENO2	5.341978
ENO3	3.441078
ENO4	2.75661
ENOPH1	8.75709

ENOSF1	7.513484
ENOX1	2.998492
ENOX2	7.138346
ENPEP	3.1285
ENPP1	2.41316
ENPP2	2.75367
ENPP3	2.581286
ENPP4	5.336212
ENPP5	4.83357
ENPP6	2.995122
ENPP7	5.157016
ENSA	6.219544
ENTHD1	2.912414
ENTPD1	3.08481
ENTPD2	4.60487
ENTPD3	2.83285
ENTPD4	7.2262
ENTPD5	5.764266
ENTPD6	7.762412
ENTPD7	5.248532
ENTPD8	5.169316
ENY2	7.721856
EOMES	4.938548
EP300	7.33153
EP400	6.166232
EP400NL	3.961594
EPAG	2.312388
EPAS1	8.293212
EPB41	5.934488
EPB41L1	6.740748
EPB41L2	7.561802
EPB41L3	2.701998
EPB41L4A	6.638482
EPB41L4B	5.303172
EPB41L5	5.1869
EPB42	3.690362
EPB49	5.661154
EPC1	7.007768
EPC2	6.224996
EPCAM	9.305828
EPCAM	9.844252
EPDR1	7.011282
EPGN	3.268552

EPHA1	6.801224
EPHA10	3.353526
EPHA10	4.809218
EPHA2	8.421278
EPHA3	2.745396
EPHA4	5.321864
EPHA5	2.998356
EPHA6	2.574044
EPHA7	2.955892
EPHA8	4.22638
EPHB1	3.592638
EPHB1	5.09809
EPHB2	5.862478
EPHB3	6.940178
EPHB4	7.869024
EPHB6	4.482412
EPHX1	8.761204
EPHX2	4.85217
EPHX3	3.536812
EPHX4	4.497908
EPM2A	3.272414
EPM2AIP1	6.172424
EPN1	6.737926
EPN2	6.572648
EPN3	5.949912
EPO	4.828214
EPOR	4.022596
EPRS	8.86192
EPS15	8.27529
EPS15L1	7.470876
EPS8	9.087024
EPS8L1	6.342716
EPS8L2	6.526602
EPS8L3	4.224156
EPSTI1	6.91394
EPT1	7.517942
EPX	3.40014
EPYC	2.728412
ERAL1	8.312606
ERAP1	6.630104
ERAP2	6.720582
ERAS	3.49876
ERBB2	7.619612

ERBB2IP	7.262416
ERBB3	7.323588
ERBB4	3.294906
ERC1	8.047222
ERC2	2.844358
ERCC1	6.406178
ERCC2	6.875284
ERCC3	8.177844
ERCC3	3.385602
ERCC4	5.990324
ERCC5	7.63633
ERCC6	5.72174
ERCC6L	4.097516
ERCC8	3.355816
EREG	8.223216
ERF	6.03334
ERG	2.908532
ERGIC1	7.857662
ERGIC2	6.136338
ERGIC3	9.784708
ERH	10.45176
ERI1	6.144548
ERI2	5.69085
ERI3	7.1378
ERICH1	6.225314
ERLEC1	6.406242
ERLIN1	8.02824
ERLIN2	5.6528
ERLIN2	4.30521
ERMAP	5.112502
ERMN	2.130288
ERMP1	9.03255
ERN1	5.581904
ERN2	3.408894
ERO1L	8.041438
ERO1LB	3.137702
ERP27	4.361466
ERP29	8.190296
ERP44	9.231922
ERRFI1	9.144224
ERV3	5.418546
ERVFRDE1	3.109724
ERVWE1	2.92173

ESAM	4.061702
ESCO1	6.348038
ESCO2	5.826536
ESD	5.05606
ESF1	4.617336
ESM1	2.326912
ESPL1	6.846244
ESPN	5.71269
ESPNL	4.843232
ESPNP	4.569766
ESR1	3.308964
ESR1	5.361826
ESR2	2.874736
ESRP1	10.73438
ESRP2	6.597514
ESRRA	6.963934
ESRRB	3.710434
ESRRG	2.347144
ESX1	2.815244
ESYT1	7.654946
ESYT2	9.499652
ESYT3	4.015502
ETAA1	4.409674
ETF1	7.2931
ETFA	8.462608
ETFB	8.005812
ETFDH	6.716016
ETHE1	7.496134
ETNK1	6.423742
ETNK2	4.131722
ETS1	7.367834
ETS2	8.340218
ETV1	5.843068
ETV2	4.208878
ETV3	7.70828
ETV3L	3.857812
ETV4	6.647
ETV5	7.05148
ETV6	7.685424
ETV7	4.324592
EVC	5.303194
EVC2	2.916446
EVI2A	2.193758

EVI2B	2.984712
EVI5	3.808828
EVI5L	5.37305
EVL	4.790428
EVPL	5.86071
EVX1	4.843506
EVX2	5.494768
EWSR1	9.557346
EXD1	2.724634
EXD2	7.453454
EXD3	4.940236
EXO1	8.974096
EXOC1	7.651362
EXOC2	6.828232
EXOC3	8.045402
EXOC3L	4.411484
EXOC3L2	5.047336
EXOC4	8.302188
EXOC5	2.29323
EXOC5	6.54312
EXOC5	4.273808
EXOC6	5.80224
EXOC6B	5.98565
EXOC7	6.994246
EXOC8	6.386842
EXOG	4.929002
EXOSC1	7.993648
EXOSC10	8.66479
EXOSC2	9.350532
EXOSC3	7.368792
EXOSC4	7.04862
EXOSC5	7.687372
EXOSC6	7.614302
EXOSC7	7.38456
EXOSC8	8.40139
EXOSC9	7.870752
EXPH5	5.028616
EXT1	8.821618
EXT2	7.562202
EXTL1	4.030716
EXTL2	3.237442
EXTL3	6.178442
EYA1	3.469876

EYA2	4.80671
EYA3	6.095768
EYA4	2.314208
EYS	2.215648
EYS	2.326804
EYS	2.755386
EYS	2.254984
EYS	2.173738
EZH1	6.137784
EZH2	9.091406
EZR	9.947426
F10	4.326148
F11	2.905698
F11R	9.78818
F12	4.221404
F13A1	2.636018
F13B	2.343498
F2	3.757168
F2R	4.467958
F2RL1	7.494724
F2RL2	2.69061
F2RL3	4.469586
F3	7.579172
F5	3.147962
F7	4.595068
F8	3.848814
F8A1	7.51016
F8A1	7.507022
F8A1	7.76971
F9	2.259528
FA2H	5.856788
FAAH	6.179314
FAAH2	3.716218
FABP1	2.624808
FABP12	2.292672
FABP2	2.576054
FABP3	3.38848
FABP4	2.137702
FABP5	4.51021
FABP5	4.55531
FABP6	4.83986
FABP7	2.619902
FADD	7.911022

FADS1	8.014162
FADS2	6.319046
FADS3	7.123982
FADS6	4.447194
FAF1	8.11494
FAF2	9.425016
FAH	6.377966
FAHD1	5.126882
FAHD2A	5.687988
FAHD2B	6.501498
FAIM	5.355758
FAIM2	3.573628
FAIM3	3.28755
FAM100A	5.732874
FAM100B	8.157672
FAM101A	4.27878
FAM101B	5.716392
FAM102A	8.309164
FAM102B	5.12619
FAM103A1	5.568922
FAM103A1	6.069366
FAM104A	8.258336
FAM104B	3.511224
FAM105A	4.536432
FAM105B	6.888764
FAM106A	4.99429
FAM106A	5.015256
FAM106C	4.97202
FAM107A	4.779
FAM107B	7.637986
FAM108A1	5.995026
FAM108A1	5.995026
FAM108A1	5.968632
FAM108A1	6.792254
FAM108A1	5.812492
FAM108B1	5.829744
FAM108C1	6.82668
FAM109A	5.40531
FAM109B	5.26419
FAM110A	6.833536
FAM110B	2.85408
FAM110C	6.081298
FAM111A	8.061356

FAM111B	6.775104
FAM113A	5.399152
FAM113B	4.53608
FAM114A1	7.78733
FAM114A2	6.558028
FAM115A	2.331344
FAM115A	4.476048
FAM115C	5.116826
FAM115C	5.37744
FAM116A	7.144662
FAM116B	5.29132
FAM117A	5.735392
FAM117B	4.237956
FAM118A	6.32631
FAM118B	7.50339
FAM119A	5.652586
FAM119B	4.867052
FAM120A	7.255388
FAM120A	9.225422
FAM120AOS	7.496588
FAM120B	6.301404
FAM120C	3.942406
FAM122A	6.75811
FAM122B	7.664162
FAM122C	6.449086
FAM123A	4.03729
FAM123B	4.596858
FAM123C	3.935164
FAM124A	3.298748
FAM124B	3.11981
FAM125A	7.045706
FAM125B	5.124802
FAM126A	5.768182
FAM126B	6.291968
FAM127A	8.355634
FAM127B	7.871978
FAM127C	6.461788
FAM129A	6.107684
FAM129B	8.753232
FAM129C	3.626536
FAM131A	4.761572
FAM131B	5.509248
FAM131C	5.16846

FAM132A	4.632232
FAM132B	4.890704
FAM133A	2.211736
FAM133B	6.42732
FAM133B	5.423592
FAM133B	5.72791
FAM134A	6.431966
FAM134B	7.324042
FAM134C	7.900736
FAM135A	5.140168
FAM135B	2.693714
FAM136A	8.083588
FAM136B	4.652974
FAM138A	2.633848
FAM138A	2.633848
FAM138A	2.837974
FAM138B	2.596568
FAM138D	2.470172
FAM13A	5.524746
FAM13B	6.42816
FAM13C	2.761174
FAM149A	3.048462
FAM149B1	5.737486
FAM150A	2.994778
FAM150B	5.163114
FAM151B	3.049026
FAM153B	3.262726
FAM153B	3.047692
FAM153C	3.236714
FAM154A	2.687896
FAM154B	2.793428
FAM155B	4.842236
FAM156A	5.77346
FAM156A	5.87856
FAM158A	7.42523
FAM159A	3.534574
FAM159B	2.398604
FAM160A1	7.525718
FAM160A2	5.862986
FAM160B1	6.268138
FAM160B2	5.958104
FAM161A	3.378986
FAM162A	6.751288

FAM162B	5.569168
FAM163A	3.089364
FAM163B	4.489554
FAM164A	4.340108
FAM164C	4.014262
FAM165B	5.924812
FAM167A	3.48923
FAM167B	4.106864
FAM168A	6.522836
FAM168B	8.266126
FAM169A	5.377894
FAM169B	3.094082
FAM170A	3.024546
FAM170B	4.605492
FAM171A1	4.43601
FAM171A2	4.68898
FAM171B	3.745776
FAM172A	4.702498
FAM172B	2.457462
FAM173A	6.413716
FAM173B	6.426058
FAM174A	4.924038
FAM174B	7.342594
FAM175A	3.226066
FAM175B	8.019668
FAM176A	4.971096
FAM177A1	6.17849
FAM177B	2.562306
FAM178A	5.652654
FAM178B	4.252756
FAM179A	4.210946
FAM179B	5.352688
FAM180A	3.521222
FAM180B	4.0337
FAM181A	3.8874
FAM181B	5.407548
FAM182A	3.313324
FAM182A	4.092426
FAM183A	3.627294
FAM183B	4.451588
FAM183B	3.99119
FAM184A	2.924056
FAM184B	4.451912

FAM185A	4.198528
FAM185A	5.958234
FAM186A	2.516412
FAM186B	2.91297
FAM187B	3.867976
FAM188A	5.642356
FAM188B	5.336662
FAM189A1	4.267262
FAM189A2	3.667902
FAM189B	6.528484
FAM18A	3.48634
FAM18B2	8.321114
FAM18B2	4.382356
FAM190A	3.234652
FAM190B	6.30144
FAM192A	8.517762
FAM193A	5.70694
FAM193B	5.671412
FAM194A	2.940536
FAM194B	2.540484
FAM195A	6.193292
FAM195B	5.665316
FAM196A	2.857476
FAM198A	4.00212
FAM198B	3.388276
FAM199X	6.820818
FAM19A1	2.19047
FAM19A2	2.933604
FAM19A3	5.081048
FAM19A4	3.417538
FAM19A5	5.45842
FAM200A	3.43041
FAM200B	3.959106
FAM201B	5.74066
FAM20A	3.13944
FAM20B	8.464472
FAM20C	6.409574
FAM21A	7.431362
FAM21B	7.375252
FAM21C	7.023402
FAM21C	6.489132
FAM22A	4.314154
FAM22A	4.665794

FAM22A	4.777876
FAM22A	4.35674
FAM22F	4.311606
FAM22G	4.63626
FAM23A	3.041434
FAM23A	3.041434
FAM24A	3.286424
FAM24B	6.864812
FAM25B	5.261784
FAM25B	5.261784
FAM25B	5.261784
FAM26D	2.30733
FAM26E	2.80961
FAM26F	4.065528
FAM27A	5.968044
FAM27A	4.65854
FAM27A	4.499074
FAM27A	5.967098
FAM27C	5.792818
FAM27D1	5.458194
FAM27E3	5.179674
FAM27E3	5.08768
FAM27E3	5.08768
FAM27E3	5.08768
FAM27E3	5.170548
FAM27E3	5.158726
FAM27L	3.764252
FAM32A	7.954604
FAM35A	2.319916
FAM35B	2.654016
FAM35B2	2.771234
FAM36A	6.04687
FAM36A	5.495194
FAM38A	4.740804
FAM38A	3.251774
FAM38A	7.026084
FAM38B	2.618196
FAM38B	3.327924
FAM38B	3.546692
FAM3A	7.09977
FAM3B	2.61034
FAM3C	7.851176
FAM3C	7.710292

FAM3D	3.917968
FAM40A	6.611852
FAM40B	6.158382
FAM43A	6.289776
FAM43B	6.210504
FAM45A	7.613934
FAM45B	3.97657
FAM46A	7.271286
FAM46B	4.79604
FAM46C	4.028528
FAM46D	2.305874
FAM47A	3.11869
FAM47B	4.311508
FAM47C	4.007834
FAM47E	2.882492
FAM48A	8.080098
FAM48B2	3.239408
FAM48B2	3.239408
FAM49A	2.884268
FAM49B	8.622198
FAM50A	8.035172
FAM50B	6.187658
FAM53A	5.080946
FAM53B	6.853406
FAM53C	7.058278
FAM54A	7.255546
FAM54B	6.803534
FAM54B	5.018452
FAM55A	2.495608
FAM55B	2.361984
FAM55C	5.156172
FAM55D	2.810728
FAM57A	7.132668
FAM57B	3.556642
FAM57B	4.96721
FAM58A	6.750354
FAM58A	7.55989
FAM58B	4.470416
FAM59A	5.13514
FAM59B	4.934692
FAM5B	2.817946
FAM5C	2.284576
FAM60A	7.18296

FAM63A	5.2326
FAM63B	5.510466
FAM64A	7.611886
FAM65A	5.849892
FAM65B	3.475254
FAM65C	3.580952
FAM69A	2.971918
FAM69B	5.079974
FAM69C	2.775498
FAM70A	3.196152
FAM70B	3.951586
FAM71A	3.594542
FAM71A	3.46198
FAM71B	2.84199
FAM71C	2.515948
FAM71D	2.630308
FAM71E2	3.632648
FAM71E2	5.92018
FAM71F1	3.316702
FAM71F2	3.843986
FAM72D	7.481884
FAM72D	7.455394
FAM72D	7.08113
FAM72D	7.275672
FAM73A	6.070256
FAM73B	6.125882
FAM74A3	3.178072
FAM74A3	2.835448
FAM74A3	2.83851
FAM74A3	2.762364
FAM74A3	2.749626
FAM74A3	3.006996
FAM74A3	2.582252
FAM75A7	3.595982
FAM75A7	3.51109
FAM75A7	3.69291
FAM75A7	3.598058
FAM75A7	3.59811
FAM75A7	3.51316
FAM75A7	3.647152
FAM75C1	2.672388
FAM75C1	3.51996
FAM75C2	3.57168

FAM75D1	2.702144
FAM75D4	2.75181
FAM75D5	2.57709
FAM76A	5.8041
FAM76B	5.580166
FAM78A	4.576322
FAM78B	3.82416
FAM81A	3.620274
FAM81B	2.98582
FAM82A1	2.95998
FAM82A2	6.241894
FAM82B	6.478546
FAM83A	7.917484
FAM83B	7.729266
FAM83C	3.604522
FAM83D	8.074394
FAM83E	5.956678
FAM83F	4.363664
FAM83G	5.197788
FAM83H	7.406106
FAM84A	4.764438
FAM84B	7.865034
FAM86A	6.502426
FAM86B1	7.136176
FAM86B1	7.122978
FAM86B1	6.17379
FAM86B1	7.639458
FAM86C	6.79104
FAM86DP	6.201408
FAM87A	2.985892
FAM87A	3.270288
FAM89A	5.770898
FAM89B	6.66553
FAM8A1	6.272294
FAM90A1	3.983638
FAM90A10	3.998964
FAM90A13	4.224816
FAM90A13	4.010336
FAM90A13	4.010336
FAM90A18	4.224816
FAM90A20	3.583822
FAM90A25P	4.172988
FAM90A7	4.152318

FAM90A7	4.169364
FAM90A8	4.010336
FAM90A8	4.010336
FAM91A1	9.104168
FAM92A1	5.363456
FAM92A2	3.704732
FAM92A3	2.225946
FAM92B	4.674294
FAM95B1	3.180598
FAM95B1	3.391648
FAM95B1	3.391648
FAM96A	8.145322
FAM96B	8.748458
FAM98A	8.047572
FAM98B	5.327706
FAM98C	6.771358
FAM99A	3.065028
FAM99A	2.642248
FAM9A	2.471304
FAM9B	2.593186
FAM9C	2.281606
FAN1	4.90409
FANCA	6.705004
FANCB	3.471642
FANCC	7.735052
FANCD2	8.123756
FANCE	5.959556
FANCF	5.165658
FANCG	6.471166
FANCI	7.106536
FANCL	6.96658
FANCM	4.171478
FANK1	5.355496
FAP	2.412048
FAR1	6.69686
FAR2	3.103604
FARP1	7.448104
FARP2	4.556008
FARS2	5.80409
FARSA	9.718386
FARSB	8.721298
FAS	6.203038
FASLG	2.989396

FASN	7.960618
FASTK	7.99632
FASTKD1	6.420158
FASTKD2	6.36997
FASTKD3	5.903358
FASTKD5	7.015704
FAT1	7.095314
FAT2	3.083062
FAT3	2.933344
FAT4	2.597816
FATE1	3.460822
FAU	9.456294
FBF1	5.441806
FBL	8.759466
FBLIM1	5.785262
FBLL1	4.117954
FBLN1	7.405636
FBLN2	5.292586
FBLN5	4.54942
FBLN7	4.13904
FBN1	4.87121
FBN2	2.791776
FBN3	4.650674
FBP1	8.977786
FBP2	3.2872
FBRS	6.273564
FBRSL1	6.82382
FBXL12	6.217234
FBXL13	4.667584
FBXL14	5.633538
FBXL15	4.18792
FBXL16	4.13207
FBXL16	5.40558
FBXL17	5.44176
FBXL18	6.605074
FBXL19	6.410124
FBXL2	5.006124
FBXL20	7.162254
FBXL21	2.325176
FBXL22	4.153336
FBXL3	7.971954
FBXL4	5.919062
FBXL5	8.157366

FBXL6	5.66268
FBXL7	4.317262
FBXL8	5.732564
FBXO10	8.122998
FBXO11	7.41385
FBXO15	2.627796
FBXO16	4.569982
FBXO17	6.529768
FBXO17	5.404024
FBXO18	7.213372
FBXO2	7.717958
FBXO21	7.79985
FBXO22	6.815072
FBXO24	3.952614
FBXO25	6.48315
FBXO25	6.39373
FBXO27	5.990214
FBXO28	8.557338
FBXO3	6.947918
FBXO30	7.001176
FBXO31	6.034746
FBXO32	7.31671
FBXO33	4.486416
FBXO34	8.179294
FBXO36	4.296042
FBXO38	7.292738
FBXO39	3.730986
FBXO4	6.509464
FBXO40	2.776902
FBXO41	4.490662
FBXO42	6.811006
FBXO43	3.545544
FBXO44	5.769384
FBXO45	7.81407
FBXO46	6.04387
FBXO47	2.377368
FBXO48	3.159366
FBXO5	7.238808
FBXO6	5.137482
FBXO7	9.189416
FBXO8	6.49686
FBXO9	7.073908
FBXO9	4.047184

FBXO9	3.451646
FBXO9	5.552196
FBXW10	5.653382
FBXW11	7.648208
FBXW12	2.773886
FBXW2	8.870348
FBXW4	7.301856
FBXW4P1	5.66906
FBXW5	7.262628
FBXW7	5.008154
FBXW8	6.705042
FBXW9	5.845512
FCAMR	3.80158
FCAR	3.554544
FCER1A	2.560228
FCER1G	3.235756
FCER2	3.515992
FCF1	6.938886
FCGBP	4.306846
FCGR1A	2.097272
FCGR1A	2.32707
FCGR1B	2.401434
FCGR2A	2.27458
FCGR2B	2.779594
FCGR2C	3.852084
FCGR3A	2.538706
FCGR3A	3.971656
FCGRT	4.544464
FCHO1	4.277254
FCHO2	5.595864
FCHSD1	4.861602
FCHSD2	6.089212
FCN1	4.538936
FCN2	5.100902
FCN3	3.857476
FCRL1	3.368506
FCRL2	2.502366
FCRL3	2.744382
FCRL4	3.284106
FCRL5	3.021054
FCRL6	3.908692
FCRLA	3.075326
FCRLB	5.088932

FDFT1	9.71782
FDPS	8.384008
FDPS	3.991612
FDPSL2A	6.153646
FDPSL2A	6.2964
FDX1	5.969668
FDX1L	6.547638
FDXACB1	3.91146
FDXR	5.41242
FECH	5.561848
FEM1A	5.970778
FEM1B	7.416456
FEM1C	6.766782
FEN1	8.513838
FER	4.130502
FER1L4	5.225978
FER1L4	6.122534
FER1L5	3.521088
FER1L5	3.792384
FER1L6	3.037166
FER1L6	2.845874
FERD3L	4.269808
FERMT1	6.818442
FERMT2	8.167038
FERMT3	4.539044
FES	3.82289
FETUB	2.810982
FEV	4.692578
FEZ1	3.04106
FEZ2	6.472346
FEZF1	3.615326
FEZF2	4.093944
FFAR1	4.724202
FFAR2	3.233362
FFAR3	3.259478
FFAR3	3.252238
FGA	2.551304
FGB	2.737016
FGD1	4.14022
FGD2	4.013394
FGD3	5.906918
FGD4	4.584936
FGD5	3.094222

FGD6	6.50912
FGF1	2.87291
FGF10	2.888274
FGF11	4.220316
FGF12	3.035984
FGF13	3.417656
FGF14	2.575794
FGF16	2.556372
FGF17	4.178278
FGF18	4.524796
FGF19	3.92991
FGF2	8.753082
FGF20	3.435834
FGF21	3.84442
FGF22	5.288508
FGF23	4.178808
FGF3	4.669784
FGF4	5.896874
FGF5	3.29068
FGF6	4.842284
FGF7	2.636828
FGF8	4.524354
FGF9	2.555434
FGFBP1	10.236156
FGFBP2	2.792768
FGFBP3	4.199742
FGFR1	5.305742
FGFR1OP	6.385802
FGFR1OP2	6.970816
FGFR2	3.128026
FGFR3	6.592336
FGFR4	4.578196
FGFRL1	5.523452
FGG	2.462572
FGGY	6.638554
FGL1	2.101588
FGL2	2.919754
FGR	5.342256
FH	8.329804
FHAD1	3.84187
FHDC1	5.34379
FHIT	3.072962
FHL1	5.0417

FHL2	6.89443
FHL3	5.67361
FHL5	3.177024
FHOD1	5.350682
FHOD3	3.697242
FIBCD1	6.160908
FIBIN	2.582244
FIBP	8.712776
FICD	5.661354
FIG4	6.451328
FIGF	2.603986
FIGLA	3.219844
FIGN	4.20703
FIGNL1	6.171908
FIGNL2	3.287848
FILIP1	2.75823
FILIP1L	2.69851
FIP1L1	7.81616
FIS1	6.583994
FITM1	4.845272
FITM2	5.40067
FIZ1	6.043476
FJX1	5.759836
FKBP10	7.526222
FKBP11	5.269046
FKBP14	6.79139
FKBP15	7.906342
FKBP1A	6.063086
FKBP1A	9.038046
FKBP1B	5.103612
FKBP2	7.856576
FKBP3	7.561838
FKBP4	9.379258
FKBP5	7.09402
FKBP6	2.902636
FKBP6	2.73415
FKBP7	3.18168
FKBP8	7.882634
FKBP9	7.258372
FKBP9L	4.492904
FKBPL	5.343536
FKBPL	5.343536
FKRP	5.59503

FKSG2	2.133314
FKSG83	2.9107
FKTN	5.343092
FLAD1	6.43588
FLCN	6.278994
FLG	3.361074
FLG2	2.880686
FLI1	2.64414
FLII	8.972204
FLJ00290	2.715304
FLJ10661	4.215654
FLJ10661	3.396462
FLJ11235	4.94917
FLJ11292	3.03362
FLJ11710	4.37101
FLJ13197	3.735722
FLJ13224	3.803712
FLJ13744	3.34075
FLJ14107	3.053906
FLJ16124	3.06221
FLJ16126	3.208512
FLJ16171	3.533334
FLJ16734	3.64262
FLJ20464	4.314474
FLJ20712	2.77092
FLJ22184	6.253994
FLJ23865	3.685922
FLJ25328	3.218444
FLJ25694	4.104744
FLJ25715	3.900276
FLJ25758	3.247426
FLJ25758	3.233212
FLJ26850	3.3866
FLJ27243	4.40109
FLJ27255	3.12458
FLJ30064	3.20102
FLJ30403	3.295414
FLJ30403	3.171518
FLJ30430	3.322384
FLJ30679	3.610346
FLJ30901	4.776344
FLJ31713	4.151042
FLJ31958	3.89188

FLJ32063	3.17358
FLJ32154	4.499228
FLJ32742	2.665012
FLJ32756	3.098638
FLJ32790	3.265488
FLJ32955	2.269452
FLJ33360	3.628484
FLJ33534	4.151106
FLJ33544	3.119666
FLJ33996	3.347216
FLJ34503	2.81706
FLJ34521	3.446528
FLJ34690	2.427042
FLJ35220	5.839564
FLJ35409	2.91091
FLJ35424	3.894726
FLJ35776	6.94834
FLJ35816	2.654784
FLJ35934	5.271594
FLJ35934	4.184822
FLJ36000	3.31461
FLJ36031	7.387278
FLJ36116	4.078764
FLJ36840	4.834656
FLJ37035	3.762394
FLJ37201	2.746638
FLJ37396	4.231574
FLJ37453	3.825404
FLJ37543	3.333532
FLJ37786	4.833238
FLJ37786	4.956962
FLJ38109	4.96921
FLJ38379	2.675326
FLJ38576	5.525584
FLJ38668	5.501552
FLJ38723	3.308608
FLJ38773	4.167894
FLJ38894	4.011908
FLJ39061	2.841168
FLJ39061	2.752132
FLJ39632	5.457104
FLJ39632	6.035816
FLJ39632	5.965982

FLJ39639	3.524384
FLJ39653	5.040962
FLJ40194	3.286574
FLJ40288	2.746662
FLJ40292	5.13571
FLJ40448	4.830504
FLJ40453	2.385984
FLJ40504	5.417058
FLJ40536	3.694944
FLJ40852	3.473104
FLJ41170	3.851952
FLJ41309	2.304442
FLJ41327	4.56954
FLJ41423	3.192714
FLJ41484	3.33909
FLJ41649	2.842606
FLJ41733	3.545196
FLJ42102	3.464196
FLJ42220	3.196972
FLJ42280	2.460086
FLJ42289	2.927572
FLJ42291	2.979502
FLJ42393	4.62012
FLJ42418	2.884882
FLJ42842	2.874594
FLJ42875	6.164366
FLJ42875	4.71679
FLJ43315	3.494634
FLJ43390	2.512002
FLJ43390	3.696122
FLJ43681	6.969102
FLJ43763	2.4921
FLJ43826	2.98459
FLJ43860	5.010566
FLJ43860	4.06415
FLJ43879	2.982244
FLJ44006	2.649502
FLJ44124	4.879874
FLJ44313	4.743426
FLJ44385	3.833342
FLJ44606	4.643656
FLJ44635	4.49499
FLJ44674	3.725978

FLJ44790	2.592422
FLJ44838	2.279882
FLJ44874	3.571508
FLJ44881	2.716164
FLJ44896	4.005652
FLJ45079	3.005902
FLJ45139	3.366648
FLJ45244	4.170872
FLJ45248	4.094786
FLJ45256	2.69377
FLJ45340	7.644218
FLJ45340	8.638286
FLJ45340	5.75462
FLJ45340	5.335492
FLJ45684	4.566572
FLJ45721	2.64798
FLJ45743	2.957448
FLJ45825	2.734138
FLJ45831	3.204784
FLJ45832	2.348846
FLJ45949	2.768948
FLJ45950	2.930504
FLJ45964	2.817112
FLJ45974	2.363382
FLJ45983	3.921366
FLJ46010	2.539218
FLJ46020	3.236
FLJ46111	4.122834
FLJ46120	4.046122
FLJ46257	2.874858
FLJ46300	4.301732
FLJ46358	4.404514
FLJ46361	4.289542
FLJ46363	4.532718
FLJ46365	2.875274
FLJ46836	3.758218
FLJ90680	3.345062
FLJ90757	5.473654
FLNA	8.650436
FLNB	9.917154
FLNC	4.30365
FLOT1	7.4025
FLOT1	7.4025

FLOT1	7.4025
FLOT2	8.036662
FLRT1	4.361532
FLRT2	4.164898
FLRT3	3.475422
FLT1	2.848014
FLT3	3.046518
FLT3LG	6.109744
FLT4	4.433542
FLVCR1	6.227098
FLVCR2	4.60465
FLYWCH1	5.897628
FLYWCH2	6.978394
FMN1	4.968336
FMN1	3.96736
FMN2	2.789018
FMNL1	5.678884
FMNL2	6.323932
FMNL3	4.198612
FMO1	2.250846
FMO2	2.586998
FMO3	2.45985
FMO4	5.033428
FMO5	3.11908
FMO6P	2.717808
FMO9P	2.664678
FMOD	2.481338
FMR1	7.443714
FMR1NB	4.33179
FN1	2.958172
FN1	6.466982
FN3K	4.499288
FN3KRP	8.15923
FNBP1	6.28956
FNBP1L	6.094786
FNBP4	6.436124
FNDC1	3.496206
FNDC3A	7.341736
FNDC3B	8.770198
FNDC4	3.632576
FNDC5	3.862282
FNDC7	2.806876
FNDC8	3.186618

FNIP1	6.586664
FNIP2	4.071482
FNTA	7.6611
FNTB	6.371626
FOLH1	3.890698
FOLH1	2.407484
FOLH1B	2.289272
FOLR1	3.938786
FOLR2	3.530042
FOLR3	4.133012
FOLR4	4.584966
FOS	6.628788
FOSB	5.032102
FOSL1	6.7327
FOSL2	7.224438
FOXA1	6.822232
FOXA2	4.851736
FOXA3	4.130098
FOXB1	4.902598
FOXB2	6.137464
FOXC1	5.982388
FOXC2	5.973436
FOXD1	5.574724
FOXD2	4.330714
FOXD3	4.623336
FOXD4	4.13862
FOXD4L1	4.127824
FOXD4L2	4.560876
FOXD4L2	5.046676
FOXD4L2	4.560876
FOXD4L3	4.309086
FOXD4L6	3.969324
FOXE1	4.554156
FOXE3	6.586558
FOXF1	4.515094
FOXF2	5.731912
FOXG1	4.218434
FOXH1	4.400938
FOXI1	6.59505
FOXI2	5.384636
FOXJ1	4.240706
FOXJ2	6.414698
FOXJ3	7.918318

FOXK1	7.636446
FOXK2	7.768248
FOXK2	2.990948
FOXL1	5.033704
FOXL2	4.388416
FOXM1	9.167506
FOXN1	3.128128
FOXN2	6.455182
FOXN3	5.242468
FOXN4	4.26031
FOXO1	7.236826
FOXO3	5.200348
FOXO3B	9.396888
FOXO4	5.27035
FOXP1	5.850962
FOXP2	2.482014
FOXP3	4.041894
FOXP4	6.73891
FOXQ1	6.779386
FOXR1	2.674922
FOXR2	2.45046
FOXRED1	7.279422
FOXRED2	5.73719
FOXS1	4.463056
FPGS	7.599794
FPGT	6.242394
FPR1	3.051818
FPR2	2.717468
FPR3	2.899802
FRA10AC1	3.735688
FRAS1	4.760706
FRAT1	3.643804
FRAT2	5.57467
FREM1	3.009964
FREM2	2.651672
FREM3	2.403236
FRG1	5.37929
FRG1	8.233608
FRG1B	5.513104
FRG2	2.537202
FRG2B	2.719376
FRG2C	2.593796
FRK	6.969818

FRMD1	3.633188
FRMD3	6.725588
FRMD4A	3.983596
FRMD4B	6.916656
FRMD5	4.756314
FRMD6	6.440832
FRMD7	3.243278
FRMD8	5.54354
FRMPD1	3.103402
FRMPD2	2.87676
FRMPD2	2.87676
FRMPD2	2.834474
FRMPD3	2.66315
FRMPD4	2.665076
FRRS1	6.04981
FRS2	6.22659
FRS3	5.122038
FRY	5.825344
FRYL	6.917402
FRYL	6.65082
FRZB	2.534914
FSCB	2.893872
FSCN1	8.220012
FSCN2	4.879624
FSCN3	3.241584
FSD1	4.14921
FSD1L	2.760822
FSD2	2.852496
FSHB	3.006626
FSHR	2.869052
FSIP1	2.436868
FSIP2	2.327586
FSIP2	2.379678
FSIP2	2.16822
FST	4.416592
FSTL1	8.984378
FSTL3	5.966236
FSTL4	3.569426
FSTL5	2.388384
FTCD	5.281586
FTH1	9.310022
FTH1	12.46306
FTH1P3	5.758952

FTH1P5	4.117788
FTHL17	4.182022
FTL	10.11898
FTMT	2.844014
FTO	8.117552
FTSJ1	6.669894
FTSJ2	8.460472
FTSJ3	7.759412
FTSJD1	6.329048
FTSJD2	8.170686
FUBP1	9.583828
FUBP3	8.004028
FUCA1	6.303212
FUCA2	8.555456
FUK	4.54657
FUNDC1	6.587578
FUNDC2	5.27774
FUNDC2P2	3.212952
FURIN	5.851394
FUS	10.060974
FUT1	5.427738
FUT10	5.227868
FUT11	4.288286
FUT2	3.237384
FUT3	6.175832
FUT4	5.37296
FUT5	4.89117
FUT6	4.024124
FUT7	4.00883
FUT7	3.257194
FUT8	6.483878
FUT9	4.376966
FUZ	5.201312
FXC1	6.592014
FXN	6.885722
FXR1	9.440262
FXR2	7.203346
FXYD1	4.48963
FXYD2	3.169988
FXYD2	5.352626
FXYD3	10.80764
FXYD4	4.094884
FXYD5	8.275196

FXYD6	4.293572
FXYD7	5.398176
FYB	4.480298
FYCO1	6.003112
FYN	5.626938
FYTTD1	8.54408
FZD1	5.641698
FZD10	4.105938
FZD2	5.808372
FZD3	3.96511
FZD4	6.090804
FZD5	5.584946
FZD6	7.932934
FZD7	6.171726
FZD8	6.157252
FZD9	5.744294
FZR1	6.608846
G0S2	4.754432
G2E3	4.80614
G3BP1	10.87412
G3BP2	8.167684
G6PC	2.955186
G6PC2	2.467922
G6PC3	7.705538
G6PD	10.05773
GAA	5.646214
GAB1	6.5887
GAB1	4.858224
GAB2	4.794304
GAB3	3.754574
GAB4	3.870712
GABARAP	9.164796
GABARAPL1	6.498408
GABARAPL2	9.262008
GABARAPL3	3.124534
GABBR1	4.89596
GABBR1	4.91596
GABBR1	4.983576
GABBR2	3.82421
GABPA	5.591082
GABPA	6.86078
GABPB1	7.317072
GABPB2	5.372998

GABRA1	2.854046
GABRA2	2.722186
GABRA3	2.563814
GABRA4	3.035272
GABRA5	4.183264
GABRA6	2.934454
GABRB1	2.455982
GABRB2	3.082886
GABRB3	4.098976
GABRD	4.468262
GABRE	7.751032
GABRG1	2.404378
GABRG2	2.626554
GABRG3	2.52146
GABRP	7.837542
GABRQ	3.62033
GABRR1	3.311152
GABRR2	3.977318
GABRR3	2.301772
GAD1	4.157382
GAD2	3.046438
GADD45A	8.264594
GADD45B	7.253688
GADD45G	4.637984
GADD45GIP1	7.544106
GADL1	2.69973
GAFA1	2.202444
GAFA2	3.378912
GAFA3	2.524496
GAGE12B	2.936156
GAGE12B	6.402862
GAGE12B	4.544854
GAGE12G	3.157416
GAGE12G	3.157416
GAGE12I	2.936156
GAGE12J	2.858314
GAGE12J	2.576608
GAGE12J	2.397034
GAK	6.434568
GAL	7.541952
GAL3ST1	4.019628
GAL3ST2	4.239354
GAL3ST3	4.77972

GAL3ST4	4.835078
GALC	6.194552
GALE	6.993666
GALK1	5.83243
GALK2	6.786094
GALM	2.943706
GALNS	6.67386
GALNT1	6.414726
GALNT10	7.220722
GALNT11	7.686294
GALNT12	3.684034
GALNT13	2.602904
GALNT14	3.553202
GALNT2	8.053088
GALNT3	7.255598
GALNT4	6.060742
GALNT5	2.201446
GALNT6	6.097344
GALNT7	8.17186
GALNT8	2.882986
GALNT9	5.227058
GALNTL1	3.732214
GALNTL2	3.254676
GALNTL4	3.030792
GALNTL5	2.312194
GALNTL6	2.941124
GALP	3.598368
GALR1	3.786416
GALR2	4.684378
GALR3	5.795416
GALT	5.646996
GAMT	5.427386
GAN	7.177952
GANAB	10.466492
GAP43	3.502036
GAPDH	12.93336
GAPDHS	3.369762
GAPT	2.491642
GAPVD1	8.394802
GAR1	6.77994
GARNL3	4.13465
GARS	10.110246
GART	9.523566

GAS1	3.959928
GAS2	2.640862
GAS2L1	5.654362
GAS2L2	4.209932
GAS2L3	7.879598
GAS6	4.533616
GAS7	4.734358
GAS8	6.922574
GAST	5.030308
GATA1	4.252294
GATA2	6.901628
GATA3	7.790662
GATA4	4.326756
GATA5	5.790126
GATA6	7.562466
GATAD1	6.21622
GATAD2A	8.129306
GATAD2B	7.062552
GATC	8.437214
GATM	7.707256
GATS	5.47814
GATSL1	6.181308
GATSL1	6.181308
GATSL3	5.09776
GBA	7.378248
GBA2	5.928052
GBA3	2.669654
GBAP1	5.468272
GBAS	8.574284
GBE1	7.032924
GBF1	7.560436
GBGT1	3.961514
GBP1	7.347058
GBP2	3.65918
GBP3	6.537388
GBP4	5.988664
GBP5	3.205592
GBP6	2.566494
GBP7	4.242394
GBX1	3.516872
GBX2	5.82072
GC	2.32818
GCA	6.134504

GCAT	5.649818
GCC1	6.507434
GCC2	5.948732
GCDH	6.285144
GCET2	3.086854
GCFC1	6.54976
GCG	2.380176
GCGR	4.774764
GCH1	5.763538
GCHFR	5.204854
GCK	4.11743
GCKR	3.319736
GCLC	8.303532
GCLM	6.434768
GCM1	2.560726
GCM2	2.916222
GCN1L1	8.357996
GCNT1	3.140656
GCNT2	5.098402
GCNT3	4.963588
GCNT4	2.959068
GCNT7	3.355152
GCOM1	5.562756
GCRG224	2.670728
GCSH	7.234704
GCSH	7.197454
GCSH	7.915492
GDA	6.101748
GDAP1	6.109934
GDAP1L1	3.38737
GDAP2	6.391636
GDE1	8.223758
GDEP	2.449932
GDF1	4.47222
GDF10	4.923626
GDF11	4.521386
GDF15	6.54527
GDF2	3.496846
GDF3	2.926984
GDF5	3.685278
GDF5	2.9792
GDF6	3.672074
GDF7	4.877678

GDF9	3.009932
GDI1	8.779514
GDI2	10.3095
GDNF	2.837306
GDPD1	3.516688
GDPD2	3.037442
GDPD3	7.235714
GDPD4	2.313228
GDPD5	5.757926
GEM	3.936194
GEMIN4	7.190222
GEMIN5	6.810326
GEMIN6	5.677168
GEMIN7	6.478628
GEMIN8	5.130608
GEMIN8P4	3.831722
GEN1	6.503456
GET4	7.555254
GFAP	4.378554
GFER	6.086778
GFI1	4.19353
GFI1B	4.375524
GFM1	7.361738
GFM2	6.297042
GFOD1	6.894434
GFOD2	4.756504
GFPT1	9.089342
GFPT2	7.427644
GFRA1	3.491828
GFRA2	2.82816
GFRA3	3.081106
GFRA4	5.986224
GFRAL	2.374264
GGA1	6.402278
GGA2	8.135468
GGA3	5.996238
GGCT	8.103006
GGCX	7.302326
GGH	8.027028
GGN	4.890588
GGNBP1	4.059638
GGNBP2	8.50305
GGPS1	6.733714

GGT1	5.551434
GGT3P	5.387284
GGT3P	5.296024
GGT5	4.879024
GGT6	4.891496
GGT7	3.66519
GGTA1	2.475084
GGTLC1	2.732306
GGTLC2	4.79254
GH1	3.975122
GH2	2.892034
GHDC	5.340508
GHITM	8.701154
GHR	2.377786
GHRH	3.643752
GHRHR	3.995718
GHRL	4.792408
GHSR	3.952178
GIF	2.934476
GIGYF1	5.475674
GIGYF2	7.69381
GIMAP1	2.751374
GIMAP2	3.226308
GIMAP4	2.47583
GIMAP5	2.79226
GIMAP6	3.085534
GIMAP7	2.424308
GIMAP8	2.91309
GIN1	3.036018
GINS1	8.314248
GINS2	8.206498
GINS3	7.20738
GINS4	7.283682
GIP	3.010176
GIPC1	9.208914
GIPC2	3.008046
GIPC3	4.882002
GIPR	4.679994
GIT1	7.14293
GIT2	6.934648
GJA1	3.965572
GJA10	2.447778
GJA3	4.912244

GJA4	5.094906
GJA5	5.06473
GJA8	3.709958
GJA9	2.697818
GJB1	3.22629
GJB2	5.63511
GJB3	4.601922
GJB4	4.674428
GJB5	6.711302
GJB6	2.929332
GJB7	2.735442
GJC1	8.012066
GJC2	6.602002
GJC3	3.229888
GJD2	5.406406
GJD3	5.570334
GJD4	3.652706
GJD4	4.378776
GK	5.166582
GK	6.771642
GK2	2.210402
GK3P	4.821732
GK5	6.988312
GKAP1	3.021444
GKN1	2.268452
GKN2	2.857382
GLA	5.982616
GLB1	7.810238
GLB1L	4.676794
GLB1L2	7.59462
GLB1L3	2.968948
GLCCI1	6.699266
GLCE	6.551622
GLDC	4.341766
GLDN	2.833534
GLE1	7.94379
GLG1	9.101864
GLI1	3.168104
GLI2	4.262566
GLI3	4.390222
GLI4	5.731226
GLIPR1	5.265918
GLIPR1L1	2.495512

GLIPR1L2	2.284326
GLIPR2	5.646106
GLIS1	4.562564
GLIS2	6.057574
GLIS3	3.205696
GLMN	4.312524
GLO1	8.989952
GLOD4	9.440242
GLOD5	3.347554
GLP1R	4.570786
GLP2R	4.508094
GLRA1	2.734364
GLRA2	2.811452
GLRA3	2.476434
GLRA4	3.755014
GLRB	3.299582
GLRX	7.003194
GLRX2	6.187526
GLRX3	9.449552
GLRX5	8.385758
GLRX5	8.527112
GLS	7.58186
GLS2	3.965236
GLT1D1	2.886082
GLT25D1	9.02243
GLT25D2	3.506324
GLT6D1	2.631558
GLT8D1	6.902934
GLT8D2	3.405488
GLTP	6.893598
GLTPD1	6.324374
GLTPD1	4.870428
GLTPD2	4.195876
GLTSCR1	4.640646
GLTSCR2	7.880178
GLTSCR2	7.98548
GLUD1	3.151812
GLUD2	2.410022
GLUL	8.319816
GLUL	10.39718
GLYAT	2.500166
GLYATL1	3.1016
GLYATL2	2.983042

GLYCTK	4.823982
GLYR1	7.658898
GM2A	7.095682
GMCL1	6.924178
GMCL1L	2.014376
GMDS	6.454112
GMEB1	6.695896
GMEB2	6.099242
GMFB	6.673586
GMFG	3.708804
GMIP	6.039248
GML	2.831366
GMNN	7.477742
GMPPA	6.961038
GMPPB	6.378086
GMPR	3.619896
GMPR2	7.676058
GMPS	9.879038
GNA11	6.736674
GNA12	7.422794
GNA13	7.069574
GNA14	3.260596
GNA15	4.070408
GNAI1	5.615352
GNAI2	7.849132
GNAI3	7.360084
GNAL	5.173038
GNAO1	4.203882
GNAQ	8.56495
GNAS	8.003416
GNAT1	3.154326
GNAT2	3.272016
GNAZ	4.566392
GNB1	10.47814
GNB1L	6.007416
GNB2	7.856926
GNB2L1	11.8644
GNB3	3.691806
GNB4	6.04894
GNB5	6.024596
GNE	8.629348
GNG10	8.233302
GNG11	2.719508

GNG12	8.490376
GNG13	4.997264
GNG2	2.840998
GNG3	3.101142
GNG4	6.498066
GNG5	7.85136
GNG5	9.62257
GNG7	4.744466
GNG8	4.559194
GNGT1	2.212526
GNGT2	2.681504
GNL1	7.213732
GNL1	7.256212
GNL1	7.052152
GNL2	8.417436
GNL3	8.030282
GNL3L	6.609268
GNLY	3.628828
GNMT	3.490682
GNN	2.25641
GNPAT	7.910168
GNPDA1	8.286398
GNPDA2	4.159194
GNPNAT1	8.754636
GNPTAB	5.249332
GNPTG	7.383892
GNRH1	3.275594
GNRH2	4.28829
GNRHR	2.408396
GNRHR2	4.201502
GNS	8.42538
GOLGA1	7.02335
GOLGA2	8.301352
GOLGA2B	3.914704
GOLGA2P2Y	4.348196
GOLGA2P2Y	4.348196
GOLGA3	6.955898
GOLGA4	5.58248
GOLGA4	4.218896
GOLGA5	6.655412
GOLGA6A	4.068586
GOLGA6A	4.558416
GOLGA6A	3.794042

GOLGA6A	3.949124
GOLGA6B	3.678298
GOLGA6B	3.795102
GOLGA6B	4.72838
GOLGA6L1	3.157636
GOLGA6L1	2.838352
GOLGA6L1	2.861644
GOLGA6L1	2.898662
GOLGA6L10	5.59516
GOLGA6L10	5.715734
GOLGA6L10	5.654752
GOLGA6L2	4.639618
GOLGA6L5	5.326558
GOLGA6L5	5.25774
GOLGA6L5	5.538578
GOLGA6L9	5.470644
GOLGA6L9	5.470644
GOLGA6L9	5.66639
GOLGA7	8.785198
GOLGA7B	3.923936
GOLGA8B	5.75325
GOLGA8B	5.7654
GOLGA8C	4.325066
GOLGA8DP	3.892632
GOLGA8DP	4.372364
GOLGA8DP	4.52559
GOLGA8E	4.375034
GOLGA8F	3.967344
GOLGA8G	4.162372
GOLGA8G	4.162372
GOLGA8H	4.529814
GOLGA8IP	3.549954
GOLGA8IP	3.684912
GOLGA8IP	3.89779
GOLGA8IP	4.32364
GOLGA8IP	3.430534
GOLGA8IP	4.61466
GOLGA8IP	3.694326
GOLGA8IP	3.696052
GOLGA8IP	4.61466
GOLGB1	6.649844
GOLIM4	6.386804
GOLM1	8.178228

GOLPH3	9.198736
GOLPH3L	7.29849
GOLT1A	4.517814
GOLT1B	8.379988
GON4L	6.888108
GOPC	7.991896
GORAB	5.907168
GORASP1	6.000238
GORASP2	8.379932
GOSR1	7.90668
GOSR2	6.902368
GOT1	8.499898
GOT1L1	4.226464
GOT2	9.059728
GP1BA	3.401236
GP1BB	5.227024
GP1BB	5.346422
GP1BB	6.900496
GP2	3.388778
GP5	4.387292
GP6	4.732818
GP9	4.938482
GPA33	3.539142
GPAA1	8.400156
GPAM	2.127054
GPAM	4.149552
GPAM	2.206184
GPAM	2.275876
GPAT2	5.16894
GPATCH1	6.130146
GPATCH2	6.187856
GPATCH3	5.219148
GPATCH4	4.109564
GPATCH4	7.2854
GPATCH8	7.152574
GPBAR1	3.819278
GPBP1	7.23138
GPBP1L1	8.598432
GPC1	6.36147
GPC2	3.091732
GPC2	3.951218
GPC3	3.512368
GPC4	2.632362

GPC5	2.982464
GPC6	3.049676
GPCPD1	5.55143
GPCRLTM7	2.63091
GPD1	3.84757
GPD1L	5.724516
GPD2	7.865866
GPER	5.45289
GPHA2	3.87978
GPHB5	4.32692
GPHN	6.251122
GPI	10.238474
GPIHBP1	4.106932
GPKOW	6.98564
GPLD1	3.555822
GPM6A	2.931938
GPM6B	2.648504
GPN1	8.203132
GPN2	6.37748
GPN3	6.269812
GPNMB	5.371814
GPR1	6.406608
GPR101	3.569882
GPR107	8.311314
GPR108	8.153176
GPR109A	6.339078
GPR109B	4.991476
GPR110	8.854464
GPR111	3.215684
GPR112	2.408888
GPR113	4.643404
GPR114	3.503918
GPR115	3.775298
GPR116	3.028094
GPR116	4.105142
GPR119	3.07162
GPR12	3.907964
GPR120	3.784584
GPR123	4.641144
GPR124	4.324268
GPR125	5.963198
GPR125	4.99767
GPR126	6.558876

GPR128	2.168048
GPR132	3.181342
GPR133	3.466842
GPR135	4.685944
GPR137	5.992354
GPR137B	7.375144
GPR137C	4.266596
GPR139	5.0279
GPR141	2.897936
GPR142	4.831054
GPR143	3.319192
GPR144	5.10825
GPR146	3.51954
GPR148	4.103664
GPR149	3.667124
GPR15	2.695908
GPR15	3.71493
GPR150	5.925142
GPR151	2.834548
GPR152	4.186876
GPR153	5.547586
GPR155	3.287082
GPR156	3.16621
GPR157	5.068628
GPR158	4.565044
GPR160	5.459102
GPR161	5.108782
GPR162	3.24227
GPR17	3.30269
GPR171	2.795252
GPR172A	8.369848
GPR172B	4.692882
GPR173	3.34762
GPR174	2.336144
GPR176	4.05662
GPR179	3.542316
GPR18	2.472312
GPR180	7.59638
GPR182	3.98993
GPR183	2.57245
GPR19	7.409824
GPR20	4.185522
GPR21	4.436384

GPR22	2.56123
GPR25	5.306996
GPR26	3.054934
GPR27	6.147932
GPR3	5.627698
GPR31	3.744282
GPR32	1.89996
GPR32	4.172898
GPR34	2.137966
GPR35	3.739264
GPR37	3.720706
GPR37L1	5.72916
GPR39	6.693542
GPR4	4.085968
GPR44	5.58718
GPR45	3.111166
GPR50	3.477896
GPR52	3.985356
GPR55	4.026024
GPR56	7.738072
GPR6	4.925374
GPR61	4.410386
GPR62	5.576048
GPR63	2.478596
GPR64	2.431246
GPR65	2.307936
GPR68	3.815476
GPR75	5.509734
GPR77	3.550968
GPR78	4.40612
GPR81	6.66566
GPR82	2.174482
GPR83	3.165972
GPR84	3.01531
GPR85	2.462882
GPR87	6.370308
GPR88	5.02763
GPR89A	7.030756
GPR89B	7.086132
GPR89B	7.308056
GPR97	3.43698
GPR98	2.59218
GPRASP1	2.596438

GPRASP2	2.836826
GPRC5A	9.132566
GPRC5B	6.853782
GPRC5C	4.48746
GPRC5D	3.06179
GPRC6A	2.306988
GPRIN1	5.659452
GPRIN2	4.999594
GPRIN3	2.857272
GPS1	8.113296
GPS2	7.69241
GPSM1	5.242212
GPSM2	7.834262
GPSM3	4.592536
GPSM3	5.220382
GPSM3	4.592536
GPT	4.249378
GPT2	7.5142
GPX1	7.245524
GPX2	5.819996
GPX3	7.629004
GPX4	9.350162
GPX5	2.770802
GPX6	2.967992
GPX7	3.086396
GPX8	5.862578
GRAMD1A	6.011908
GRAMD1B	5.8989
GRAMD1C	5.494842
GRAMD2	6.395402
GRAMD3	7.00238
GRAMD4	5.917702
GRAP	3.408386
GRAP2	2.935186
GRAPL	3.944998
GRAPL	4.44441
GRAPL	4.44441
GRASP	4.423992
GRB10	6.682848
GRB14	3.131888
GRB2	8.139432
GRB7	6.814618
GREB1	3.78168

GREB1L	4.617842
GREM1	4.348778
GREM2	3.259482
GRHL1	6.5823
GRHL2	8.727114
GRHL3	3.929046
GRHPR	7.868436
GRIA1	2.85796
GRIA2	2.876286
GRIA3	2.52862
GRIA4	2.725504
GRID1	3.98163
GRID2	2.697812
GRIK1	2.61292
GRIK1-AS	2.933172
GRIK2	2.656966
GRIK3	3.82868
GRIK4	4.00744
GRIK5	3.776838
GRIN1	4.095958
GRIN2A	3.010492
GRIN2B	3.114638
GRIN2C	3.800078
GRIN2D	4.695022
GRIN3A	2.582098
GRIN3B	5.570918
GRINA	7.154026
GRIP1	3.13401
GRIP2	3.696136
GRIPAP1	5.60605
GRK1	3.602768
GRK4	2.913706
GRK5	6.025906
GRK5	3.135222
GRK6	8.12166
GRK7	2.858252
GRLF1	8.338314
GRM1	3.169672
GRM2	3.555422
GRM3	2.564782
GRM4	4.896772
GRM5	5.22007
GRM6	3.696538

GRM7	2.95754
GRM8	3.197246
GRN	9.941128
GRP	4.342774
GRPEL1	8.857228
GRPEL2	8.08608
GRPR	2.775154
GRRP1	5.26368
GRSF1	7.547662
GRTP1	7.827058
GRWD1	7.835482
GS85	4.18141
GSC	4.615082
GSC2	5.843166
GSDMA	3.278638
GSDMB	6.721204
GSDMC	7.169716
GSDMD	7.166376
GSG1	3.092752
GSG1L	4.453294
GSG2	6.301178
GSK3A	7.31492
GSK3B	8.374792
GSN	9.16395
GSPT1	9.74942
GSPT2	3.228794
GSR	9.782604
GSS	8.467248
GSTA1	3.19744
GSTA2	2.13185
GSTA3	3.068778
GSTA4	2.856258
GSTA5	2.396634
GSTCD	6.344552
GSTK1	9.640192
GSTM1	3.611426
GSTM2	3.274628
GSTM2	3.198822
GSTM2P1	3.011396
GSTM3	4.77375
GSTM4	5.232434
GSTM5	3.087434
GSTO1	6.387456

GSTO2	4.724076
GSTP1	3.897322
GSTT1	3.476592
GSTT2	5.360176
GSTT2	5.360176
GSTTP1	2.896676
GSTTP1	3.286248
GSTTP1	2.544382
GSTZ1	6.045824
GSX1	5.894056
GSX2	5.29918
GTDC1	5.674636
GTF2A1	6.915644
GTF2A2	6.855242
GTF2B	7.809348
GTF2E1	7.856342
GTF2E2	6.83992
GTF2F1	7.802832
GTF2F2	6.591352
GTF2H1	6.365446
GTF2H2	5.954076
GTF2H2	6.07231
GTF2H2	5.66979
GTF2H2	5.66979
GTF2H2D	6.033042
GTF2H3	7.530142
GTF2H4	6.247962
GTF2H4	5.260242
GTF2H4	6.247962
GTF2H4	6.247962
GTF2H5	5.487596
GTF2I	8.955716
GTF2IRD1	7.134656
GTF2IRD2	5.53486
GTF2IRD2B	5.439332
GTF3A	8.125826
GTF3C1	7.571484
GTF3C2	7.953228
GTF3C3	8.558338
GTF3C4	8.005072
GTF3C5	7.736492
GTF3C6	7.936696
GTPBP1	7.435526

GTPBP10	6.767558
GTPBP2	8.277144
GTPBP3	5.909158
GTPBP4	8.662568
GTPBP5	7.439554
GTPBP6	5.662332
GTPBP8	6.52241
GTSCR1	3.059558
GTSE1	7.339718
GTSF1	2.63881
GTSF1L	2.479268
GUCA1A	4.341882
GUCA1B	4.046978
GUCA1C	1.933524
GUCA2A	4.466154
GUCA2B	4.903538
GUCY1A2	3.29268
GUCY1A3	2.542318
GUCY1B2	3.797888
GUCY1B3	3.122442
GUCY2C	2.579822
GUCY2D	4.518872
GUCY2F	2.911948
GUF1	6.63948
GUK1	8.181854
GULP1	5.978896
GUSB	9.683982
GUSBL1	10.32066
GUSBP1	8.197768
GUSBP1	10.072724
GUSBP3	6.309798
GUSBP3	8.2992
GUSBP3	6.913422
GUSBP3	6.924748
GUSBP4	5.914838
GVINP1	2.419642
GXYLT1	5.801028
GXYLT2	2.52896
GYG1	9.30919
GYG2	5.404082
GYLTL1B	5.521572
GYPA	2.206268
GYPB	2.101836

GYPC	4.108722
GYPE	3.01597
GYS1	7.037832
GYS2	2.438154
GZF1	6.120718
GZMA	3.110532
GZMB	4.346198
GZMH	3.408808
GZMK	2.015644
GZMM	4.586942
H19	4.52651
H1F0	8.994614
H1FNT	5.38754
H1FOO	4.561862
H1FX	7.05919
H2AFB1	3.467618
H2AFB1	2.80059
H2AFB1	3.467618
H2AFJ	6.36567
H2AFV	9.011316
H2AFX	7.321304
H2AFY	5.598314
H2AFY	9.390738
H2AFY2	7.257008
H2AFZ	10.56742
H2BFM	5.857432
H2BFWT	3.828032
H3F3A	9.910258
H3F3A	9.856446
H3F3A	10.024766
H3F3A	10.372958
H3F3B	9.373814
H3F3C	9.194234
H6PD	5.62207
HAAO	4.781298
HABP2	3.566322
HABP4	6.015288
HACE1	6.027192
HACL1	8.108192
HADH	7.9672
HADHA	9.188482
HADHB	8.902148
HAGH	7.268708

HAGHL	5.830394
HAL	3.25096
HAMP	3.93195
HAND1	3.59033
HAND2	3.444076
HAO1	2.513718
HAO2	2.805734
HAP1	5.770792
HAPLN1	2.41138
HAPLN2	4.579458
HAPLN3	3.746694
HAPLN4	3.949024
HAPLN4	7.704016
HARBI1	5.48488
HARS	8.262572
HARS2	7.115834
HAS1	5.38249
HAS2	3.033736
HAS3	5.219456
HAT1	7.904914
HAUS1	4.3235
HAUS2	5.808764
HAUS3	4.754712
HAUS4	7.698488
HAUS5	6.194654
HAUS6	5.40784
HAUS6	6.062822
HAUS7	5.358664
HAUS8	7.21047
HAVCR1	2.564222
HAVCR2	2.491172
HAX1	8.346126
HBA1	5.203886
HBA2	5.203886
HBB	3.52185
HBCBP	3.647302
HBD	3.096526
HBE1	2.993574
HBEGF	6.056046
HBG1	3.226288
HBG1	3.226288
HBII-52-27	2.017704
HBII-52-28	1.976148

HBM	5.05496
HBP1	7.547072
HBQ1	6.18994
HBS1L	8.234072
HBXIP	6.642308
HBZ	2.306012
HBZ	5.516686
HCCS	8.093774
HCFC1	7.300688
HCFC1R1	5.826088
HCFC2	4.587816
HCG27	4.668402
HCG27	4.93933
HCG27	4.875732
HCG4	5.902132
HCG8	4.104786
HCG9	3.471654
HCG9	3.471654
HCK	4.17986
HCLS1	3.664352
HCN1	2.901604
HCN2	5.535488
HCN3	5.301246
HCN4	4.211376
HCP5	8.004748
HCRP1	3.037342
HCRT	3.78781
HCRTR1	3.586238
HCRTR2	2.887252
HCST	4.665878
HDAC1	10.49484
HDAC10	5.869788
HDAC11	7.04797
HDAC2	8.216422
HDAC3	8.058308
HDAC4	4.540016
HDAC5	6.02285
HDAC6	5.978046
HDAC7	5.978326
HDAC8	6.285854
HDAC9	4.450338
HDC	2.681652
HDDC2	7.990238

HDDC3	6.11083
HDGF	10.9072
HDGFL1	5.358642
HDGFRP2	6.44827
HDGFRP3	5.445698
HDHD1	6.558032
HDHD2	5.895954
HDHD3	7.665152
HDLBP	3.575256
HDLBP	8.743054
HDX	2.773818
HEATR1	8.245222
HEATR2	7.283242
HEATR3	5.994498
HEATR4	3.800022
HEATR5A	7.35912
HEATR5B	6.429764
HEATR6	6.166178
HEATR7A	7.561406
HEATR7A	6.335548
HEATR7B1	3.665298
HEATR7B2	2.891918
HEBP1	7.647376
HEBP2	8.720136
HECA	6.358764
HECTD1	7.788258
HECTD2	3.833516
HECTD3	6.192272
HECW1	3.071434
HECW2	3.876386
HEG1	5.008444
HEJ1	2.239502
HELB	4.52952
HELLS	6.758742
HELQ	4.280422
HELT	4.26081
HELZ	6.34172
HEMGN	2.172448
HEMK1	5.08758
HEPACAM	3.289672
HEPACAM2	2.26329
HEPH	2.64131
HEPHL1	3.019624

HEPN1	3.39475
HERC1	5.95352
HERC2	7.01044
HERC2P2	8.00339
HERC2P2	8.0658
HERC2P3	8.13613
HERC2P4	7.05149
HERC2P4	7.55353
HERC3	6.951768
HERC4	8.01637
HERC5	8.5078
HERC6	9.51865
HERPUD1	9.1323
HERPUD2	7.47446
HES1	8.081694
HES2	4.652204
HES3	4.861904
HES4	6.465292
HES5	5.351368
HES6	5.331014
HES7	5.343954
HESX1	2.893408
HEXA	7.478998
HEXB	7.834974
HEXDC	5.200248
HEXIM1	8.574672
HEXIM2	6.210268
HEY1	2.80982
HEY2	3.171082
HEYL	4.258698
HFE	6.957628
HFE2	3.296818
HFM1	2.16711
HFM1	2.186734
HGC6.3	4.681434
HGD	2.623294
HGD	2.623294
HGF	2.715054
HGFAC	4.41711
HGS	7.519028
HGSNAT	8.13507
HHAT	5.045574
HHATL	4.17405

HHEX	4.915272
HHIP	2.917062
HHIPL1	4.383452
HHIPL2	4.526666
HHLA1	3.262236
HHLA2	2.753192
HHLA3	5.58798
HIAT1	9.906442
HIATL1	9.866204
HIATL2	9.293578
HIBADH	8.482598
HIBCH	5.119208
HIC1	5.347848
HIC2	5.57363
HIF1A	8.691898
HIF1AN	6.817766
HIF3A	4.137332
HIGD1A	8.52961
HIGD1A	8.328382
HIGD1B	3.92626
HIGD2A	7.832606
HIGD2B	3.491202
HILS1	5.01016
HINFP	6.501924
HINT1	8.230544
HINT2	7.873464
HINT3	6.302084
HIP1	5.031292
HIP1R	6.197658
HIPK1	6.675258
HIPK2	6.217776
HIPK3	7.339404
HIPK4	4.866444
HIRA	7.611718
HIRIP3	6.064684
HIST1H1A	8.928164
HIST1H1B	10.358474
HIST1H1C	7.394022
HIST1H1D	8.304958
HIST1H1E	10.95388
HIST1H1T	5.783584
HIST1H2AA	2.825856
HIST1H2AB	9.011568

HIST1H2AC	9.573456
HIST1H2AE	6.341688
HIST1H2AG	6.30249
HIST1H2AH	5.569666
HIST1H2AI	8.175206
HIST1H2AI	8.29738
HIST1H2AJ	3.859784
HIST1H2AK	7.00983
HIST1H2AL	6.534714
HIST1H2AM	5.624074
HIST1H2BA	2.673864
HIST1H2BB	3.109878
HIST1H2BC	6.326418
HIST1H2BD	6.757668
HIST1H2BE	6.740516
HIST1H2BF	8.613012
HIST1H2BG	7.052186
HIST1H2BH	9.398426
HIST1H2BI	4.84631
HIST1H2BJ	6.827222
HIST1H2BK	10.77062
HIST1H2BK	9.190192
HIST1H2BL	5.217732
HIST1H2BM	10.9992
HIST1H2BN	5.595506
HIST1H2BO	5.258036
HIST1H3A	7.19635
HIST1H3B	9.539802
HIST1H3C	5.31671
HIST1H3D	9.507602
HIST1H3E	4.036824
HIST1H3F	7.896598
HIST1H3G	7.70955
HIST1H3H	8.077856
HIST1H3I	10.9967
HIST1H3J	7.142634
HIST1H4A	7.361796
HIST1H4B	8.533006
HIST1H4C	9.517698
HIST1H4D	7.077946
HIST1H4E	7.325524
HIST1H4F	4.067186
HIST1H4G	2.90235

HIST1H4H	7.961052
HIST1H4I	4.966566
HIST1H4J	8.611224
HIST1H4K	9.099798
HIST1H4L	7.666736
HIST2H2AA3	10.42944
HIST2H2AA3	10.42944
HIST2H2AB	9.13986
HIST2H2AC	10.245062
HIST2H2BA	9.490126
HIST2H2BC	9.589236
HIST2H2BE	7.913666
HIST2H2BF	5.326926
HIST2H2BF	3.868574
HIST2H2BF	6.19546
HIST2H3A	9.094504
HIST2H3A	9.094504
HIST2H3D	9.387422
HIST2H3D	9.50732
HIST2H4A	8.219896
HIST2H4A	8.219896
HIST3H2A	7.049568
HIST3H2BB	6.450236
HIST3H3	4.271218
HIST4H4	4.880978
HIVEP1	6.888574
HIVEP2	5.673642
HIVEP3	3.99015
HJURP	8.789346
HK1	6.974946
HK2	7.082082
HK3	3.374016
HKDC1	3.233856
HKR1	4.608662
HLA-A	11.3053
HLA-A	11.41718
HLA-A	11.6319
HLA-B	11.31464
HLA-B	11.39714
HLA-B	11.52178
HLA-C	11.0937
HLA-C	11.09754
HLA-DMA	6.397882

HLA-DMA	6.484978
HLA-DMA	6.484978
HLA-DMB	4.68785
HLA-DMB	4.743256
HLA-DOA	4.000064
HLA-DOA	4.638024
HLA-DOB	4.58857
HLA-DOB	4.678608
HLA-DPA1	3.509746
HLA-DPA1	3.509746
HLA-DPA1	3.905014
HLA-DPB1	3.474176
HLA-DPB1	3.25978
HLA-DPB1	3.448676
HLA-DPB2	2.900496
HLA-DQA1	3.241526
HLA-DQA1	4.464676
HLA-DQA1	4.464676
HLA-DQA2	3.644894
HLA-DQA2	3.665044
HLA-DQB1	5.264412
HLA-DQB1	3.521832
HLA-DQB1	4.982886
HLA-DQB1	4.982886
HLA-DQB2	4.776328
HLA-DQB2	5.906828
HLA-DRA	4.010436
HLA-DRA	4.231076
HLA-DRA	3.99968
HLA-DRB3	4.218622
HLA-DRB3	4.380792
HLA-DRB4	4.089634
HLA-DRB5	3.51326
HLA-E	9.47783
HLA-E	9.59033
HLA-E	9.59033
HLA-F	7.63724
HLA-F	8.32407
HLA-F	8.245636
HLA-G	10.95228
HLA-G	10.95228
HLA-H	10.357812
HLA-J	11.4245

HLA-J	11.6381
HLA-L	4.907382
HLA-L	4.907382
HLCS	6.468632
HLF	3.361712
HLTF	7.32817
HLX	4.557558
HM13	8.367668
HMBOX1	5.561248
HMBS	7.306452
HMCN1	2.56269
HMCN2	4.14657
HMCN2	4.42815
HMG20A	6.924152
HMG20B	7.542848
HMGA1	6.911482
HMGA2	4.104732
HMGA2	3.610022
HMGB1	6.080148
HMGB1	10.63106
HMGB2	8.204796
HMGB3	6.947996
HMGB3P1	3.590898
HMGB4	2.367588
HMGB4	2.970014
HMGCL	7.674576
HMGCLL1	3.000182
HMGCR	8.179874
HMGCS1	8.75075
HMGCS2	3.295082
HMGN1	6.36506
HMGN1	9.409076
HMGN2	11.37054
HMGN2	11.68228
HMGN2	11.5976
HMGN3	6.547414
HMGN4	7.423964
HMGN5	2.943876
HMGXB3	7.595346
HMGXB4	7.932564
HMHA1	4.735854
HMHB1	4.225314
HMMR	6.56076

HMOX1	6.51602
HMOX2	7.197378
HMP19	2.729584
HMX1	5.709924
HMX2	3.988664
HMX3	5.38472
HN1	8.147534
HN1L	8.046914
HNF1A	4.067208
HNF1B	3.439312
HNF4A	4.365854
HNF4G	2.520616
HNMT	4.279104
HNRNPA0	8.942462
HNRNPA1	10.50102
HNRNPA1	5.69361
HNRNPA1	11.24526
HNRNPA1	11.06064
HNRNPA1L2	4.535516
HNRNPA1P1(	11.29644
HNRNPA2B1	9.726832
HNRNPA3	10.40766
HNRNPA3	9.849842
HNRNPA3	10.37066
HNRNPA3P1	7.328418
HNRNPAB	11.01732
HNRNPC	10.77588
HNRNPC	9.655626
HNRNPCL1	3.538408
HNRNPD	10.62496
HNRNPF	10.60962
HNRNPH1	10.297584
HNRNPH2	7.839458
HNRNPH3	7.053066
HNRNPK	11.28306
HNRNPL	9.520548
HNRNPM	10.41282
HNRNPR	9.559996
HNRNPU	10.053702
HNRNPUL1	8.353308
HNRNPUL2	9.364054
HNRPDL	8.418886
HNRPLL	6.596262

HOMER1	6.073776
HOMER2	5.732986
HOMER3	8.165198
HOOK1	5.325366
HOOK2	7.271466
HOOK3	6.501718
HOPX	3.206184
HORMAD1	8.138204
HORMAD2	2.73736
HOXA1	4.378736
HOXA10	5.768582
HOXA11	3.679732
HOXA13	5.380954
HOXA2	3.57756
HOXA3	3.47146
HOXA4	4.553286
HOXA5	4.754712
HOXA6	4.607426
HOXA7	5.136254
HOXA9	5.12552
HOXB1	4.458786
HOXB13	4.679438
HOXB2	2.469718
HOXB3	5.441502
HOXB4	4.86499
HOXB5	2.703098
HOXB6	4.689746
HOXB7	5.898594
HOXB8	3.852322
HOXB9	5.163116
HOXC10	6.170318
HOXC11	5.684492
HOXC12	3.900552
HOXC13	7.168734
HOXC5	3.770836
HOXC6	5.122862
HOXC8	4.745634
HOXC9	5.015422
HOXD1	5.373696
HOXD10	3.273468
HOXD11	3.929848
HOXD12	2.882628
HOXD13	4.536804

HOXD3	2.988082
HOXD4	4.601338
HOXD8	3.325142
HOXD9	4.375138
HP	2.289638
HP1BP3	9.05487
HPCA	5.122144
HPCAL1	8.313866
HPCAL4	3.235464
HPD	3.310786
HPDL	4.82174
HPGD	2.306124
HPGDS	2.223976
HPN	4.23362
HPR	2.352014
HPRT1	8.701906
HPS1	5.559788
HPS3	8.691678
HPS4	6.562396
HPS5	6.006248
HPS6	5.198586
HPSE	5.872134
HPSE2	2.581378
HPX	3.92976
HR	6.487932
HRAS	6.692856
HRASLS	3.314202
HRASLS2	7.437624
HRASLS5	4.608862
HRC	3.622284
HRCT1	4.364656
HRG	2.842972
HRH1	6.037156
HRH2	3.172108
HRH3	4.235086
HRH4	2.377038
HRK	7.419384
HRNR	2.361736
HRSP12	7.360702
HS1BP3	5.101902
HS2ST1	6.394868
HS3ST1	5.456446
HS3ST2	4.668014

HS3ST3A1	4.773022
HS3ST3B1	3.718062
HS3ST4	4.762964
HS3ST5	3.870012
HS3ST6	5.629006
HS6ST1	5.961584
HS6ST1	6.423362
HS6ST2	5.123338
HS6ST3	3.876748
HSBP1	7.947776
HSCB	6.41447
HSD11B1	2.887192
HSD11B1L	3.964896
HSD11B2	5.075182
HSD17B1	5.428064
HSD17B10	8.788086
HSD17B11	6.178818
HSD17B12	8.073066
HSD17B13	2.63154
HSD17B14	3.666394
HSD17B2	4.05686
HSD17B3	2.790416
HSD17B4	7.750438
HSD17B6	4.627748
HSD17B7	5.377076
HSD17B7P2	4.437888
HSD17B8	6.966614
HSD17B8	6.966614
HSD17B8	6.966614
HSD3B1	2.507538
HSD3B1	2.634854
HSD3B2	2.475146
HSD3B7	5.95401
HSD3BP4	3.847912
HSDL1	5.79575
HSDL2	8.38023
HSF1	8.14553
HSF2	5.558104
HSF2BP	5.459254
HSF4	4.541246
HSF5	2.949894
HSFX1	4.035764
HSFX1	4.035764

HSFY2	2.213068
HSFY2	2.213068
HSH2D	5.88137
HSP90AA1	10.187106
HSP90AA2	7.146362
HSP90AA4P	3.608902
HSP90AA5P	2.665262
HSP90AA6P	3.507302
HSP90AB1	11.54228
HSP90AB2P	5.920736
HSP90AB3P	8.461436
HSP90AB4P	3.603044
HSP90AB5P	3.25295
HSP90AB6P	2.833962
HSP90B1	10.131042
HSP90B2P	2.528376
HSP90B3P	3.5692
HSPA12A	4.73211
HSPA12B	4.857844
HSPA13	7.223364
HSPA14	5.911486
HSPA1A	10.94314
HSPA1A	10.9238
HSPA1B	9.085926
HSPA1B	9.171244
HSPA1B	9.171244
HSPA1L	4.132512
HSPA1L	4.132512
HSPA1L	4.132512
HSPA2	6.97056
HSPA4	7.988166
HSPA4L	5.443668
HSPA5	11.16598
HSPA6	3.645776
HSPA7	6.455576
HSPA8	12.1088
HSPA9	10.16138
HSPB1	11.36964
HSPB11	5.991598
HSPB2	3.825766
HSPB3	2.648554
HSPB6	4.59996
HSPB7	3.18021

HSPB8	3.064088
HSPB9	3.876818
HSPBAP1	4.707448
HSPBP1	7.524848
HSPC157	6.467042
HSPC159	6.715294
HSPD1	10.315044
HSPE1	7.378284
HSPG2	6.354304
HSPH1	8.969652
HTATIP2	8.536798
HTATSF1	8.877206
HTN1	2.48637
HTN3	2.444866
HTR1A	3.624522
HTR1B	5.378778
HTR1D	6.746588
HTR1E	2.908212
HTR1F	2.753652
HTR2A	2.735988
HTR2B	2.449054
HTR2C	3.689346
HTR3A	3.834698
HTR3B	2.600542
HTR3C	3.234564
HTR3D	4.319748
HTR3E	3.772446
HTR4	3.03293
HTR5A	4.252274
HTR6	4.379524
HTR7	4.157872
HTR7P1	4.994566
HTRA1	7.470008
HTRA2	6.630246
HTRA3	5.04992
HTRA4	3.30263
HTT	6.372204
HUNK	5.309016
HUS1	6.10649
HUS1B	5.03587
HUWE1	7.961032
HVCN1	3.59617
HYAL1	5.18513

HYAL2	5.3946
HYAL3	4.360958
HYAL4	2.971828
HYDIN	2.698496
HYDIN	3.080342
HYDIN	3.394658
HYDIN	3.426408
HYI	5.350702
HYLS1	3.44439
HYOU1	8.136204
IAH1	7.49878
IAPP	1.967346
IARS	10.57898
IARS2	8.231896
IBSP	2.906056
IBTK	7.446952
ICA1	5.32164
ICA1L	2.639038
ICAM1	6.976304
ICAM2	6.248476
ICAM3	5.73795
ICAM4	3.551768
ICAM5	5.396588
ICK	5.352364
ICMT	7.270684
ICOS	2.295108
ICOSLG	5.091512
ICT1	8.47147
ID1	9.031838
ID2	5.387272
ID2B	4.343948
ID3	8.749974
ID4	6.018218
IDE	6.050232
IDH1	10.141792
IDH2	7.972756
IDH3A	9.273432
IDH3B	9.033226
IDH3G	8.021274
IDI1	8.34096
IDI2	2.71702
IDO1	3.249978
IDO2	2.572096

IDS	7.102734
IDS	7.39902
IDUA	5.251454
IER2	9.234218
IER3	7.95071
IER3	8.217852
IER3	7.95071
IER3IP1	2.41018
IER3IP1	4.757208
IER3IP1	5.844532
IER5	8.048852
IER5L	6.595902
IFFO1	4.187434
IFFO2	6.0349
IFI16	4.414946
IFI27	8.267436
IFI27L1	5.88322
IFI27L2	5.922846
IFI30	8.880962
IFI35	10.111132
IFI44	8.95319
IFI44L	8.270418
IFI6	10.720308
IFIH1	8.114214
IFIT1	10.253306
IFIT1B	2.661596
IFIT2	8.99318
IFIT3	8.23445
IFIT5	6.764626
IFITM1	10.002606
IFITM2	7.76871
IFITM3	8.830912
IFITM5	3.20203
IFLTD1	2.436686
IFNA1	2.629774
IFNA10	2.039084
IFNA13	3.80301
IFNA14	2.876448
IFNA16	2.16712
IFNA17	2.620754
IFNA2	2.193188
IFNA21	2.18527
IFNA4	2.541408

IFNA5	2.26965
IFNA6	2.87314
IFNA8	3.170358
IFNAR1	7.57504
IFNAR2	5.510894
IFNB1	2.540302
IFNE	2.64133
IFNG	2.628766
IFNGR1	7.608692
IFNGR2	7.344002
IFNK	2.532968
IFNW1	2.517402
IFRD1	7.323962
IFRD2	4.565222
IFRD2	6.709256
IFT122	7.072332
IFT140	4.811658
IFT172	5.401082
IFT20	6.896154
IFT27	6.413128
IFT46	7.322962
IFT52	5.978834
IFT57	5.296854
IFT74	3.355634
IFT80	6.977878
IFT81	3.99354
IFT88	3.777966
IGBP1	7.735162
IGDCC3	4.433584
IGDCC4	3.661404
IGF1	2.829704
IGF1R	4.400084
IGF1R	4.410686
IGF2	2.826532
IGF2AS	4.654456
IGF2BP1	3.923324
IGF2BP2	6.933402
IGF2BP3	8.446018
IGF2BP3	4.988052
IGF2R	7.574476
IGFALS	4.859126
IGFBP1	4.56773
IGFBP2	6.589786

IGFBP3	9.507982
IGFBP4	6.31474
IGFBP5	5.404538
IGFBP6	9.062178
IGFBP7	4.301046
IGFBPL1	5.827258
IGFL1	3.907754
IGFL2	3.042356
IGFL3	3.31289
IGFL4	3.272012
IGFN1	3.91358
IGHA1	6.8584
IGHA1	3.194892
IGHA2	3.781774
IGHD	4.014134
IGHE	5.245982
IGHM	2.881888
IGHMBP2	5.526558
IGHV1OR21-	2.7987
IGHV3-48	3.820108
IGHV3-48	4.859726
IGHV4-59	2.812178
IGHV5-78	4.34837
IGHV7-81	2.861758
IGJ	2.47414
IGK@	3.377262
IGK@	3.443294
IGK@	3.495888
IGKC	3.263886
IGKC	6.173386
IGKC	2.61659
IGKC	3.979356
IGKC	2.991564
IGKV1-5	6.36523
IGKV1D-8	3.07977
IGKV2-40	2.372814
IGKV3D-11	2.686174
IGKV4-1	2.654018
IGLJ3	4.874624
IGLL1	5.634558
IGLL3P	5.621644
IGLL5	4.85702
IGLON5	3.421516

IGLV6-57	3.89131
IGLV7-46	3.165038
IGSF1	2.844028
IGSF10	2.548464
IGSF11	3.413466
IGSF21	4.137134
IGSF22	3.316556
IGSF3	5.160368
IGSF5	2.883786
IGSF6	3.061836
IGSF8	5.981908
IGSF9	6.032276
IGSF9B	3.696186
IGSF9B	3.9031
IHH	4.048598
IK	8.650014
IKBIP	2.98468
IKBKAP	7.979232
IKBKB	7.051414
IKBKE	5.700322
IKBKE	3.348922
IKBKG	4.365592
IKZF1	2.691336
IKZF2	4.1381
IKZF3	2.717404
IKZF4	4.366176
IKZF5	6.75117
IL10	3.073162
IL10RA	3.01129
IL10RB	8.797924
IL11	5.511952
IL11RA	4.526808
IL12A	2.921102
IL12B	3.589878
IL12RB1	3.256578
IL12RB2	2.831006
IL13	3.389266
IL13RA1	7.21683
IL13RA2	2.827568
IL15	3.280438
IL15RA	6.54644
IL16	3.799624
IL17A	3.982656

IL17B	3.758882
IL17C	5.578922
IL17D	5.788656
IL17F	3.115972
IL17RA	6.875964
IL17RB	4.880558
IL17RC	6.75395
IL17RD	4.383914
IL17RE	5.852596
IL17REL	3.703906
IL18	7.83881
IL18BP	4.90145
IL18R1	2.63073
IL18RAP	2.772612
IL19	2.856788
IL1A	3.200928
IL1B	4.834914
IL1F10	3.442888
IL1F5	4.118908
IL1F6	3.69895
IL1F7	2.694266
IL1F8	2.580468
IL1F9	3.13736
IL1R1	4.21389
IL1R2	4.73286
IL1RAP	7.225972
IL1RAPL1	3.055858
IL1RAPL2	2.745454
IL1RL1	2.695742
IL1RL2	3.998628
IL1RN	5.628944
IL2	2.232606
IL20	2.61
IL20RA	4.699408
IL20RB	6.74018
IL21	2.696354
IL21R	3.805754
IL22	2.620522
IL22RA1	4.38555
IL22RA2	2.948588
IL23A	4.97582
IL23R	2.154048
IL24	2.624704

IL25	3.294974
IL26	2.399832
IL27	4.21339
IL27RA	6.99813
IL27RA	8.345554
IL28A	6.583688
IL28B	7.808394
IL28RA	4.906734
IL29	6.758278
IL2RA	3.212978
IL2RB	4.078612
IL2RG	4.73781
IL3	3.028532
IL31	2.494448
IL31RA	2.601404
IL32	5.464162
IL33	2.387722
IL34	5.61266
IL3RA	2.765442
IL3RA	2.765442
IL4	2.499258
IL4I1	5.48984
IL4R	7.667954
IL5	2.362974
IL5RA	2.624814
IL6	7.043576
IL6	3.065362
IL6R	4.899088
IL6ST	6.135036
IL7	2.460764
IL7R	6.571138
IL8	5.374732
IL9	2.669278
IL9R	4.752874
IL9R	4.752874
ILDR1	4.614568
ILDR2	3.329466
ILF2	10.86134
ILF3	9.131398
ILK	7.109562
ILKAP	4.888942
ILVBL	7.109462
IMMP1L	5.09011

IMMP2L	3.548582
IMMT	9.00903
IMP3	7.268492
IMP4	8.831848
IMP5	4.82516
IMPA1	6.285432
IMPA2	9.177994
IMPACT	6.542052
IMPAD1	5.96069
IMPAD1	8.73679
IMPDH1	6.156922
IMPDH2	8.689906
IMPG1	2.657016
IMPG2	3.0419
INA	7.477002
INADL	7.68962
INCA1	3.984438
INCENP	7.338664
INE1	3.731768
INF2	5.939918
INF2	7.161946
ING1	5.82497
ING2	5.938506
ING3	5.187312
ING4	8.262636
ING5	4.19365
ING5	4.344546
INGX	4.869356
INHA	4.4158
INHBA	5.520214
INHBB	6.085954
INHBC	3.813596
INHBE	3.446562
INMT	4.43082
INO80	7.39108
INO80B	5.922346
INO80C	4.882782
INO80D	6.121676
INO80E	6.47158
INPP1	6.907008
INPP4A	6.093408
INPP4B	2.564564
INPP4B	7.853944

INPP5A	7.103506
INPP5B	7.178132
INPP5D	3.983654
INPP5E	5.604778
INPP5F	3.823884
INPP5J	4.673732
INPP5K	6.867466
INPPL1	6.523864
INSC	2.933276
INSIG1	8.248234
INSIG2	7.53415
INS-IGF2	4.351862
INSL3	5.00906
INSL4	2.508308
INSL5	2.288996
INSL6	2.879514
INSM1	5.72732
INSM2	5.13423
INSR	5.589842
INSRR	3.20473
INTS1	6.527728
INTS10	6.44342
INTS12	5.835286
INTS2	6.267054
INTS3	8.114902
INTS4	7.1612
INTS4L1	8.037776
INTS4L2	8.034536
INTS5	6.905326
INTS6	4.60471
INTS7	8.313068
INTS8	7.678532
INTS9	7.075082
INTU	3.653278
INVS	5.68751
IP6K1	6.352276
IP6K2	6.753072
IP6K3	3.616098
IPCEF1	3.088042
IPMK	5.465056
IPO11	7.239542
IPO13	6.2259
IPO4	6.844716

IPO5	9.738772
IPO7	7.652024
IPO8	8.05186
IPO9	9.379024
IPP	6.943216
IPPK	7.38312
IQCA1	2.929222
IQCB1	7.648284
IQCC	5.222438
IQCD	4.07952
IQCE	6.7554
IQCF1	2.665312
IQCF2	2.107304
IQCG	4.594988
IQCH	3.293582
IQCJ	2.727716
IQCK	5.806538
IQGAP1	9.359914
IQGAP2	3.398722
IQGAP3	6.822062
IQSEC1	5.708036
IQSEC2	4.505404
IQSEC3	6.512678
IQSEC3	4.37554
IQSEC3	6.338632
IQUB	2.39288
IRAK1	7.748794
IRAK1BP1	3.990538
IRAK2	6.879598
IRAK3	2.188342
IRAK4	5.886088
IREB2	7.723468
IRF1	7.835386
IRF2	7.267168
IRF2BP1	6.33118
IRF2BP2	8.071514
IRF3	7.670402
IRF4	2.744676
IRF5	5.12761
IRF6	7.263612
IRF7	4.961646
IRF8	3.443294
IRF9	8.368242

IRG1	2.289718
IRGC	3.469762
IRGQ	4.724898
IRS1	4.833926
IRS2	5.575198
IRS4	3.52939
IRX1	4.495004
IRX2	6.134844
IRX3	7.811274
IRX4	5.348314
IRX5	6.59586
IRX6	4.728382
ISCA1	6.83368
ISCA1	6.698724
ISCA2	6.602142
ISCU	7.372198
ISG15	6.846772
ISG20	6.068012
ISG20L2	8.469976
ISL1	3.691414
ISL2	5.399978
ISLR	2.981016
ISLR2	3.525776
ISM1	3.185128
ISM2	3.572878
ISOC1	6.63993
ISOC2	7.432456
ISX	3.151252
ISY1	7.487888
ISYNA1	6.225018
ITCH	8.113246
ITFG1	7.611168
ITFG1	2.95878
ITFG2	6.96976
ITFG3	7.227964
ITGA10	3.233354
ITGA11	3.161308
ITGA2	8.266444
ITGA2B	4.025058
ITGA3	9.577068
ITGA4	3.284912
ITGA5	6.953136
ITGA6	9.285732

ITGA7	3.53642
ITGA8	2.601962
ITGA9	4.108824
ITGAD	3.563272
ITGAE	4.49091
ITGAL	2.883322
ITGAM	4.300552
ITGAV	7.785904
ITGAX	3.772542
ITGB1	9.934984
ITGB1BP1	7.469172
ITGB1BP2	3.37393
ITGB1BP3	4.90184
ITGB2	5.297732
ITGB3	3.018426
ITGB3BP	6.397052
ITGB4	7.107
ITGB5	7.86649
ITGB6	7.04908
ITGB7	4.647778
ITGB8	7.452432
ITGBL1	4.965352
ITIH1	3.498022
ITIH2	3.071684
ITIH3	3.905732
ITIH4	4.000688
ITIH5	3.843732
ITIH5L	3.58819
ITK	3.11863
ITLN1	2.913274
ITLN2	3.072068
ITM2A	2.975114
ITM2B	9.094798
ITM2C	7.47598
ITPA	7.259126
ITPK1	7.590788
ITPKA	4.94293
ITPKB	4.574666
ITPKC	6.179062
ITPR1	4.74298
ITPR2	5.895948
ITPR3	8.20524
ITPRIP	6.532802

ITPRIPL1	3.442884
ITPRIPL2	6.996728
ITSN1	5.818768
ITSN2	5.568042
IVD	8.229956
IVL	4.145748
IVNS1ABP	8.518554
IWS1	7.098138
IYD	2.74418
IZUMO1	3.874564
IZUMO2	3.243684
IZUMO4	4.50949
JAG1	7.020676
JAG2	5.63462
JAGN1	9.155794
JAK1	8.561056
JAK2	4.195564
JAK3	4.115228
JAKMIP1	3.804664
JAKMIP2	2.514146
JAKMIP3	4.836022
JAM2	2.862128
JAM3	2.887062
JARID2	6.645534
JAZF1	5.601
JDP2	6.925704
JHDM1D	3.013906
JKAMP	7.739224
JMJD1C	5.659078
JMJD1C	5.553322
JMJD4	5.87091
JMJD5	4.873492
JMJD5	2.934208
JMJD6	6.437366
JMJD7-PLA2(	5.3134
JMJD8	6.846066
JMY	4.74181
JOSD1	9.726506
JOSD2	5.7664
JPH1	4.30831
JPH2	5.733686
JPH3	4.46529
JPH4	5.337684

JRK	6.517214
JRKL	6.23311
JSRP1	6.125132
JTB	9.634748
JUB	7.389842
JUN	8.354148
JUNB	8.216332
JUND	7.592082
JUP	9.145148
KAAG1	3.33928
KAL1	2.606714
KALRN	3.682172
KANK1	6.00665
KANK2	6.595138
KANK3	4.16509
KANK4	3.915426
KARS	9.062
KAT2A	6.717168
KAT2B	6.488642
KAT5	7.145734
KATNA1	5.856984
KATNAL1	5.132352
KATNAL2	3.8373
KATNB1	5.703166
KAZ	5.362332
KAZ	3.808874
KAZ	4.281314
KAZALD1	5.833906
KBTBD10	2.318886
KBTBD11	4.394112
KBTBD12	3.236526
KBTBD13	4.826018
KBTBD2	7.169864
KBTBD3	2.687776
KBTBD4	7.70039
KBTBD5	3.927396
KBTBD6	6.31334
KBTBD7	5.459132
KBTBD8	2.493588
KCMF1	9.184912
KCNA1	3.561144
KCNA10	3.766716
KCNA2	2.929898

KCNA3	3.5416
KCNA4	3.19995
KCNA5	3.356868
KCNA6	3.706844
KCNA7	3.633596
KCNAB1	3.1343
KCNAB2	5.737636
KCNAB3	3.236492
KCNB1	3.926716
KCNB2	2.830824
KCNC1	4.558998
KCNC2	3.512854
KCNC3	5.189256
KCNC4	5.239174
KCND1	3.168704
KCND2	3.373328
KCND3	2.806848
KCNE1	3.733766
KCNE1L	4.272896
KCNE2	2.790094
KCNE3	3.342436
KCNE4	3.69828
KCNF1	5.193066
KCNG1	4.83287
KCNG1	4.69846
KCNG2	5.548342
KCNG3	4.315232
KCNG4	4.380816
KCNH1	3.033154
KCNH2	4.84004
KCNH3	4.500856
KCNH4	4.459922
KCNH5	2.44653
KCNH6	3.872348
KCNH7	2.59185
KCNH8	2.995742
KCNIP1	2.98602
KCNIP2	3.77737
KCNIP3	5.293052
KCNIP4	2.57778
KCNJ1	2.396762
KCNJ10	2.633772
KCNJ11	3.75858

KCNJ12	6.083578
KCNJ13	2.572026
KCNJ14	4.179564
KCNJ15	2.857394
KCNJ16	2.745564
KCNJ2	3.46468
KCNJ3	3.34763
KCNJ4	5.464314
KCNJ5	4.255234
KCNJ6	2.600048
KCNJ8	3.206792
KCNJ9	3.80976
KCNK1	8.255446
KCNK10	2.785336
KCNK12	5.444696
KCNK13	4.831144
KCNK15	5.88076
KCNK16	3.872802
KCNK17	3.530242
KCNK18	3.603184
KCNK2	2.724252
KCNK3	4.986556
KCNK4	5.024684
KCNK5	7.420022
KCNK6	5.835174
KCNK7	5.267288
KCNK9	3.591684
KCNMA1	2.888586
KCNMB1	4.046238
KCNMB2	2.54432
KCNMB3	4.71581
KCNMB4	5.235552
KCNN1	4.111638
KCNN2	2.928026
KCNN3	4.256778
KCNN4	7.669802
KCNQ1	4.501642
KCNQ1DN	4.899216
KCNQ2	3.539828
KCNQ3	4.224238
KCNQ4	4.075526
KCNQ5	3.067278
KCNRG	4.219822

KCNS1	2.882202
KCNS2	3.499408
KCNS3	7.200936
KCNT1	3.884728
KCNT2	2.41487
KCNV1	3.347046
KCNV2	3.880604
KCP	5.709248
KCP	4.822656
KCTD1	6.413644
KCTD10	7.291752
KCTD11	5.923086
KCTD12	4.997476
KCTD13	5.897054
KCTD14	5.963776
KCTD15	6.501862
KCTD16	3.232184
KCTD17	5.729914
KCTD18	6.311654
KCTD19	3.157856
KCTD2	7.048848
KCTD20	6.949034
KCTD21	3.348208
KCTD21	5.17175
KCTD3	6.98615
KCTD4	3.268414
KCTD5	7.69655
KCTD6	5.430048
KCTD7	5.386728
KCTD8	4.705218
KCTD9	8.296426
KDELC1	5.607286
KDELC2	6.594598
KDELR1	9.420848
KDELR2	10.18352
KDELR3	6.777016
KDM1A	9.541222
KDM1B	6.577876
KDM2A	7.932854
KDM2B	6.217296
KDM3A	6.172124
KDM3B	8.31935
KDM4A	8.139686

KDM4B	5.35043
KDM4C	6.039976
KDM4D	3.883296
KDM5A	8.346568
KDM5B	8.533514
KDM5C	6.684974
KDM5D	2.96393
KDM6A	6.074338
KDM6B	5.502754
KDR	3.208922
KDSR	6.746938
KEAP1	8.268618
KEL	3.610518
KERA	2.717802
KGFLP1	2.517852
KGFLP1	2.912168
KGFLP1	2.912168
KGFLP1	2.912168
KGFLP2	2.952728
KGFLP2	2.94584
KGFLP2	4.035974
KGFLP2	3.447294
KHDC1	3.951812
KHDRBS1	7.911956
KHDRBS2	3.117022
KHDRBS3	4.287108
KHK	4.68531
KHNYN	5.914332
KHSRP	7.639742
KIAA0020	5.67971
KIAA0040	5.239062
KIAA0087	2.702794
KIAA0090	8.458124
KIAA0100	8.580756
KIAA0101	9.176286
KIAA0125	4.128608
KIAA0125	2.899768
KIAA0141	6.327782
KIAA0146	7.738032
KIAA0174	9.88018
KIAA0182	7.303656
KIAA0195	6.395144
KIAA0196	7.997798

KIAA0226	7.77752
KIAA0232	6.535222
KIAA0240	5.7142
KIAA0247	6.129852
KIAA0284	6.192836
KIAA0317	7.786772
KIAA0319	4.471282
KIAA0319L	7.15334
KIAA0355	5.905516
KIAA0368	8.749876
KIAA0391	6.846682
KIAA0415	6.031052
KIAA0427	4.293222
KIAA0430	7.599884
KIAA0467	5.125488
KIAA0494	8.379772
KIAA0495	3.991396
KIAA0513	6.83962
KIAA0528	6.78191
KIAA0556	6.093902
KIAA0562	6.75261
KIAA0564	6.170514
KIAA0586	5.943806
KIAA0649	6.836552
KIAA0664	7.009316
KIAA0664L3	5.924614
KIAA0748	2.613402
KIAA0753	6.595582
KIAA0776	6.742862
KIAA0802	4.569998
KIAA0895	5.745332
KIAA0895L	5.656752
KIAA0907	6.962566
KIAA0913	5.14762
KIAA0913	3.997444
KIAA0922	5.847798
KIAA0947	7.040984
KIAA1009	3.232186
KIAA1024	3.418156
KIAA1033	6.244266
KIAA1045	3.129594
KIAA1107	2.812748
KIAA1109	5.786516

KIAA1143	7.041052
KIAA1143	6.547008
KIAA1147	5.596852
KIAA1161	5.33505
KIAA1191	7.643798
KIAA1199	3.839524
KIAA1210	2.840274
KIAA1211	4.892968
KIAA1217	6.60423
KIAA1239	2.774566
KIAA1244	5.756054
KIAA1257	4.016254
KIAA1267	6.60751
KIAA1274	4.29581
KIAA1279	7.887614
KIAA1310	6.41469
KIAA1324	3.096878
KIAA1324L	2.315682
KIAA1328	2.628732
KIAA1328	2.734394
KIAA1370	5.564218
KIAA1377	3.232758
KIAA1383	3.864412
KIAA1407	4.109906
KIAA1409	2.752932
KIAA1429	7.9507
KIAA1430	6.012374
KIAA1432	6.25884
KIAA1462	3.911112
KIAA1467	6.218744
KIAA1468	5.555514
KIAA1486	2.759482
KIAA1522	6.797458
KIAA1524	7.265772
KIAA1530	5.300846
KIAA1539	5.411474
KIAA1543	6.11502
KIAA1549	5.993342
KIAA1586	5.02019
KIAA1598	6.18922
KIAA1609	7.477384
KIAA1614	3.64553
KIAA1632	4.94334

KIAA1644	3.815488
KIAA1654	3.73866
KIAA1656	2.5172
KIAA1671	7.287938
KIAA1683	4.731998
KIAA1704	7.861538
KIAA1712	4.955304
KIAA1715	6.161678
KIAA1731	4.931678
KIAA1737	5.951746
KIAA1751	3.193744
KIAA1755	3.995302
KIAA1797	5.690688
KIAA1804	5.38814
KIAA1826	5.760074
KIAA1841	6.25154
KIAA1875	3.938368
KIAA1919	6.490832
KIAA1949	5.425566
KIAA1958	5.702274
KIAA1967	6.966416
KIAA1984	4.475914
KIAA2013	5.661246
KIAA2013	7.504838
KIAA2018	6.289506
KIAA2022	2.75831
KIAA2026	5.95232
KIDINS220	6.607348
KIF11	6.545902
KIF12	4.649146
KIF13A	7.381328
KIF13B	6.327724
KIF14	6.084346
KIF15	5.378352
KIF16B	4.288262
KIF17	3.925968
KIF18A	5.496882
KIF18B	6.154806
KIF18B	6.37514
KIF19	4.44989
KIF19	4.658994
KIF1A	3.616244
KIF1B	6.192964

KIF1C	7.924404
KIF20A	9.009864
KIF20B	4.436114
KIF21A	4.60088
KIF21B	4.384992
KIF22	8.858594
KIF22	8.86806
KIF23	9.161508
KIF24	5.025348
KIF25	3.350148
KIF26A	4.733816
KIF26B	3.38728
KIF27	5.02183
KIF27	6.466836
KIF27	4.182932
KIF2A	6.57053
KIF2B	4.603808
KIF2C	8.293488
KIF3A	4.050624
KIF3B	6.599362
KIF3C	5.117258
KIF4A	7.949112
KIF4B	5.858702
KIF5A	3.072728
KIF5A	3.231126
KIF5B	9.061376
KIF5C	3.99552
KIF6	2.991902
KIF7	5.58545
KIF9	3.150024
KIFAP3	7.154134
KIFC1	8.294438
KIFC1	8.56912
KIFC2	5.657332
KIFC3	5.177148
KIN	5.779646
KIR2DL1	3.489138
KIR2DL2	3.303972
KIR2DL3	3.14991
KIR2DL3	3.232836
KIR2DL4	3.238922
KIR2DL4	3.147526
KIR2DL5A	3.794092

KIR2DS1	3.53114
KIR2DS2	3.479872
KIR2DS4	3.658062
KIR2DS5	3.036726
KIR3DL1	3.18561
KIR3DL1	4.800454
KIR3DL2	3.313384
KIR3DL3	3.674938
KIR3DL3	3.687852
KIR3DS1	3.283108
KIR3DX1	2.772716
KIRREL	6.491482
KIRREL2	3.436264
KIRREL3	4.486836
KISS1	4.135922
KISS1R	5.078618
KIT	2.595838
KITLG	6.792004
KL	2.750464
KLB	3.185028
KLC1	7.381808
KLC2	6.966078
KLC3	5.169508
KLC4	6.211318
KLF1	4.96433
KLF10	7.708084
KLF11	6.175034
KLF12	3.358448
KLF13	7.12838
KLF14	5.04409
KLF15	3.408886
KLF16	6.82097
KLF17	3.865828
KLF2	6.177556
KLF3	7.804888
KLF4	8.842996
KLF5	8.230674
KLF6	6.82548
KLF7	6.066856
KLF8	3.738366
KLF9	5.160252
KLHDC1	2.812256
KLHDC10	7.245434

KLHDC10	5.670738
KLHDC10	3.015986
KLHDC2	8.11968
KLHDC3	8.585038
KLHDC4	5.9327
KLHDC5	6.79361
KLHDC7A	4.986618
KLHDC7B	7.164178
KLHDC8A	3.653632
KLHDC8B	4.847304
KLHDC9	3.118518
KLHL1	2.926254
KLHL10	2.821572
KLHL11	6.310986
KLHL12	7.126698
KLHL13	2.89324
KLHL14	2.995398
KLHL15	3.697546
KLHL17	5.228972
KLHL18	5.826762
KLHL2	5.727812
KLHL20	6.967432
KLHL21	4.904388
KLHL22	5.758388
KLHL23	5.54465
KLHL24	6.107554
KLHL25	4.003428
KLHL26	6.20557
KLHL28	6.18863
KLHL29	4.54589
KLHL3	3.395616
KLHL30	4.324104
KLHL31	3.424474
KLHL31	2.962324
KLHL32	2.385752
KLHL33	3.984382
KLHL34	5.446342
KLHL35	5.648178
KLHL36	6.749758
KLHL38	3.376308
KLHL4	2.40316
KLHL5	6.991128
KLHL6	4.084552

KLHL7	6.450332
KLHL8	6.07364
KLHL9	6.455266
KLK1	5.692798
KLK10	10.036836
KLK11	4.808892
KLK12	3.321438
KLK13	3.678214
KLK14	4.57228
KLK15	3.883486
KLK2	3.492408
KLK3	3.826446
KLK4	3.852754
KLK5	10.9118
KLK6	10.64396
KLK7	6.327606
KLK8	6.527386
KLK9	5.472332
KLKB1	2.471082
KLKP1	3.725718
KLRAP1	2.625344
KLRAQ1	5.827408
KLRB1	2.242986
KLRC1	2.446154
KLRC2	2.4983
KLRC3	2.312174
KLRC4	2.38807
KLRD1	2.130234
KLRF1	2.048464
KLRG1	2.586568
KLRG2	4.897434
KLRK1	2.209438
KMO	3.377412
KNCN	5.288124
KNDC1	4.099786
KNG1	2.69454
KNTC1	6.892982
KPNA1	8.866934
KPNA2	11.2315
KPNA2	11.05544
KPNA3	7.15213
KPNA4	8.372928
KPNA5	4.172704

KPNA6	8.68078
KPNA7	5.678746
KPNB1	9.120262
KPRP	3.360268
KPTN	5.243934
KRAS	7.793814
KRBA1	5.21526
KRBA2	4.325312
KRCC1	5.923612
KREMEN1	4.90789
KREMEN2	6.027696
KRI1	6.623364
KRIT1	6.378198
KRR1	6.166938
KRT1	3.167958
KRT10	7.39301
KRT12	3.140168
KRT13	8.949046
KRT14	6.923174
KRT15	10.94892
KRT16	4.331574
KRT16	9.14121
KRT16P3	7.603288
KRT17	9.277452
KRT17	9.449236
KRT17	10.073342
KRT18	10.6069
KRT18	12.05286
KRT19	7.044396
KRT2	3.413674
KRT20	3.553796
KRT222	2.244428
KRT23	6.900546
KRT24	4.288306
KRT25	2.830466
KRT26	2.707434
KRT27	3.6464
KRT28	2.244458
KRT3	4.4671
KRT31	3.659222
KRT32	3.623364
KRT33A	4.85998
KRT33B	4.764878

KRT34	3.70916
KRT35	3.759152
KRT36	3.013508
KRT37	3.9091
KRT38	3.363414
KRT39	2.904662
KRT39	2.904662
KRT4	7.52032
KRT40	3.13753
KRT40	3.13753
KRT5	5.959634
KRT6A	7.931296
KRT6B	8.274408
KRT6C	9.326516
KRT7	10.321588
KRT71	4.179082
KRT72	4.419218
KRT73	3.369738
KRT74	3.423402
KRT75	3.914524
KRT76	4.275484
KRT77	4.439766
KRT78	3.587744
KRT79	4.610722
KRT8	11.93446
KRT80	6.174458
KRT81	7.05297
KRT82	3.29082
KRT83	4.084496
KRT84	4.07236
KRT85	4.114914
KRT86	6.15385
KRT9	4.23603
KRTAP10-1	5.53101
KRTAP10-10	5.443128
KRTAP10-11	4.397836
KRTAP10-12	5.764616
KRTAP10-2	6.120122
KRTAP10-3	4.076566
KRTAP10-4	5.884672
KRTAP10-5	4.139968
KRTAP10-6	5.20453
KRTAP10-7	3.481452

KRTAP10-8	4.295042
KRTAP10-9	5.270008
KRTAP1-1	6.530506
KRTAP1-1	5.47559
KRTAP1-1	5.47559
KRTAP11-1	3.063054
KRTAP12-1	3.711778
KRTAP12-2	5.023084
KRTAP12-3	5.102672
KRTAP12-4	5.708284
KRTAP1-3	5.066402
KRTAP1-3	5.066402
KRTAP13-1	2.510816
KRTAP13-2	2.668494
KRTAP13-3	3.110724
KRTAP13-4	3.724802
KRTAP1-5	4.769352
KRTAP1-5	4.769352
KRTAP15-1	2.756578
KRTAP17-1	4.948808
KRTAP19-1	3.334494
KRTAP19-2	2.805746
KRTAP19-3	2.85703
KRTAP19-4	2.482094
KRTAP19-5	3.988374
KRTAP19-6	2.259502
KRTAP19-7	2.92278
KRTAP19-8	2.98232
KRTAP20-1	2.99764
KRTAP20-2	3.399496
KRTAP20-3	2.116934
KRTAP20-4	2.634406
KRTAP2-1	6.912342
KRTAP2-1	6.883136
KRTAP21-1	2.441486
KRTAP21-2	2.599784
KRTAP22-1	3.798218
KRTAP23-1	3.683572
KRTAP2-4	5.713262
KRTAP2-4	6.18935
KRTAP2-4	6.18935
KRTAP2-4	5.713262
KRTAP24-1	2.894912

KRTAP26-1	2.675782
KRTAP27-1	2.704978
KRTAP3-1	3.250428
KRTAP3-1	3.250428
KRTAP3-2	2.90528
KRTAP3-2	2.90528
KRTAP3-3	2.409428
KRTAP3-3	2.409428
KRTAP4-1	3.471632
KRTAP4-11	5.63644
KRTAP4-11	3.61338
KRTAP4-11	4.843892
KRTAP4-12	5.27241
KRTAP4-12	5.576414
KRTAP4-12	5.27241
KRTAP4-2	5.170234
KRTAP4-3	3.81384
KRTAP4-4	4.6213
KRTAP4-5	3.154544
KRTAP4-7	5.736024
KRTAP4-7	5.646704
KRTAP4-9	5.732536
KRTAP5-1	5.859102
KRTAP5-1	6.935604
KRTAP5-10	6.147034
KRTAP5-11	5.091918
KRTAP5-2	3.91873
KRTAP5-2	5.431702
KRTAP5-3	7.204302
KRTAP5-4	3.592734
KRTAP5-5	4.072436
KRTAP5-6	3.605098
KRTAP5-7	6.32887
KRTAP5-9	4.563726
KRTAP6-1	4.191006
KRTAP6-2	3.657972
KRTAP6-3	3.989572
KRTAP7-1	2.733164
KRTAP8-1	3.775222
KRTAP9-1	3.991538
KRTAP9-2	3.921148
KRTAP9-3	3.420246
KRTAP9-4	4.340738

KRTAP9-8	4.330554
KRTCAP2	8.458984
KRTCAP3	5.208332
KRTDAP	3.804032
KSR1	5.31718
KSR2	4.38448
KTI12	7.482366
KTN1	6.367106
KU-MEL-3	3.222882
KY	3.542388
KYNU	4.353722
L1CAM	6.72084
L1TD1	2.503384
L2HGDH	6.458768
L3MBTL1	3.883866
L3MBTL2	6.864808
L3MBTL3	4.795976
L3MBTL4	2.937514
LACE1	5.078022
LACRT	3.792686
LACTB	5.099544
LACTB2	5.600062
LAD1	6.499452
LAG3	4.00988
LAGE3	6.353132
LAIR1	3.26919
LAIR2	4.106098
LALBA	2.7266
LAMA1	3.40863
LAMA2	3.496566
LAMA3	6.75813
LAMA4	8.371708
LAMA5	7.099886
LAMB1	7.438914
LAMB2	7.343898
LAMB3	8.174914
LAMB4	2.723208
LAMC1	8.910972
LAMC2	9.525366
LAMC3	4.659234
LAMP1	8.9921
LAMP2	8.050594
LAMP3	8.635838

LANCL1	6.785162
LANCL2	7.954892
LANCL3	3.63023
LAP3	8.716002
LAPTM4A	11.35596
LAPTM4B	10.51724
LAPTM5	3.706114
LARGE	4.980778
LARP1	8.832834
LARP1B	4.824702
LARP4	7.98874
LARP4B	8.297468
LARP6	6.363806
LARP7	4.378394
LARS	9.00105
LARS2	6.815926
LAS1L	6.799848
LASP1	9.6603
LASS2	8.390974
LASS3	2.994394
LASS4	5.775186
LASS5	7.128844
LASS6	7.796168
LAT	5.609758
LAT	4.475938
LAT2	4.131008
LATS1	6.193164
LATS2	6.935348
LAX1	3.320264
LAYN	2.774632
LAYN	4.137732
LBH	3.501166
LBP	2.967038
LBR	3.318148
LBR	8.53996
LBX1	5.014676
LBX2	5.07073
LCA10	4.996968
LCA5	2.742134
LCA5L	2.874232
LCAT	4.916976
LCE1A	5.44872
LCE1B	2.901846

LCE1C	4.48003
LCE1D	6.14216
LCE1E	5.686664
LCE1F	5.606042
LCE2A	5.85409
LCE2B	4.459626
LCE2C	2.648634
LCE2D	3.429774
LCE3A	4.955842
LCE3B	6.31156
LCE3C	3.600952
LCE3D	3.973458
LCE3E	3.280638
LCE4A	4.069634
LCE5A	4.14344
LCK	4.13168
LCLAT1	6.891068
LCMT1	8.112386
LCMT2	7.357598
LCN1	4.54018
LCN1	5.698288
LCN10	4.86039
LCN12	5.023458
LCN15	3.363042
LCN2	10.360836
LCN6	4.650274
LCN8	4.099586
LCN8	2.616854
LCN9	3.043922
LCNL1	4.886642
LCOR	5.957464
LCORL	4.603298
LCP1	6.837356
LCP2	3.295862
LCT	3.403394
LCTL	3.496994
LDB1	6.428198
LDB2	3.630442
LDB3	3.70934
LDHA	10.86062
LDHAL6A	2.488812
LDHAL6B	3.742524
LDHB	10.83752

LDHC	2.127898
LDHD	4.336384
LDLR	9.107814
LDLRAD1	4.371522
LDLRAD2	4.912546
LDLRAD3	7.1753
LDLRAP1	7.268856
LDOC1	3.785828
LDOC1L	6.137576
LEAP2	4.161942
LECT1	2.710846
LECT2	2.971866
LEF1	3.377562
LEFTY1	5.474028
LEFTY2	5.476018
LEKR1	2.858504
LELP1	4.323246
LEMD1	5.633616
LEMD2	7.54287
LEMD3	6.112452
LENEP	4.486482
LENG1	5.13706
LENG8	7.25301
LENG9	5.255942
LEO1	6.74339
LEP	4.08888
LEPR	4.96002
LEPRE1	5.648706
LEPREL1	8.419364
LEPREL2	4.338914
LEPREL4	5.98354
LEPROTL1	7.725384
LETM1	7.351334
LETM2	4.47251
LETMD1	8.300882
LFNG	5.162992
LGALS1	10.583486
LGALS12	3.725556
LGALS13	2.356336
LGALS14	3.076606
LGALS16	2.495858
LGALS17A	2.95045
LGALS2	3.523774

LGALS3	7.733358
LGALS3BP	10.041016
LGALS4	4.47185
LGALS7	5.478166
LGALS7	5.424968
LGALS8	8.013318
LGALS8	4.794854
LGALS9	4.055208
LGALS9B	8.00082
LGALS9C	7.745348
LGI1	2.508106
LGI2	2.982068
LGI3	4.073542
LGI4	3.663856
LGMN	9.265452
LGR4	6.047882
LGR5	2.973552
LGR6	3.307402
LGSN	2.508042
LGTN	6.203756
LHB	5.13072
LHCGR	3.316612
LHFP	5.88952
LHFPL1	2.36409
LHFPL2	6.263278
LHFPL3	3.291302
LHFPL4	5.37726
LHFPL5	4.029226
LHPP	7.435724
LHX1	5.4444
LHX2	5.509916
LHX3	5.4086
LHX4	4.057476
LHX5	4.612702
LHX6	4.27451
LHX8	3.10885
LHX9	3.713628
LIAS	5.06524
LIF	4.724504
LIF	6.12526
LIFR	2.993904
LIG1	7.265412
LIG3	6.82773

LIG4	4.767108
LILRA1	3.361328
LILRA2	2.835746
LILRA3	3.911468
LILRA4	3.27791
LILRA5	3.12357
LILRB1	3.220282
LILRB2	4.002814
LILRB3	3.802742
LILRB4	3.410672
LILRB5	3.720788
LILRP2	3.891484
LIM2	3.818782
LIMA1	8.910814
LIMCH1	6.49032
LIMD1	6.411746
LIMD2	6.112308
LIME1	5.922782
LIMK1	6.647078
LIMK2	6.619828
LIMS1	7.699432
LIMS2	4.753934
LIMS3	7.473642
LIMS3	7.473642
LIN28A	3.820828
LIN28A	3.556544
LIN28B	2.679896
LIN37	6.74233
LIN52	6.958012
LIN54	6.8203
LIN7A	2.69502
LIN7B	5.327464
LIN7C	6.23922
LIN9	4.809988
LINGO1	3.628302
LINGO2	2.820746
LINGO3	4.104984
LINGO4	5.059832
LINS	4.637468
LIPA	9.103356
LIPC	2.698256
LIPE	5.201918
LIPF	2.172082

LIPG	3.457718
LIPH	9.285274
LIPI	2.15245
LIPJ	2.57127
LIPK	2.981898
LIPM	3.07414
LIPN	2.68386
LIPT1	3.698536
LIPT2	5.00997
LITAF	9.707446
LIX1	2.843526
LIX1L	4.575842
LLGL1	6.47908
LLGL2	7.023524
LLPH	2.562334
LLPH	6.669898
LLPH	7.175938
LMAN1	7.412246
LMAN1L	3.960532
LMAN2	9.697256
LMAN2L	8.217518
LMBR1	9.242732
LMBR1L	5.07343
LMBRD1	7.199116
LMBRD2	5.953806
LMCD1	5.981194
LMF1	6.180562
LMF2	6.952952
LMLN	5.552146
LMNA	8.485984
LMNB1	9.336336
LMNB2	7.942026
LMO1	4.852584
LMO2	4.542906
LMO3	2.701796
LMO4	6.189798
LMO7	7.53994
LMOD1	3.218946
LMOD2	3.117816
LMOD3	2.558426
LMTK2	6.390128
LMTK3	4.810748
LMX1A	3.725332

LMX1B	5.030456
LNPEP	6.512162
LNX1	5.068036
LNX2	8.335362
LOC1000497	4.677152
LOC1001255	6.523238
LOC1001278	2.257626
LOC1001279	3.067408
LOC1001279	2.655822
LOC1001279	2.514276
LOC1001280	4.910624
LOC1001281	2.262114
LOC1001281	2.34932
LOC1001282	2.43985
LOC1001282	2.366394
LOC1001282	3.647598
LOC1001282	4.943904
LOC1001282	3.192622
LOC1001283	2.834294
LOC1001283	3.711158
LOC1001283	2.898278
LOC1001283	3.84853
LOC1001283	4.443068
LOC1001285	3.77578
LOC1001285	5.352384
LOC1001286	4.404396
LOC1001286	3.728858
LOC1001286	3.311712
LOC1001287	3.078644
LOC1001288	3.06349
LOC1001288	3.673236
LOC1001288	2.897936
LOC1001288	2.863152
LOC1001289	2.354268
LOC1001289	2.47485
LOC1001290	2.91731
LOC1001291	4.773482
LOC1001291	5.263486
LOC1001292	2.57422
LOC1001293	4.087028
LOC1001293	3.354566
LOC1001293	2.34698
LOC1001294	2.735204

LOC1001294	3.338064
LOC1001294	8.326708
LOC1001294	3.44996
LOC1001294	6.067394
LOC1001295	3.29689
LOC1001295	4.804898
LOC1001295	4.192284
LOC1001296	3.466148
LOC1001297	3.901308
LOC1001297	5.66016
LOC1001298	4.386132
LOC1001298	3.940024
LOC1001298	2.57284
LOC1001298	2.784606
LOC1001298	4.699548
LOC1001300	4.906986
LOC1001301	2.695962
LOC1001302	4.668344
LOC1001302	5.369836
LOC1001302	4.668344
LOC1001302	3.299372
LOC1001302	3.275272
LOC1001302	3.91972
LOC1001302	6.215894
LOC1001303	8.480496
LOC1001304	5.058468
LOC1001304	2.6448
LOC1001304	6.417824
LOC1001305	3.676434
LOC1001305	5.389158
LOC1001305	2.069468
LOC1001306	3.26761
LOC1001307	3.379992
LOC1001308	2.425558
LOC1001308	4.05816
LOC1001308	5.021088
LOC1001308	5.414574
LOC1001309	3.984176
LOC1001309	3.03603
LOC1001311	2.479504
LOC1001311	2.4558
LOC1001311	5.281532
LOC1001311	3.69137

LOC1001311	2.902086
LOC1001312	4.248354
LOC1001312	3.269668
LOC1001313	4.196956
LOC1001314	3.113966
LOC1001314	2.586688
LOC1001315	3.658518
LOC1001315	2.837378
LOC1001315	4.569714
LOC1001315	2.513398
LOC1001317	3.895472
LOC1001317	2.523794
LOC1001317	4.761368
LOC1001317	2.975144
LOC1001317	2.56896
LOC1001318	3.121364
LOC1001318	3.142512
LOC1001319	3.281754
LOC1001320	3.533718
LOC1001320	3.767064
LOC1001321	3.032026
LOC1001321	2.573302
LOC1001321	2.451638
LOC1001321	6.33801
LOC1001321	3.032026
LOC1001321	3.070084
LOC1001321	2.5024
LOC1001321	2.532616
LOC1001321	2.532616
LOC1001321	3.171304
LOC1001321	3.547036
LOC1001322	7.229042
LOC1001322	6.86582
LOC1001322	8.923064
LOC1001323	2.631212
LOC1001323	3.953162
LOC1001323	3.128952
LOC1001323	4.945152
LOC1001326	2.781118
LOC1001329	7.746152
LOC1001330	6.11614
LOC1001331	3.065602
LOC1001331	2.608918

LOC1001331	2.608918
LOC1001331	4.12555
LOC1001332	2.598414
LOC1001332	4.825284
LOC1001333	6.602338
LOC1001337	2.80223
LOC1001348	4.004902
LOC1001348	3.831502
LOC1001709	9.908154
LOC1001909	4.663784
LOC1001909	5.91298
LOC1002718	9.180732
LOC1002872	7.369584
LOC1002874	2.97416
LOC1002878	3.131682
LOC1002878	2.972016
LOC1002879	4.694456
LOC1002879	4.590462
LOC1002879	2.504
LOC1002880	6.221242
LOC1002881	5.15682
LOC1002882	2.58926
LOC1002883	4.11125
LOC1002885	5.779676
LOC1002885	4.458982
LOC1002887	6.903504
LOC1002887	5.628692
LOC1002888	2.866
LOC1002888	3.623258
LOC1002890	5.31868
LOC1002891	4.425482
LOC1002918	5.38071
LOC1002931	3.662134
LOC1002935	8.75211
LOC1002935	3.223004
LOC1004992	3.458104
LOC1005055	3.053788
LOC1005065	3.379116
LOC1005069	2.783492
LOC1005074	2.307944
LOC1005074	2.307944
LOC1005079	4.137106
LOC1005080	4.53848

LOC1005081[illegible]	6.528296
LOC1005086[illegible]	2.86422
LOC1005087[illegible]	3.099098
LOC1005090[illegible]	5.873558
LOC1005107[illegible]	3.305506
LOC113230	5.144868
LOC120824	2.542334
LOC121952	2.514802
LOC126536	3.263758
LOC128322	4.529816
LOC138412	7.987536
LOC143188	3.11338
LOC143506	3.264274
LOC143506	3.264274
LOC143506	3.204654
LOC145757	4.31796
LOC146336	6.005748
LOC146429	4.958202
LOC146880	6.327456
LOC147727	4.643252
LOC147804	9.451228
LOC148696	2.931858
LOC149134	3.183762
LOC149620	2.602142
LOC149837	4.843694
LOC149950	3.807276
LOC150197	4.27829
LOC150527	2.86148
LOC150527	2.86148
LOC150622	3.103002
LOC151009	5.855362
LOC151009	3.921628
LOC151009	6.547078
LOC151121	4.447872
LOC151174	3.452538
LOC151760	3.274424
LOC152217	6.38355
LOC152586	2.170228
LOC154872	2.804256
LOC157740	3.487804
LOC158572	3.181008
LOC158696	2.66952
LOC158960	6.155216

LOC162632	6.462518
LOC168474	4.370866
LOC1720	8.314084
LOC200261	3.268526
LOC202181	4.145886
LOC220077	4.799436
LOC220594	6.260204
LOC220594	6.665456
LOC221442	2.667138
LOC222699	5.558508
LOC254028	3.386046
LOC255411	2.269692
LOC257358	3.03937
LOC25845	7.19776
LOC283174	3.427242
LOC283314	5.543508
LOC283392	5.370406
LOC283588	2.58542
LOC283693	4.098218
LOC284009	4.511838
LOC284023	4.954174
LOC284232	3.365624
LOC284379	3.013636
LOC284412	3.2582
LOC284581	4.890798
LOC284751	4.574336
LOC284757	2.708664
LOC284788	2.236854
LOC284837	3.75021
LOC284861	4.157576
LOC285033	4.65063
LOC285456	3.026916
LOC285696	3.868952
LOC285735	2.42314
LOC286359	2.24851
LOC338579	2.257372
LOC338797	4.04684
LOC338799	5.138818
LOC339524	3.4171
LOC339524	2.80293
LOC339524	3.453306
LOC339674	5.007532
LOC339803	5.987144

LOC342541	5.706582
LOC344887	6.867704
LOC347376	3.924598
LOC347411	3.852814
LOC348840	2.54545
LOC348926	6.217294
LOC349196	5.651642
LOC349196	4.819426
LOC349196	5.216102
LOC349196	5.216102
LOC349196	4.810242
LOC349196	4.810242
LOC349196	4.572204
LOC349196	5.391508
LOC349196	4.810242
LOC349196	4.810242
LOC349196	4.572204
LOC349196	4.77143
LOC349196	4.74881
LOC349196	4.74881
LOC349196	4.656282
LOC349196	5.22803
LOC349196	5.22803
LOC349196	4.656282
LOC349196	4.77143
LOC349196	4.784512
LOC349196	4.870596
LOC349196	4.870596
LOC349196	4.870596
LOC349196	4.870596
LOC349196	4.870596
LOC349196	5.60013
LOC374491	2.807338
LOC375010	2.227022
LOC375196	3.771198
LOC375295	2.94284
LOC387646	3.25614
LOC387720	5.288502
LOC387790	5.045976
LOC388022	8.55871
LOC388152	5.993008
LOC388282	4.125466
LOC388387	3.313552

LOC388428	4.9349
LOC388630	4.126234
LOC388692	3.113322
LOC388692	3.59059
LOC388692	2.879644
LOC388796	7.48508
LOC388906	4.47069
LOC388946	3.690044
LOC389458	4.925174
LOC389493	5.674886
LOC389607	3.528744
LOC389634	6.819372
LOC389791	2.95317
LOC389834	4.509722
LOC390414	4.247282
LOC390561	3.661662
LOC390940	3.266704
LOC391169	5.429376
LOC391764	4.889552
LOC391766	4.156008
LOC392364	3.49924
LOC399744	4.969632
LOC399898	2.795152
LOC399900	3.782834
LOC399904	5.249936
LOC400464	3.259474
LOC400499	3.364458
LOC400590	5.154186
LOC400657	4.891064
LOC400707	3.131662
LOC400804	3.190028
LOC400891	3.57809
LOC400931	5.67488
LOC400940	3.0932
LOC400965	2.722486
LOC400968	3.613198
LOC400968	3.433248
LOC400986	3.141078
LOC401010	4.863856
LOC401052	4.820092
LOC401097	2.837072
LOC401097	2.061146
LOC401109	4.975012

LOC401127	5.077986
LOC401286	3.21969
LOC401286	3.21969
LOC401296	4.091896
LOC401357	4.9739
LOC401387	2.675936
LOC401397	6.125064
LOC401410	3.00685
LOC401433	4.261652
LOC401557	2.7191
LOC401589	3.172732
LOC401589	3.172732
LOC402778	6.556238
LOC404266	4.589352
LOC407835	5.77768
LOC439914	3.785426
LOC439938	3.039386
LOC440131	2.82822
LOC440173	2.934838
LOC440297	3.614526
LOC440297	3.614526
LOC440297	3.56157
LOC440297	3.614526
LOC440297	4.07087
LOC440297	3.614526
LOC440313	3.391898
LOC440337	3.036788
LOC440419	3.296568
LOC440461	6.093606
LOC440518	4.435006
LOC440563	3.362484
LOC440570	2.538258
LOC440570	2.538258
LOC440742	2.63154
LOC440792	3.120148
LOC440896	3.101088
LOC440905	3.194342
LOC440925	4.097164
LOC440944	4.003508
LOC440993	4.974198
LOC441016	4.75777
LOC441046	5.963576
LOC441177	3.978506

LOC441178	2.601858
LOC441178	2.601858
LOC441204	5.368724
LOC441208	7.25329
LOC441233	2.913796
LOC441268	8.234688
LOC441601	3.550912
LOC441666	2.000046
LOC441666	2.213222
LOC441666	1.86538
LOC441956	3.853484
LOC442132	5.129414
LOC442381	2.237344
LOC442421	4.496252
LOC442454	4.890198
LOC445341	2.880474
LOC494127	2.968462
LOC494141	4.034644
LOC494150	5.18375
LOC51145	2.81548
LOC51152	3.033884
LOC51152	2.795864
LOC554202	6.599716
LOC554206	3.421718
LOC554207	2.901348
LOC554223	6.654888
LOC554223	6.694264
LOC554249	5.176784
LOC554249	6.060566
LOC554249	5.169586
LOC554249	5.356904
LOC554249	5.176784
LOC57399	2.243612
LOC613037	7.366134
LOC613206	3.400234
LOC613266	3.47174
LOC619207	4.573966
LOC641367	3.006346
LOC642648	6.184318
LOC642864	2.864578
LOC642947	2.46897
LOC642980	3.75209
LOC642980	3.66663

LOC643008	4.401106
LOC643406	3.215494
LOC643659	3.249478
LOC643763	3.051816
LOC643797	5.952298
LOC643837	5.163006
LOC643923	2.868196
LOC644249	2.311016
LOC644249	2.697628
LOC644538	4.039758
LOC644634	3.318246
LOC644634	3.318246
LOC644714	3.308986
LOC644714	2.858516
LOC644714	3.086758
LOC644714	2.943674
LOC644936	5.476886
LOC644950	2.889702
LOC645166	4.252278
LOC645188	2.210768
LOC645212	6.963666
LOC645261	3.698954
LOC645332	5.521576
LOC645739	2.660666
LOC645877	2.160486
LOC646049	5.519682
LOC646214	3.890276
LOC646508	4.090142
LOC646513	3.719074
LOC646701	2.885126
LOC646813	6.343856
LOC646851	3.872498
LOC646853	4.102088
LOC646870	4.063416
LOC646903	3.028994
LOC646976	4.93026
LOC646982	2.792632
LOC646993	3.599772
LOC646999	3.538262
LOC648987	3.52522
LOC650157	2.470446
LOC652276	4.821474
LOC652493	2.85146

LOC652811	2.97851
LOC653075	4.811142
LOC653113	6.095268
LOC654342	7.103322
LOC654433	3.695026
LOC728024	5.929122
LOC728175	3.450988
LOC728317	3.834602
LOC728407	2.9383
LOC728437	3.561822
LOC728606	3.389498
LOC728613	6.91073
LOC728675	6.260996
LOC728690	4.686558
LOC728819	2.904216
LOC728855	3.522452
LOC729020	4.394146
LOC729040	5.669996
LOC729173	3.004616
LOC729420	3.366588
LOC729444	2.9791
LOC729461	3.088688
LOC729461	2.733698
LOC729461	3.15012
LOC729461	2.678186
LOC729461	3.56198
LOC729461	2.976392
LOC729461	3.15012
LOC729566	2.868228
LOC729603	4.135474
LOC729862	3.297052
LOC729863	2.182018
LOC729866	4.098082
LOC730441	2.505132
LOC732275	4.404452
LOC79015	3.726948
LOC80054	3.376584
LOC80154	5.73881
LOC80154	5.905006
LOC80154	5.806034
LOC80154	5.73089
LOC80154	5.502496
LOC81691	6.18284

LOC84856	4.63873
LOC84931	3.113934
LOC91316	10.31876
LOC91316	7.04552
LOC93432	2.949856
LOC93463	2.72348
LOC93622	7.406606
LOH12CR1	5.973912
LOH3CR2A	2.987748
LONP1	7.295662
LONP2	7.779932
LONRF1	4.924294
LONRF2	3.529626
LONRF3	5.895728
LOR	3.428092
LOX	3.222986
LOXHD1	3.272984
LOXL1	5.83021
LOXL2	6.39627
LOXL3	4.525432
LOXL4	3.378892
LPA	3.149768
LPAL2	3.431942
LPAR1	5.389296
LPAR2	6.330938
LPAR3	2.605572
LPAR4	2.620532
LPAR5	6.357956
LPAR6	4.564592
LPCAT1	7.185576
LPCAT2	7.38635
LPCAT3	10.31291
LPCAT4	5.98559
LPGAT1	6.124638
LPHN1	7.13214
LPHN2	3.008516
LPHN3	2.405708
LPIN1	7.0985
LPIN2	6.033784
LPIN3	4.727586
LPL	3.065634
LPO	3.855922
LPP	6.54788

LPPR1	3.299304
LPPR2	6.29481
LPPR3	4.434728
LPPR4	3.396952
LPPR5	2.35913
LPXN	6.070454
LRAT	4.871962
LRBA	7.88848
LRCH1	4.434902
LRCH2	2.42989
LRCH3	7.716504
LRCH4	5.350602
LRDD	5.244406
LRFN1	4.695642
LRFN2	3.485894
LRFN2	2.93349
LRFN3	4.940982
LRFN4	5.794436
LRFN5	3.599092
LRG1	4.692004
LRGUK	2.5219
LRIG1	4.785152
LRIG2	5.618902
LRIG3	6.13376
LRIT1	3.030318
LRIT2	2.682402
LRIT3	2.419396
LRMP	2.51917
LRP1	5.075562
LRP10	7.04944
LRP11	3.679092
LRP11	6.381528
LRP12	3.30784
LRP1B	2.544422
LRP2	2.95402
LRP2BP	2.619764
LRP3	4.909644
LRP4	3.717262
LRP5	6.350844
LRP5L	4.870486
LRP6	7.059332
LRP8	6.973992
LRPAP1	8.475452

LRPPRC	7.883716
LRRC1	7.076352
LRRC10	2.883222
LRRC14	6.68842
LRRC14B	4.235968
LRRC15	4.335732
LRRC16A	8.181362
LRRC16B	4.115114
LRRC17	3.035884
LRRC18	2.937256
LRRC19	2.256368
LRRC2	2.277472
LRRC20	5.445134
LRRC23	4.747852
LRRC24	6.03778
LRRC24	6.07628
LRRC25	4.520352
LRRC26	6.459356
LRRC27	5.383834
LRRC28	5.193598
LRRC29	4.246808
LRRC3	4.538824
LRRC31	2.809258
LRRC32	3.398966
LRRC33	4.123654
LRRC34	2.583484
LRRC36	2.773848
LRRC37A2	6.822536
LRRC37A2	6.827204
LRRC37A2	5.077516
LRRC37A3	6.701458
LRRC37A4	6.703736
LRRC37A4	6.643032
LRRC37B	4.989542
LRRC37BP1	5.898252
LRRC38	4.029894
LRRC39	2.330892
LRRC3B	3.710496
LRRC4	4.270836
LRRC40	5.021696
LRRC41	7.733204
LRRC42	7.42559
LRRC43	3.177938

LRRC45	5.26873
LRRC46	4.43752
LRRC47	7.530984
LRRC48	3.790406
LRRC49	3.762086
LRRC4B	5.355242
LRRC4C	3.05559
LRRC50	3.864764
LRRC52	3.442504
LRRC53	2.631946
LRRC55	3.529686
LRRC56	4.00687
LRRC57	6.78912
LRRC58	6.470718
LRRC59	9.856026
LRRC6	3.002154
LRRC61	7.188372
LRRC63	2.578176
LRRC66	2.452954
LRRC67	2.18524
LRRC68	5.969362
LRRC7	2.565218
LRRC70	2.468502
LRRC8A	7.280106
LRRC8B	5.370296
LRRC8C	4.202198
LRRC8D	7.730798
LRRC8E	6.05136
LRRC9	2.209204
LRRCC1	2.823656
LRRFIP1	6.016434
LRRFIP1	6.42168
LRRFIP1	5.568904
LRRFIP1	3.557084
LRRFIP1	5.024608
LRRFIP1	7.839008
LRRFIP1	6.864068
LRRFIP1	4.05014
LRRFIP1	3.233968
LRRFIP1	3.346298
LRRFIP2	6.508834
LRRIQ1	2.604372
LRRIQ3	2.131098

LRRIQ3	2.19237
LRRIQ4	2.870628
LRRK1	3.13905
LRRK2	2.325314
LRRN1	2.788022
LRRN2	4.33422
LRRN3	2.852628
LRRN4	4.294202
LRRN4CL	3.86148
LRRTM1	3.379752
LRRTM2	2.878998
LRRTM3	3.242634
LRRTM4	2.629042
LRSAM1	5.658538
LRTM1	2.960258
LRTM2	4.110118
LRTOMT	4.96797
LRWD1	5.824872
LSAMP	3.1032
LSG1	8.830248
LSM1	6.896916
LSM10	6.868632
LSM11	4.794796
LSM12	8.829642
LSM12	8.757836
LSM14A	8.726478
LSM14B	6.369838
LSM14B	5.956002
LSM14B	3.18811
LSM2	6.643
LSM2	6.643
LSM2	6.643
LSM3	8.493592
LSM4	9.622326
LSM5	6.0613
LSM6	5.280332
LSM7	6.743324
LSMD1	6.322334
LSP1	5.094282
LSP1	4.468436
LSR	8.55909
LSS	7.174066
LST1	4.883958

LST1	4.883958
LST1	4.883958
LST-3TM12	4.951254
LTA	3.638878
LTA	4.147426
LTA	3.627848
LTA4H	8.369314
LTB	6.389952
LTB	6.389952
LTB	6.389952
LTB4R	5.660732
LTB4R2	5.17929
LTBP1	4.118352
LTBP2	5.203076
LTBP3	7.462432
LTBP4	5.539672
LTBR	7.497452
LTC4S	4.932654
LTF	3.647076
LTK	3.704016
LTN1	5.500574
LTV1	7.104582
LUC7L	6.452346
LUC7L2	8.141334
LUC7L3	7.040002
LUM	2.396166
LUZP1	7.633694
LUZP2	2.815164
LUZP3P	3.387294
LUZP4	2.606704
LUZP6	8.629094
LXN	6.693502
LY6D	7.407234
LY6E	10.75142
LY6G5B	6.081832
LY6G5B	6.360398
LY6G5C	3.536526
LY6G5C	3.37839
LY6G6C	4.45171
LY6G6C	4.598312
LY6G6C	4.701844
LY6G6D	3.345548
LY6G6D	4.016676

LY6G6D	4.016676
LY6G6E	4.82721
LY6G6E	4.82721
LY6G6E	4.82721
LY6G6F	4.63145
LY6G6F	4.63145
LY6H	5.036002
LY6K	4.883804
LY75	2.684436
LY86	2.397412
LY86-AS	4.469596
LY9	2.994316
LY96	2.35983
LYAR	6.30842
LYG1	3.606
LYG2	2.986212
LYL1	5.55542
LYN	8.31898
LYNX1	7.356084
LYPD1	5.055308
LYPD2	6.778604
LYPD3	7.346216
LYPD4	3.41707
LYPD5	5.426844
LYPD6	3.214502
LYPD6B	5.171966
LYPLA1	8.257584
LYPLA2	8.66165
LYPLA2	7.49074
LYPLA2P1	8.275642
LYPLAL1	4.52702
LYRM1	5.60265
LYRM2	8.083842
LYRM2	6.557236
LYRM4	5.043702
LYRM5	5.65495
LYRM7	3.357726
LYSMD1	3.945034
LYSMD2	5.524214
LYSMD3	4.687786
LYSMD4	3.512464
LYST	5.340468
LYVE1	2.516446

LYZ	2.592338
LYZL1	2.189196
LYZL2	2.614812
LYZL4	3.36745
LYZL6	2.85894
LZIC	7.565982
LZTFL1	3.891466
LZTR1	6.208816
LZTS1	4.550746
LZTS2	5.663446
M6PR	10.7297
MAB21L1	3.158404
MAB21L2	3.428664
MACC1	6.953702
MACF1	6.982556
MACF1	4.740898
MACROD1	6.300864
MACROD2	3.398596
MAD1L1	6.281952
MAD2L1	7.400436
MAD2L1BP	7.964796
MAD2L2	7.957024
MADCAM1	5.691172
MADD	7.444344
MAEA	7.670224
MAEL	2.470004
MAF	3.44585
MAF1	8.133168
MAFA	5.424808
MAFB	5.44284
MAFF	5.596024
MAFG	6.72923
MAFG	7.29935
MAFG	5.553578
MAFIP	5.7121
MAFIP	5.817474
MAFIP	5.891378
MAFK	6.074538
MAG	3.698128
MAGEA1	3.537666
MAGEA10	3.186618
MAGEA11	2.59515
MAGEA12	3.91057

MAGEA2	4.066172
MAGEA2	3.740648
MAGEA3	3.76589
MAGEA4	3.026124
MAGEA5	3.981672
MAGEA6	4.88256
MAGEA8	5.410024
MAGEA9	4.442604
MAGEA9	4.442604
MAGEB1	2.859048
MAGEB10	2.506604
MAGEB16	2.771456
MAGEB17	4.380832
MAGEB18	2.74453
MAGEB2	2.396242
MAGEB3	3.147
MAGEB5	2.384374
MAGEB6	3.183358
MAGEC1	2.86081
MAGEC2	2.594848
MAGEC3	3.13792
MAGED1	7.299076
MAGED2	7.145284
MAGED4	3.725782
MAGED4	3.725782
MAGEE1	4.545804
MAGEE2	2.791644
MAGEF1	8.502066
MAGEH1	2.913764
MAGEL2	3.599898
MAGI1	5.419318
MAGI2	3.230386
MAGI3	5.195368
MAGIX	3.338904
MAGOH	7.253498
MAGOHB	7.93835
MAGT1	2.368136
MAGT1	8.39514
MAK	3.63607
MAK16	7.28398
MAL	9.452094
MAL2	11.4631
MALAT1	11.6188

MALAT1	6.290096
MALL	8.353172
MALT1	4.932754
MAMDC2	3.161954
MAMDC4	4.95926
MAML1	6.236836
MAML2	5.61658
MAML3	2.913402
MAMLD1	4.321796
MAMSTR	4.473432
MAN1A1	5.102466
MAN1A2	6.28723
MAN1B1	7.149992
MAN1C1	5.320796
MAN2A1	5.150884
MAN2A2	6.20905
MAN2B1	7.777994
MAN2B2	6.649624
MAN2C1	6.268606
MANBA	6.786286
MANBAL	7.522236
MANEA	5.008204
MANEAL	6.602802
MANF	8.25192
MANSC1	8.322584
MAOA	3.357584
MAOB	3.05837
MAP1A	3.43895
MAP1B	4.163764
MAP1LC3A	4.05663
MAP1LC3B	6.040012
MAP1LC3B2	7.356052
MAP1LC3C	4.62545
MAP1S	6.636836
MAP2	3.514578
MAP2K1	8.754394
MAP2K2	6.690918
MAP2K3	7.298186
MAP2K4	6.99287
MAP2K5	5.900914
MAP2K6	4.964618
MAP2K7	5.443804
MAP3K1	7.036892

MAP3K10	5.582622
MAP3K11	7.02803
MAP3K12	4.151624
MAP3K13	6.628012
MAP3K14	5.681728
MAP3K14	5.793296
MAP3K15	2.861826
MAP3K2	6.56731
MAP3K3	5.82846
MAP3K4	6.028702
MAP3K4	5.569534
MAP3K5	6.348024
MAP3K6	6.29903
MAP3K7	6.898744
MAP3K8	7.123608
MAP3K9	6.401398
MAP4	6.050446
MAP4K1	3.395454
MAP4K2	5.511644
MAP4K3	6.410058
MAP4K4	7.559986
MAP4K5	6.73286
MAP6	3.853198
MAP6D1	5.36271
MAP7	7.688888
MAP7D1	7.224408
MAP7D2	2.711178
MAP7D3	6.16135
MAP9	2.156316
MAPK1	9.838566
MAPK10	4.670058
MAPK11	4.880348
MAPK12	5.700598
MAPK13	8.346988
MAPK14	7.239948
MAPK15	5.001116
MAPK1IP1L	8.952906
MAPK3	8.491124
MAPK4	3.784118
MAPK6	6.570654
MAPK7	5.796342
MAPK8	5.759098
MAPK8IP1	5.159222

MAPK8IP1	5.264892
MAPK8IP1	5.69219
MAPK8IP2	4.265064
MAPK8IP3	5.714164
MAPK9	8.518064
MAPKAP1	8.580498
MAPKAPK2	7.180506
MAPKAPK3	7.240676
MAPKAPK5	7.788156
MAPKBP1	5.075198
MAPKSP1	6.710912
MAPRE1	10.26484
MAPRE2	5.542234
MAPRE3	5.43363
MAPT	3.904282
MAPT	3.64341
1-Mar	2.6676
10-Mar	2.988196
11-Mar	2.921114
11-Mar	3.18267
2-Mar	6.604478
3-Mar	4.05567
4-Mar	4.342222
5-Mar	7.455156
6-Mar	9.654296
7-Mar	8.009312
8-Mar	6.405868
9-Mar	4.953572
MARCKS	7.685946
MARCKSL1	9.032584
MARCO	3.930084
MARK1	2.420612
MARK2	8.546482
MARK3	7.72597
MARK4	5.79712
MARS	9.316592
MARS2	7.57689
MARVELD1	6.41208
MARVELD2	6.447672
MARVELD2	6.447672
MARVELD3	7.092838
MAS1	2.310682
MAS1L	2.738048

MAS1L	2.738048
MAS1L	2.738048
MASP1	3.262506
MASP2	3.59758
MAST1	4.070974
MAST2	6.561844
MAST3	5.931644
MAST4	5.437686
MASTL	8.841652
MAT1A	3.566578
MAT2A	10.9246
MAT2B	8.719838
MATK	3.93568
MATN1	4.214718
MATN1	3.757696
MATN2	7.317194
MATN3	2.97278
MATN4	3.913058
MATR3	7.768472
MAU2	6.497978
MAVS	7.260756
MAX	6.337748
MAZ	7.609712
MB	7.117324
MBD1	6.26257
MBD2	7.68295
MBD3	6.53977
MBD3L1	2.278374
MBD3L2	3.79925
MBD3L2	3.712486
MBD3L3	3.913226
MBD3L3	3.913226
MBD3L5	3.941304
MBD4	7.582742
MBD5	4.620442
MBD6	6.01524
MBIP	6.393646
MBL1P	2.221728
MBL2	3.49676
MBLAC1	4.699774
MBLAC2	3.644692
MBNL1	8.299568
MBNL2	9.289882

MBNL3	4.852882
MBOAT1	8.679508
MBOAT2	6.91088
MBOAT4	2.86355
MBOAT7	8.56457
MBP	4.786458
MBTD1	5.33224
MBTPS1	8.93188
MBTPS2	5.61547
MC1R	4.372132
MC2R	2.609408
MC3R	3.298462
MC4R	3.355182
MC5R	2.631582
MCAM	3.92687
MCART1	3.88318
MCART2	2.14297
MCART3P	2.500218
MCART6	4.250436
MCAT	7.77485
MCC	5.799544
MCCC1	8.956336
MCCC2	7.778118
MCCC2	7.778118
MCCD1	4.609486
MCCD1	4.412372
MCCD1	4.412372
MCEE	5.647406
MCF2	2.231516
MCF2L	5.791614
MCF2L2	3.733472
MCFD2	7.581358
MCHR1	4.058596
MCHR2	3.429392
MCL1	9.251456
MCM10	7.808874
MCM2	8.955492
MCM3	9.02746
MCM3AP	7.021646
MCM3AP-AS	4.603726
MCM4	9.636592
MCM5	9.12338
MCM6	9.054346

MCM7	9.291358
MCM8	7.949792
MCM9	5.721746
MCM9	6.294624
MCOLN1	6.7078
MCOLN2	2.63524
MCOLN3	2.646472
MCPH1	5.485116
MCRS1	6.688076
MCTP1	2.608982
MCTP2	2.67418
MCTS1	6.184638
MDC1	6.20864
MDC1	6.20864
MDFI	5.443448
MDFIC	7.063628
MDGA1	4.449068
MDGA2	2.73031
MDH1	8.66446
MDH1B	2.43272
MDH2	10.372488
MDK	8.063458
MDM1	4.293436
MDM2	7.788416
MDM4	6.352268
MDN1	6.8766
MDP1	5.563696
MDS2	3.475586
ME1	9.58797
ME2	6.230624
ME3	5.696364
MEA1	8.56374
MEAF6	7.37605
MECOM	2.590766
MECOM	3.64864
MECP2	6.374354
MECR	7.28091
MED1	8.021912
MED10	8.354076
MED11	6.528848
MED12	6.087452
MED12L	4.229166
MED13	8.233402

MED13L	6.775738
MED14	7.850548
MED15	8.019164
MED16	6.135174
MED17	7.448766
MED18	5.02605
MED19	5.00978
MED20	7.754752
MED21	7.768842
MED22	7.02068
MED23	6.668344
MED24	7.881222
MED25	6.055964
MED26	6.024944
MED27	5.959728
MED28	7.436492
MED29	7.330478
MED30	4.655544
MED31	7.164426
MED4	6.406242
MED6	8.087918
MED7	6.469102
MED8	7.176374
MED9	6.396248
MEF2A	6.447604
MEF2B	6.81474
MEF2C	2.39748
MEF2D	6.76741
MEFV	3.750938
MEG3	3.502886
MEGF10	3.09176
MEGF11	4.110162
MEGF6	5.880206
MEGF8	4.902022
MEGF8	5.676624
MEGF9	6.142952
MEI1	3.041466
MEIG1	2.211244
MEIS1	4.480836
MEIS2	5.67573
MEIS3	5.039108
MEIS3P1	6.319588
MEIS3P1	6.052572

MELK	8.779324
MEMO1	6.750368
MEMO1	6.870866
MEN1	5.824668
MEOX1	4.223378
MEOX2	4.019464
MEP1A	2.906278
MEP1B	2.433362
MEPCE	8.252922
MEPE	2.79574
MERTK	3.1739
MESDC1	6.64484
MESDC2	6.97971
MESP1	5.65645
MESP2	5.071354
MEST	7.167356
MET	7.661606
METAP1	8.760542
METAP1D	4.926988
METAP2	7.634646
METRN	5.856302
METRNL	5.830868
METT10D	7.084338
METT11D1	6.848132
METT5D1	3.10454
METT5D1	4.918914
METTL1	5.73451
METTL1	5.707662
METTL10	7.32901
METTL11A	6.982108
METTL13	8.138888
METTL14	6.118036
METTL2A	7.391988
METTL2B	4.23899
METTL3	7.19486
METTL4	7.507918
METTL5	9.003902
METTL6	7.75552
METTL7A	7.381784
METTL7B	4.487306
METTL8	4.943602
METTL9	8.182174
MEX3B	4.182332

MEX3C	6.05005
MEX3D	6.655294
MFAP1	6.843812
MFAP2	4.229146
MFAP3	8.293652
MFAP3L	3.031246
MFAP4	2.617566
MFAP5	2.60285
MFF	7.521056
MFGE8	6.440702
MFHAS1	5.780696
MFI2	8.18593
MFN1	7.417684
MFN2	8.804772
MFNG	4.934028
MFSD1	9.47452
MFSD10	7.345212
MFSD11	6.744644
MFSD2A	5.553872
MFSD2B	3.036422
MFSD2B	2.946452
MFSD3	6.352098
MFSD4	4.023368
MFSD5	6.184384
MFSD6	7.17354
MFSD6L	3.008682
MFSD7	5.241752
MFSD8	6.614826
MFSD9	5.174822
MGA	5.79346
MGAM	2.608218
MGAT1	6.982218
MGAT2	6.416056
MGAT3	6.006702
MGAT4A	4.92798
MGAT4B	7.93403
MGAT4C	2.321926
MGAT5	6.490628
MGAT5B	4.600666
MGC10814	5.050116
MGC14436	2.633446
MGC15705	3.583954
MGC16025	3.619614

MGC16121	4.969274
MGC16703	3.417368
MGC23270	3.553964
MGC23284	4.54829
MGC24103	2.40522
MGC2752	6.777518
MGC29506	3.758156
MGC34796	3.824482
MGC34800	4.71267
MGC39372	4.738992
MGC39584	2.47942
MGC42105	3.339752
MGC4294	4.079738
MGC44328	2.720464
MGC45800	3.67086
MGC45922	3.712784
MGC5590	2.627702
MGC57346	7.821046
MGC70870	3.902266
MGC72080	7.083434
MGC72080	7.108838
MGC72080	7.126686
MGC72080	7.146906
MGC72080	7.152638
MGC72080	7.188232
MGC72080	7.148644
MGC72080	7.052212
MGC72080	6.956714
MGC72080	6.90714
MGC72080	7.05869
MGC87042	2.874796
MGEA5	7.455436
MGLL	7.363442
MGMT	7.404274
MGP	9.566498
MGRN1	5.462634
MGST1	9.351892
MGST2	7.931374
MGST3	10.36506
MIA	5.2232
MIA2	2.483852
MIA3	6.08114
MIB1	6.182918

MIB2	5.496672
MICA	5.96334
MICA	5.820646
MICAL1	4.622362
MICAL2	5.287588
MICAL3	6.059592
MICAL3	7.619844
MICALCL	3.616446
MICALL1	7.171908
MICALL2	6.279874
MICB	6.451122
MICB	6.385834
MID1	4.752498
MID1IP1	6.402622
MID2	4.918332
MIDN	6.336296
MIER1	6.22011
MIER2	4.958756
MIER3	6.41475
MIF	9.56774
MIF4GD	6.211976
MIIP	6.309872
MINA	5.021982
MINA	5.757014
MINK1	6.96077
MINPP1	2.404918
MIOS	7.052278
MIOX	4.648226
MIP	3.07668
MIPEP	7.27607
MIPOL1	4.069742
MIR100	2.08317
MIR101-1	2.3059
MIR101-2	2.21314
MIR103-1	2.662574
MIR103-2	3.469676
MIR105-1	2.880688
MIR105-2	2.914602
MIR106A	2.129048
MIR106B	4.081282
MIR107	3.764492
MIR10A	2.336218
MIR10B	1.941096

MIR1-1	2.23413
MIR1-2	2.003136
MIR122	2.211956
MIR124-1	2.71866
MIR124-2	3.55987
MIR124-3	3.002388
MIR1247	5.157856
MIR125A	3.362964
MIR125B1	4.061384
MIR125B2	2.568102
MIR126	2.406596
MIR127	3.639836
MIR128-1	2.743576
MIR128-2	2.465894
MIR129-1	3.794898
MIR129-2	2.718842
MIR130A	2.929
MIR130B	3.406756
MIR132	3.75835
MIR133A1	2.286502
MIR133B	3.446688
MIR134	4.192878
MIR135A1	3.156026
MIR135A2	1.962406
MIR136	2.214128
MIR137	2.845048
MIR138-1	5.082294
MIR138-2	3.503018
MIR139	6.451878
MIR140	2.938106
MIR141	4.955302
MIR142	2.158366
MIR143	3.318204
MIR144	1.848136
MIR145	2.261604
MIR146A	2.328966
MIR147	3.827858
MIR148A	2.251696
MIR148B	3.595902
MIR149	5.446882
MIR150	4.510264
MIR152	4.736734
MIR153-1	2.329626

MIR153-2	2.314372
MIR154	2.584672
MIR155	3.095172
MIR15A	4.68928
MIR15B	3.367664
MIR16-1	2.883288
MIR16-2	2.460336
MIR17HG	4.869738
MIR181A1	2.209742
MIR181A2	2.964546
MIR181B1	2.870036
MIR181B2	3.407716
MIR181C	4.648578
MIR182	3.771774
MIR183	2.538614
MIR184	2.72515
MIR185	5.306664
MIR186	2.40272
MIR187	5.01254
MIR188	4.428248
MIR190	2.024352
MIR191	4.140688
MIR192	3.73533
MIR193A	4.74904
MIR194-1	2.551626
MIR194-2	6.748294
MIR195	3.668824
MIR196A1	3.439478
MIR196A2	3.546918
MIR197	5.242742
MIR199A1	2.397612
MIR199A2	4.516176
MIR199B	2.103608
MIR19B2	2.349792
MIR200A	2.729298
MIR200B	4.64084
MIR200C	5.904928
MIR202	2.430354
MIR203	2.920578
MIR204	2.265624
MIR205	5.479036
MIR206	2.964002
MIR208A	2.373222

MIR21	8.024928
MIR210	6.078402
MIR211	4.268498
MIR212	5.467386
MIR214	4.233578
MIR215	2.248988
MIR216A	2.90783
MIR217	2.30862
MIR218-1	2.668844
MIR218-2	3.952268
MIR219-1	4.518592
MIR219-2	3.3913
MIR221	3.13992
MIR222	3.20365
MIR223	2.573806
MIR224	4.950674
MIR23A	5.607702
MIR23B	6.72557
MIR24-1	3.23591
MIR24-2	4.19036
MIR25	6.020802
MIR26A1	3.50799
MIR26A2	2.827642
MIR26B	3.672916
MIR27A	5.6321
MIR27B	6.871824
MIR28	2.088538
MIR296	4.276668
MIR299	2.35633
MIR29A	2.561434
MIR29B1	2.093106
MIR29B2	2.662854
MIR29C	3.67326
MIR301A	2.290722
MIR302A	1.855396
MIR302B	1.969638
MIR30A	3.482198
MIR30B	2.14197
MIR30C1	2.6838
MIR30C2	3.677998
MIR30D	2.547608
MIR30E	3.188226
MIR31	3.027126

MIR32	3.013118
MIR320A	5.609564
MIR320C1	3.947306
MIR323	2.058076
MIR326	6.890952
MIR330	4.21432
MIR331	2.817088
MIR339	6.578794
MIR33A	2.434232
MIR34A	2.770358
MIR34A	6.342988
MIR34B	2.723746
MIR34C	2.235438
MIR365-1	3.835994
MIR375	4.964328
MIR376C	1.95467
MIR377	1.982772
MIR382	2.217252
MIR410	2.406078
MIR412	2.748662
MIR423	4.572018
MIR425	4.568464
MIR429	4.492168
MIR451	2.204334
MIR455	3.188164
MIR485	3.994106
MIR487A	2.251584
MIR492	3.054542
MIR494	2.692612
MIR495	2.372668
MIR503	4.282538
MIR504	4.211866
MIR509-1	2.260182
MIR516B1	2.242744
MIR516B2	2.261404
MIR517C	1.663924
MIR519A2	2.328566
MIR519A2	2.031164
MIR519A2	2.192648
MIR520C	2.099338
MIR622	12.50496
MIR7-2	2.803524
MIR7-3	3.929946

MIR9-1	2.555566
MIR9-2	1.933916
MIR92A2	2.834058
MIR93	4.318494
MIR9-3	3.945846
MIR95	2.434106
MIR96	2.4196
MIR98	2.667714
MIR99A	3.00984
MIR99B	5.659746
MIRLET7A1	3.593224
MIRLET7A2	2.790386
MIRLET7A3	3.863734
MIRLET7B	5.065942
MIRLET7C	2.388846
MIRLET7D	4.786028
MIRLET7E	3.82031
MIRLET7F1	6.044
MIRLET7F2	2.77486
MIRLET7G	3.698566
MIRLET7I	5.690778
MIS12	6.1161
MITD1	4.42252
MITF	5.335544
MIXL1	4.611072
MKI67	9.660386
MKI67IP	7.952848
MKKS	6.368894
MKL1	7.0044
MKL2	7.027936
MKLN1	6.573264
MKNK1	8.383766
MKNK2	7.232498
MKRN1	9.900328
MKRN2	8.292182
MKRN3	3.601978
MKRN9P	4.446952
MKS1	5.860988
MKX	4.613506
MLANA	3.081402
MLC1	4.221606
MLEC	8.080468
MLF1	5.301534

MLF1IP	5.961278
MLF2	10.4569
MLH1	7.930324
MLH3	4.97322
MLKL	7.243186
MLL	7.232732
MLL	4.546074
MLL2	6.114992
MLL3	7.25421
MLL4	5.501304
MLL5	6.51897
MLLT1	6.934862
MLLT10	6.77412
MLLT11	3.27563
MLLT3	2.592114
MLLT4	7.246014
MLLT6	6.524314
MLLT6	4.243532
MLN	4.602142
MLNR	5.020758
MLPH	5.82334
MLST8	6.77266
MLX	7.776812
MLXIP	6.423522
MLXIPL	3.931058
MLYCD	5.460762
MMAA	5.05023
MMAB	6.233058
MMACHC	5.603614
MMADHC	9.564422
MMD	5.564034
MMD2	4.190252
MME	6.601574
MMEL1	3.198624
MMGT1	8.311268
MMP1	2.969172
MMP10	2.715426
MMP11	4.363434
MMP12	2.182488
MMP13	2.592486
MMP14	4.07315
MMP15	4.63034
MMP16	2.3461

MMP16	2.670484
MMP17	4.713458
MMP19	4.954864
MMP19	4.766722
MMP2	3.690902
MMP20	2.795696
MMP21	3.745026
MMP23B	5.311258
MMP24	3.397446
MMP25	4.134378
MMP26	2.637282
MMP27	2.641368
MMP28	6.85566
MMP3	2.415878
MMP7	3.028452
MMP8	2.342238
MMP9	3.703222
MMRN1	2.307114
MMRN2	3.48596
MMS19	6.341478
MMS22L	6.314708
MN1	4.793872
MNAT1	5.538336
MND1	4.892052
MNDA	2.501052
MNS1	3.538998
MNT	5.599352
MNX1	5.901102
MOAP1	6.134382
MOB2	4.71669
MOBKL1A	5.126258
MOBKL1B	9.501016
MOBKL2A	6.158838
MOBKL2B	5.666446
MOBKL2C	6.114796
MOBKL3	7.88708
MOBP	3.371568
MOCOS	6.884044
MOCS1	4.987836
MOCS2	6.726158
MOCS3	6.579126
MOG	3.291302
MOG	3.327732

MOG	3.2438
MOGAT1	2.62508
MOGAT2	3.532174
MOGAT3	4.039946
MOGS	6.956546
MON1A	4.382294
MON1B	6.073668
MON2	6.968428
MOP-1	2.058538
MORC1	2.542916
MORC2	7.304388
MORC3	6.072372
MORC4	3.43528
MORC4	4.048432
MORC4	5.946574
MORF4	3.659818
MORF4L1	8.927122
MORF4L2	8.809448
MORN1	5.746458
MORN2	5.621566
MORN3	3.862198
MORN4	5.232696
MORN5	2.898566
MOS	3.514858
MOSC1	5.695946
MOSC2	4.935188
MOSPD1	6.160368
MOSPD2	3.39112
MOSPD3	7.033578
MOV10	7.566626
MOV10L1	2.97407
MOXD1	2.392236
MPDU1	9.05442
MPDZ	4.133804
MPEG1	3.908182
MPG	5.659832
MPHOSPH10	5.782696
MPHOSPH6	7.04638
MPHOSPH8	7.14537
MPHOSPH9	6.025438
MPI	6.772496
MPL	3.664684
MPND	5.064764

MPO	3.119468
MPP1	4.35309
MPP2	4.500586
MPP3	5.68366
MPP4	3.068948
MPP5	6.884924
MPP6	5.303718
MPP7	6.214092
MPPE1	6.13316
MPPED1	5.79272
MPPED2	2.839304
MPRIP	5.775266
MPST	6.59806
MPV17	8.6447
MPV17L	4.923698
MPV17L2	8.035386
MPZ	3.697092
MPZL1	8.693928
MPZL2	8.270092
MPZL3	7.006278
MR1	7.319816
MRAP	4.119516
MRAP2	2.704672
MRAS	5.057468
MRC1	2.745892
MRC1	2.745892
MRC2	4.517878
MRE11A	5.195288
MREG	7.067532
MRFAP1	10.31678
MRFAP1L1	8.122472
MRGPRD	3.187394
MRGPRE	5.161222
MRGPRF	4.374336
MRGPRG	3.908192
MRGPRX1	3.658704
MRGPRX2	3.029232
MRGPRX3	2.799824
MRGPRX4	2.621526
MRI1	6.41133
MRM1	6.13511
MRO	2.945572
MRP63	7.004808

MRPL1	6.315902
MRPL10	7.227748
MRPL11	7.854454
MRPL12	7.640746
MRPL13	3.71782
MRPL14	7.186792
MRPL15	7.630052
MRPL16	6.836006
MRPL17	7.761498
MRPL18	8.568792
MRPL19	6.393112
MRPL2	8.939782
MRPL20	8.148002
MRPL20	8.148002
MRPL21	7.449892
MRPL22	7.560466
MRPL23	6.241162
MRPL24	9.169254
MRPL27	7.68011
MRPL28	7.467404
MRPL3	9.900544
MRPL30	8.234662
MRPL32	7.366612
MRPL33	5.981104
MRPL34	6.744518
MRPL35	6.249996
MRPL36	9.24019
MRPL37	9.349812
MRPL38	6.641684
MRPL39	6.56031
MRPL4	6.733572
MRPL40	5.21446
MRPL41	7.822106
MRPL42	7.245424
MRPL43	6.175966
MRPL44	5.916182
MRPL45	10.27316
MRPL46	7.002128
MRPL47	9.372922
MRPL48	6.938304
MRPL49	8.906042
MRPL50	7.67808
MRPL51	10.5661

MRPL52	5.298678
MRPL53	8.135282
MRPL54	5.126618
MRPL55	5.944834
MRPL9	7.920788
MRPS10	9.221694
MRPS11	6.544424
MRPS12	5.977886
MRPS14	8.016224
MRPS15	9.497414
MRPS16	8.110684
MRPS17	7.086352
MRPS18A	8.006864
MRPS18B	8.043306
MRPS18B	8.043306
MRPS18B	8.043306
MRPS18C	5.243956
MRPS2	7.48085
MRPS21	8.18574
MRPS22	6.832022
MRPS23	6.675758
MRPS24	7.794226
MRPS25	7.221614
MRPS26	7.141952
MRPS27	7.157794
MRPS28	6.690454
MRPS30	7.949824
MRPS31	6.445532
MRPS33	6.038336
MRPS34	7.540278
MRPS35	8.126862
MRPS36	3.491808
MRPS5	7.51535
MRPS7	7.603828
MRPS9	6.275856
MRRF	8.149242
MRRFP1	4.519994
MRS2	7.543336
MRTO4	8.170082
MRVI1	3.560418
MS4A1	2.688148
MS4A10	3.478618
MS4A12	2.663194

MS4A13	2.0269
MS4A15	3.867704
MS4A2	2.365248
MS4A3	2.942286
MS4A4A	2.153052
MS4A5	2.480392
MS4A6A	2.798016
MS4A6E	2.311716
MS4A7	2.786916
MS4A8B	2.80704
MSC	3.132176
MSGN1	3.885034
MSH2	7.63355
MSH3	5.9468
MSH4	2.313704
MSH5	5.26873
MSH5	4.81443
MSH6	7.973432
MSI1	4.39456
MSI2	6.959776
MSL1	7.355824
MSL2	9.56124
MSL3	6.320478
MSL3L2	6.7627
MSLN	7.402142
MSLNL	6.107592
MSMB	3.033692
MSN	9.06601
MSR1	2.242516
MSRA	5.24629
MSRB2	8.269124
MSRB3	3.12594
MST1	3.26852
MST1P2	3.624496
MST1P9	3.807796
MST1R	7.164702
MST4	8.437108
MSTN	2.125146
MSTO1	6.447628
MSTO2P	6.433366
MSX1	4.889712
MSX2	5.376696
MSX2P1	5.247208

MT1A	5.538754
MT1B	4.568816
MT1DP	5.528186
MT1E	5.48938
MT1F	6.648198
MT1G	7.308422
MT1H	4.426606
MT1IP	5.097294
MT1JP	4.18367
MT1L	6.875982
MT1M	3.157948
MT1P2	4.990784
MT1P3	3.69226
MT1X	8.334574
MT2A	10.87322
MT2A	11.21632
MT2A	10.73888
MT3	5.676318
MT4	3.69956
MTA1	6.342752
MTA2	9.743582
MTA3	6.751146
MTAP	6.399806
MTBP	6.226648
MTCH1	7.338682
MTCH2	10.55444
MTCP1	5.261206
MTDH	8.84524
MTERF	4.33745
MTERFD1	5.887112
MTERFD2	4.41109
MTERFD3	4.58558
MTF1	7.018584
MTF2	7.481744
MTFMT	6.58686
MTFP1	5.414204
MTFR1	8.310902
MTG1	7.0059
MTHFD1	9.823916
MTHFD1L	6.849404
MTHFD1L	5.214196
MTHFD1L	5.417006
MTHFD1L	5.417006

MTHFD1L	5.241196
MTHFD2	9.78746
MTHFD2	9.305808
MTHFD2L	3.232008
MTHFD2L	4.25907
MTHFR	5.436034
MTHFS	6.826054
MTHFSD	6.257312
MTIF2	6.842338
MTIF3	6.524946
MTL5	5.265604
MTM1	6.284268
MTMR1	6.745732
MTMR10	4.728618
MTMR11	5.456926
MTMR12	7.8125
MTMR14	8.23161
MTMR2	8.04578
MTMR3	7.585852
MTMR3	5.789288
MTMR4	7.302334
MTMR6	6.914836
MTMR7	2.735462
MTMR8	2.634574
MTMR9	5.02962
MTMR9LP	5.2912
MTNR1A	3.32505
MTNR1B	4.108372
MTO1	7.326878
MTOR	7.534742
MTPAP	6.10497
MTR	6.413576
MTRF1	5.870498
MTRF1L	6.506514
MTRF1L	6.133368
MTRR	8.95927
MTSS1	4.002068
MTSS1L	5.108862
MTSS1L	6.0725
MTTP	2.50728
MTUS1	5.503104
MTUS2	3.71719
MTX1	6.492086

MTX2	6.990762
MTX3	5.402052
MUC1	8.87892
MUC12	4.353264
MUC12	4.135516
MUC12	2.569046
MUC13	2.33582
MUC15	2.772934
MUC16	7.366738
MUC17	2.9121
MUC19	2.8071
MUC2	3.818998
MUC20	8.923288
MUC21	3.693878
MUC21	3.619894
MUC21	4.73434
MUC3A	4.596264
MUC3A	3.521168
MUC4	4.941986
MUC5B	5.414168
MUC6	4.016356
MUC7	2.936146
MUCL1	3.204352
MUDENG	7.278132
MUL1	7.258906
MUM1	5.985648
MUM1L1	3.023588
MURC	2.977468
MUS81	6.980436
MUSK	2.746558
MUSTN1	3.430362
MUT	6.98049
MUTED	7.177798
MUTYH	6.044692
MVD	7.37269
MVK	5.872492
MVP	7.40303
MX1	10.112964
MX2	7.868824
MXD1	8.576442
MXD3	5.63899
MXD4	5.66816
MXI1	5.659112

MXRA5	3.50737
MXRA7	3.336048
MXRA7	5.081896
MXRA8	8.03945
MYADM	8.4348
MYADML	4.208564
MYADML2	4.88421
MYB	6.131752
MYBBP1A	7.158588
MYBL1	6.500198
MYBL2	8.735646
MYBPC1	2.918208
MYBPC2	3.425142
MYBPC3	4.202626
MYBPH	4.240896
MYBPHL	3.083854
MYC	8.954456
MYCBP	6.059212
MYCBP2	6.3487
MYCBPAP	3.345002
MYCL1	4.33141
MYCN	3.403646
MYCNOS	3.06847
MYCT1	2.300718
MYD88	7.553992
MYEF2	6.026024
MYEOV	7.144302
MYEOV2	4.429104
MYF5	3.184458
MYF6	3.571054
MYH1	2.186372
MYH10	6.701368
MYH11	3.609298
MYH13	2.809346
MYH14	7.68851
MYH15	3.16962
MYH16	3.592266
MYH2	2.666562
MYH3	3.217386
MYH4	2.361818
MYH6	3.399788
MYH7	3.615216
MYH7B	4.398958

MYH8	2.79732
MYH9	9.18529
MYL1	2.603614
MYL10	3.35474
MYL12A	6.705336
MYL12B	10.72776
MYL2	3.039488
MYL3	3.255218
MYL4	3.05551
MYL5	5.060774
MYL6	10.56902
MYL6B	6.47456
MYL7	3.724742
MYL9	5.460588
MYLIP	7.335878
MYLK	4.022244
MYLK2	4.004542
MYLK3	3.829542
MYLK4	3.536996
MYLPF	5.260044
MYNN	6.414174
MYO10	7.994086
MYO15A	3.7364
MYO15B	5.468368
MYO15B	3.814396
MYO15B	3.930878
MYO16	3.570184
MYO18A	7.622312
MYO18B	3.65334
MYO19	8.186234
MYO1A	3.29571
MYO1B	8.189636
MYO1C	8.941078
MYO1D	7.202628
MYO1E	8.95958
MYO1F	4.059622
MYO1G	4.047636
MYO1H	2.353056
MYO3A	2.393678
MYO3B	2.833264
MYO5A	4.710052
MYO5B	5.738606
MYO5C	7.588966

MYO6	7.100014
MYO7A	4.025494
MYO7B	3.904716
MYO9A	5.38737
MYO9B	7.044518
MYOC	2.5265
MYOCD	2.842374
MYOD1	3.400214
MYOF	9.606758
MYOG	4.462692
MYOM1	3.135182
MYOM2	2.562698
MYOM3	3.301174
MYOT	2.795458
MYOZ1	3.063288
MYOZ2	2.461036
MYOZ3	4.433096
MYPN	4.794934
MYPOP	5.81364
MYRIP	3.368164
MYSM1	6.38623
MYST1	7.944182
MYST2	8.112386
MYST3	7.007352
MYST4	5.760082
MYT1	3.688108
MYT1L	3.04367
MZF1	5.195016
MZT2A	5.61878
MZT2A	5.61878
MZT2A	7.526756
MZT2B	8.986926
MZT2B	4.854372
N4BP1	7.812552
N4BP2	4.083372
N4BP2L1	3.42986
N4BP2L2	7.301444
N4BP3	5.215372
N6AMT1	4.800292
N6AMT2	7.21362
NAA10	7.548096
NAA11	3.333732
NAA15	8.049328

NAA16	5.980066
NAA20	6.007682
NAA25	7.039596
NAA30	6.896222
NAA35	8.738552
NAA38	5.30974
NAA40	7.801654
NAA50	11.2845
NAAA	6.214666
NAALAD2	2.501884
NAALADL1	4.078398
NAALADL2	3.65588
NAB1	6.48256
NAB2	5.724838
NACA	10.075464
NACA2	3.300914
NACAD	4.503742
NACAP1	4.495104
NACC1	7.13107
NACC2	6.467952
NADK	7.188728
NADSYN1	6.81094
NAE1	7.396734
NAF1	4.356526
NAG18	2.171416
NAGA	7.98389
NAGK	8.80477
NAGLU	6.680302
NAGPA	5.140646
NAGS	5.879602
NAIF1	5.191616
NAIP	3.658002
NAIP	3.4593
NAIP	3.4593
NALCN	3.169068
NAMPT	9.573934
NAMPT	8.535458
NANOG	2.872772
NANOG	2.950562
NANOGNB	2.429222
NANOGP1	2.764592
NANOS1	5.336102
NANOS2	4.487258

NANOS3	5.580878
NANP	5.851248
NANS	9.290038
NAP1L1	9.283794
NAP1L2	2.531988
NAP1L3	2.465354
NAP1L4	7.637108
NAP1L5	4.09189
NAPA	7.963266
NAPB	4.55543
NAPEPLD	6.00959
NAPG	7.480476
NAPRT1	5.668484
NAPSA	4.268602
NAPSB	4.723054
NARF	6.04263
NARFL	4.856038
NARG2	6.646108
NARS	9.46177
NARS2	6.540614
NASP	7.683274
NAT1	2.822578
NAT10	7.683456
NAT14	6.89255
NAT15	7.295668
NAT2	2.788614
NAT8	3.167862
NAT8B	3.992734
NAT8L	4.741242
NAT9	6.361802
NAV1	6.255918
NAV2	6.327652
NAV3	5.072516
NBAS	5.932976
NBEA	5.088382
NBEAL1	6.409638
NBEAL1	5.190962
NBEAL2	5.766996
NBL1	6.905826
NBLA00301	2.66112
NBN	6.921036
NBPF1	11.4678
NBPF10	10.9469

NBPF10	8.32236
NBPF11	11.53446
NBPF11	8.676586
NBPF11	8.680406
NBPF11	9.010838
NBPF14	10.87774
NBPF14	11.58986
NBPF15	8.848052
NBPF16	8.670712
NBPF16	8.970458
NBPF16	8.959762
NBPF22P	2.948084
NBPF3	6.56083
NBPF3	10.19894
NBPF4	5.056178
NBPF4	4.758804
NBPF6	4.890906
NBPF7	3.036898
NBPF9	10.72796
NBR1	7.910932
NBR2	6.424972
NCALD	3.153508
NCAM1	3.211608
NCAM2	3.082276
NCAN	3.58652
NCAPD2	9.876406
NCAPD3	8.151724
NCAPG	8.543508
NCAPG2	9.514628
NCAPH	7.646422
NCAPH2	6.69991
NCBP1	8.770894
NCBP2	8.21797
NCBP2L	2.40649
NCCRP1	6.453462
NCDN	5.86191
NCEH1	8.65413
NCF1	4.262034
NCF1	4.311954
NCF1	4.248174
NCF2	8.604866
NCF4	3.903566
NCK1	8.719396

NCK2	7.92095
NCKAP1	8.718424
NCKAP1L	2.555842
NCKAP5	2.632664
NCKAP5L	4.931638
NCKAP5L	3.503644
NCKIPSD	4.92454
NCL	10.32666
NCLN	6.634146
NCOA1	6.180396
NCOA2	6.59693
NCOA3	7.857384
NCOA4	10.007058
NCOA5	7.063324
NCOA6	6.30727
NCOA7	7.047716
NCOR1	7.940732
NCOR2	5.45014
NCOR2	6.659514
NCR1	3.807536
NCR2	4.704576
NCR3	3.936568
NCR3	3.936568
NCR3	3.936568
NCRNA0002[illegible]	3.141136
NCRNA0002[illegible]	3.749506
NCRNA0005[illegible]	2.499116
NCRNA0008[illegible]	4.139228
NCRNA0008[illegible]	4.700186
NCRNA0008[illegible]	4.067822
NCRNA0008[illegible]	4.227696
NCRNA0009[illegible]	4.329206
NCRNA0009[illegible]	4.610078
NCRNA0011[illegible]	2.737908
NCRNA0011[illegible]	4.84226
NCRNA0011[illegible]	4.224124
NCRNA0015[illegible]	8.613568
NCRNA0015[illegible]	8.782294
NCRNA0015[illegible]	2.443232
NCRNA0016[illegible]	2.236786
NCRNA0016[illegible]	3.3481
NCRNA0016[illegible]	4.91874
NCRNA0017[illegible]	3.459872

NCRNA0017	4.57176
NCRNA0017	3.83334
NCRNA0017	5.235628
NCRNA0018	2.356112
NCRNA0018	2.17158
NCRNA0018	9.819032
NCRNA0018	2.19992
NCRNA0020	2.810986
NCRNA0020	2.829544
NCRNA0020	2.842478
NCRNA0020	4.621778
NCRNA0020	3.466876
NCRNA0020	2.661454
NCRNA0021	6.475468
NCRNA0022	2.344308
NCRNA0023	2.204112
NCRNA0024	3.039386
NCRNA0024	2.657434
NCRNA0024	3.897822
NCRNA0024	3.977762
NCRNA0024	3.680908
NCRNA0024	3.621482
NCRNA0025	3.16426
NCRNA0025	2.424138
NCRNA0026	3.232976
NCRNA0026	3.101422
NCRNA0026	4.877524
NCRNA0026	4.13387
NCRNA0026	4.794484
NCRNA0027	2.957458
NCRNA0027	8.715096
NCRNA0027	3.247202
NCRNA0028	3.472036
NCRNA0028	4.51817
NCRNA0028	4.785914
NCRNA0028	3.879304
NCRNA0029	3.847962
NCS1	6.09344
NCSTN	8.28558
ND1	11.3807
ND2	8.076782
ND4	11.71118
ND5	11.7674

ND6	10.29582
NDC80	7.425194
NDE1	7.884266
NDEL1	8.073158
NDFIP1	8.064858
NDFIP2	7.208454
NDN	2.924896
NDNL2	5.778634
NDOR1	6.066984
NDP	3.083626
NDRG1	7.643272
NDRG2	6.09698
NDRG3	7.635624
NDRG4	4.471216
NDST1	7.755864
NDST2	5.861942
NDST3	2.509664
NDST4	2.418412
NDUFA1	7.62673
NDUFA10	6.522314
NDUFA11	7.840906
NDUFA12	8.454794
NDUFA13	7.177292
NDUFA2	7.553242
NDUFA3	8.974974
NDUFA4	8.75662
NDUFA4L2	3.83364
NDUFA5	6.014836
NDUFA6	8.692252
NDUFA7	8.24157
NDUFA8	8.625706
NDUFA9	9.327312
NDUFAB1	8.639606
NDUFAF1	7.947228
NDUFAF2	5.316588
NDUFAF2	5.452962
NDUFAF3	6.058576
NDUFAF4	6.167816
NDUFB1	6.479282
NDUFB10	6.525464
NDUFB11	8.069512
NDUFB2	7.301226
NDUFB3	5.813446

NDUFB4	9.669746
NDUFB4	9.669746
NDUFB5	9.744638
NDUFB6	5.820054
NDUFB7	8.099404
NDUFB8	8.313292
NDUFB9	9.960856
NDUFC1	9.684518
NDUFC2	8.730802
NDUFS1	9.051906
NDUFS2	9.787554
NDUFS3	9.533466
NDUFS4	4.217804
NDUFS5	9.936106
NDUFS6	9.905494
NDUFS7	6.184346
NDUFS7	5.028248
NDUFS8	7.39594
NDUFV1	9.441736
NDUFV2	8.074078
NDUFV2	8.05494
NDUFV3	6.90997
NEB	2.81195
NEBL	5.767948
NECAB1	2.88637
NECAB2	3.756638
NECAB3	6.032374
NECAP1	8.944416
NECAP2	8.736542
NEDD1	7.34691
NEDD4	5.095782
NEDD4L	6.07718
NEDD8	9.510034
NEDD9	6.661098
NEFH	3.363214
NEFL	4.33789
NEFM	3.489468
NEGR1	3.345432
NEIL1	4.100812
NEIL2	6.17364
NEIL3	7.578376
NEK1	4.90084
NEK10	2.651642

NEK11	3.886178
NEK2	6.302648
NEK3	2.604202
NEK4	6.211584
NEK5	3.543482
NEK6	6.468474
NEK7	7.845388
NEK8	5.68927
NEK9	7.63976
NELF	6.252636
NELL1	2.711554
NELL2	2.952322
NENF	7.30612
NEO1	4.146004
NES	4.111398
NET1	8.733086
NETO1	2.748382
NETO2	7.094644
NEU1	7.554118
NEU1	7.580784
NEU1	7.554118
NEU2	3.42349
NEU3	5.471258
NEU4	4.707708
NEURL	6.797956
NEURL2	5.199606
NEURL3	5.89598
NEURL4	4.605816
NEUROD1	3.196104
NEUROD2	3.783984
NEUROD4	2.87549
NEUROD6	2.718444
NEUROG1	4.184912
NEUROG2	3.958534
NEUROG3	3.769384
NEXN	2.826286
NF1	7.840374
NF1	5.726194
NF1	5.726194
NF2	7.68905
NFAM1	5.490288
NFASC	3.675884
NFAT5	7.084212

NFATC1	5.237322
NFATC2	5.611248
NFATC2IP	7.883564
NFATC3	6.675026
NFATC4	4.484764
NFE2	3.889704
NFE2L1	9.083548
NFE2L2	8.059356
NFE2L3	6.81051
NFE4	6.140154
NFIA	6.89651
NFIB	7.288826
NFIC	6.966338
NFIL3	7.111756
NFIX	7.903578
NFKB1	8.702166
NFKB2	6.42662
NFKBIA	8.972298
NFKBIB	6.975714
NFKBID	4.995348
NFKBIE	6.184438
NFKBIL1	5.636536
NFKBIL1	5.636536
NFKBIL1	5.636536
NFKBIL2	6.423494
NFKBIZ	6.353662
NFRKB	7.22373
NFS1	7.588992
NFU1	5.939894
NFX1	8.018024
NFXL1	6.291576
NFYA	7.306292
NFYB	6.432404
NFYB	2.944166
NFYC	7.38828
NFYC	5.87369
NGB	4.19681
NGDN	7.16312
NGEF	4.905344
NGF	4.847502
NGFR	5.251342
NGFRAP1	5.935562
NGLY1	7.388456

NGRN	8.496386
NHEDC1	2.417628
NHEDC1	2.610056
NHEDC2	4.679094
NHEJ1	6.06865
NHLH1	3.453942
NHLH2	2.258752
NHLRC1	4.82572
NHLRC2	6.525694
NHLRC3	7.216638
NHLRC4	4.615424
NHP2	8.069802
NHP2	7.365772
NHP2L1	8.053714
NHS	3.952858
NHSL1	4.857072
NHSL2	3.217652
NICN1	5.09023
NID1	3.09056
NID2	3.29355
NIF3L1	5.946814
NIN	5.09293
NINJ1	7.083746
NINJ2	4.832782
NINL	5.510432
NIP7	9.247038
NIPA1	6.914494
NIPA2	8.95255
NIPAL1	6.475572
NIPAL2	6.77111
NIPAL3	8.048784
NIPAL4	3.858804
NIPBL	7.083954
NIPSNAP1	7.012778
NIPSNAP3A	7.077458
NIPSNAP3B	3.590364
NISCH	5.97201
NIT1	7.392992
NIT2	7.754164
NKAIN1	3.96156
NKAIN2	2.37069
NKAIN3	2.691846
NKAIN4	3.981446

NKAP	4.572252
NKAPL	2.437632
NKAPP1	3.682398
NKD1	4.442264
NKD2	4.50895
NKG7	4.831598
NKIRAS1	5.497294
NKIRAS2	7.40953
NKPD1	4.787228
NKRF	4.399762
NKTR	5.824014
NKX1-2	5.074334
NKX2-1	3.12211
NKX2-2	3.56622
NKX2-3	4.679634
NKX2-4	6.506078
NKX2-5	5.981006
NKX2-6	4.913552
NKX2-8	4.301414
NKX3-1	5.263572
NKX3-2	4.572936
NKX6-1	5.581542
NKX6-2	4.907918
NKX6-3	5.912744
NLE1	6.03073
NLGN1	2.38538
NLGN2	5.198672
NLGN3	5.013436
NLGN4X	3.158134
NLGN4Y	2.505842
NLK	6.62947
NLN	5.434492
NLRC3	3.247268
NLRC4	2.906892
NLRC5	5.879124
NLRP1	3.48641
NLRP10	3.221226
NLRP11	2.561966
NLRP12	3.46901
NLRP13	2.67536
NLRP14	2.40833
NLRP2	2.706482
NLRP3	2.848688

NLRP4	2.735454
NLRP5	2.870738
NLRP6	3.963684
NLRP7	2.568486
NLRP8	2.740594
NLRP9	2.937648
NLRX1	5.647136
NMB	4.77617
NMBR	3.31502
NMD3	8.660764
NME1	8.59515
NME3	5.155584
NME4	6.35118
NME5	2.117832
NME6	4.932436
NME7	5.302926
NMI	7.42671
NMNAT1	5.247196
NMNAT2	6.28436
NMNAT3	3.421156
NMRAL1	6.98822
NMS	3.41576
NMT1	8.806022
NMT2	5.029524
NMU	5.829748
NMUR1	4.468194
NMUR2	3.020208
NNAT	3.433246
NNMT	5.415564
NNT	8.175012
NOB1	7.868268
NOBOX	3.665146
NOC2L	8.254014
NOC3L	5.126032
NOC4L	6.08815
NOD1	4.853612
NOD2	4.525742
NODAL	2.741956
NOG	4.710064
NOL10	7.166292
NOL11	6.51076
NOL12	6.74895
NOL3	6.105776

NOL4	2.894148
NOL6	6.884718
NOL7	7.595646
NOL7	2.271922
NOL8	6.65018
NOL9	7.197312
NOLC1	8.399906
NOM1	7.558932
NOMO3	9.874474
NOMO3	9.874474
NOMO3	9.874474
NONO	9.758296
NOP10	8.00854
NOP14	7.44197
NOP16	7.20716
NOP2	8.781254
NOP56	6.916678
NOP58	8.597302
NOS1	3.613486
NOS1AP	5.817352
NOS2	3.466058
NOS3	4.181644
NOSIP	6.753792
NOSTRIN	2.50371
NOTCH1	6.065184
NOTCH2	7.045576
NOTCH2NL	7.121022
NOTCH3	7.031418
NOTCH4	5.00004
NOTUM	4.596744
NOV	6.9769
NOVA1	2.56521
NOVA2	5.363206
NOX1	2.901252
NOX3	3.02829
NOX4	2.541062
NOXA1	6.360138
NOXO1	4.72749
NPAS1	3.756834
NPAS2	7.173892
NPAS3	3.204502
NPAS4	3.43088
NPAT	5.959156

NPB	6.717988
NPB	6.717988
NPBWR1	3.964518
NPBWR2	3.308142
NPC1	8.020692
NPC1L1	4.050416
NPC2	8.88464
NPCDR1	2.686912
NPDC1	6.777684
NPEPL1	6.355598
NPEPPS	9.29624
NPEPPS	8.744128
NPFF	3.943246
NPFFR1	4.309928
NPFFR2	2.319688
NPHP1	2.619042
NPHP3	5.33797
NPHP4	4.644074
NPHS1	3.699808
NPHS2	2.709652
NPIP	8.415608
NPIP	8.446784
NPIP	8.187702
NPIPL2	7.293684
NPIPL3	8.835476
NPIPL3	8.833502
NPIPL3	9.001368
NPL	5.334596
NPLOC4	9.480284
NPM1	3.923898
NPM2	4.752016
NPM3	5.77734
NPNT	7.924908
NPPA	3.016544
NPPB	3.258774
NPPC	6.103144
NPR1	5.334968
NPR2	5.321926
NPR3	3.310542
NPRL2	5.013386
NPRL3	6.90741
NPSR1	3.140078
NPTN	7.820398

NPTX1	4.714368
NPTX2	4.153126
NPTXR	5.421136
NPVF	2.507338
NPW	5.44422
NPY	3.911924
NPY1R	2.414376
NPY2R	2.752282
NPY5R	2.375716
NPY6R	2.825272
NQO1	10.89246
NQO2	5.757384
NQO2	5.466292
NR0B1	2.651676
NR0B2	3.804132
NR1D1	6.151296
NR1D2	6.709378
NR1H2	7.750358
NR1H3	6.116442
NR1H4	2.30335
NR1I2	2.867138
NR1I3	2.914712
NR2C1	6.248818
NR2C2	7.216136
NR2C2AP	7.072792
NR2E1	2.483224
NR2E3	3.752442
NR2F1	4.898462
NR2F2	7.125008
NR2F6	8.01568
NR3C1	7.5159
NR3C2	3.026864
NR4A1	5.464264
NR4A2	5.656138
NR4A3	3.684154
NR5A1	3.91909
NR5A2	4.66179
NR6A1	6.1749
NRAP	3.17443
NRARP	5.858706
NRAS	8.460234
NRBF2	5.89792
NRBP1	8.327246

NRBP2	5.90112
NRCAM	3.172054
NRD1	8.756094
NRF1	5.762456
NRG1	4.12336
NRG1	3.663096
NRG2	5.778244
NRG3	2.945854
NRG4	6.148576
NRGN	5.786198
NRIP1	7.81128
NRIP2	4.272026
NRIP3	3.123226
NRK	2.640306
NRL	4.905368
NRM	5.736048
NRM	6.190458
NRM	6.190458
NRN1	3.8462
NRN1L	5.40289
NRP1	7.37004
NRP2	7.284028
NRSN1	3.382242
NRSN2	5.067804
NRTN	5.727218
NRXN1	3.134438
NRXN2	4.28681
NRXN3	2.903872
NS3BP	5.221044
NSA2	7.202414
NSAP11	2.95537
NSD1	7.429922
NSDHL	7.541014
NSF	8.290988
NSFL1C	9.106214
NSL1	7.180128
NSMAF	7.851456
NSMCE1	8.283384
NSMCE2	8.344576
NSMCE4A	7.405894
NSUN2	10.28244
NSUN3	6.232184
NSUN4	7.900792

NSUN5	8.272458
NSUN5P2	6.749512
NSUN5P2	6.857754
NSUN6	5.188
NSUN7	5.436904
NT5C	5.65976
NT5C1A	4.26833
NT5C1B	3.10479
NT5C2	9.589456
NT5C3	5.507044
NT5C3L	6.905694
NT5DC1	6.501602
NT5DC2	6.962268
NT5DC3	6.838472
NT5DC4	3.090788
NT5E	7.614846
NT5M	5.241436
NTAN1	6.892584
NTF3	2.705168
NTF4	5.079442
NTHL1	6.04397
NTM	3.231752
NTN1	6.357728
NTN3	4.916074
NTN4	8.65355
NTN5	4.692818
NTNG1	3.09208
NTNG2	4.2482
NTRK1	5.231698
NTRK2	2.882416
NTRK3	3.175842
NTS	2.548308
NTSR1	3.118772
NTSR2	4.462042
NUAK1	3.867642
NUAK2	4.798674
NUB1	9.00632
NUB1	3.613572
NUBP1	8.598072
NUBP2	6.522116
NUBPL	6.395708
NUCB1	8.187916
NUCB2	6.110744

NUCKS1	4.884578
NUCKS1	10.063878
NUDC	8.150006
NUDCD1	8.122358
NUDCD2	5.823752
NUDCD3	7.040938
NUDT1	6.582708
NUDT10	5.401682
NUDT11	2.782842
NUDT12	4.385808
NUDT13	3.33371
NUDT13	5.11261
NUDT14	5.725412
NUDT15	6.54048
NUDT16	6.614536
NUDT16L1	5.987206
NUDT16P1	4.381238
NUDT17	4.100416
NUDT18	4.723226
NUDT2	5.959938
NUDT21	10.48782
NUDT22	6.70596
NUDT3	7.495986
NUDT4	3.29057
NUDT4P1	8.254502
NUDT5	8.217774
NUDT6	5.448852
NUDT7	5.881514
NUDT8	5.382176
NUDT9	6.62606
NUDT9P1	2.492122
NUF2	6.976206
NUFIP1	7.65882
NUFIP2	9.131742
NUMA1	8.023088
NUMB	7.87091
NUMBL	6.71834
NUP107	8.396072
NUP133	6.92255
NUP153	8.628488
NUP155	9.578594
NUP160	8.811822
NUP188	7.968042

NUP205	8.454186
NUP210	8.838566
NUP210L	2.804462
NUP214	7.89599
NUP35	6.096304
NUP37	8.346768
NUP43	8.626016
NUP50	6.323128
NUP54	6.714054
NUP62CL	3.426996
NUP85	8.05634
NUP88	8.632698
NUP93	9.259902
NUP98	8.280062
NUPL1	8.334624
NUPL2	6.48019
NUPR1	9.599316
NUS1	8.480694
NUSAP1	8.638816
NUTF2	9.319036
NVL	6.936434
NWD1	5.431708
NXF1	9.574418
NXF2	2.599998
NXF2B	2.617118
NXF3	2.959668
NXF4	2.443608
NXF5	2.806254
NXN	4.853584
NXNL1	5.538986
NXNL2	5.249126
NXPH1	3.49879
NXPH2	3.51082
NXPH3	4.938488
NXPH4	5.496404
NXT1	6.331802
NXT2	5.569984
NYNRIN	5.21508
NYNRIN	5.208956
NYX	5.324588
OAF	7.033618
OAS1	10.487808
OAS2	9.23282

OAS3	8.311782
OASL	10.095552
OAT	10.50112
OAZ1	10.39728
OAZ2	6.988932
OAZ3	3.087368
OBFC1	5.2832
OBFC2A	6.827166
OBFC2B	8.099766
OBP2A	4.602638
OBP2B	5.919872
OBSCN	4.432172
OBSCN	4.367358
OBSL1	4.954118
OC90	3.573866
OCA2	4.265242
OCEL1	5.864378
OCIAD1	7.407704
OCIAD2	7.78908
OCLM	4.32283
OCLN	7.87336
OCM	2.99857
OCM2	2.778398
OCR1	2.976778
OCRL	7.750902
ODAM	2.502144
ODC1	9.916144
ODF1	3.139282
ODF2	7.621046
ODF2L	2.986312
ODF3	3.78225
ODF3B	5.243288
ODF3L1	3.337858
ODF3L2	4.998138
ODF4	3.094048
ODZ1	2.91861
ODZ2	3.102898
ODZ3	3.188184
ODZ4	3.736322
OFCC1	2.446726
OFD1	6.140194
OGDH	9.322362
OGDHL	4.39516

OGFOD1	8.216106
OGFOD2	5.095718
OGFR	8.081004
OGFRL1	6.07647
OGG1	5.980286
OGN	2.30859
OGT	7.742558
OIP5	6.888338
OIT3	3.21863
OK	5.065352
OK	2.367698
OLA1	7.977968
OLAH	2.806722
OLFM1	3.393978
OLFM2	5.1732
OLFM3	2.44364
OLFM4	2.713016
OLFML1	2.21362
OLFML2A	5.243796
OLFML2B	3.050236
OLFML3	3.42067
OLIG1	5.3234
OLIG2	3.73949
OLIG3	3.896182
OLR1	5.352592
OMA1	6.951198
OMD	2.126408
OMG	2.324538
OMP	5.432532
ONECUT1	5.190806
ONECUT2	4.44863
OPA1	8.126346
OPA3	5.28145
OPALIN	3.043324
OPCML	2.634528
OPHN1	5.919282
OPLAH	4.836458
OPN1LW	2.731706
OPN1MW	3.60955
OPN1MW2	3.49931
OPN1SW	3.24901
OPN3	7.697676
OPN4	4.621958

OPN5	2.883398
OPRD1	4.361476
OPRK1	3.468404
OPRL1	3.603478
OPRM1	2.872326
OPTC	3.598594
OPTN	7.221008
OR10A2	2.449018
OR10A3	3.10779
OR10A4	3.441858
OR10A5	2.491914
OR10A6	2.21714
OR10A7	2.520178
OR10AD1	2.836654
OR10AG1	2.554134
OR10C1	2.822514
OR10C1	2.897952
OR10C1	2.897952
OR10D1P	3.35096
OR10D3	2.579134
OR10D4P	2.68777
OR10G2	3.902694
OR10G3	3.488172
OR10G4	2.89843
OR10G6	3.57335
OR10G7	4.66314
OR10G8	2.933026
OR10G9	3.107694
OR10H1	3.048226
OR10H2	2.786088
OR10H3	2.837412
OR10H4	2.597478
OR10H5	4.605304
OR10J1	2.658074
OR10J3	2.55005
OR10J3	2.745676
OR10J5	2.291294
OR10K1	2.635864
OR10K2	2.423142
OR10P1	3.38998
OR10Q1	3.966592
OR10R2	2.114394
OR10R3P	2.44433

OR10S1	3.843446
OR10T2	2.655498
OR10V1	3.77175
OR10W1	2.525714
OR10X1	2.596348
OR10Z1	3.02212
OR11A1	2.131758
OR11A1	2.803124
OR11A1	2.803124
OR11G2	2.469004
OR11H1	2.71683
OR11H1	2.755296
OR11H1	2.973398
OR11H1	2.71683
OR11H4	2.427762
OR11H6	2.63204
OR11L1	2.865004
OR12D2	2.486052
OR12D2	2.471324
OR12D3	2.908044
OR12D3	2.83669
OR13A1	3.262516
OR13C2	2.009528
OR13C3	2.258502
OR13C4	2.869292
OR13C5	4.534018
OR13C8	2.519842
OR13C9	2.735074
OR13D1	2.518652
OR13F1	3.59887
OR13G1	2.786812
OR13H1	2.817468
OR13J1	3.483012
OR14A16	2.527004
OR14A2	2.32927
OR14C36	3.59969
OR14I1	3.13557
OR14J1	2.843764
OR14J1	2.843764
OR1A1	2.908296
OR1A2	2.721688
OR1B1	3.580042
OR1C1	2.726394

OR1D2	2.45314
OR1D5	3.109374
OR1D5	3.241648
OR1E1	2.806428
OR1E2	2.346348
OR1F1	3.337864
OR1F2P	3.66949
OR1G1	2.641164
OR1I1	4.16108
OR1J1	3.450294
OR1J2	2.182808
OR1J4	2.632686
OR1K1	3.384054
OR1L1	2.488916
OR1L3	2.655304
OR1L4	2.469324
OR1L6	2.12173
OR1L8	3.494332
OR1M1	3.719376
OR1N1	3.11333
OR1N2	2.51403
OR1Q1	2.906222
OR1S1	2.215002
OR1S2	2.258198
OR2A1	3.907298
OR2A1	3.707728
OR2A12	2.492858
OR2A14	2.8843
OR2A2	2.87783
OR2A20P	5.966382
OR2A25	2.310564
OR2A4	4.662178
OR2A5	2.557008
OR2A7	4.522
OR2A9P	5.959916
OR2AE1	3.412096
OR2AG1	2.863742
OR2AG2	2.994974
OR2AJ1	2.363818
OR2AK2	2.60413
OR2AP1	2.420594
OR2AT4	3.467722
OR2B11	3.29811

OR2B2	2.77509
OR2B3	2.662344
OR2B3	2.662344
OR2B3	2.662344
OR2B6	2.943318
OR2C1	3.179532
OR2C3	3.670808
OR2D2	2.72832
OR2D3	2.611594
OR2F1	3.193212
OR2F2	2.519724
OR2G2	3.097042
OR2G3	3.292158
OR2G6	2.807768
OR2H1	3.491264
OR2H2	5.08145
OR2H2	4.035698
OR2H2	4.035698
OR2J1	2.349232
OR2J2	2.711048
OR2J2	2.711048
OR2J2	2.684492
OR2J3	2.722446
OR2J3	2.768974
OR2J3	2.768974
OR2K2	2.698142
OR2L13	2.87242
OR2L2	2.78409
OR2L3	3.015158
OR2L5	2.595436
OR2L8	3.816078
OR2M1P	2.72311
OR2M2	2.170842
OR2M3	2.577426
OR2M4	2.6343
OR2M5	4.013022
OR2M7	2.575086
OR2S2	3.808794
OR2T1	2.706926
OR2T10	2.518168
OR2T11	2.91705
OR2T12	3.50891
OR2T2	2.939388

OR2T27	2.922642
OR2T29	2.567006
OR2T3	2.802314
OR2T33	3.458716
OR2T34	2.802314
OR2T35	2.936698
OR2T4	2.141176
OR2T5	2.591776
OR2T6	2.407306
OR2T8	2.417124
OR2V1	3.330898
OR2V2	3.534368
OR2W1	2.48208
OR2W1	2.48208
OR2W1	2.48208
OR2W3	2.758134
OR2W3	2.758134
OR2W5	2.996048
OR2Y1	3.475918
OR2Z1	3.423686
OR3A1	3.049044
OR3A2	3.092406
OR3A3	4.090042
OR3A4	3.664282
OR4A13P	2.441964
OR4A15	2.490228
OR4A16	2.343118
OR4A47	2.405058
OR4A47	2.345684
OR4A5	2.794486
OR4B1	2.580034
OR4C11	2.533164
OR4C12	2.258048
OR4C12	2.310344
OR4C13	2.956366
OR4C13	3.38323
OR4C15	2.388564
OR4C16	2.321504
OR4C3	2.597168
OR4C45	2.013768
OR4C46	2.600188
OR4C46	2.68456
OR4C5	2.508352

OR4C6	2.9615
OR4D1	2.89396
OR4D10	2.55361
OR4D10	3.574546
OR4D11	2.53512
OR4D2	2.49647
OR4D5	4.053276
OR4D6	2.882016
OR4D9	2.363448
OR4E2	2.926106
OR4F15	2.438764
OR4F16	2.763766
OR4F16	2.763766
OR4F16	2.763766
OR4F17	2.32306
OR4F17	2.32306
OR4F17	2.32306
OR4F21	2.746
OR4F6	2.797346
OR4H12P	2.553432
OR4K1	2.511446
OR4K13	2.65042
OR4K14	2.593612
OR4K15	2.366984
OR4K15	2.304174
OR4K17	2.361158
OR4K2	2.800294
OR4K5	2.192264
OR4L1	2.653652
OR4M1	2.718142
OR4M2	2.430134
OR4N2	2.912146
OR4N3P	2.502948
OR4N4	2.453786
OR4N5	3.03531
OR4P4	2.611666
OR4Q2	2.884748
OR4Q3	2.721748
OR4S1	2.542586
OR4S2	2.42045
OR4X1	2.954508
OR4X2	2.505278
OR51A2	2.745186

OR51A4	2.801432
OR51A7	2.315814
OR51B2	3.153702
OR51B4	3.026132
OR51B5	2.712528
OR51B6	2.410634
OR51D1	2.85639
OR51E1	3.071782
OR51E2	3.004842
OR51F1	3.047726
OR51F2	2.800572
OR51G1	2.725218
OR51G2	2.712814
OR51H1P	2.922506
OR51I1	3.064476
OR51I2	3.195802
OR51J1	2.690806
OR51L1	2.409658
OR51M1	2.9084
OR51Q1	2.540942
OR51S1	3.828236
OR51T1	2.58318
OR51V1	2.827636
OR52A1	2.586272
OR52A4	2.590466
OR52A5	2.31572
OR52B2	3.834434
OR52B4	2.886256
OR52B6	3.396912
OR52D1	3.489344
OR52E2	2.7838
OR52E4	2.34718
OR52E5	2.730024
OR52E6	2.44036
OR52E8	2.759682
OR52H1	2.902958
OR52I1	2.83719
OR52I2	2.54048
OR52J3	2.703278
OR52K1	2.894858
OR52K2	2.495626
OR52K3P	3.017964
OR52L1	2.62247

OR52L2P	2.841832
OR52M1	3.625018
OR52N1	2.852408
OR52N2	4.003332
OR52N4	2.36697
OR52N5	2.522256
OR52R1	3.695848
OR52W1	3.307204
OR56A1	2.704856
OR56A3	3.215932
OR56A4	2.952472
OR56A5	2.736892
OR56B1	2.763884
OR56B4	2.84877
OR5A1	3.54623
OR5A2	2.75104
OR5AC2	2.319016
OR5AK2	2.516496
OR5AK3P	2.30917
OR5AN1	2.506866
OR5AP2	2.415048
OR5AR1	3.459912
OR5AS1	2.495438
OR5AU1	3.17843
OR5B12	2.935868
OR5B17	2.748434
OR5B2	2.269304
OR5B21	3.193432
OR5B3	2.694456
OR5C1	4.461088
OR5D13	2.399196
OR5D14	2.314666
OR5D16	2.79265
OR5D18	2.810874
OR5D3P	2.235946
OR5E1P	2.83062
OR5F1	3.797312
OR5H1	2.262942
OR5H14	2.02249
OR5H15	2.315222
OR5H2	2.281112
OR5H6	2.457306
OR5I1	2.641984

OR5J2	2.72712
OR5K1	2.112832
OR5K2	2.338738
OR5K3	2.687504
OR5K4	2.434114
OR5L1	2.562718
OR5L2	2.11965
OR5M1	2.385092
OR5M10	2.415522
OR5M11	2.514484
OR5M3	2.201298
OR5M3	2.652786
OR5M8	2.816748
OR5M9	2.566672
OR5P2	2.325792
OR5P3	2.703048
OR5R1	2.727254
OR5T1	2.08072
OR5T2	2.44263
OR5T3	2.145172
OR5V1	2.117344
OR5W2	2.521862
OR6A2	2.174004
OR6B1	3.190212
OR6B2	2.287578
OR6B3	2.535856
OR6C1	2.262706
OR6C2	2.317284
OR6C3	2.588764
OR6C4	2.561356
OR6C6	2.576142
OR6C65	2.56567
OR6C68	2.001972
OR6C70	2.047666
OR6C74	2.359578
OR6C75	2.447878
OR6C76	2.485714
OR6F1	3.21189
OR6K2	3.305558
OR6K3	2.105454
OR6K6	3.037478
OR6M1	2.308032
OR6N1	2.704266

OR6N2	3.104942
OR6P1	2.790194
OR6Q1	2.704184
OR6S1	2.294732
OR6T1	2.975042
OR6V1	3.283728
OR6W1P	3.045232
OR6X1	3.046846
OR6Y1	3.13774
OR7A10	2.24489
OR7A17	3.48198
OR7A2P	3.104826
OR7A5	2.357754
OR7C1	2.75325
OR7C2	3.574314
OR7D2	3.419868
OR7D4	3.729852
OR7D4	2.465974
OR7E125P	4.594544
OR7E125P	4.981486
OR7E125P	5.783172
OR7E13P	2.50817
OR7E14P	7.132794
OR7E154P	5.59265
OR7E19P	3.06805
OR7E24	2.934404
OR7E35P	4.548356
OR7E5P	7.141596
OR7E87P	3.462852
OR7E87P	2.245082
OR7E87P	4.778224
OR7E87P	6.69811
OR7E91P	4.404972
OR7G1	2.896282
OR7G2	2.914234
OR7G3	2.775388
OR8A1	3.2403
OR8B12	2.57689
OR8B2	2.331506
OR8B3	2.323166
OR8B4	2.309882
OR8B8	2.648426
OR8D1	2.305256

OR8D2	2.515916
OR8D4	2.275184
OR8G2	2.626608
OR8G5	2.505698
OR8H1	2.380076
OR8H2	2.27312
OR8H3	2.315014
OR8I2	2.62917
OR8J1	2.349878
OR8J3	2.535324
OR8K1	2.494916
OR8K3	2.54458
OR8K5	2.105764
OR8S1	3.354586
OR8U1	2.63791
OR9A2	2.300302
OR9A4	2.379014
OR9G1	2.822362
OR9G4	2.894664
OR9H1P	3.036438
OR9I1	3.142184
OR9K2	2.546078
OR9Q1	2.835946
OR9Q2	2.98235
ORAI1	6.49542
ORAI2	5.688826
ORAI3	5.9608
ORAOV1	6.516748
ORC1	6.55232
ORC2	7.351368
ORC3	7.870526
ORC4	6.259788
ORC5	7.308404
ORC6	7.10112
ORM1	2.957336
ORM2	3.143146
ORMDL1	6.736418
ORMDL2	8.09205
ORMDL3	7.88489
OS9	8.725198
OSBP	8.206478
OSBP2	5.635756
OSBPL10	6.801072

OSBPL11	6.521954
OSBPL1A	6.62536
OSBPL2	7.984202
OSBPL3	6.954898
OSBPL5	5.768408
OSBPL6	3.727372
OSBPL7	5.203958
OSBPL8	5.826234
OSBPL9	8.338722
OSCAR	3.944176
OSCP1	3.688084
OSGEP	8.105576
OSGEPL1	4.402738
OSGIN1	5.697548
OSGIN2	7.068002
OSM	3.366994
OSMR	7.472786
OSR1	4.021554
OSR2	6.693856
OSTalpha	4.406814
OSTBETA	3.871728
OSTC	8.24959
OSTCL	2.94597
OSTF1	9.093712
OSTM1	5.628494
OSTN	2.961586
OTC	3.04679
OTOA	4.029588
OTOF	3.66018
OTOG	3.577418
OTOL1	2.860002
OTOP1	3.676592
OTOP2	4.281508
OTOP3	3.682826
OTOR	2.277328
OTOS	5.724032
OTP	3.009338
OTUB1	8.42571
OTUB2	4.677732
OTUD1	8.228162
OTUD3	6.127936
OTUD4	6.30224
OTUD5	6.328612

OTUD6A	3.841446
OTUD6B	7.311412
OTUD7A	4.848488
OTUD7A	4.96714
OTUD7B	6.012596
OTX1	6.03272
OTX2	3.750604
OVCH1	3.21881
OVCH2	2.725066
OVGP1	4.644428
OVOL1	6.683942
OVOL2	5.756524
OVOL3	3.482468
OVOS	4.779316
OVOS	4.839762
OVOS2	3.438722
OXA1L	9.467732
OXCT1	7.841496
OXCT2	4.887092
OXCT2	4.887092
OXER1	4.081382
OXGR1	3.101994
OXNAD1	7.29
OXR1	6.535386
OXSM	5.828542
OXSR1	9.051766
OXT	5.019542
OXTR	5.229228
P2RX1	3.197616
P2RX2	4.06
P2RX3	4.229694
P2RX4	7.134698
P2RX5	6.40389
P2RX6	4.67779
P2RX7	4.876146
P2RY1	3.84128
P2RY10	2.456244
P2RY12	2.373506
P2RY13	2.35983
P2RY14	3.008108
P2RY2	6.513716
P2RY4	3.712334
P2RY6	5.184984

P2RY8	3.264702
P2RY8	3.264702
P4HA1	6.779202
P4HA2	7.653464
P4HA2	3.543278
P4HA3	3.52568
P4HB	10.329548
P4HB	10.6891
P4HTM	6.468168
PA2G4	10.301
PAAF1	6.50467
PABPC1	11.68742
PABPC1L	5.573418
PABPC1L2A	3.324776
PABPC1L2B	4.529452
PABPC1P2	3.585032
PABPC3	6.703436
PABPC4	8.709106
PABPC5	3.209276
PABPN1	9.306256
PACRG	2.619838
PACRGL	6.152574
PACS1	6.266642
PACS2	6.303864
PACS2	5.212264
PACSIN1	4.917808
PACSIN2	8.22386
PACSIN3	7.514852
PADI1	4.541828
PADI2	8.263874
PADI3	5.052726
PADI4	3.296912
PADI6	2.885462
PAEP	5.152152
PAF1	8.932262
PAFAH1B1	8.938152
PAFAH1B2	7.97082
PAFAH1B3	7.302214
PAFAH2	6.370596
PAG1	2.798636
PAGE1	3.092658
PAGE2	2.274562
PAGE2B	3.017132

PAGE3	2.449616
PAGE4	2.49933
PAGE5	4.139652
PAH	2.869338
PAICS	9.371374
PAIP1	6.091018
PAIP1	7.936964
PAIP2	8.838808
PAIP2B	6.00768
PAK1	4.406032
PAK1	7.936614
PAK1IP1	7.249338
PAK2	7.973014
PAK3	3.11246
PAK4	7.693374
PAK6	4.830452
PAK7	3.607792
PALB2	6.805578
PALLD	7.12425
PALM	6.07369
PALM2-AKAF	4.22846
PALM3	5.827694
PALMD	4.821028
PAM	5.73156
PAM16	6.126498
PAMR1	2.797156
PAN2	6.437852
PAN3	8.030638
PANK1	5.9719
PANK2	7.677612
PANK3	7.427324
PANK4	5.877082
PANX1	7.41636
PANX2	5.77852
PANX3	2.904794
PAOX	5.203116
PAPD4	6.332002
PAPD5	5.578194
PAPD7	8.135442
PAPL	4.77404
PAPLN	4.711838
PAPOLA	7.885422
PAPOLB	2.749864

PAPOLG	7.206574
PAPPA	3.052612
PAPPA	2.630464
PAPPA2	3.153664
PAPSS1	7.530276
PAPSS2	3.288922
PAQR3	3.343264
PAQR4	6.60106
PAQR5	5.771786
PAQR6	5.647068
PAQR7	6.184234
PAQR8	2.978946
PAQR9	3.476596
PAR4	2.016062
PAR5	3.0034
PARD3	8.094436
PARD3B	3.870542
PARD6A	5.015842
PARD6B	8.156284
PARD6G	2.656382
PARG	5.9829
PARK2	3.51669
PARK7	10.25758
PARL	8.834708
PARM1	3.216824
PARN	8.764098
PARP1	8.917198
PARP10	6.644498
PARP11	3.999906
PARP12	7.643544
PARP14	8.748386
PARP15	2.29131
PARP16	5.894288
PARP2	8.282064
PARP3	6.195412
PARP4	8.63906
PARP6	8.028664
PARP8	5.099772
PARP9	9.148022
PARS2	6.782292
PART1	2.289694
PARVA	7.03037
PARVB	5.070876

PARVG	4.04393
PASD1	2.627834
PASK	4.516842
PATE1	3.395176
PATE2	2.85366
PATL1	8.929248
PATL2	4.258506
PATZ1	6.291828
PAWR	5.619784
PAX1	4.156982
PAX2	4.31432
PAX3	3.07336
PAX4	3.952444
PAX5	3.776708
PAX6	5.501872
PAX7	4.054102
PAX8	4.991898
PAX9	5.548692
PAXIP1	7.406112
PBK	7.211518
PBLD	4.26303
PBOV1	2.608084
PBRM1	6.903484
PBX1	7.51799
PBX2	5.99686
PBX2	5.946216
PBX3	5.786636
PBX4	6.331402
PBXIP1	5.358136
PC	5.78656
PCBD1	7.862946
PCBD2	5.735272
PCBP1	9.076084
PCBP2	10.061274
PCBP3	4.37432
PCBP4	5.004826
PCCA	5.258546
PCCB	8.933546
PCDH1	7.533478
PCDH10	3.486978
PCDH11X	2.879552
PCDH11Y	2.991434
PCDH12	3.45556

PCDH15	2.45285
PCDH17	3.668988
PCDH18	2.578844
PCDH19	2.632864
PCDH20	3.034072
PCDH7	5.35279
PCDH8	4.09023
PCDH9	2.389722
PCDHAC1	3.55847
PCDHB1	2.876826
PCDHB10	4.848348
PCDHB11	2.574482
PCDHB12	2.61395
PCDHB13	2.825144
PCDHB14	4.635438
PCDHB15	2.668036
PCDHB16	4.732278
PCDHB17	3.191062
PCDHB18	3.093388
PCDHB2	5.879526
PCDHB3	3.356366
PCDHB4	2.317012
PCDHB5	3.238562
PCDHB6	2.908334
PCDHB7	3.012796
PCDHB8	4.43521
PCDHB9	4.94642
PCDHGC5	3.67209
PCDP1	2.830854
PCF11	6.90213
PCGF1	6.396048
PCGF2	7.46487
PCGF3	5.97066
PCGF5	5.920562
PCGF6	6.035552
PCGF6	4.301778
PCID2	8.0522
PCID2	5.679696
PCIF1	6.400686
PCIF1	5.433386
PCK1	2.945104
PCK2	6.687284
PCLO	3.281048

PCM1	5.657744
PCMT1	8.231526
PCMTD1	2.143716
PCMTD1	7.332418
PCMTD2	3.778414
PCMTD2	3.778414
PCMTD2	7.247212
PCNA	10.169842
PCNP	8.479242
PCNP	9.20065
PCNT	6.377668
PCNX	6.632188
PCNXL2	5.385474
PCNXL3	6.723396
PCNXL3	7.907158
PCOLCE	5.2601
PCOLCE2	3.96349
PCOTH	3.523626
PCP2	5.13516
PCP4	3.598244
PCP4L1	4.885938
PCSK1	2.816108
PCSK1N	6.119476
PCSK2	3.441428
PCSK4	4.692812
PCSK5	7.267708
PCSK6	5.334104
PCSK7	6.457892
PCSK9	4.682034
PCTP	6.631786
PCYOX1	7.608114
PCYOX1L	6.001978
PCYT1A	8.482468
PCYT1B	3.453622
PCYT2	7.158562
PCYT2	7.097438
PDAP1	7.376322
PDC	2.410048
PDCD1	5.324732
PDCD10	6.862968
PDCD11	6.52779
PDCD11	4.548138
PDCD1LG2	4.489444

PDCD2	6.842158
PDCD2L	7.508354
PDCD4	8.480598
PDCD5	6.64734
PDCD6IP	8.26821
PDCD7	7.102266
PDCL	7.438182
PDCL2	2.877248
PDCL3	5.500604
PDDC1	6.337952
PDE10A	2.85727
PDE11A	2.809014
PDE12	5.50113
PDE1A	2.398938
PDE1B	3.087982
PDE1C	3.236548
PDE2A	3.178736
PDE3A	3.204634
PDE3B	2.767842
PDE4A	4.225974
PDE4B	4.57127
PDE4C	5.078922
PDE4D	3.38832
PDE4DIP	2.433208
PDE4DIP	5.054922
PDE5A	2.540264
PDE6A	2.925568
PDE6B	4.22965
PDE6C	2.503772
PDE6D	7.287774
PDE6G	3.814916
PDE6H	3.68356
PDE7A	5.043832
PDE7B	2.547774
PDE8A	5.269164
PDE8B	3.756208
PDE9A	6.13998
PDGFA	3.091
PDGFA	6.497044
PDGFB	5.180308
PDGFC	6.33331
PDGFD	2.516436
PDGFRA	2.870574

PDGFRB	3.58255
PDGFRL	3.951138
PDHA1	8.349208
PDHA2	3.412374
PDHB	7.486512
PDHX	7.856574
PDIA2	3.503966
PDIA3	10.86594
PDIA3P	10.93428
PDIA4	9.24924
PDIA5	6.228438
PDIA6	6.213534
PDIA6	11.56426
PDIK1L	6.645652
PDILT	2.89172
PDK1	6.443602
PDK2	6.917318
PDK3	5.00164
PDK4	5.007672
PDLIM1	8.502374
PDLIM2	5.226992
PDLIM3	3.434514
PDLIM4	4.810076
PDLIM5	8.074494
PDLIM7	6.833088
PDP1	8.561104
PDP2	5.58869
PDPK1	6.422896
PDPK1	5.110846
PDPK1	5.086612
PDPK1	7.688238
PDPN	3.434928
PDPR	7.64676
PDRG1	5.648098
PDS5A	7.444796
PDS5B	7.48601
PDSS1	7.208504
PDSS2	6.147518
PDX1	4.340154
PDXDC1	9.113694
PDXDC2P	7.032332
PDXDC2P	7.054612
PDXDC2P	7.050416

PDXDC2P	7.104708
PDXDC2P	6.96715
PDXK	7.870766
PDXP	6.244272
PDYN	2.468944
PDZD11	4.417174
PDZD2	6.71444
PDZD3	3.368434
PDZD4	4.668354
PDZD7	4.2034
PDZD8	6.675462
PDZD9	2.4908
PDZK1	2.46893
PDZK1IP1	7.725568
PDZRN3	3.084616
PDZRN4	2.808822
PEA15	8.7634
PEAR1	4.620272
PEBP1	8.147886
PEBP4	3.300122
PECAM1	5.818034
PECI	5.992514
PECR	5.154658
PEF1	6.63139
PEG10	9.14915
PEG3	3.545118
PELI1	6.961996
PELI2	3.486666
PELI3	4.846308
PELO	4.695296
PELP1	7.037494
PEMT	7.71286
PENK	3.86
PEPD	9.059346
PER1	3.927242
PER2	4.723232
PER3	6.740526
PERP	9.287064
PES1	7.602772
PET112L	6.498456
PEX1	6.265456
PEX10	6.155206
PEX11A	6.362314

PEX11B	6.18823
PEX11G	5.65017
PEX12	6.170374
PEX13	7.966274
PEX14	6.862188
PEX16	6.192706
PEX19	7.004366
PEX2	6.537248
PEX26	7.206564
PEX3	6.298878
PEX5	7.226304
PEX5L	2.881042
PEX6	6.626654
PEX7	5.802564
PF4	3.13674
PF4V1	3.777298
PFAS	5.695332
PFDN1	7.489204
PFDN2	7.87005
PFDN4	5.287368
PFDN5	7.138108
PFDN6	7.493172
PFDN6	7.493172
PFDN6	7.493172
PFKFB1	3.168648
PFKFB2	6.191816
PFKFB3	6.29726
PFKFB4	4.782208
PFKL	7.875922
PFKM	7.12451
PFKP	7.210242
PFN1	9.277614
PFN2	5.090642
PFN3	4.044086
PFN4	4.91227
PGA3	3.20845
PGA4	3.136742
PGA5	3.184636
PGAM1	8.770634
PGAM1	8.832756
PGAM1	8.934164
PGAM2	5.213532
PGAM4	8.574112

PGAM4	8.574112
PGAM5	7.509304
PGAP1	6.009582
PGAP2	7.277532
PGAP3	5.825604
PGBD1	3.417362
PGBD2	4.943334
PGBD4	5.012498
PGBD5	2.977292
PGC	7.042242
PGCP	2.76423
PGD	10.66832
PGF	4.09215
PGGT1B	6.796482
PGK1	9.642984
PGK2	2.566652
PGLS	6.533764
PGLYRP1	4.419636
PGLYRP2	2.808916
PGLYRP3	4.079058
PGLYRP4	4.116714
PGM1	9.286868
PGM2	8.112676
PGM2L1	3.403392
PGM3	7.61955
PGM5	6.136326
PGM5	2.599416
PGM5P2	5.612902
PGM5P2	5.675268
PGP	7.069758
PGPEP1	5.805812
PGPEP1L	4.315904
PGR	2.736806
PGRMC1	8.600642
PGRMC2	6.296382
PGS1	6.25588
PHACTR1	3.267044
PHACTR2	4.860998
PHACTR3	4.1973
PHACTR4	8.036486
PHAX	6.709416
PHB	9.852886
PHB2	9.918994

PHC1	5.550872
PHC1	5.51246
PHC2	5.794728
PHC3	8.22471
PHEX	4.245794
PHF1	5.97948
PHF10	5.335864
PHF10	6.335272
PHF11	6.689908
PHF12	7.18704
PHF13	6.502884
PHF14	7.427956
PHF15	7.512836
PHF16	5.145804
PHF17	6.043724
PHF19	7.727878
PHF2	6.633262
PHF20	6.567488
PHF20L1	6.434286
PHF21A	5.784022
PHF21B	5.082216
PHF23	6.775002
PHF3	7.073878
PHF5A	8.307508
PHF6	7.578744
PHF7	4.486144
PHF8	5.181466
PHGDH	8.965202
PHIP	6.770818
PHKA1	5.943476
PHKA2	5.677418
PHKB	7.6303
PHKG1	3.646494
PHKG2	8.035956
PHLDA1	9.103932
PHLDA2	7.109764
PHLDA3	6.792372
PHLDB1	5.11738
PHLDB2	6.917242
PHLDB2	3.447724
PHLDB3	5.657956
PHLDB3	5.133574
PHLPP1	5.679652

PHLPP2	6.053884
PHOSPHO1	3.488962
PHOX2A	3.594444
PHOX2B	3.044858
PHPT1	6.97653
PHRF1	6.323848
PHTF1	5.03887
PHTF2	5.917598
PHYH	6.237426
PHYHD1	4.13338
PHYHIP	3.78674
PHYHIPL	2.690756
PI15	2.430652
PI16	4.541368
PI3	9.472954
PI4K2A	7.51685
PI4K2B	6.942282
PI4KA	7.551202
PI4KAP2	7.594962
PI4KAP2	7.837032
PI4KB	7.10337
PIAS1	8.32227
PIAS2	6.040796
PIAS3	5.989018
PIAS4	7.183296
PIBF1	4.143198
PICALM	8.738768
PICK1	5.656814
PID1	3.424686
PID1	9.417554
PIF1	5.597774
PIGA	6.002556
PIGB	6.635046
PIGC	6.50956
PIGF	5.537712
PIGG	6.285346
PIGH	3.953202
PIGK	4.371476
PIGL	7.06772
PIGM	6.391304
PIGN	4.627646
PIGO	6.825768
PIGP	4.955194

PIGQ	5.714742
PIGR	3.821502
PIGS	7.878576
PIGT	7.20816
PIGU	8.156682
PIGV	7.692912
PIGW	7.512118
PIGX	8.352212
PIGY	8.759164
PIGZ	3.38659
PIH1D1	6.60304
PIH1D2	3.179828
PIK3AP1	4.44925
PIK3C2A	5.435492
PIK3C2B	6.123254
PIK3C2G	2.661024
PIK3C3	5.389738
PIK3CA	6.484464
PIK3CB	8.344008
PIK3CD	5.19843
PIK3CG	2.782214
PIK3IP1	3.965398
PIK3R1	6.138982
PIK3R2	6.40855
PIK3R3	8.787692
PIK3R4	7.602742
PIK3R5	4.05908
PIK3R6	3.672824
PIKFYVE	6.098678
PILRA	4.486858
PILRB	6.61025
PIM1	7.77723
PIM2	5.110486
PIM3	7.54341
PIN1	7.555126
PIN1P1	6.252376
PIN4	3.552864
PINK1	6.55197
PINK1	5.157248
PINK1	5.25744
PINX1	4.28192
PION	5.72809
PION	6.418388

PIP	2.584892
PIP4K2A	6.992902
PIP4K2B	7.573992
PIP4K2C	8.23572
PIP5K1A	8.286078
PIP5K1B	2.478668
PIP5K1C	5.391608
PIP5K1P1	2.831892
PIP5KL1	6.031782
PIPOX	4.530988
PIPSL	4.565382
PIR	7.718806
PIRT	3.557044
PISD	6.89857
PITPNA	8.721192
PITPNB	9.457912
PITPNC1	4.448512
PITPNM1	6.185572
PITPNM2	4.827386
PITPNM3	6.145952
PITRM1	8.629992
PITX1	7.826452
PITX2	3.712318
PITX3	4.357212
PIWIL1	3.009108
PIWIL2	2.351868
PIWIL3	3.094932
PIWIL4	3.474542
PJA1	5.70726
PJA2	7.241636
PK155	4.711978
PKD1	5.51721
PKD1	8.084586
PKD1L1	3.303466
PKD1L2	4.494752
PKD1L3	2.699476
PKD1P1	8.223396
PKD1P1	8.144784
PKD1P1	8.41125
PKD1P1	8.392542
PKD1P1	8.23161
PKD2	6.831002
PKD2L1	3.172324

PKD2L2	2.457296
PKDCC	5.116166
PKDREJ	2.882028
PKHD1	2.732276
PKHD1L1	2.449658
PKIA	2.413388
PKIB	3.793026
PKIG	4.154222
PKLR	4.345726
PKM2	10.85128
PKMYT1	6.210934
PKN1	8.488348
PKN2	6.95445
PKN3	6.69263
PKNOX1	6.045162
PKNOX2	3.829868
PKP1	4.198248
PKP2	8.164768
PKP3	6.322096
PKP4	7.28201
PLA1A	3.396654
PLA2G10	4.172536
PLA2G12A	5.922044
PLA2G12B	4.28097
PLA2G15	5.750354
PLA2G16	8.578688
PLA2G1B	3.43595
PLA2G2A	3.802386
PLA2G2C	4.328134
PLA2G2D	4.87827
PLA2G2E	4.243868
PLA2G2F	4.402682
PLA2G3	4.318086
PLA2G4A	2.297922
PLA2G4C	3.973846
PLA2G4D	4.30894
PLA2G4E	3.511606
PLA2G4F	4.378806
PLA2G5	3.753136
PLA2G6	3.80655
PLA2G7	7.233666
PLA2R1	5.050502
PLAA	7.641972

PLAC1	5.612378
PLAC1L	2.278088
PLAC2	5.807616
PLAC4	2.677474
PLAC8	8.972914
PLAC8L1	3.386594
PLAC9	4.303042
PLAG1	2.589026
PLAGL1	4.541154
PLAGL2	5.488118
PLAT	6.101666
PLAU	6.666556
PLAUR	8.470722
PLB1	3.311794
PLBD1	8.914336
PLBD2	5.933632
PLCB1	2.781286
PLCB2	3.452592
PLCB3	7.642384
PLCB4	3.394206
PLCD1	5.204622
PLCD3	6.959502
PLCD4	3.257228
PLCE1	4.785358
PLCG1	6.0076
PLCG2	6.806942
PLCH1	3.220068
PLCH2	4.466332
PLCL1	2.885066
PLCL2	5.046694
PLCXD1	5.677192
PLCXD1	5.819244
PLCXD2	4.168028
PLCXD3	4.667116
PLCZ1	2.548704
PLD1	5.603142
PLD2	6.268712
PLD3	7.574196
PLD4	4.717948
PLD5	3.240944
PLD6	5.446506
PLDN	6.912928
PLEC	8.142354

PLEK	2.436674
PLEK2	8.126518
PLEKHA1	7.784522
PLEKHA2	6.999508
PLEKHA3	6.333264
PLEKHA4	6.131632
PLEKHA5	4.6141
PLEKHA6	7.020984
PLEKHA7	5.976498
PLEKHA8	6.180538
PLEKHA9	6.095082
PLEKHB1	6.117308
PLEKHB2	9.861992
PLEKHF1	6.636932
PLEKHF2	7.064304
PLEKHG1	4.133806
PLEKHG2	5.962444
PLEKHG3	5.437258
PLEKHG4	4.323814
PLEKHG4B	4.54337
PLEKHG5	4.961484
PLEKHG6	6.216412
PLEKHG7	2.39929
PLEKHH1	3.977502
PLEKHH2	2.768078
PLEKHH3	5.782276
PLEKHJ1	6.312168
PLEKHM1	6.379848
PLEKHM1P	7.909412
PLEKHM2	7.096934
PLEKHM3	5.159184
PLEKHM3	6.086916
PLEKHN1	5.84513
PLEKHO1	3.459072
PLEKHO2	5.191672
PLG	3.215438
PLGLA	2.801012
PLGLB2	3.096458
PLGLB2	3.096458
PLIN1	4.21263
PLIN2	3.116698
PLIN3	7.680666
PLIN4	4.417542

PLK1	9.619368
PLK1S1	4.296468
PLK2	9.462352
PLK3	5.867362
PLK4	6.584002
PLK5P	5.275206
PLLP	6.024328
PLN	2.059824
PLOD1	7.76978
PLOD2	7.705484
PLOD3	7.318288
PLP1	3.249036
PLP2	9.299592
PLRG1	10.192642
PLS1	7.529496
PLS3	8.329464
PLSCR1	8.82582
PLSCR2	2.683472
PLSCR3	5.619294
PLSCR4	2.58115
PLTP	3.863846
PLUNC	3.171216
PLVAP	4.691636
PLXDC1	3.638546
PLXDC2	3.712672
PLXNA1	7.467934
PLXNA2	5.891718
PLXNA3	5.524048
PLXNA4	3.809866
PLXNB1	5.209752
PLXNB2	8.20854
PLXNB3	5.081338
PLXNC1	2.737474
PLXND1	6.128462
PM20D1	3.09823
PM20D2	7.668574
PMAIP1	6.80217
PMCH	2.197822
PMCHL1	2.249258
PMCHL2	2.320354
PMCHL2	2.325302
PMEPA1	6.489174
PMF1	8.966548

PMFBP1	2.88573
PML	6.059022
PMM1	6.153652
PMM2	7.07457
PMP2	2.610632
PMP22	7.374272
PMPCA	8.28041
PMPCB	8.120794
PMS1	6.174916
PMS2	8.178122
PMS2CL	5.805716
PMS2L2	9.430136
PMS2L2	9.491962
PMS2L2	9.431134
PMS2P1	8.024168
PMS2P11	8.992186
PMS2P3	7.393116
PMS2P4	10.02509
PMS2P5	8.59577
PMS2P5	8.787852
PMS2P5	9.333268
PMS2P5	9.494962
PMVK	6.499372
PNCK	4.911544
PNKD	5.64563
PNKP	6.270186
PNLDC1	3.175032
PNLIP	2.402806
PNLIPRP1	3.413654
PNLIPRP2	2.99181
PNLIPRP3	2.285954
PNMA1	7.52577
PNMA2	3.270722
PNMA3	3.870986
PNMA5	3.963272
PNMA6A	3.824652
PNMA6A	4.587236
PNMAL1	3.77633
PNMT	4.003194
PNN	8.388972
PNO1	7.341712
PNO1	3.744448
PNOC	3.843678

PNP	9.818262
PNPLA1	3.752132
PNPLA2	6.97103
PNPLA3	5.751062
PNPLA4	6.725788
PNPLA5	3.990356
PNPLA6	6.250714
PNPLA7	4.194856
PNPLA8	5.474032
PNPO	8.294922
PNPT1	8.445364
PNRC1	8.473346
PNRC2	9.711694
PNRC2	9.711694
POC1A	5.542298
POC1B	5.544386
POC5	5.781264
PODN	3.910898
PODNL1	5.21188
PODXL	8.713578
PODXL2	4.970256
POF1B	2.476958
POFUT1	7.126154
POFUT2	5.465824
POGK	8.245658
POGLUT1	7.396126
POGZ	5.83034
POLA1	6.68021
POLA2	8.474978
POLB	7.097218
POLD1	7.1106
POLD2	7.978094
POLD3	7.803926
POLD4	7.720198
POLDIP2	9.211594
POLDIP3	8.50131
POLE	6.913084
POLE2	5.977472
POLE3	7.63613
POLE4	8.14411
POLG	5.72338
POLG2	5.450192
POLH	7.69079

POLI	4.157912
POLK	4.387468
POLL	4.921976
POLM	5.058382
POLN	4.03294
POLQ	6.126554
POLR1A	7.72541
POLR1B	7.299808
POLR1C	8.29253
POLR1D	7.929466
POLR1E	7.317674
POLR2A	7.638186
POLR2B	9.737944
POLR2C	7.325118
POLR2D	8.117748
POLR2E	8.065986
POLR2F	8.357742
POLR2G	7.96621
POLR2H	9.485946
POLR2I	6.83804
POLR2J	7.215658
POLR2J2	7.849188
POLR2J3	7.940022
POLR2J3	7.819982
POLR2J4	7.941786
POLR2K	9.836022
POLR2L	7.983958
POLR3A	6.276974
POLR3B	5.882798
POLR3C	7.638382
POLR3D	5.955746
POLR3E	7.340682
POLR3F	4.688008
POLR3G	3.907166
POLR3GL	3.874066
POLR3H	6.783528
POLR3K	8.481026
POLRMT	6.639138
POM121	7.309788
POM121	5.911248
POM121	7.215682
POM121C	6.762222
POM121L10I	3.972882

POM121L12	3.403348
POM121L1P	3.89181
POM121L1P	3.89181
POM121L1P	3.868972
POM121L1P	3.930836
POM121L2	2.611068
POM121L4P	3.54609
POM121L8P	4.684552
POM121L8P	4.544704
POM121L9P	4.713172
POMC	4.081586
POMGNT1	7.08962
POMP	9.02669
POMT1	7.272004
POMT2	5.800376
POMZP3	5.983552
PON1	2.480206
PON2	8.356164
PON3	3.706664
POP1	7.511508
POP4	7.755544
POP5	6.856826
POP7	7.361906
POPDC2	4.003594
POPDC3	6.165802
POR	8.038532
PORCN	4.476346
POSTN	2.547228
POT1	5.421144
POTEA	2.272232
POTEB	2.458582
POTEC	3.004948
POTED	2.316652
POTEE	6.892224
POTEE	2.465232
POTEE	11.0653
POTEE	2.488784
POTEF	11.43298
POTEF	2.390306
POTEG	2.479928
POTEKP	8.470902
POTEM	2.726192
POTEM	9.39424

POTEM	2.724362
POU1F1	2.825052
POU2AF1	3.891546
POU2F1	6.442016
POU2F2	4.779714
POU2F3	5.325594
POU3F1	5.451614
POU3F2	4.873478
POU3F3	6.512526
POU3F4	4.590302
POU4F1	5.344522
POU4F2	3.848174
POU4F3	4.440872
POU5F1	4.521254
POU5F1	4.750616
POU5F1	4.521254
POU5F1B	4.150436
POU5F1P3	4.557398
POU5F1P4	4.020114
POU5F2	4.52757
POU6F1	5.007536
POU6F2	3.277274
PP13004	3.250566
PP13439	4.012696
PP14571	4.104054
PP2672	2.407152
PPA1	7.111828
PPA2	4.29065
PPAN-P2RY1	6.484518
PPAP2A	5.188598
PPAP2B	5.73756
PPAP2C	8.79026
PPAPDC1A	3.321964
PPAPDC1B	6.792096
PPAPDC2	5.506206
PPAPDC3	5.891076
PPARA	5.819428
PPARD	5.841208
PPARG	7.25435
PPARGC1A	2.74378
PPARGC1B	5.737586
PPAT	6.929324
PPBP	3.213938

PPBPL2	3.01374
PPCDC	5.100582
PPCS	7.89484
PPDPF	8.175326
PPEF1	2.611192
PPEF2	3.171498
PPFIA1	7.931434
PPFIA2	2.94471
PPFIA3	3.877936
PPFIA4	3.820628
PPFIBP1	6.765526
PPFIBP2	4.389128
PPHLN1	7.906858
PPIA	11.717
PPIA	8.121908
PPIAL4A	5.18716
PPIAL4A	5.18716
PPIAL4A	3.511604
PPIAL4A	3.511604
PPIAL4A	3.44168
PPIAL4A	11.9337
PPIAL4G	4.345376
PPIB	9.525356
PPIC	7.152992
PPID	6.38292
PPIE	7.533814
PPIEL	3.459636
PPIF	8.326568
PPIG	5.339454
PPIH	8.603018
PPIL1	6.869444
PPIL2	7.344546
PPIL3	7.594044
PPIL4	5.792846
PPIL5	6.039268
PPIL5	6.022444
PPIL6	4.660834
PPIP5K1	6.933948
PPIP5K2	4.76649
PPL	6.718106
PPM1A	5.647416
PPM1B	7.100634
PPM1D	6.91849

PPM1E	3.308846
PPM1F	5.791264
PPM1G	7.931414
PPM1H	6.39597
PPM1J	4.582008
PPM1K	5.831712
PPM1L	3.834192
PPM1M	4.14248
PPM1N	5.354926
PPME1	7.946012
PPOX	5.474094
PPP1CA	9.919048
PPP1CB	9.166838
PPP1CC	10.48644
PPP1R10	7.637374
PPP1R10	7.637374
PPP1R10	7.637374
PPP1R11	7.01432
PPP1R11	7.01432
PPP1R12A	6.134768
PPP1R12B	4.975442
PPP1R12C	6.627438
PPP1R13B	6.492826
PPP1R13L	6.310994
PPP1R14A	4.976488
PPP1R14B	8.714932
PPP1R14C	6.776694
PPP1R14D	4.45914
PPP1R15A	7.310972
PPP1R15B	8.546182
PPP1R16A	6.3895
PPP1R16A	3.85381
PPP1R16B	4.46951
PPP1R1A	4.10612
PPP1R1B	5.778668
PPP1R1C	4.369914
PPP1R2	7.699996
PPP1R2P1	3.656192
PPP1R2P1	3.656192
PPP1R2P1	3.656192
PPP1R2P3	3.644468
PPP1R2P9	3.295114
PPP1R3A	2.756864

PPP1R3B	4.520548
PPP1R3C	5.711404
PPP1R3D	7.676094
PPP1R3E	5.663942
PPP1R3F	5.434494
PPP1R7	6.878012
PPP1R8	8.398168
PPP1R9A	4.303642
PPP1R9B	6.091246
PPP2CA	9.140058
PPP2CB	8.036864
PPP2R1A	9.44932
PPP2R1B	8.358258
PPP2R2A	7.824426
PPP2R2B	2.892758
PPP2R2C	6.908792
PPP2R2D	7.791702
PPP2R3A	6.078722
PPP2R3B	5.039794
PPP2R3B	5.118636
PPP2R3C	4.755144
PPP2R4	9.291138
PPP2R5A	6.699854
PPP2R5B	6.091368
PPP2R5C	6.151168
PPP2R5D	8.14193
PPP2R5E	8.080846
PPP3CA	8.528472
PPP3CB	6.665524
PPP3CC	5.693868
PPP3R1	8.441698
PPP3R1	3.844276
PPP3R1	2.063222
PPP3R2	3.695656
PPP4C	8.864538
PPP4R1	9.14561
PPP4R1L	4.143714
PPP4R2	7.366218
PPP4R4	2.728464
PPP5C	7.102466
PPP6C	9.461976
PPP6R1	8.126218
PPP6R2	6.451512

PPP6R3	8.46647
PPPDE1	6.364566
PPPDE2	7.408444
PPRC1	5.791706
PPT1	9.859286
PPT2	5.828444
PPT2	5.897
PPTC7	7.194542
PPWD1	5.653054
PPY	3.102476
PPY2	3.884684
PPYR1	4.939808
PQBP1	7.708664
PQLC1	5.925326
PQLC2	6.142632
PQLC3	7.09671
PRAC	3.689416
PRAF2	5.04492
PRAM1	5.259366
PRAME	3.41107
PRAMEF1	2.592256
PRAMEF10	3.01355
PRAMEF10	3.01355
PRAMEF10	2.958552
PRAMEF11	2.584848
PRAMEF12	3.359548
PRAMEF13	2.538742
PRAMEF13	2.538742
PRAMEF15	2.831236
PRAMEF15	2.831236
PRAMEF16	2.812326
PRAMEF17	2.900668
PRAMEF18	3.112266
PRAMEF18	3.112266
PRAMEF2	2.447162
PRAMEF20	4.053296
PRAMEF20	3.95429
PRAMEF22	3.635848
PRAMEF3	3.39729
PRAMEF3	3.677166
PRAMEF4	2.853014
PRAMEF5	2.868472
PRAMEF5	2.839192

PRAMEF5	2.868472
PRAMEF5	2.818552
PRAMEF7	4.449118
PRAMEF7	4.449118
PRAMEF7	4.449118
PRAMEL	3.187528
PRAP1	4.817928
PRB1	4.406286
PRB2	4.395026
PRB3	5.419182
PRB4	4.188102
PRC1	9.628096
PRCC	9.097356
PRCP	6.620766
PRDM1	5.199316
PRDM10	5.719222
PRDM11	4.185344
PRDM12	5.122364
PRDM13	5.20444
PRDM14	3.100212
PRDM15	5.102756
PRDM16	4.1105
PRDM2	5.356704
PRDM4	6.62051
PRDM5	2.891716
PRDM6	4.40525
PRDM7	2.71817
PRDM8	4.400132
PRDM9	2.485766
PRDX1	10.86508
PRDX2	5.921542
PRDX3	9.849656
PRDX4	7.249154
PRDX5	9.936744
PRDX6	10.29194
PREB	7.712184
PRELID1	9.754322
PRELID2	6.058092
PRELP	4.070874
PREP	7.611124
PREPL	7.30663
PREX1	3.795556
PREX2	2.673406

PRF1	4.427308
PRG2	3.454524
PRG3	4.535954
PRG4	2.642832
PRH1	4.464476
PRH2	2.296536
PRHOXNB	4.43663
PRIC285	6.461824
PRICKLE1	6.533662
PRICKLE2	5.361676
PRICKLE3	5.50465
PRICKLE4	5.360926
PRIM1	8.84486
PRIM2	7.134714
PRIMA1	3.817926
PRKAA1	8.543722
PRKAA2	2.904976
PRKAB1	7.337758
PRKAB2	4.769882
PRKACA	8.154164
PRKACB	3.991906
PRKACG	3.602436
PRKAG1	9.46868
PRKAG2	6.524104
PRKAG3	3.373646
PRKAR1A	10.416416
PRKAR1B	7.219008
PRKAR2A	6.6639
PRKAR2B	7.728656
PRKCA	4.56472
PRKCB	3.259326
PRKCD	7.830832
PRKCDBP	7.666024
PRKCE	5.82279
PRKCG	3.574672
PRKCH	5.956156
PRKCI	8.807348
PRKCQ	3.92937
PRKCSH	9.437234
PRKCZ	6.5817
PRKD1	2.39879
PRKD2	6.391744
PRKD3	4.171072

PRKDC	9.738548
PRKG1	2.635946
PRKG2	2.371348
PRKRA	8.058432
PRKRIP1	6.400138
PRKRIR	6.23398
PRKX	6.083602
PRKY	3.973636
PRL	2.519566
PRLH	5.476422
PRLHR	4.100904
PRLR	4.019236
PRM1	3.379926
PRM2	3.835476
PRM3	4.138902
PRMT1	8.474572
PRMT10	6.22419
PRMT2	6.496086
PRMT3	5.74466
PRMT5	9.370836
PRMT6	4.75606
PRMT7	7.119622
PRMT8	3.227222
PRND	3.669816
PRNP	9.955174
PRNT	2.891508
PRO0611	2.92427
PRO1768	2.79163
PRO2012	4.60339
PRO2012	3.027492
PRO2964	2.010674
PROC	3.360046
PROCA1	4.699056
PROCR	4.507542
PRODH	4.360422
PRODH2	3.520604
PROK1	3.300544
PROK2	4.65483
PROKR1	3.522412
PROKR2	3.672578
PROL1	2.694168
PROM1	2.91754
PROM2	7.045982

PROP1	5.440868
PRORSD1P	5.076714
PROS1	2.664864
PROS1	3.033574
ProSAPiP1	5.187544
PROSC	6.955936
PROX1	2.670062
PROX2	2.457888
PROZ	3.757054
PRPF18	6.433996
PRPF19	9.098046
PRPF3	8.25065
PRPF31	7.747802
PRPF38A	8.396982
PRPF38B	6.552832
PRPF39	5.887322
PRPF4	9.980136
PRPF40A	7.839132
PRPF40B	5.462224
PRPF4B	6.583004
PRPF6	8.861704
PRPF8	9.49888
PRPH	4.373944
PRPH2	3.205656
PRPS1	7.872014
PRPS1L1	3.41978
PRPS2	7.407488
PRPSAP1	7.6898
PRPSAP2	7.599568
PRR11	8.183346
PRR12	6.07371
PRR13	9.283466
PRR13	9.042312
PRR14	6.540626
PRR15	8.359658
PRR15L	6.211624
PRR16	2.730196
PRR18	5.72591
PRR19	3.512596
PRR20A	3.884324
PRR20A	3.884324
PRR20A	3.884324
PRR20A	3.884324

PRR20A	3.884324
PRR21	3.451462
PRR22	4.798888
PRR23A	5.39558
PRR23B	4.548324
PRR23C	2.775498
PRR25	4.1522
PRR3	6.114308
PRR4	4.757058
PRR5	6.553772
PRR5L	4.061834
PRR7	5.514018
PRRC1	8.13826
PRRG1	5.939
PRRG2	4.38739
PRRG3	3.383352
PRRG4	6.599248
PRRT1	4.799284
PRRT1	4.73708
PRRT1	4.73708
PRRT2	4.642938
PRRT3	4.264964
PRRX1	3.126186
PRRX2	5.57325
PRSS1	2.535558
PRSS12	7.76683
PRSS16	5.116242
PRSS2	3.667492
PRSS21	4.704538
PRSS22	6.104546
PRSS23	5.917876
PRSS27	5.636952
PRSS29P	5.78283
PRSS3	4.778468
PRSS30P	4.98717
PRSS33	4.738126
PRSS35	3.47626
PRSS36	5.089108
PRSS37	2.565148
PRSS38	2.709352
PRSS41	4.656084
PRSS42	6.123128
PRSS45	4.261056

PRSS48	2.331288
PRSS50	3.787126
PRSS53	4.286402
PRSS54	3.925904
PRSS55	2.717458
PRSS8	7.273228
PRSSL1	5.86492
PRTFDC1	4.105714
PRTG	3.708038
PRTN3	5.809706
PRUNE	5.561574
PRUNE2	3.15449
PRUNE2	2.729496
PRX	4.432504
PRY	2.962288
PRY	2.9177
PRY	2.948566
PRY	2.9177
PS1TP4	5.310298
PSAP	10.56158
PSAPL1	3.934568
PSAT1	9.666342
PSCA	7.180098
PSCA	5.307744
PSD	4.356222
PSD2	4.344194
PSD3	4.461936
PSD3	5.196768
PSD4	5.888972
PSEN1	9.016648
PSEN2	5.689572
PSENEN	9.134782
PSG1	3.285138
PSG11	2.799842
PSG2	2.434648
PSG3	2.436354
PSG4	5.31518
PSG5	4.28493
PSG6	3.758592
PSG7	4.877118
PSG8	2.805084
PSG9	4.772104
PSIP1	5.678392

psiTPTE22	3.200328
psiTPTE22	3.314184
PSKH1	6.545162
PSKH2	3.821534
PSMA1	9.597402
PSMA2	7.601984
PSMA3	8.364824
PSMA4	10.5621
PSMA5	8.700962
PSMA6	10.30876
PSMA7	9.355712
PSMA8	2.41079
PSMB1	8.867464
PSMB10	6.361166
PSMB11	2.68935
PSMB2	10.459
PSMB3	10.77426
PSMB4	10.385826
PSMB5	7.495376
PSMB6	9.848178
PSMB7	10.84354
PSMB8	8.61452
PSMB8	8.61452
PSMB8	8.61452
PSMB9	9.015418
PSMB9	9.015418
PSMB9	9.015418
PSMC1	9.44192
PSMC1	9.644348
PSMC2	10.40498
PSMC3	9.399062
PSMC3IP	7.8045
PSMC4	8.471424
PSMC5	9.532822
PSMC6	7.021794
PSMC6	7.257886
PSMD1	9.587452
PSMD10	6.771734
PSMD11	10.3088
PSMD12	8.338296
PSMD13	7.207798
PSMD14	9.228474
PSMD2	9.353398

PSMD3	9.52879
PSMD4	7.170546
PSMD5	9.388516
PSMD6	8.741116
PSMD7	9.952424
PSMD8	8.815372
PSMD9	6.928326
PSME1	9.163872
PSME2	8.61923
PSME3	9.517716
PSME4	7.832748
PSMF1	9.28478
PSMG1	7.28626
PSMG2	8.915172
PSMG3	7.956856
PSMG4	4.723456
PSORS1C1	3.89003
PSORS1C1	4.347982
PSORS1C2	4.855936
PSORS1C2	4.847462
PSORS1C3	2.79746
PSORS1C3	2.79746
PSPC1	8.544906
PSPH	8.77085
PSPH	8.989976
PSPN	4.931766
PSRC1	6.329788
PSTK	4.111566
PSTPIP1	4.182984
PSTPIP2	2.942268
PTAFR	6.011184
PTAR1	6.301728
PTBP1	10.13929
PTBP2	5.389488
PTCD1	5.99276
PTCD2	6.429266
PTCD3	8.784214
PTCH1	5.493816
PTCH2	4.06973
PTCH2	4.295538
PTCHD1	3.73627
PTCHD2	4.011642
PTCHD3	2.377866

PTCRA	4.727068
PTDSS1	9.924278
PTDSS2	5.747166
PTEN	3.736714
PTENP1	2.481564
PTER	7.430596
PTF1A	5.42437
PTGDR	3.366886
PTGDS	4.337772
PTGER1	5.182866
PTGER2	5.437078
PTGER3	2.772646
PTGER4	4.578792
PTGES	9.027792
PTGES2	6.535356
PTGES3	9.89861
PTGFR	2.603422
PTGFRN	7.341592
PTGIR	3.905676
PTGIS	6.387854
PTGR1	8.151666
PTGR2	7.28402
PTGS1	8.210666
PTGS2	7.005688
PTH	2.107522
PTH1R	4.336012
PTH2	6.029104
PTH2R	3.099324
PTHLH	3.273724
PTK2	9.380416
PTK2B	6.268206
PTK6	5.79606
PTK7	7.37308
PTMA	10.40536
PTMA	10.4491
PTMA	5.232506
PTMA	12.22696
PTMA	12.0553
PTMAP5	9.430916
PTMS	8.803992
PTN	2.97456
PTOV1	6.392828
PTP4A1	9.169078

PTP4A2	10.4711
PTP4A3	6.852868
PTPDC1	5.403152
PTPLA	6.305616
PTPLAD1	7.898308
PTPLAD2	2.311868
PTPLB	9.452244
PTPMT1	7.604148
PTPN1	7.641744
PTPN11	8.22544
PTPN12	7.56678
PTPN13	7.121362
PTPN14	6.414744
PTPN18	5.59837
PTPN2	7.165458
PTPN20A	4.149418
PTPN20A	4.45339
PTPN20A	3.93675
PTPN20B	4.164106
PTPN21	5.24824
PTPN22	2.478548
PTPN23	5.76804
PTPN3	6.598712
PTPN4	5.644288
PTPN5	3.852826
PTPN6	7.378922
PTPN7	3.730118
PTPN9	7.276748
PTPRA	6.356906
PTPRB	2.791228
PTPRC	2.407248
PTPRCAP	5.452014
PTPRD	2.849438
PTPRE	5.080118
PTPRF	7.667866
PTPRG	5.06644
PTPRH	5.850936
PTPRJ	7.748866
PTPRK	9.15442
PTPRM	6.616704
PTPRN	3.799258
PTPRN2	3.882242
PTPRO	2.692036

PTPRR	2.928362
PTPRS	7.514284
PTPRT	3.526214
PTPRU	6.556564
PTPRZ1	2.703478
PTRF	7.882082
PTRH1	3.515038
PTRH1	6.209392
PTRH2	6.647676
PTS	7.744342
PTTG1	9.707054
PTTG1IP	9.920522
PTTG2	2.99156
PTTG3P	3.759156
PTX3	2.851588
PTX4	4.79648
PUF60	9.374522
PUM1	9.17307
PUM2	8.206028
PURA	7.305458
PURB	8.205866
PURG	2.623686
PUS1	5.53696
PUS10	4.822102
PUS3	5.477852
PUS7	6.353716
PUS7L	5.36313
PUSL1	5.797804
PVALB	3.264814
PVR	7.500626
PVRIG	4.370472
PVRL1	5.366236
PVRL2	6.772114
PVRL3	2.99691
PVRL4	6.08877
PWP1	7.966352
PWP2	7.057318
PWWP2A	6.437606
PWWP2B	6.12933
PXDN	6.96247
PXDNL	3.022374
PXK	4.340294
PXMP2	8.557828

PXMP2	5.567102
PXMP4	5.674094
PXN	6.767528
PXN	4.782482
PXT1	2.261168
PYCARD	7.12481
PYCR1	6.647628
PYCR2	7.758202
PYCRL	6.576484
PYDC1	3.587488
PYDC2	3.397608
PYGB	9.038412
PYGL	6.073368
PYGM	4.182326
PYGO1	3.064626
PYGO2	6.0956
PYHIN1	2.482706
PYROXD1	5.299454
PYROXD2	5.040622
PYY	4.195668
PYY2	5.766926
PZP	2.63345
QARS	8.341232
QDPR	6.079702
QKI	5.672632
QPCT	2.511422
QPCTL	6.166992
QPRT	4.255702
QRFP	4.924844
QRFPR	1.905472
QRFPR	2.61563
QRICH1	8.059386
QRICH2	4.018862
QRSL1	7.734454
QSER1	6.547382
QSOX1	8.501976
QSOX2	6.187558
QTRT1	7.006002
QTRTD1	7.588488
R3HCC1	5.87354
R3HDM1	6.2737
R3HDM2	6.41665
R3HDML	3.230394

RAB10	9.634518
RAB11A	9.37332
RAB11B	8.12512
RAB11FIP1	5.974736
RAB11FIP2	4.723032
RAB11FIP3	5.474368
RAB11FIP4	6.974502
RAB11FIP5	5.720224
RAB12	6.397008
RAB13	7.44296
RAB13	6.851008
RAB14	9.858262
RAB15	6.414938
RAB17	4.825008
RAB18	8.57092
RAB19	6.089394
RAB1A	9.584056
RAB1B	10.39414
RAB1C	7.860036
RAB20	5.494602
RAB21	8.20358
RAB22A	7.563278
RAB23	5.569952
RAB24	6.643706
RAB25	9.595146
RAB26	6.0014
RAB27A	7.360354
RAB27B	5.496522
RAB28	5.580384
RAB2A	9.214156
RAB2B	6.45554
RAB30	4.33428
RAB31	8.1525
RAB32	6.380192
RAB33A	4.115088
RAB33B	5.812528
RAB34	5.623234
RAB35	7.632306
RAB36	4.184828
RAB36	3.677878
RAB37	3.726298
RAB38	3.634376
RAB39	2.231718

RAB39B	2.629824
RAB3A	4.646376
RAB3B	3.136102
RAB3C	2.974926
RAB3D	6.675032
RAB3GAP1	8.420178
RAB3GAP2	6.528184
RAB3IL1	4.348562
RAB3IP	5.67773
RAB40A	4.70339
RAB40AL	4.267974
RAB40B	5.625952
RAB40C	6.007482
RAB41	2.861806
RAB42	3.403234
RAB43	7.686498
RAB43	5.81438
RAB44	3.107022
RAB44	3.666268
RAB4A	5.835148
RAB4B	5.944234
RAB5A	9.405616
RAB5B	7.53947
RAB5C	9.66607
RAB6A	7.839132
RAB6B	5.151004
RAB6C	7.151592
RAB6C	7.50408
RAB7A	10.56172
RAB7L1	6.833978
RAB8A	8.73879
RAB8B	5.85123
RAB9A	7.21136
RAB9B	3.117478
RAB9BP1	2.418468
RABAC1	8.062222
RABEP1	6.64357
RABEP2	5.69749
RABEPK	6.4702
RABGAP1	9.44011
RABGAP1L	5.50094
RABGEF1	9.466864
RABGGTA	6.07514

RABGGTB	7.651294
RABIF	6.740002
RABL2A	6.50051
RABL2B	6.362838
RABL3	7.533822
RABL5	7.202116
RAC1	7.67089
RAC2	5.81379
RAC3	5.139152
RACGAP1	9.388548
RACGAP1P	5.20608
RAD1	7.73064
RAD17	4.920894
RAD17	4.7579
RAD18	7.641278
RAD21	9.931568
RAD21L1	2.272614
RAD21L1	3.98336
RAD23A	9.169094
RAD23B	8.923036
RAD50	6.626844
RAD51	7.799192
RAD51AP1	7.519984
RAD51C	6.528616
RAD51L1	6.393994
RAD51L3	6.663994
RAD52	6.932722
RAD54B	5.459396
RAD54L	6.20733
RAD54L2	6.101826
RAD9A	7.311778
RAD9B	3.557316
RADIL	4.201322
RAE1	8.629634
RAET1E	4.94558
RAET1G	5.538838
RAET1L	7.985134
RAF1	9.131934
RAG1	2.426602
RAG1AP1	8.68851
RAG2	2.297868
RAGE	5.459268
RAI1	6.281988

RAI14	3.951358
RAI2	5.217926
RALA	6.95263
RALB	8.340916
RALBP1	8.659076
RALGAPA1	4.884656
RALGAPA2	5.780776
RALGAPB	7.98459
RALGDS	5.17313
RALGPS1	6.226162
RALGPS2	7.000198
RALY	9.06579
RALYL	2.642588
RAMP1	5.908876
RAMP2	3.841728
RAMP3	5.38475
RAN	8.373946
RAN	10.160368
RANBP1	9.73305
RANBP10	6.535958
RANBP17	6.381458
RANBP2	6.8066
RANBP3	8.309938
RANBP3L	2.310158
RANBP6	6.44127
RANBP9	8.324304
RANGAP1	8.04666
RANGRF	4.099562
RAP1A	6.51722
RAP1B	8.37636
RAP1B	8.528548
RAP1GAP	4.885764
RAP1GAP2	6.386732
RAP1GDS1	7.185714
RAP2A	6.834344
RAP2B	7.498774
RAP2C	6.545828
RAPGEF1	6.580114
RAPGEF1	3.460252
RAPGEF2	5.545266
RAPGEF3	4.021478
RAPGEF3	4.322982
RAPGEF4	3.455652

RAPGEF5	4.407964
RAPGEF6	5.125782
RAPGEFL1	6.69372
RAPH1	5.813726
RAPSN	4.827554
RARA	6.108368
RARB	3.320528
RARG	7.50327
RARRES1	10.65018
RARRES2	7.005998
RARRES3	8.356024
RARS	9.39329
RARS2	7.598596
RASA1	7.646684
RASA2	5.580766
RASA3	6.025732
RASA4	4.985518
RASA4	5.474106
RASA4	5.12514
RASA4	3.528732
RASAL1	5.829922
RASAL2	6.60194
RASAL3	3.840986
RASD1	5.2076
RASD2	5.797286
RASEF	5.988064
RASGEF1A	4.459336
RASGEF1B	2.486584
RASGEF1C	4.066146
RASGRF1	4.04698
RASGRF1	4.178754
RASGRF2	3.132628
RASGRP1	3.057958
RASGRP2	4.131544
RASGRP3	2.403654
RASGRP4	3.657838
RASIP1	4.422124
RASL10A	4.616194
RASL10B	5.208986
RASL11A	4.68963
RASL11B	3.028556
RASL12	4.02028
RASSF1	6.02463

RASSF10	4.711176
RASSF2	5.745492
RASSF3	5.223318
RASSF4	3.695138
RASSF5	5.3581
RASSF6	2.49183
RASSF7	6.322094
RASSF8	5.695522
RASSF9	2.54837
RAVER1	6.499934
RAVER2	5.948132
RAX	4.455262
RAX2	4.527404
RB1	6.735382
RB1CC1	5.196196
RBAK	6.161256
RBBP4	10.157368
RBBP5	7.19165
RBBP6	6.813918
RBBP7	9.22953
RBBP8	7.20457
RBBP9	4.058884
RBCK1	7.2608
RBFA	5.615144
RBFOX1	2.55448
RBFOX2	8.61607
RBFOX3	4.61894
RBFOX3	4.38091
RBKS	5.006722
RBL1	3.835356
RBL1	6.802398
RBL2	7.117586
RBM10	6.863704
RBM11	2.141288
RBM12B	6.749092
RBM14	8.307872
RBM14	6.903916
RBM14	6.870108
RBM15	7.161908
RBM15B	5.950054
RBM16	6.966882
RBM17	6.270046
RBM18	7.423716

RBM19	6.460206
RBM22	8.807266
RBM23	8.201948
RBM24	3.42983
RBM25	7.440998
RBM26	7.268744
RBM27	7.44604
RBM28	6.414192
RBM3	7.243978
RBM33	6.782922
RBM33	6.514888
RBM34	7.559844
RBM38	6.538622
RBM38	3.277142
RBM39	9.112958
RBM4	8.535444
RBM41	4.120064
RBM42	7.58971
RBM43	3.922476
RBM44	2.08786
RBM45	4.199362
RBM46	2.557238
RBM47	8.659948
RBM4B	7.692084
RBM5	7.218814
RBM6	6.058798
RBM7	6.031484
RBM8A	4.944396
RBMS1	5.231928
RBMS1	7.233128
RBMS1	6.119684
RBMS2	7.800042
RBMS3	3.156396
RBMX	9.092988
RBMX2	8.63259
RBMXL2	5.281868
RBMXL3	4.317424
RBMY1A1	2.193874
RBMY1A1	2.219874
RBMY1A1	2.204136
RBMY1A1	2.180024
RBMY1A3P	2.611112
RBMY1B	2.220866

RBMY1B	2.220866
RBMY1B	2.301652
RBMY2EP	2.402376
RBP1	5.89455
RBP2	3.96221
RBP3	3.8732
RBP4	3.21302
RBP5	4.581898
RBP7	2.798786
RBPJ	7.456684
RBPJ	4.38509
RBPJL	4.390132
RBPMS	6.467498
RBPMS2	5.470624
RBX1	9.64758
RC3H1	7.382468
RC3H2	7.844748
RCAN1	7.817552
RCAN2	3.93779
RCAN3	6.425384
RCBTB1	5.528676
RCBTB2	4.375648
RCC1	8.225968
RCC2	9.660466
RCCD1	7.304778
RCE1	7.686184
RCHY1	5.198134
RCL1	7.062212
RCN1	8.851308
RCN1	5.865584
RCN2	7.859872
RCN3	3.77223
RCOR1	7.345462
RCOR2	3.383682
RCOR3	5.675226
RCSD1	4.371786
RCVRN	3.560994
RD3	3.064148
RDBP	7.65223
RDBP	7.65223
RDBP	7.65223
RDH10	8.173822
RDH11	7.943192

RDH12	3.618454
RDH13	7.47128
RDH14	6.189078
RDH16	3.922832
RDH5	4.674144
RDH8	3.552558
RDM1	4.920432
RDX	7.735588
REC8	5.408718
RECK	3.247186
RECQL	7.117692
RECQL4	6.27188
RECQL5	5.766606
REEP1	3.626466
REEP2	4.796448
REEP3	7.076432
REEP3	5.913502
REEP4	7.311276
REEP5	7.972522
REEP6	6.212262
REG1A	3.579518
REG1B	2.160864
REG1P	2.63135
REG3A	2.377356
REG3G	2.673874
REG4	2.65454
REL	6.515594
RELA	8.44689
RELB	5.784012
RELL1	6.795198
RELL2	5.405248
RELN	3.139288
RELT	5.629158
REM1	4.219582
REM2	3.725352
REN	3.808856
RENBP	4.064748
REP15	2.555394
REPIN1	7.682664
REPS1	7.724466
REPS2	4.087018
RER1	9.169054
RERE	6.206202

RERG	5.881324
RERGL	2.775626
RESP18	3.46049
REST	6.804544
RET	3.88102
RETN	5.277844
RETNLB	3.094018
RETSAT	7.248938
REV1	6.370846
REV3L	5.514738
REXO1	5.590232
REXO1L1	5.061694
REXO1L1	4.624204
REXO1L1	5.061694
REXO1L1	5.061694
REXO1L1	5.061694
REXO1L1	5.061694
REXO1L2P	5.22986
REXO1L2P	5.367684
REXO1L2P	5.22986
REXO1L2P	4.109272
REXO2	8.165354
REXO4	6.456594
RFC1	7.798922
RFC1	1.925796
RFC2	8.97296
RFC3	9.80639
RFC4	8.817254
RFC5	7.499808
RFESD	3.008614
RFFL	7.726044
RFK	6.50246
RFNG	5.10968
RFPL1	2.757172
RFPL2	3.186126
RFPL3	1.827932
RFPL4A	2.618924
RFPL4A	2.53628
RFPL4B	4.800228
RFT1	4.351012
RFT1	7.962296
RFTN1	3.79273
RFTN1	2.676216

RFTN2	2.630574
RFWD2	7.42982
RFWD3	8.761226
RFX1	4.980464
RFX2	4.708766
RFX3	3.61718
RFX4	2.541156
RFX5	7.508826
RFX6	2.56417
RFX7	5.057164
RFX8	2.400436
RFXANK	8.531356
RFXAP	4.764422
RG9MTD1	5.5516
RG9MTD2	4.437038
RG9MTD3	5.361992
RGAG1	3.319822
RGAG4	3.138508
RGL1	5.514566
RGL2	6.394726
RGL2	6.493052
RGL2	6.493052
RGL3	3.750598
RGL4	3.416888
RGMA	5.467042
RGMB	4.41695
RGN	2.540224
RGNEF	6.054974
RGP1	6.537116
RGPD1	6.200208
RGPD1	6.200208
RGPD3	7.555924
RGPD4	7.35699
RGPD5	7.518488
RGPD5	7.692242
RGPD5	7.647702
RGPD6	7.455334
RGR	3.62177
RGS1	2.207564
RGS10	6.688026
RGS11	3.784874
RGS12	5.202922
RGS13	2.704908

RGS14	5.437426
RGS16	4.4789
RGS17	4.487108
RGS18	2.09687
RGS19	6.42471
RGS2	5.387768
RGS20	4.27159
RGS21	2.683124
RGS22	2.422406
RGS3	6.438546
RGS4	3.267048
RGS5	3.490682
RGS6	3.5725
RGS7	2.863646
RGS7BP	2.801082
RGS8	3.61302
RGS9	3.33825
RGS9BP	3.664126
RGSL1	2.545922
RGSL1	2.493458
RHAG	2.929838
RHBDD1	7.234212
RHBDD2	8.79515
RHBDD3	6.610476
RHBDF1	5.697182
RHBDF2	5.231722
RHBDL1	5.206024
RHBDL2	6.580262
RHBDL3	3.898954
RHBG	4.310826
RHCE	3.019018
RHCG	3.440726
RHD	3.35927
RHEB	9.320066
RHEB	8.717488
RHEBL1	4.548878
RHO	3.423134
RHOA	10.80494
RHOB	7.69866
RHOBTB1	2.723568
RHOBTB2	5.169004
RHOBTB3	7.731726
RHOC	8.287536

RHOD	7.721192
RHOF	7.285928
RHOG	7.021984
RHOH	2.796122
RHOJ	2.995428
RHOQ	7.849258
RHOQ	6.09781
RHOT1	7.459656
RHOT2	6.547974
RHOU	5.436092
RHOV	6.505828
RHOXF1	3.91218
RHOXF2	3.709214
RHOXF2	3.709214
RHPN1	5.167058
RHPN2	8.797554
RIBC1	4.378338
RIBC2	5.103758
RIC3	2.579332
RIC8A	7.746694
RIC8B	5.800918
RICTOR	6.654226
RIF1	7.5335
RILP	6.224134
RILPL1	5.19342
RILPL2	5.194506
RIMBP2	3.76174
RIMBP3	4.094412
RIMBP3	3.407282
RIMBP3	4.538028
RIMBP3	4.655188
RIMBP3C	3.030844
RIMKLA	4.936734
RIMKLB	4.72222
RIMS1	2.729518
RIMS2	3.323852
RIMS3	4.52884
RIMS4	4.397948
RIN1	5.371478
RIN2	6.537972
RIN3	5.605516
RING1	7.165346
RING1	7.211082

RINL	3.863904
RINT1	5.86589
RINT1	2.190382
RIOK1	7.115602
RIOK2	5.269502
RIOK3	8.408244
RIPK1	6.723496
RIPK2	8.300744
RIPK3	3.123464
RIPK4	7.724586
RIPPLY1	3.407448
RIPPLY2	3.475438
RIT1	7.543936
RIT2	2.999422
RLBP1	4.548582
RLF	6.378498
RLIM	3.092998
RLIM	9.376778
RLIM	6.526544
RLN1	3.02365
RLN2	3.127484
RLN3	3.899628
RLTPR	4.537198
RMI1	6.36882
RMND1	4.309056
RMND5A	8.566304
RMND5B	7.405254
RMRP	10.243166
RN18S1	11.53428
RN28S1	12.9798
RN28S1	12.7779
RN7SK	12.56774
RN7SL1	11.83462
RNASE1	4.77163
RNASE10	2.667438
RNASE11	2.217664
RNASE12	2.590182
RNASE13	3.06699
RNASE2	2.4626
RNASE3	3.924742
RNASE6	2.732312
RNASE7	3.434686
RNASE8	2.602722

RNASE9	2.999584
RNASEH1	7.22565
RNASEH2A	9.538378
RNASEH2B	5.671986
RNASEH2C	7.653032
RNASEK	10.46444
RNASEK	6.793586
RNASEK	5.909444
RNASEL	6.062828
RNASET2	4.966576
RND1	4.277494
RND2	4.056844
RND3	7.754826
RNF10	8.43876
RNF103	6.89454
RNF11	6.602546
RNF111	6.806968
RNF112	3.271026
RNF113A	3.889346
RNF113B	4.771536
RNF114	9.492302
RNF115	6.955212
RNF121	8.275728
RNF122	4.281332
RNF123	5.291858
RNF125	3.000978
RNF126	6.613364
RNF126P1	5.082092
RNF128	2.65673
RNF13	7.188832
RNF130	7.32559
RNF133	2.101792
RNF135	6.24318
RNF138	5.697774
RNF139	7.196386
RNF14	7.88108
RNF141	5.562002
RNF144A	4.79965
RNF144B	6.961634
RNF145	7.805722
RNF146	7.029134
RNF148	2.93166
RNF149	8.16414

RNF150	3.226378
RNF151	4.467524
RNF152	3.686482
RNF157	4.319456
RNF165	4.075238
RNF166	5.49436
RNF167	7.976464
RNF168	8.679134
RNF169	6.672238
RNF17	2.321914
RNF170	7.093406
RNF175	2.514074
RNF180	2.266424
RNF181	7.712682
RNF182	4.569476
RNF183	4.718378
RNF185	6.64314
RNF185	2.865612
RNF186	4.112542
RNF187	7.507114
RNF19A	7.305612
RNF19B	7.870982
RNF2	5.67383
RNF20	7.48769
RNF207	5.104862
RNF207	5.558666
RNF208	4.997974
RNF208	4.997974
RNF212	3.393844
RNF213	7.944762
RNF213	8.229516
RNF213	3.82079
RNF214	5.71701
RNF215	5.48769
RNF216	7.03492
RNF216L	5.560924
RNF217	6.592002
RNF219	6.68611
RNF220	6.353188
RNF222	5.304266
RNF24	7.434984
RNF25	6.056138
RNF26	7.576216

RNF31	6.866684
RNF31	5.32385
RNF32	5.249814
RNF34	8.101486
RNF38	6.623032
RNF39	5.834884
RNF39	5.834884
RNF4	7.224286
RNF40	6.932856
RNF41	6.423488
RNF43	4.444236
RNF44	5.744884
RNF5	7.617804
RNF5	7.617804
RNF5P1	7.244942
RNF5P1	8.411366
RNF6	6.34715
RNF7	6.713672
RNF8	6.125132
RNF8	3.892996
RNFT1	7.337528
RNFT2	6.325984
RNGTT	6.99367
RNH1	7.068078
RNLS	6.025202
RNMT	6.682708
RNMTL1	6.956854
RNPC3	4.61875
RNPEP	9.542912
RNPEPL1	5.248278
RNPS1	9.291344
RNU105B	3.843324
RNU105C	3.396098
RNU11	6.627606
RNU1-1	9.558646
RNU1-1	9.558646
RNU1-1	9.558646
RNU1-1	9.558646
RNU1-1	9.703378
RNU1-1	9.703378
RNU1-1	9.558646
RNU1-1	9.542456
RNU1-1	9.542456

RNU12	5.23019
RNU2-1	5.961792
RNU2-1	10.24718
RNU2-1	10.24718
RNU2-1	10.24718
RNU2-1	10.24718
RNU2-1	10.24718
RNU2-1	10.24718
RNU2-1	10.24718
RNU2-1	10.24718
RNU2-1	10.24718
RNU2-1	10.18752
RNU2-1	10.207218
RNU4-1	5.892316
RNU4-2	4.317262
RNU4ATAC	6.215524
RNU5A	6.165846
RNU5B-1	5.644954
RNU5D	10.97048
RNU5E	10.012292
RNU5F	2.295826
RNU6ATAC	4.787556
RNU6ATAC	5.512704
RNY4P8	6.706626
ROBLD3	6.216318
ROBO1	3.486372
ROBO2	3.049898
ROBO3	4.143388
ROBO4	4.543566
ROCK1	5.92639
ROCK1P1	2.641996
ROCK2	6.79362
ROD1	9.202832
ROGDI	6.692132
ROM1	4.43438
ROMO1	6.85203
ROPN1	2.796176
ROPN1B	2.697582
ROPN1L	3.658458
ROR1	6.035914
ROR2	4.052708
RORA	3.135232
RORB	3.293888

RORC	4.599854
ROS1	2.62064
RP1	2.44206
RP1L1	3.490916
RP2	5.84073
RP9	6.407476
RP9P	6.26322
RPA1	8.927534
RPA2	6.855544
RPA3	6.502152
RPA4	3.12923
RPAIN	6.74036
RPAP1	5.563024
RPAP2	4.530342
RPAP3	5.041482
RPE	7.48682
RPE65	2.54526
RPF1	6.393682
RPF2	8.484978
RPF2	7.481164
RPGR	3.029146
RPGRIP1	3.060928
RPGRIP1L	4.005056
RPH3A	2.897928
RPH3AL	5.570266
RPIA	6.702578
RPL10	9.211908
RPL10	11.95786
RPL10	9.161738
RPL10A	8.94027
RPL10L	3.528748
RPL11	10.267616
RPL12	11.72394
RPL12	11.74112
RPL12	11.50888
RPL12	5.816788
RPL13	7.09446
RPL13A	11.02008
RPL13AP17	2.659388
RPL13AP20	8.77235
RPL13AP3	4.942194
RPL13AP5	10.87354
RPL13AP6	4.870748

RPL13P5	5.08242
RPL14	10.81146
RPL14	6.513606
RPL15	9.551948
RPL15	10.37042
RPL17	9.88835
RPL17	10.30862
RPL17	5.06804
RPL18	11.74392
RPL18A	10.65752
RPL18A	8.141068
RPL18AP3	10.71356
RPL19	8.961316
RPL21	10.46634
RPL21	10.6271
RPL21	10.52786
RPL21	10.3378
RPL21P28	10.15322
RPL21P44	3.061538
RPL22	6.26744
RPL22L1	5.279982
RPL22P15	5.99705
RPL23	7.527486
RPL23A	10.042214
RPL23A	10.6114
RPL23A	10.6588
RPL23AP32	4.228406
RPL23AP53	2.930482
RPL23AP64	2.79411
RPL23AP7	7.62163
RPL23AP71	3.266376
RPL23AP82	7.086424
RPL23AP82	6.969102
RPL23AP82	7.21958
RPL23P8	5.60355
RPL24	10.186362
RPL24	9.946462
RPL26	7.470542
RPL26L1	7.754068
RPL26L1	3.728708
RPL27	8.65658
RPL27A	6.538712
RPL27A	5.6793

RPL28	8.908678
RPL29	7.801322
RPL3	10.97224
RPL30	11.60436
RPL31	6.6551
RPL32	6.231066
RPL32P3	5.869898
RPL34	10.88312
RPL34	5.256306
RPL35	10.69158
RPL35A	11.6064
RPL36	6.88112
RPL36A	8.676366
RPL36A	8.346736
RPL36A	8.04196
RPL36AL	8.970026
RPL36AP40	2.39859
RPL37	8.322052
RPL37A	6.80232
RPL38	9.831866
RPL39	8.801376
RPL39	8.721456
RPL39L	5.02965
RPL3L	4.726864
RPL4	3.407028
RPL41	6.151158
RPL41	7.023894
RPL41	11.1226
RPL41	11.1226
RPL41	11.12182
RPL41	11.18622
RPL41	11.16772
RPL5	5.320386
RPL5	11.70438
RPL5	10.35808
RPL6	11.06722
RPL6	11.1434
RPL7	9.936228
RPL7	10.136892
RPL7	9.708078
RPL7A	10.29728
RPL7L1	9.550672
RPL8	12.0228

RPL9	10.90954
RPL9	10.9245
RPL9	10.82606
RPL9	10.81098
RPLP0	9.556936
RPLP0	10.026574
RPLP1	10.62352
RPLP2	9.454742
RPN1	10.37072
RPN2	9.74678
RPP14	7.366712
RPP21	7.152258
RPP21	7.375722
RPP21	7.375722
RPP25	5.947236
RPP30	7.51735
RPP38	5.4673
RPP40	5.082108
RPPH1	8.755264
RPRD1A	6.792164
RPRD1B	8.3798
RPRD2	6.839356
RPRM	3.839402
RPRML	6.502636
RPS10	9.831478
RPS10P7	4.610654
RPS11	11.3081
RPS12	7.6836
RPS13	10.054788
RPS14	10.061508
RPS14P3	4.378708
RPS15	10.134442
RPS15A	11.0854
RPS16	10.39722
RPS17	11.07226
RPS17	11.07226
RPS18	8.850444
RPS18	8.850444
RPS18	8.850444
RPS18P9	6.880942
RPS19	8.656036
RPS19BP1	7.971494
RPS2	10.89074

RPS2	10.75544
RPS2	11.08922
RPS20	11.411
RPS20P27	6.459638
RPS21	6.946644
RPS23	7.855916
RPS24	7.512822
RPS25	8.350404
RPS25	10.89054
RPS25	7.715352
RPS26	6.505868
RPS26	7.721832
RPS26	9.769054
RPS26	6.881354
RPS26	7.73953
RPS26P11	6.311666
RPS27	10.91902
RPS27	10.307
RPS27	10.26844
RPS27	11.35646
RPS27A	9.119084
RPS27A	7.915818
RPS27A	7.633338
RPS27L	5.039674
RPS28	11.19492
RPS28	11.16588
RPS28	11.1416
RPS29	5.479032
RPS2P32	6.60265
RPS3	11.67522
RPS3A	10.176212
RPS3A	10.63494
RPS3A	9.629702
RPS3A	10.4136
RPS4X	11.12078
RPS4XP21	2.68304
RPS4Y1	3.038686
RPS4Y2	2.781078
RPS5	9.604326
RPS6	10.42618
RPS6KA1	7.13286
RPS6KA2	5.4015
RPS6KA3	6.653496

RPS6KA4	6.35882
RPS6KA5	4.791234
RPS6KA6	2.354118
RPS6KB1	6.984454
RPS6KB2	7.332162
RPS6KC1	5.849904
RPS6KL1	4.636672
RPS6P6	3.046188
RPS7	6.737176
RPS7	8.47968
RPS8	9.8073
RPS9	10.151812
RPSA	9.911992
RPSA	10.092286
RPSAP15	6.421548
RPSAP52	2.367616
RPSAP58	2.985832
RPTN	2.527362
RPTOR	6.00301
RPUSD1	6.020962
RPUSD2	5.806918
RPUSD3	8.234552
RPUSD4	5.875992
RQCD1	8.565208
RRAD	3.416028
RRAGA	6.443348
RRAGB	4.99593
RRAGC	8.139792
RRAGD	4.885832
RRAS	7.59869
RRAS2	7.253874
RRBP1	7.19233
RREB1	6.54501
RRH	2.727264
RRM1	9.045098
RRM2	8.029674
RRM2B	6.510616
RRN3	8.69103
RRN3	7.989682
RRN3P1	8.170926
RRN3P1	8.650792
RRP1	7.91584
RRP12	5.940432

RRP15	5.979628
RRP1B	7.014672
RRP36	6.863004
RRP7A	8.630424
RRP7B	8.748018
RRP7B	4.385342
RRP8	4.878804
RRP9	6.549164
RRS1	7.436682
RS1	2.83799
RSAD1	5.960204
RSAD2	8.749458
RSBN1	4.528444
RSBN1L	6.829596
RSF1	6.573528
RSL1D1	9.396046
RSL24D1	7.309676
RSPH1	3.379588
RSPH10B	3.276662
RSPH10B	3.28856
RSPH3	5.253908
RSPH4A	2.670858
RSPH6A	4.681318
RSPH9	3.351544
RSPO1	4.650046
RSPO2	3.057158
RSPO3	2.899616
RSPO4	4.866428
RSPRY1	7.352916
RSRC1	7.772452
RSRC2	6.69416
RSU1	7.567516
RTBDN	3.895814
RTCD1	7.249888
RTDR1	3.758014
RTEL1	4.971138
RTF1	7.364586
RTKN	6.7672
RTKN2	4.305324
RTL1	3.269852
RTN1	3.651354
RTN2	6.058796
RTN3	6.355194

RTN4	7.612788
RTN4IP1	6.913072
RTN4R	5.603212
RTN4RL1	4.983164
RTN4RL2	5.763952
RTP1	4.464734
RTP2	3.610034
RTP3	2.558184
RTP4	8.424488
RTTN	4.806776
RUFY1	8.524172
RUFY2	5.605058
RUFY3	5.241352
RUFY4	3.834134
RUNDC1	7.327768
RUNDC2A	5.122262
RUNDC2C	4.87586
RUNDC2C	3.98345
RUNDC2C	5.563772
RUNDC3A	3.867698
RUNDC3B	2.788594
RUNX1	7.799046
RUNX1T1	3.033814
RUNX2	5.556674
RUNX3	5.654152
RUSC1	5.98977
RUSC2	5.73184
RUVBL1	8.780142
RUVBL2	8.500514
RWDD1	6.6622
RWDD2A	5.702028
RWDD2B	7.564274
RWDD3	3.601686
RWDD4	5.501724
RWDD4	5.595806
RXFP1	2.300728
RXFP2	2.394452
RXFP3	4.882238
RXFP4	3.438092
RXRA	7.198212
RXRB	6.474784
RXRB	6.512994
RXRB	6.481078

RXRG	3.074718
RYBP	6.430656
RYK	9.032984
RYR1	4.458264
RYR2	4.304546
RYR3	2.980124
S100A1	3.523578
S100A10	11.7657
S100A11	9.921232
S100A12	2.565122
S100A13	5.48427
S100A14	10.0245
S100A16	9.897022
S100A2	4.588238
S100A3	4.27235
S100A4	7.140744
S100A5	3.02801
S100A6	8.773196
S100A7	4.824948
S100A7A	2.732548
S100A7L2	2.321024
S100A8	7.110014
S100A9	6.5726
S100B	2.761928
S100G	2.567264
S100P	8.34749
S100PBP	7.662366
S100Z	2.93469
S1PR1	2.37938
S1PR2	6.076658
S1PR3	5.45106
S1PR4	4.181084
S1PR5	6.421508
SAA1	4.414706
SAA1	2.295534
SAA2	4.453442
SAA3P	2.663898
SAA4	3.523022
SAAL1	6.555696
SAC3D1	6.970384
SACM1L	8.260222
SACS	3.58826
SAE1	9.433136

SAFB	8.405066
SAFB2	7.41239
SAG	2.733044
SAGE1	2.13098
SALL1	3.769252
SALL2	3.556506
SALL3	5.04504
SALL4	4.1164
SAMD1	6.388716
SAMD10	5.4777
SAMD11	5.213752
SAMD12	6.697912
SAMD13	2.655016
SAMD14	4.313812
SAMD15	3.2838
SAMD3	2.69648
SAMD4A	4.670892
SAMD4B	7.17209
SAMD5	3.024018
SAMD7	2.823626
SAMD8	7.17806
SAMD9	7.61617
SAMD9L	5.550718
SAMHD1	4.698094
SAMHD1	8.698352
SAMM50	8.291688
SAMSN1	2.404932
SAP130	8.102716
SAP18	9.531322
SAP30	6.99644
SAP30BP	7.728868
SAP30L	6.800238
SAR1A	9.584086
SAR1B	8.390352
SARDH	4.481052
SARM1	4.885198
SARNP	6.395456
SARS	9.593486
SARS2	5.168958
SART1	8.303962
SART3	7.958614
SASH1	5.96003
SASH3	4.818586

SASS6	4.40483
SAT1	8.523472
SAT2	6.871718
SATB1	6.297552
SATB2	4.588914
SATL1	2.687856
SAV1	8.488552
SBDS	9.962178
SBF1	6.487726
SBF2	6.74301
SBK1	5.043204
SBK2	4.6451
SBNO1	7.658628
SBNO2	6.204782
SBSN	4.110024
SC4MOL	7.89797
SC5DL	5.941624
SCAF1	5.874506
SCAI	6.144022
SCAMP1	7.096194
SCAMP2	8.697038
SCAMP3	8.254052
SCAMP4	5.688668
SCAMP5	5.310694
SCAND1	5.36848
SCAND2	4.451466
SCAND3	2.709786
SCAP	6.64304
SCAPER	4.945218
SCARA3	6.075762
SCARA5	4.119636
SCARB1	7.237338
SCARB2	7.287932
SCARF1	5.411456
SCARF2	5.18046
SCARNA1	4.249442
SCARNA10	7.823744
SCARNA11	3.60179
SCARNA12	8.224768
SCARNA13	5.526612
SCARNA14	2.85509
SCARNA15	2.22517
SCARNA16	5.811486

SCARNA17	9.470118
SCARNA17	9.850252
SCARNA22	5.37152
SCARNA23	1.989254
SCARNA4	3.499976
SCARNA5	6.327146
SCARNA6	6.791006
SCARNA7	9.848322
SCARNA8	5.047388
SCARNA9	7.04611
SCARNA9	9.837142
SCARNA9L	7.218284
SCARNA9L	5.379902
SCCPDH	7.20497
SCD	10.217866
SCD	3.291704
SCD5	5.746412
SCEL	3.853562
SCFD1	6.5567
SCFD2	6.901566
SCG2	2.578588
SCG3	2.359164
SCG5	3.113732
SCGB1A1	4.949666
SCGB1C1	4.624734
SCGB1C1	4.49989
SCGB1D1	3.032974
SCGB1D2	2.636966
SCGB1D4	2.909342
SCGB2A1	4.139206
SCGB2A2	3.537798
SCGB3A1	5.537618
SCGB3A2	3.220674
SCGBL	3.390418
SCGN	2.759564
SCHIP1	7.738762
SCIN	3.158454
SCLT1	4.90232
SCLY	5.359346
SCMH1	6.601736
SCML1	4.275834
SCML2	2.580634
SCML4	3.643122

SCN10A	3.22507
SCN11A	2.833562
SCN1A	2.600572
SCN1B	4.18838
SCN2A	2.313306
SCN2B	3.706752
SCN3A	2.504796
SCN3B	3.647922
SCN4A	4.117768
SCN4B	4.313948
SCN5A	3.628996
SCN7A	2.397324
SCN8A	3.728708
SCN9A	2.921916
SCNM1	7.999098
SCNN1A	8.45215
SCNN1B	4.488778
SCNN1D	4.707986
SCNN1G	3.70929
SCO1	7.556562
SCO2	5.46143
SCOC	5.143864
SCP2	7.06932
SCPEP1	8.964262
SCRG1	2.949872
SCRIB	6.512748
SCRN1	9.400522
SCRN2	5.798682
SCRN3	4.70946
SCRT1	3.559612
SCRT1	5.065148
SCRT2	4.639884
SCT	5.251434
SCTR	3.225178
SCUBE1	4.248522
SCUBE2	3.140444
SCUBE3	4.23866
SCXA	4.289238
SCXA	4.289238
SCYL1	7.761362
SCYL2	7.814968
SCYL3	6.91586
SCYL3	6.77393

SDAD1	7.182954
SDC1	7.790942
SDC2	3.10829
SDC3	4.710426
SDC4	10.062806
SDCBP	9.627894
SDCBP2	8.27013
SDCCAG1	2.448794
SDCCAG1	5.66654
SDCCAG1	13.06234
SDCCAG3	7.142756
SDCCAG8	6.140022
SDF2	8.953854
SDF2L1	6.310698
SDF4	7.711948
SDHA	10.46992
SDHAF2	9.043018
SDHAP1	10.084242
SDHAP2	10.62502
SDHAP2	9.67012
SDHB	8.84002
SDHC	7.362694
SDHC	10.022276
SDHD	9.509844
SDHD	6.848002
SDK1	4.043724
SDK2	3.428506
SDPR	3.29706
SDR16C5	3.250876
SDR39U1	6.795326
SDR42E1	6.225864
SDR9C7	3.473994
SDS	3.355732
SDSL	5.427776
SEBOX	3.733984
SEC1	4.277686
SEC11A	7.038228
SEC11C	6.129818
SEC13	8.378172
SEC14L1	6.791688
SEC14L2	5.606482
SEC14L3	2.867624
SEC14L4	2.703468

SEC14L4	3.798354
SEC14L5	4.081052
SEC16A	7.259032
SEC16B	3.250978
SEC22A	6.501136
SEC22B	8.367868
SEC22C	6.686098
SEC23A	7.541556
SEC23B	8.359224
SEC23IP	7.847146
SEC24A	6.724458
SEC24B	7.923084
SEC24C	7.130716
SEC24D	5.513372
SEC31A	9.769972
SEC31B	3.995974
SEC61A1	10.30738
SEC61A2	6.221494
SEC61B	9.85782
SEC61G	9.562496
SEC62	6.769908
SEC63	10.086512
SECISBP2	6.746342
SECISBP2L	5.301586
SECTM1	6.161742
SEH1L	7.971814
SEL1L	7.295628
SEL1L2	2.471574
SEL1L3	7.883502
SELE	2.640828
SELENBP1	8.215224
SELK	7.195768
SELL	5.411936
SELM	5.708894
SELO	6.074498
SELP	2.974688
SELPLG	3.683846
SELS	6.283506
SELT	9.907548
SELT	10.23436
SELV	5.030906
SEMA3A	5.550412
SEMA3B	5.180818

SEMA3C	7.4272
SEMA3D	2.412232
SEMA3E	3.522422
SEMA3F	5.313204
SEMA3G	3.676338
SEMA4A	4.480362
SEMA4B	6.113376
SEMA4C	5.606506
SEMA4D	3.945678
SEMA4D	5.706344
SEMA4F	4.635386
SEMA4F	3.823658
SEMA4G	3.864512
SEMA5A	3.272316
SEMA5B	4.504068
SEMA6A	3.115932
SEMA6B	4.988042
SEMA6C	3.870674
SEMA6D	3.177874
SEMA7A	5.317726
SEMG1	2.526958
SEMG2	2.678396
SENP1	7.498874
SENP2	8.786614
SENP3	7.650602
SENP5	9.121888
SENP6	6.840548
SENP7	2.81875
SENP8	4.286718
15-Sep	9.22922
SEPHS1	8.949778
SEPHS2	7.72038
SEPN1	6.726634
SEPP1	2.363814
SEPSECS	5.656184
1-Sep	3.64337
10-Sep	6.472012
10-Sep	6.596788
11-Sep	7.2129
12-Sep	4.06779
14-Sep	4.293966
14-Sep	5.325628
14-Sep	4.293966

14-Sep	4.293966
14-Sep	4.165714
14-Sep	4.293966
14-Sep	4.293966
14-Sep	4.293966
14-Sep	4.293966
14-Sep	3.333714
14-Sep	4.293966
2-Sep	8.14735
3-Sep	4.87156
4-Sep	4.009512
5-Sep	5.648456
5-Sep	5.044448
6-Sep	4.996244
7-Sep	7.403494
SEPT7L	5.716396
8-Sep	6.89811
9-Sep	7.25128
SEPW1	6.953854
SEPX1	7.339586
SERAC1	5.223764
SERBP1	6.644884
SERF1A	4.940588
SERF1A	4.940588
SERF1A	4.940588
SERF2	6.087556
SERGEF	5.181312
SERHL	3.743952
SERHL2	2.870024
SERINC1	9.143878
SERINC2	8.307886
SERINC3	9.700608
SERINC4	3.067102
SERINC5	8.469548
SERP1	8.984012
SERP2	3.122488
SERPINA1	5.359916
SERPINA10	3.923096
SERPINA11	3.506038
SERPINA12	3.354964
SERPINA13	3.290366
SERPINA2	3.236346
SERPINA3	9.373104

SERPINA4	3.353924
SERPINA5	5.272682
SERPINA6	2.877388
SERPINA7	2.266358
SERPINA9	2.989762
SERPINB1	8.766396
SERPINB10	2.773608
SERPINB11	2.417022
SERPINB12	2.209214
SERPINB13	2.859124
SERPINB2	5.374566
SERPINB3	2.680854
SERPINB4	2.551842
SERPINB5	7.126838
SERPINB6	7.709852
SERPINB7	4.548854
SERPINB8	4.421818
SERPINB9	3.47342
SERPINC1	3.359536
SERPIND1	2.947174
SERPINE1	5.874428
SERPINE2	4.408468
SERPINF1	2.990238
SERPINF2	4.296616
SERPING1	4.202364
SERPINH1	8.090706
SERPINI1	2.636054
SERPINI2	2.5362
SERTAD1	6.287684
SERTAD2	6.058486
SERTAD3	5.891026
SERTAD4	4.390908
SESN1	5.792148
SESN2	5.69596
SESN3	2.4613
SESTD1	7.0103
SET	10.3311
SET	10.42342
SETBP1	4.432324
SETD1A	6.441456
SETD1B	5.830664
SETD2	6.797554
SETD3	7.25882

SETD4	5.883262
SETD5	9.029112
SETD6	5.709832
SETD7	8.336112
SETD8	8.289844
SETD8	5.72717
SETD8	8.419126
SETDB1	6.334758
SETDB2	3.728626
SETMAR	5.460822
SETX	7.246962
SEZ6	3.489104
SEZ6L	3.380422
SEZ6L2	6.76619
SF1	7.972272
SF3A1	7.540024
SF3A2	7.372816
SF3A3	9.250192
SF3B1	9.661798
SF3B14	8.458164
SF3B2	9.373366
SF3B3	10.089754
SF3B4	6.46705
SF3B5	7.139366
SFI1	5.654838
SFMBT1	6.586238
SFMBT2	3.66788
SFN	9.890114
SFPQ	9.468148
SFRP1	7.174384
SFRP2	3.50527
SFRP4	2.804938
SFRP5	4.422966
SFRS15	7.620344
SFRS18	7.369328
SFSWAP	7.576174
SFT2D1	7.219668
SFT2D2	8.609014
SFT2D3	6.44908
SFTA2	3.909468
SFTA2	3.993278
SFTPA1	3.386626
SFTPA1	3.386626

SFTPA2	4.125946
SFTPA2	4.125946
SFTPB	4.169428
SFTPC	4.650038
SFTPD	4.959342
SFXN1	9.245934
SFXN2	5.910206
SFXN3	5.683002
SFXN4	6.480232
SFXN5	5.362616
SGCA	4.650932
SGCB	3.419026
SGCD	2.997968
SGCE	8.535346
SGCG	2.557388
SGCZ	2.712722
SGIP1	2.780762
SGK1	5.458052
SGK196	8.375532
SGK2	3.912086
SGK223	5.677492
SGK269	5.589566
SGK3	6.09132
SGK494	5.072654
SGMS1	5.197812
SGMS2	6.02757
SGOL1	7.124808
SGOL2	4.397762
SGPL1	8.125586
SGPP1	6.526866
SGPP2	6.045512
SGSH	6.041496
SGSM1	5.008006
SGSM2	5.684504
SGSM3	5.702572
SGTA	7.182488
SGTB	4.498292
SH2B1	5.006562
SH2B2	4.465394
SH2B3	5.58612
SH2D1A	2.960786
SH2D1B	2.829922
SH2D2A	4.714106

SH2D3A	5.66402
SH2D3C	4.522406
SH2D4A	7.162186
SH2D4B	3.72898
SH2D5	3.937008
SH2D6	4.140778
SH2D7	3.198146
SH3BGR	3.303372
SH3BGRL	7.200858
SH3BGRL2	3.371546
SH3BGRL3	7.47843
SH3BP1	6.083628
SH3BP1	5.74606
SH3BP2	5.47693
SH3BP4	5.645526
SH3BP5	6.530686
SH3BP5L	6.539758
SH3D19	6.370388
SH3D20	5.070722
SH3GL1	6.75694
SH3GL2	6.641096
SH3GL3	3.123856
SH3GLB1	6.799992
SH3GLB2	8.376982
SH3KBP1	11.87682
SH3KBP1	7.554146
SH3PXD2A	4.723274
SH3PXD2B	5.977172
SH3RF1	6.957044
SH3RF2	6.888696
SH3RF3	5.121332
SH3TC1	4.93501
SH3TC2	6.703784
SH3YL1	6.123238
SHANK1	3.884826
SHANK2	5.27109
SHANK3	4.871242
SHARPIN	7.966934
SHB	6.139484
SHBG	3.8102
SHC1	7.819104
SHC2	4.374618
SHC3	7.20899

SHC4	2.856214
SHCBP1	9.145326
SHD	4.033384
SHE	3.982308
SHF	4.428052
SHFM1	8.289686
SHH	4.499458
SHISA2	5.920178
SHISA3	3.407422
SHISA4	6.54045
SHISA5	9.7888
SHISA6	3.739876
SHISA6	2.639788
SHISA7	3.156908
SHISA7	5.86317
SHISA9	4.953628
SHKBP1	6.562748
SHMT1	8.460348
SHMT2	9.373954
SHOC2	6.649328
SHOX	3.674664
SHOX	3.674664
SHOX2	6.189752
SHPK	6.397608
SHPRH	5.104032
SHQ1	6.787954
SHROOM1	5.376278
SHROOM2	3.998386
SHROOM3	6.235574
SHROOM4	3.340764
SI	2.416078
SIAE	8.753412
SIAE	5.479962
SIAH1	6.443908
SIAH2	7.354318
SIAH3	3.614906
SIDT1	3.988336
SIDT2	6.290548
SIGIRR	6.166864
SIGLEC1	4.116222
SIGLEC10	3.600818
SIGLEC11	2.960704
SIGLEC12	4.82905

SIGLEC14	3.93646
SIGLEC15	3.974338
SIGLEC16	3.562786
SIGLEC5	4.178594
SIGLEC6	3.488572
SIGLEC7	3.14638
SIGLEC8	4.654924
SIGLEC9	3.429034
SIGLECP3	3.016318
SIGMAR1	6.57117
SIK1	6.375504
SIK2	6.931458
SIK3	6.099894
SIKE1	6.131918
SIL1	6.078052
SILV	4.791188
SIM1	5.87747
SIM2	5.577088
SIN3A	7.6533
SIN3B	6.998414
SIP1	5.420698
SIPA1	6.06893
SIPA1L1	6.616848
SIPA1L2	5.537592
SIPA1L3	5.739214
SIRPA	5.063266
SIRPB1	6.004004
SIRPB2	3.716346
SIRPD	3.962726
SIRPG	3.67631
SIRT1	6.965498
SIRT2	7.357238
SIRT3	6.524796
SIRT4	3.453316
SIRT5	8.329238
SIRT6	5.428632
SIRT7	7.57065
SIT1	3.276966
SIVA1	6.26217
SIX1	6.441302
SIX2	6.116752
SIX3	4.011228
SIX4	7.02967

SIX5	5.694628
SIX6	5.07404
SKA1	5.13051
SKA2	9.104158
SKA2	9.19143
SKA3	7.463774
SKAP1	2.930454
SKAP2	7.627004
SKCG-1	2.687624
SKI	6.3937
SKIL	7.154914
SKIV2L	6.826254
SKIV2L	6.826254
SKIV2L	6.948238
SKIV2L2	7.706192
SKOR1	5.516496
SKOR2	4.84576
SKP1	6.41571
SKP2	8.90524
SLA	2.815786
SLA2	3.403424
SLAIN1	2.951794
SLAIN2	8.285164
SLAMF1	2.50818
SLAMF6	2.608626
SLAMF7	2.48623
SLAMF8	3.107074
SLAMF9	3.486528
SLBP	7.028274
SLC10A1	3.79484
SLC10A2	3.697412
SLC10A3	6.740314
SLC10A4	4.957476
SLC10A5	2.502226
SLC10A6	3.127712
SLC10A7	4.944474
SLC11A1	4.017726
SLC11A2	8.200758
SLC12A1	2.68837
SLC12A2	6.019614
SLC12A3	4.549874
SLC12A4	6.111832
SLC12A5	3.04442

SLC12A6	5.885874
SLC12A7	6.059106
SLC12A8	6.627996
SLC12A9	6.13772
SLC13A1	2.56614
SLC13A2	4.750866
SLC13A3	3.651546
SLC13A4	3.881842
SLC13A5	4.833272
SLC14A1	2.50452
SLC14A2	3.213238
SLC15A1	3.653364
SLC15A2	6.276276
SLC15A3	7.121696
SLC15A4	6.977724
SLC15A5	2.931008
SLC16A1	8.949766
SLC16A10	3.523238
SLC16A11	4.203558
SLC16A12	2.61123
SLC16A13	7.257714
SLC16A14	6.53094
SLC16A2	4.156598
SLC16A3	7.621802
SLC16A4	4.48045
SLC16A5	6.776206
SLC16A6	6.454456
SLC16A7	7.1898
SLC16A8	5.187286
SLC16A9	3.004038
SLC17A1	2.522912
SLC17A2	2.90581
SLC17A3	2.435186
SLC17A4	2.66515
SLC17A5	7.268128
SLC17A6	2.627746
SLC17A7	3.755078
SLC17A8	3.138954
SLC17A9	4.502762
SLC18A1	3.114938
SLC18A2	3.031924
SLC18A3	4.040334
SLC19A1	6.234852

SLC19A2	7.411896
SLC19A3	5.126996
SLC1A1	3.09379
SLC1A2	2.91105
SLC1A3	4.54006
SLC1A4	5.15578
SLC1A5	5.452558
SLC1A5	8.462292
SLC1A6	4.312266
SLC1A7	3.57656
SLC20A1	8.327678
SLC20A2	8.407036
SLC22A1	3.87155
SLC22A10	3.746066
SLC22A11	4.62108
SLC22A12	3.734102
SLC22A13	3.561402
SLC22A14	3.106628
SLC22A15	2.86692
SLC22A16	2.98977
SLC22A17	5.044686
SLC22A18	6.271234
SLC22A18AS	3.227252
SLC22A2	3.03519
SLC22A20	5.270854
SLC22A23	5.398486
SLC22A24	2.58127
SLC22A25	3.115804
SLC22A3	2.52555
SLC22A4	4.844082
SLC22A5	6.565098
SLC22A6	4.1418
SLC22A7	3.38564
SLC22A8	3.74007
SLC22A9	3.569922
SLC23A1	6.229404
SLC23A2	7.285152
SLC23A3	4.115402
SLC24A1	4.45534
SLC24A2	2.93913
SLC24A3	4.392976
SLC24A4	3.165746
SLC24A5	2.64607

SLC24A6	6.14438
SLC25A1	7.82693
SLC25A10	7.082832
SLC25A11	8.28299
SLC25A12	6.785772
SLC25A13	8.130052
SLC25A14	5.747398
SLC25A15	6.987644
SLC25A16	4.509128
SLC25A17	7.142806
SLC25A18	4.410148
SLC25A19	5.56565
SLC25A2	3.681412
SLC25A20	6.075044
SLC25A21	2.578732
SLC25A22	6.259362
SLC25A23	6.395858
SLC25A24	6.29345
SLC25A25	5.993626
SLC25A26	6.87035
SLC25A27	3.937476
SLC25A28	6.848468
SLC25A29	6.438544
SLC25A3	9.53878
SLC25A30	4.412586
SLC25A31	2.783882
SLC25A32	8.778336
SLC25A33	6.774946
SLC25A33	6.65633
SLC25A34	4.567624
SLC25A35	5.725674
SLC25A36	6.380004
SLC25A37	7.594594
SLC25A37	6.957652
SLC25A38	7.069754
SLC25A39	8.336792
SLC25A4	7.896982
SLC25A40	7.384886
SLC25A41	3.761468
SLC25A42	6.120454
SLC25A43	5.955544
SLC25A44	6.774148
SLC25A45	4.909032

SLC25A46	6.734164
SLC25A47	4.241272
SLC25A48	4.582968
SLC25A5	9.819068
SLC25A5	4.966936
SLC25A6	9.524694
SLC25A6	9.524694
SLC26A1	4.447828
SLC26A10	3.811634
SLC26A11	5.459182
SLC26A2	4.72044
SLC26A3	2.56356
SLC26A4	2.682832
SLC26A5	2.745994
SLC26A6	5.90079
SLC26A7	2.280098
SLC26A8	3.12529
SLC26A9	3.296158
SLC27A1	5.517226
SLC27A2	7.098802
SLC27A3	4.240164
SLC27A4	7.12875
SLC27A5	4.529196
SLC27A6	2.487468
SLC28A1	3.51233
SLC28A2	3.044136
SLC28A3	4.157406
SLC29A1	6.40728
SLC29A2	6.628908
SLC29A3	5.894724
SLC29A4	4.39689
SLC2A1	9.637272
SLC2A10	5.01793
SLC2A11	5.900648
SLC2A12	5.160486
SLC2A13	3.234538
SLC2A14	4.06148
SLC2A2	2.94935
SLC2A3	2.804176
SLC2A4	4.790624
SLC2A4RG	6.429982
SLC2A5	4.374536
SLC2A6	6.420334

SLC2A7	2.647576
SLC2A8	6.009574
SLC2A9	3.648874
SLC30A1	6.113156
SLC30A10	2.364314
SLC30A2	3.890582
SLC30A3	4.784356
SLC30A4	5.347406
SLC30A5	6.60446
SLC30A6	7.847256
SLC30A7	7.0761
SLC30A8	2.784358
SLC30A9	7.515776
SLC31A1	9.23948
SLC31A2	7.667136
SLC32A1	3.727202
SLC33A1	8.207834
SLC34A1	4.147356
SLC34A2	4.171004
SLC34A3	4.17075
SLC35A1	6.89314
SLC35A2	6.643238
SLC35A3	6.652512
SLC35A4	8.80386
SLC35A5	8.533938
SLC35B1	8.068392
SLC35B2	7.734046
SLC35B3	6.02253
SLC35B4	5.257798
SLC35C1	6.954298
SLC35C2	7.652416
SLC35D1	5.2073
SLC35D2	6.91023
SLC35D3	4.741478
SLC35E1	8.850698
SLC35E2B	5.391504
SLC35E3	6.311204
SLC35E4	4.15622
SLC35F1	2.85924
SLC35F2	8.443024
SLC35F3	5.814476
SLC35F4	2.658976
SLC35F5	8.151354

SLC36A1	6.078966
SLC36A2	3.072192
SLC36A3	2.861216
SLC36A4	5.22664
SLC37A1	7.179356
SLC37A2	4.136676
SLC37A3	7.213848
SLC37A4	6.705762
SLC38A1	9.765906
SLC38A10	7.07927
SLC38A11	2.758448
SLC38A2	10.66502
SLC38A3	3.34123
SLC38A4	2.508388
SLC38A5	3.194186
SLC38A6	5.748534
SLC38A7	5.322426
SLC38A8	3.824022
SLC38A9	6.088574
SLC39A1	8.739706
SLC39A10	6.697708
SLC39A11	7.481236
SLC39A12	2.676666
SLC39A13	5.858604
SLC39A14	6.548564
SLC39A2	2.988616
SLC39A3	5.706132
SLC39A4	5.80499
SLC39A5	4.005532
SLC39A6	6.010752
SLC39A7	8.978014
SLC39A7	8.978014
SLC39A7	8.978014
SLC39A8	9.1125
SLC39A9	8.987026
SLC3A1	3.058852
SLC3A2	10.091494
SLC40A1	2.953914
SLC41A1	5.96076
SLC41A2	7.161098
SLC41A3	6.669424
SLC43A1	5.76439
SLC43A2	6.913282

SLC43A3	8.272874
SLC44A1	9.02667
SLC44A2	10.192734
SLC44A3	5.548654
SLC44A4	4.56626
SLC44A4	4.56626
SLC44A4	4.56626
SLC44A5	2.80074
SLC45A1	3.74782
SLC45A2	2.807062
SLC45A3	5.891058
SLC45A4	5.704704
SLC45A4	6.64074
SLC46A1	6.016964
SLC46A2	4.10958
SLC46A3	7.397466
SLC47A1	5.189534
SLC47A2	3.653784
SLC48A1	6.976032
SLC4A1	3.482702
SLC4A10	2.688698
SLC4A11	8.2768
SLC4A1AP	8.140232
SLC4A2	7.464758
SLC4A3	4.803672
SLC4A4	2.746122
SLC4A5	2.924548
SLC4A7	5.74266
SLC4A8	2.686932
SLC4A9	3.691322
SLC5A1	2.633714
SLC5A10	4.205096
SLC5A10	3.340728
SLC5A11	3.61718
SLC5A12	2.678278
SLC5A2	4.465258
SLC5A3	6.20509
SLC5A3	6.28283
SLC5A4	2.625854
SLC5A5	3.5214
SLC5A6	9.138964
SLC5A7	2.810336
SLC5A8	2.66398

SLC5A9	3.115192
SLC6A1	3.508292
SLC6A11	6.93025
SLC6A12	3.7683
SLC6A13	3.489284
SLC6A14	9.461024
SLC6A15	2.353946
SLC6A16	3.780064
SLC6A17	4.238136
SLC6A18	4.049682
SLC6A19	4.08632
SLC6A2	3.737514
SLC6A20	4.239964
SLC6A3	4.74271
SLC6A4	3.287656
SLC6A5	3.17653
SLC6A6	7.77267
SLC6A7	3.57187
SLC6A8	4.556506
SLC6A8	4.652156
SLC6A8	6.879572
SLC6A9	6.967098
SLC7A1	9.16928
SLC7A10	5.647426
SLC7A11	7.174562
SLC7A13	2.110522
SLC7A14	3.095756
SLC7A14	2.848504
SLC7A2	2.90235
SLC7A3	3.516238
SLC7A4	4.028666
SLC7A5	10.291032
SLC7A5P2	9.279028
SLC7A6	7.124736
SLC7A6OS	6.829716
SLC7A7	2.756846
SLC7A8	3.679452
SLC7A9	3.707902
SLC8A1	3.38823
SLC8A2	3.544262
SLC8A3	3.148648
SLC9A1	6.502484
SLC9A10	2.305334

SLC9A11	2.488104
SLC9A2	2.922852
SLC9A3	3.850404
SLC9A3R1	9.301056
SLC9A3R2	6.97224
SLC9A4	2.885366
SLC9A5	3.482862
SLC9A6	8.617158
SLC9A7	5.9986
SLC9A8	7.335808
SLC9A9	2.481116
SLCO1A2	2.359952
SLCO1B1	3.025028
SLCO1B3	7.678518
SLCO1C1	2.75504
SLCO2A1	4.067408
SLCO2B1	2.93524
SLCO3A1	8.273124
SLCO4A1	7.68395
SLCO4A1	4.652208
SLCO4C1	3.089096
SLCO4C1	2.264366
SLCO5A1	3.247614
SLCO5A1	4.891804
SLCO6A1	2.375618
SLED1	4.168798
SLFN11	2.51518
SLFN12	6.144292
SLFN12L	4.435696
SLFN13	2.993938
SLFN14	2.292112
SLFN5	8.232822
SLFNL1	3.714946
SLIT1	3.153404
SLIT2	6.657324
SLIT3	3.678666
SLITRK1	3.10903
SLITRK2	3.6286
SLITRK3	2.644572
SLITRK4	2.829934
SLITRK5	4.231952
SLITRK6	3.961112
SLK	6.746452

SLMAP	6.67346
SLMO1	6.094064
SLMO2	8.746814
SLN	3.016066
SLPI	10.9036
SLTM	7.30035
SLU7	7.313532
SLURP1	5.497416
SLX4	5.44175
SMA5	6.300912
SMA5	8.291198
SMA5	7.069394
SMA5	6.677874
SMA5	8.01436
SMAD1	6.312892
SMAD2	5.195516
SMAD2	7.094036
SMAD3	7.848858
SMAD4	6.901404
SMAD5	6.3385
SMAD5OS	3.050938
SMAD6	5.722932
SMAD7	4.3821
SMAD9	4.412474
SMAGP	7.661432
SMAP1	7.626132
SMAP2	6.255386
SMARCA1	2.797958
SMARCA2	6.80561
SMARCA4	7.384226
SMARCA5	8.944672
SMARCAD1	6.024796
SMARCAL1	6.637122
SMARCB1	7.84533
SMARCC1	8.19161
SMARCC2	8.273606
SMARCD1	6.598822
SMARCD2	7.675638
SMARCD3	4.903074
SMARCE1	3.44825
SMARCE1	8.38105
SMC1A	7.604708
SMC1B	2.5385

SMC2	6.897124
SMC3	6.435844
SMC4	8.671406
SMC5	6.483148
SMC6	6.011786
SMCHD1	8.584936
SMCHD1	7.987324
SMCP	2.797418
SMCR5	3.204166
SMCR7	6.412602
SMCR7L	6.911312
SMCR8	6.03405
SMEK1	7.405556
SMEK2	7.729872
SMEK3P	2.912632
SMG1	8.74181
SMG1	9.745532
SMG1	8.285114
SMG1	10.7416
SMG1	8.74181
SMG1	9.631026
SMG1	9.284424
SMG1	8.841408
SMG1	9.062272
SMG1	9.36033
SMG1	9.00152
SMG5	7.515096
SMG6	5.93048
SMG7	8.235064
SMN1	6.883248
SMN1	7.035976
SMN1	6.883248
SMNDC1	7.28837
SMO	4.572662
SMOC1	6.611704
SMOC2	4.038178
SMOX	7.805096
SMPD1	4.670926
SMPD2	7.143162
SMPD3	3.34278
SMPD4	6.851096
SMPD4	8.52092
SMPDL3A	2.883644

SMPDL3B	6.880012
SMPX	2.574724
SMR3A	2.992708
SMR3B	2.296402
SMS	7.839818
SMTN	5.50714
SMTN	4.145928
SMTNL2	5.21537
SMU1	9.55017
SMUG1	6.112702
SMURF1	7.950576
SMURF2	7.550484
SMYD1	2.53954
SMYD2	4.821182
SMYD3	5.696586
SMYD4	6.936844
SMYD5	5.958546
SNAI1	4.209002
SNAI2	6.206556
SNAI3	5.31502
SNAP23	7.102284
SNAP25	3.369402
SNAP29	7.186068
SNAP47	6.308296
SNAP91	3.410282
SNAPC1	5.239478
SNAPC2	6.418106
SNAPC3	7.288036
SNAPC4	5.612542
SNAPC5	6.957614
SNAPIN	6.812832
SNCA	2.825988
SNCAIP	3.676412
SNCB	4.132454
SNCG	5.519924
SND1	8.881286
SNED1	3.99047
SNF8	7.737488
SNHG11	5.460572
SNHG12	7.803312
SNIP1	7.159398
SNN	5.583272
SNORA1	4.95731

SNORA10	6.505014
SNORA10	6.505014
SNORA13	3.085502
SNORA14A	4.004734
SNORA14B	5.67596
SNORA15	4.913668
SNORA15	4.892362
SNORA15	4.892362
SNORA16A	7.186154
SNORA16B	2.663352
SNORA19	2.64411
SNORA20	5.635182
SNORA21	8.106786
SNORA22	8.697704
SNORA23	6.972292
SNORA24	8.049482
SNORA27	5.887578
SNORA28	3.780038
SNORA29	3.556318
SNORA2A	2.833696
SNORA2B	4.693546
SNORA3	6.853614
SNORA30	3.105488
SNORA31	5.726968
SNORA33	5.402314
SNORA35	3.730694
SNORA36A	2.968034
SNORA36C	3.09488
SNORA37	4.448264
SNORA38	5.724356
SNORA38B	4.976662
SNORA4	8.332316
SNORA40	6.239922
SNORA40	3.95469
SNORA41	3.327222
SNORA42	6.014842
SNORA44	5.416176
SNORA45	5.23081
SNORA46	4.866622
SNORA48	8.665882
SNORA49	6.09943
SNORA50	6.54793
SNORA51	4.201516

SNORA52	5.412016
SNORA54	4.204476
SNORA55	5.357124
SNORA56	4.555396
SNORA57	7.170028
SNORA58	2.74927
SNORA59A	5.200818
SNORA59A	5.200818
SNORA5A	5.114192
SNORA5B	3.178176
SNORA5C	6.070838
SNORA6	5.61127
SNORA60	4.615762
SNORA61	8.07109
SNORA62	5.50384
SNORA64	7.489258
SNORA65	7.056632
SNORA67	7.149316
SNORA68	8.656286
SNORA69	2.361266
SNORA70	9.747172
SNORA70	9.65533
SNORA70B	2.69706
SNORA70C	3.231956
SNORA70D	4.175944
SNORA70E	3.093916
SNORA70F	2.579088
SNORA70G	4.109228
SNORA71A	7.143262
SNORA71B	6.72457
SNORA71C	7.96269
SNORA71D	7.3968
SNORA73A	10.03148
SNORA74A	4.957358
SNORA75	4.49404
SNORA7B	6.08774
SNORA8	5.996784
SNORA80	5.622332
SNORA80B	5.68735
SNORA9	6.250592
SNORD101	6.225586
SNORD102	3.64836
SNORD103A	3.471566

SNORD103A	3.471566
SNORD104	7.479646
SNORD105	2.637712
SNORD109A	2.401506
SNORD109A	2.401506
SNORD113-3	2.123988
SNORD113-4	2.205434
SNORD114-2	2.2621
SNORD114-2	2.492876
SNORD114-3	2.85436
SNORD114-6	2.071928
SNORD115-1	2.207514
SNORD115-1	2.207514
SNORD115-1	2.207514
SNORD115-1	1.909624
SNORD115-1	2.21827
SNORD115-1	2.216288
SNORD115-1	2.21827
SNORD115-1	2.21827
SNORD115-1	2.291094
SNORD115-1	2.21827
SNORD115-1	2.21527
SNORD115-1	2.21527
SNORD115-1	2.21527
SNORD115-1	2.100154
SNORD115-1	1.804866
SNORD115-1	2.159556
SNORD115-1	2.159556
SNORD115-1	2.159556
SNORD115-2	2.029496
SNORD115-2	2.215754
SNORD115-2	1.991216
SNORD115-2	2.318982
SNORD115-2	2.12683
SNORD115-2	2.223194
SNORD115-3	1.868516
SNORD115-3	1.922718
SNORD115-3	1.996806
SNORD115-3	2.467756
SNORD115-3	2.214716
SNORD115-3	2.058932
SNORD115-3	2.288176
SNORD115-3	2.022502

SNORD115-3	2.335674
SNORD115-4	1.74196
SNORD115-4	1.973692
SNORD115-4	1.975004
SNORD115-4	2.15848
SNORD115-4	2.30026
SNORD115-4	2.234932
SNORD115-6	2.154516
SNORD115-7	1.934892
SNORD115-8	2.058698
SNORD116-1	3.224944
SNORD116-1	2.081352
SNORD116-1	2.56519
SNORD116-1	2.302832
SNORD116-1	2.117746
SNORD116-1	6.906676
SNORD116-1	7.977722
SNORD116-1	1.95039
SNORD116-1	7.521644
SNORD116-1	7.521644
SNORD116-1	2.197494
SNORD116-2	3.16963
SNORD116-2	5.487542
SNORD116-2	5.724756
SNORD116-2	2.545932
SNORD116-2	3.41857
SNORD116-2	5.473396
SNORD116-2	2.964982
SNORD116-2	2.079886
SNORD116-2	2.88565
SNORD116-2	2.496684
SNORD116-2	2.701338
SNORD116-3	5.757424
SNORD116-3	5.757424
SNORD116-4	3.83064
SNORD116-5	4.737216
SNORD116-5	4.737216
SNORD116-6	2.360658
SNORD116-8	5.872586
SNORD117	4.76663
SNORD12C	6.19726
SNORD13	8.75528
SNORD13	8.85073

SNORD13P1	1.91246
SNORD13P2	5.312352
SNORD14C	7.047876
SNORD14E	4.803402
SNORD15A	6.035922
SNORD15B	6.521902
SNORD1A	4.590278
SNORD1B	3.114004
SNORD1C	7.99847
SNORD20	2.492034
SNORD21	5.767502
SNORD22	5.744914
SNORD24	7.088166
SNORD25	5.420248
SNORD26	8.51094
SNORD27	6.228
SNORD28	9.12727
SNORD29	7.859324
SNORD30	7.64266
SNORD31	6.099588
SNORD32A	6.801694
SNORD32B	3.700118
SNORD33	10.88664
SNORD34	5.948024
SNORD35A	7.679498
SNORD36B	5.564542
SNORD36C	6.412124
SNORD37	2.989562
SNORD38A	4.27495
SNORD38B	4.32158
SNORD3A	11.029382
SNORD3A	11.029382
SNORD3A	11.029382
SNORD3A	11.029382
SNORD3A	11.029382
SNORD41	8.135144
SNORD42A	3.87099
SNORD42B	6.239102
SNORD43	6.979764
SNORD44	8.053492
SNORD45A	8.931668
SNORD45B	6.044092
SNORD45C	5.660852

SNORD46	8.598176
SNORD47	7.008832
SNORD49A	6.1069
SNORD49B	6.779542
SNORD4A	5.391492
SNORD4B	6.181502
SNORD5	6.735536
SNORD50A	7.903472
SNORD50B	6.189962
SNORD51	3.398976
SNORD52	6.743968
SNORD53	5.507596
SNORD54	5.994376
SNORD55	5.960358
SNORD56B	2.348904
SNORD57	9.377208
SNORD58A	3.002286
SNORD59A	2.529506
SNORD59B	4.848328
SNORD6	5.209636
SNORD60	6.575432
SNORD61	3.920646
SNORD62A	4.629066
SNORD62A	4.629066
SNORD63	3.766138
SNORD68	5.583142
SNORD7	3.836882
SNORD73A	6.118358
SNORD74	8.688036
SNORD75	7.784408
SNORD76	9.560818
SNORD77	3.699222
SNORD78	8.111192
SNORD79	6.201884
SNORD8	7.132778
SNORD80	6.655962
SNORD82	2.905698
SNORD83A	4.300196
SNORD83B	5.224516
SNORD94	6.76941
SNORD95	9.327906
SNORD96A	5.77246
SNORD96B	3.363224

SNRK	6.99877
SNRNP200	9.6666
SNRNP25	6.620526
SNRNP27	7.651598
SNRNP35	4.35899
SNRNP40	8.297142
SNRNP48	6.477424
SNRNP70	8.77794
SNRPA	8.315828
SNRPA1	4.603878
SNRPB	9.770206
SNRPB2	7.352646
SNRPC	7.70001
SNRPD1	7.948286
SNRPD2	7.929128
SNRPD2P2	2.730922
SNRPD3	10.022506
SNRPE	5.12976
SNRPE	8.329136
SNRPF	6.769052
SNRPG	8.183108
SNRPG	6.091632
SNRPN	5.535722
SNRPN	1.914522
SNTA1	5.669858
SNTB1	4.271278
SNTB2	7.140128
SNTG1	2.960614
SNTG2	3.446422
SNTN	2.296904
SNUPN	7.203186
SNW1	8.678504
SNX1	6.649224
SNX10	5.387272
SNX11	6.568672
SNX12	6.59948
SNX13	6.749016
SNX14	7.358696
SNX15	6.043432
SNX16	4.788984
SNX17	8.280102
SNX18	5.368676
SNX19	6.853208

SNX2	5.448854
SNX20	3.167388
SNX21	5.099966
SNX22	4.98727
SNX24	5.611514
SNX25	4.76552
SNX27	6.819416
SNX29	6.05104
SNX3	7.78223
SNX3	3.044734
SNX30	6.953594
SNX31	3.125078
SNX32	2.675124
SNX33	6.60685
SNX4	6.742738
SNX5	7.379028
SNX6	7.918006
SNX7	5.648174
SNX8	7.599378
SNX9	7.15852
SOAT1	8.083774
SOAT2	4.134152
SOBP	3.50758
SOCS1	4.928968
SOCS2	3.813376
SOCS3	4.729076
SOCS4	5.80407
SOCS5	7.06793
SOCS6	7.252764
SOCS7	5.940158
SOD1	10.4144
SOD2	8.971492
SOD3	3.977268
SOHLH1	4.647416
SOHLH2	2.745202
SOLH	5.816564
SON	8.196648
SORBS1	4.69315
SORBS2	3.103746
SORBS2	2.485244
SORBS3	5.611294
SORCS1	3.107134
SORCS2	4.826166

SORCS3	3.217504
SORD	7.683244
SORL1	4.438004
SORT1	7.990212
SOS1	6.658224
SOS2	7.07745
SOST	4.964574
SOSTDC1	2.646226
SOX1	4.780002
SOX10	5.478008
SOX11	5.107932
SOX12	5.902368
SOX13	5.528848
SOX14	4.18329
SOX15	4.604134
SOX17	5.362926
SOX18	5.889154
SOX2	3.483036
SOX21	4.965352
SOX3	6.17277
SOX30	3.436752
SOX4	6.644102
SOX5	2.882942
SOX6	3.081206
SOX7	4.86838
SOX8	5.397204
SOX9	6.705604
SP1	8.12506
SP100	6.267462
SP110	6.20439
SP140	3.283
SP140L	4.862116
SP2	5.499266
SP3	6.567232
SP3P	3.508728
SP4	6.483008
SP5	5.216376
SP6	6.268802
SP7	4.92833
SP8	4.172708
SP9	5.430992
SPA17	4.97418
SPACA1	2.527364

SPACA3	3.325962
SPACA4	2.647376
SPACA5	3.271918
SPACA5	3.40023
SPAG1	5.797602
SPAG11B	3.01742
SPAG11B	2.583474
SPAG16	4.343328
SPAG17	2.205462
SPAG4	3.963044
SPAG5	8.307162
SPAG6	2.907876
SPAG7	6.174676
SPAG8	3.082534
SPAG9	8.349962
SPAM1	2.395812
SPANXA2	2.91723
SPANXA2	2.91723
SPANXB1	2.590998
SPANXB1	2.590998
SPANXE	3.076026
SPANXE	3.350488
SPANXN1	2.752238
SPANXN2	2.589656
SPANXN3	2.281058
SPANXN4	2.905634
SPANXN5	3.082918
SPARC	9.665604
SPARCL1	2.716586
SPAST	6.667754
SPATA1	2.355052
SPATA12	3.332674
SPATA13	5.59596
SPATA16	2.56875
SPATA17	3.649468
SPATA18	3.009458
SPATA19	2.764226
SPATA2	5.629522
SPATA20	6.20503
SPATA21	4.119968
SPATA22	2.37237
SPATA24	4.557826
SPATA2L	5.379768

SPATA3	3.367834
SPATA4	2.642852
SPATA5	6.892806
SPATA5L1	4.93918
SPATA6	3.185174
SPATA7	3.746374
SPATA8	2.425668
SPATA9	2.387474
SPATC1	4.706962
SPATS1	3.100722
SPATS2	7.46278
SPATS2L	9.752266
SPC24	6.753746
SPC25	7.454348
SPCS1	8.933076
SPCS2	9.197824
SPCS2	9.614392
SPCS3	7.84543
SPDEF	5.441698
SPDYA	2.429322
SPDYC	4.703154
SPDYE1	4.638156
SPDYE1	4.779232
SPDYE1	4.688944
SPDYE2	4.296886
SPDYE2	4.240598
SPDYE2	4.429758
SPDYE3	2.78466
SPDYE4	3.708482
SPDYE5	4.752272
SPDYE7P	4.624456
SPDYE7P	4.16216
SPDYE8P	4.638198
SPDYE8P	4.373226
SPDYE8P	4.39982
SPDYE8P	4.544374
SPDYE8P	4.337298
SPDYE8P	4.544374
SPDYE8P	4.391014
SPDYE8P	4.355602
SPECC1	7.238882
SPECC1L	7.41172
SPEF1	4.485214

SPEF2	2.463542
SPEG	4.663802
SPEM1	3.994266
SPEN	7.360248
SPERT	2.755962
SPESP1	3.281652
SPG11	6.857984
SPG20	6.90663
SPG21	8.990234
SPG7	6.141954
SPHK1	5.254064
SPHK2	4.888232
SPHKAP	2.448744
SPI1	4.930826
SPIB	4.84488
SPIC	2.411932
SPICE1	5.33648
SPIN1	8.973088
SPIN2A	2.664408
SPIN2B	4.905044
SPIN3	3.98914
SPIN4	5.991298
SPINK1	2.611216
SPINK14	2.235324
SPINK2	2.324794
SPINK4	3.408718
SPINK5	2.906768
SPINK6	5.347212
SPINK7	2.258212
SPINK9	2.227476
SPINLW1	2.980532
SPINT1	7.70345
SPINT2	9.478452
SPINT3	2.3714
SPINT4	2.276694
SPIRE1	7.483152
SPIRE2	6.57857
SPN	3.386714
SPNS1	7.87996
SPNS2	6.289464
SPNS3	3.879076
SPO11	2.32291
SPOCD1	3.844382

SPOCK1	5.978046
SPOCK2	6.467728
SPOCK3	2.841992
SPON1	3.474802
SPON2	4.693048
SPOP	8.186138
SPOPL	6.752858
SPP1	3.120676
SPP2	2.722946
SPPL2A	8.72131
SPPL2B	6.071492
SPPL3	8.06827
SPR	6.88423
SPRED1	5.536556
SPRED2	5.646254
SPRED3	4.780214
SPRN	4.368406
SPRNP1	5.9279
SPRR1A	8.15245
SPRR1B	7.530818
SPRR2A	3.88611
SPRR2B	3.742642
SPRR2B	3.067044
SPRR2D	2.885808
SPRR2E	2.477092
SPRR2G	4.539104
SPRR3	6.222124
SPRR4	4.894648
SPRY1	6.252978
SPRY2	6.358952
SPRY3	2.905734
SPRY3	2.905734
SPRY4	4.87147
SPRYD3	7.258
SPRYD4	5.510116
SPRYD5	2.598358
SPRYD5	2.829474
SPRYD5	2.619956
SPRYD5	2.619956
SPSB1	6.34083
SPSB2	5.64668
SPSB3	6.344086
SPSB4	4.370062

SPTA1	2.6929
SPTAN1	9.063396
SPTB	3.956762
SPTBN1	9.471916
SPTBN2	6.005342
SPTBN4	3.555284
SPTBN5	3.219524
SPTBN5	4.406402
SPTLC1	8.549208
SPTLC2	7.06717
SPTLC3	6.065716
SPTY2D1	5.512808
SPZ1	2.397836
SQLE	8.991784
SQRDL	8.827222
SQSTM1	11.00158
SR140	8.303284
SRA1	6.680228
SRBD1	5.81164
SRC	5.387028
SRCAP	7.43921
SRCIN1	5.061748
SRCRB4D	4.437042
SRD5A1	7.770008
SRD5A2	3.818578
SRD5A3	7.575502
SREBF1	7.277386
SREBF2	7.587474
SREK1	6.28155
SREK1IP1	4.569962
SRF	7.30611
SRFBP1	5.325422
SRGAP1	7.275038
SRGAP2	7.02104
SRGAP2	5.992582
SRGAP2P2	7.543558
SRGAP3	4.918384
SRGN	3.451992
SRI	8.630658
SRL	3.243334
SRM	8.059804
SRMS	4.9931
SRP14	9.440102

SRP19	7.092698
SRP19	5.666452
SRP54	8.140792
SRP68	9.342802
SRP72	8.667852
SRP9	8.229176
SRP9	8.904982
SRPK1	8.53383
SRPK2	8.078222
SRPK3	4.914526
SRPR	9.541686
SRPRB	8.964168
SRPX	3.396246
SRPX2	6.579136
SRR	6.41132
SRRD	6.832648
SRRM1	8.114916
SRRM2	8.355558
SRRM3	5.294066
SRRM4	3.154152
SRRT	7.996246
SRSF1	10.54828
SRSF10	7.333022
SRSF10	7.57889
SRSF11	8.497142
SRSF12	2.86274
SRSF2	9.046378
SRSF2IP	6.39274
SRSF3	9.010904
SRSF4	9.128738
SRSF5	10.068076
SRSF6	8.15054
SRSF7	8.610748
SRSF8	6.821098
SRSF9	8.61017
SRXN1	7.798204
SRXN1	4.14784
SRY	2.30322
SS18	8.18795
SS18L1	5.693984
SS18L2	8.098342
SSB	8.82715
SSBP1	8.751798

SSBP2	5.890646
SSBP3	7.539602
SSBP4	6.7412
SSC5D	5.120214
SSFA2	7.702438
SSH1	3.731828
SSH2	6.021634
SSH3	7.134578
SSNA1	7.71627
SSPN	7.080824
SSPO	5.225568
SSPO	4.965998
SSR1	9.687984
SSR2	10.99732
SSR3	8.647114
SSR4	6.70992
SSRP1	9.391892
SSRP1	4.79337
SSSCA1	7.519482
SST	3.578408
SSTR1	3.270164
SSTR2	4.355642
SSTR3	4.384936
SSTR4	3.66395
SSTR5	3.88391
SSU72	8.162232
SSX1	2.320038
SSX2	2.702034
SSX2	2.671376
SSX2IP	4.946408
SSX3	3.129612
SSX4	2.680488
SSX4	2.735852
SSX5	2.654656
SSX6	2.707582
SSX7	3.125772
SSX8	3.557178
SSX9	2.5488
ST13	9.19938
ST13P4	6.758606
ST13P5	7.093418
ST14	9.03847
ST18	2.483852

ST20	7.739588
ST3GAL1	6.979974
ST3GAL2	4.529844
ST3GAL3	4.718742
ST3GAL4	6.613476
ST3GAL5	6.493422
ST3GAL6	3.202794
ST5	6.534588
ST6GAL1	6.643094
ST6GAL2	3.825382
ST6GALNAC1	3.615236
ST6GALNAC2	5.338136
ST6GALNAC3	3.083712
ST6GALNAC4	5.614172
ST6GALNAC5	5.481514
ST6GALNAC6	7.54713
ST7	5.989002
ST7L	4.986
ST8SIA1	2.1794
ST8SIA1	4.077748
ST8SIA2	3.539132
ST8SIA3	2.549242
ST8SIA4	3.227448
ST8SIA5	3.246626
ST8SIA6	3.319416
STAB1	4.106606
STAB2	3.161276
STAC	4.318164
STAC2	4.329948
STAC3	4.051784
STAG1	6.506478
STAG2	7.879884
STAG3	3.944666
STAG3L2	8.727464
STAG3L3	8.729816
STAG3L3	8.576964
STAG3L4	7.724106
STAM	8.05296
STAM2	6.113026
STAMBP	8.051922
STAMBPL1	4.694032
STAP1	2.181122
STAP2	6.607218

STAR	3.991242
STARD10	6.824462
STARD13	6.337902
STARD3	7.516856
STARD3NL	6.315892
STARD4	6.121476
STARD5	5.332938
STARD6	2.265762
STARD7	9.820124
STARD8	3.53075
STARD9	3.211862
STARD9	2.879982
STARD9	3.468624
STAT1	10.38834
STAT2	7.597884
STAT3	10.73696
STAT4	3.662312
STAT5A	5.764416
STAT5B	6.39774
STAT6	8.09507
STATH	2.255912
STAU1	8.808728
STAU2	6.26587
STBD1	4.490928
STC1	3.273158
STC2	6.726246
STEAP1	3.275418
STEAP2	4.131904
STEAP3	6.029174
STEAP4	8.31403
STELLAR	3.751216
STGC3	2.80344
STH	3.126306
STIL	7.375514
STIM1	6.52894
STIM2	6.535184
STIP1	9.607902
STK10	6.921054
STK11	6.951562
STK11IP	4.84426
STK16	6.16057
STK17A	6.670302
STK17B	7.903184

STK19	6.516296
STK19	6.516296
STK19	6.698324
STK24	9.18218
STK25	5.747058
STK3	7.625144
STK31	2.564516
STK32A	3.273964
STK32B	3.490114
STK32C	5.105052
STK33	2.542108
STK35	8.018354
STK36	4.992078
STK38	8.23987
STK38L	6.806028
STK39	8.446034
STK4	7.840812
STK40	5.786378
STMN1	4.432332
STMN1	9.574374
STMN2	3.123318
STMN3	5.55161
STMN4	3.006252
STOM	6.769066
STOML1	5.09954
STOML2	8.787856
STOML3	2.80698
STON1	4.368008
STON1-GTF2	3.559276
STON2	3.421978
STOX1	3.024518
STOX2	4.913892
STRA13	6.85495
STRA6	6.921404
STRA8	2.807074
STRADA	5.59094
STRADB	6.064494
STRADB	6.372354
STRAP	8.906496
STRBP	6.893282
STRC	3.011282
STRC	3.175852
STRN	6.935798

STRN3	7.72872
STRN4	6.808974
STS	8.28587
STT3A	10.86574
STT3B	9.15589
STUB1	7.806328
STX10	8.553486
STX11	4.96948
STX12	7.585602
STX16	8.014694
STX17	7.101336
STX18	6.481692
STX19	2.62562
STX1A	4.678514
STX1B	4.058802
STX2	4.79121
STX3	6.596554
STX4	8.099656
STX5	8.13319
STX6	6.946702
STX7	6.954862
STX8	6.64227
STXBP1	7.259402
STXBP2	8.315168
STXBP3	6.449552
STXBP4	3.946612
STXBP5	4.521538
STXBP5L	2.219048
STXBP6	3.613506
STYK1	5.67414
STYX	7.518756
STYXL1	6.886068
SUB1	9.034668
SUCLA2	8.364494
SUCLG1	8.12553
SUCLG2	8.167068
SUCNR1	2.190658
SUDS3	7.952038
SUDS3	7.24004
SUFU	4.026824
SUGP1	7.277968
SUGP2	7.699082
SUGT1	6.604658

SUGT1P1	3.248496
SUGT1P3	4.268214
SULF1	2.817794
SULF2	8.479572
SULT1A1	5.363242
SULT1A2	6.577012
SULT1A3	8.668278
SULT1A3	8.582904
SULT1B1	2.263362
SULT1C2	2.56512
SULT1C3	2.581352
SULT1C4	2.374384
SULT1E1	2.49251
SULT2A1	2.485446
SULT2B1	6.35482
SULT4A1	4.441364
SULT6B1	2.384732
SUMF1	6.165424
SUMF2	9.244048
SUMO1	8.235688
SUMO1P1	3.673764
SUMO1P3	3.110302
SUMO2	9.287598
SUMO2	10.027396
SUMO2	9.393628
SUMO3	9.213394
SUMO4	4.334906
SUN1	8.956222
SUN2	7.288696
SUN3	2.66086
SUN5	3.213204
SUOX	5.47321
SUPT16H	9.259724
SUPT3H	4.231854
SUPT4H1	8.491928
SUPT5H	8.030762
SUPT6H	8.672446
SUPT7L	7.289802
SUPV3L1	7.781208
SURF1	6.84126
SURF2	7.734112
SURF4	9.715184
SURF6	6.586658

SUSD1	5.803682
SUSD2	5.074654
SUSD3	5.345756
SUSD4	5.907184
SUSD5	4.40231
SUV39H1	6.031166
SUV39H2	6.841662
SUV420H1	6.712778
SUV420H2	6.438492
SUZ12	8.573946
SUZ12P	7.157398
SV2A	3.513034
SV2B	2.349598
SV2C	2.990008
SVEP1	3.337078
SVIL	7.122162
SVIP	6.483998
SVOP	2.683666
SVOPL	5.389864
SWAP70	6.875
SYAP1	7.947394
SYBU	7.241044
SYCE1	2.946718
SYCE2	4.840322
SYCN	4.48696
SYCP1	2.230768
SYCP2	4.087426
SYCP2L	3.524968
SYCP3	2.141078
SYDE1	5.25498
SYDE2	3.420018
SYF2	6.83517
SYK	7.26257
SYMPK	7.412298
SYN1	3.98641
SYN2	3.967032
SYN3	4.489204
SYNC	4.811542
SYNCRIP	8.631442
SYNE1	3.01039
SYNE2	6.64921
SYNGAP1	5.130338
SYNGR1	6.172284

SYNGR2	7.191022
SYNGR3	6.54264
SYNGR4	5.348136
SYNJ1	5.496332
SYNJ2	6.300196
SYNJ2BP	6.603702
SYNM	6.19023
SYNPO	5.280282
SYNPO2	3.460178
SYNPO2L	3.68076
SYNPR	2.287334
SYNRG	7.178714
SYP	3.786774
SYPL1	9.248654
SYPL2	3.968088
SYS1	6.489484
SYT1	3.526706
SYT10	3.17862
SYT11	3.079076
SYT12	5.854682
SYT13	2.670868
SYT14	3.686502
SYT14L	2.457778
SYT15	5.103898
SYT16	3.6711
SYT17	7.098104
SYT2	3.711338
SYT2	3.299938
SYT3	4.166098
SYT4	3.13159
SYT5	4.175692
SYT6	3.559292
SYT7	5.272522
SYT8	5.272548
SYT9	3.117476
SYTL1	5.106934
SYTL2	4.845408
SYTL3	4.908682
SYTL4	5.631808
SYTL5	2.86158
SYVN1	6.316566
T	4.075278
T1560	2.704152

TAAR1	2.157782
TAAR2	2.701136
TAAR3	2.268276
TAAR5	3.084678
TAAR6	2.39021
TAAR8	2.407072
TAAR9	3.110846
TAB1	5.284652
TAB2	7.936958
TAB3	5.462016
TAC1	2.459498
TAC3	3.063562
TAC4	4.408742
TACC1	6.294466
TACC2	7.15674
TACC3	7.344526
TACO1	7.647032
TACR1	3.472204
TACR2	3.912792
TACR3	2.688404
TACSTD2	9.989146
TADA1	8.260754
TADA2A	7.488134
TADA2B	7.378438
TADA3	7.054152
TAF1	5.534622
TAF10	8.525058
TAF11	6.851172
TAF12	6.64457
TAF13	6.446214
TAF15	10.44072
TAF1A	5.004106
TAF1B	5.680306
TAF1C	5.369396
TAF1D	6.820878
TAF1L	3.679004
TAF2	8.182908
TAF3	6.388532
TAF4	6.373994
TAF4B	4.292534
TAF5	5.90575
TAF5L	6.903464
TAF6	6.776618

TAF6L	6.58611
TAF6L	4.850324
TAF7	7.473186
TAF7L	3.04066
TAF8	7.78858
TAF9	6.01989
TAF9	5.949924
TAF9B	6.011218
TAF9B	6.011218
TAGAP	3.290718
TAGLN	4.509722
TAGLN2	8.910378
TAGLN3	3.15089
tAKR	3.16073
TAL1	3.683208
TAL2	2.901226
TALDO1	9.39345
TANC1	7.484952
TANC2	4.88947
TANK	4.916472
TAOK1	7.800774
TAOK2	7.326124
TAOK3	8.309272
TAP1	8.54987
TAP1	8.54987
TAP1	8.54987
TAP2	7.849716
TAP2	7.852918
TAP2	7.854566
TAPBP	8.644412
TAPBP	9.618184
TAPBP	9.727382
TAPBPL	7.010056
TAPT1	6.795226
TARBP1	6.982292
TARBP2	5.599484
TARDBP	9.657856
TARP	2.339874
TARP	3.154798
TARP	2.850248
TARS	9.98962
TARS2	6.414802
TARSL2	3.70962

TAS1R1	4.200574
TAS1R2	3.662516
TAS1R3	5.25964
TAS2R1	3.06087
TAS2R10	2.536294
TAS2R13	2.956424
TAS2R14	3.5753
TAS2R16	2.371304
TAS2R19	3.43429
TAS2R20	2.723676
TAS2R3	3.252952
TAS2R31	3.968584
TAS2R38	3.008232
TAS2R39	2.382382
TAS2R4	2.383378
TAS2R40	2.977618
TAS2R41	3.28387
TAS2R42	2.736276
TAS2R43	2.176564
TAS2R46	2.427782
TAS2R5	3.1394
TAS2R50	2.812282
TAS2R60	2.635066
TAS2R7	2.518996
TAS2R8	2.18143
TAS2R9	2.384592
TASP1	5.779472
TAT	2.66936
TATDN1	6.177144
TATDN2	8.15099
TAX1BP1	8.307368
TAX1BP3	8.437906
TAZ	6.427108
TBC1D1	7.936998
TBC1D10A	7.13366
TBC1D10B	6.883906
TBC1D10C	4.72018
TBC1D12	5.22313
TBC1D13	7.103104
TBC1D14	8.056576
TBC1D15	7.012148
TBC1D16	5.683126
TBC1D17	6.469986

TBC1D19	4.013374
TBC1D2	8.214968
TBC1D20	7.457972
TBC1D21	3.112044
TBC1D22A	7.724192
TBC1D22B	6.340132
TBC1D23	6.208484
TBC1D24	5.53346
TBC1D25	5.543506
TBC1D26	4.460672
TBC1D27	4.750836
TBC1D28	3.961736
TBC1D29	4.007368
TBC1D2B	5.609126
TBC1D2B	6.164276
TBC1D3	7.256402
TBC1D3	7.216822
TBC1D30	4.3203
TBC1D3B	7.219674
TBC1D3B	7.233846
TBC1D3C	6.982792
TBC1D3F	7.241822
TBC1D3G	7.233806
TBC1D3G	7.24675
TBC1D3H	7.206248
TBC1D3P2	6.544076
TBC1D3P2	6.074652
TBC1D4	5.605322
TBC1D5	5.71122
TBC1D7	7.027972
TBC1D8	6.302216
TBC1D8B	2.67892
TBC1D9	7.242912
TBC1D9B	6.834492
TBCA	8.461922
TBCB	8.5019
TBCC	6.889128
TBCCD1	7.310026
TBCD	7.306716
TBCE	7.636884
TBCEL	6.715112
TBCK	6.844838
TBK1	6.802148

TBKBP1	4.240932
TBL1X	6.0084
TBL1XR1	8.777426
TBL1Y	3.428448
TBL2	7.210102
TBL3	6.166242
TBP	7.259246
TBPL1	6.772168
TBPL2	2.32052
TBR1	3.386646
TBRG1	7.279958
TBRG4	6.912988
TBX1	5.187064
TBX10	4.431528
TBX15	3.11726
TBX18	2.758588
TBX19	4.006552
TBX2	4.652134
TBX20	3.724046
TBX21	4.685694
TBX22	2.40444
TBX3	5.275782
TBX4	3.740504
TBX5	3.692292
TBX6	4.203466
TBXA2R	4.430026
TBXAS1	3.316174
TC2N	7.629606
TCAP	4.238538
TCEA1	9.394862
TCEA1	9.200606
TCEA2	6.954226
TCEA3	6.418566
TCEAL1	5.760148
TCEAL2	4.11333
TCEAL3	5.552594
TCEAL4	5.613534
TCEAL5	3.2578
TCEAL6	5.854108
TCEAL7	3.22741
TCEAL8	4.362448
TCEANC	4.655492
TCEANC	3.360832

TCEB1	5.947484
TCEB2	8.445986
TCEB3	7.403542
TCEB3B	2.951802
TCEB3C	4.190648
TCEB3CL	4.036944
TCEB3CL	4.092978
TCERG1	7.94761
TCERG1L	5.674546
TCF12	6.518568
TCF15	6.002964
TCF19	7.561624
TCF19	7.561624
TCF19	7.570068
TCF20	6.965958
TCF21	3.938868
TCF23	5.0261
TCF24	6.411938
TCF25	8.485098
TCF3	6.80845
TCF4	2.68444
TCF7	4.440184
TCF7L1	5.975622
TCF7L2	6.478928
TCFL5	6.980602
TCHH	3.774524
TCHHL1	2.733504
TCHP	6.556178
TCIRG1	5.891626
TCL1A	3.412374
TCL1B	3.06309
TCL6	2.781478
TCN1	3.125654
TCN2	4.684696
TCOF1	6.641094
TCP1	11.04674
TCP10	3.410064
TCP10	3.5332
TCP10L	3.747376
TCP10L	3.147172
TCP11	3.327328
TCP11L1	5.148682
TCP11L2	5.91405

TCTA	7.828454
TCTE1	3.985502
TCTE3	3.019452
TCTEX1D1	3.274134
TCTEX1D2	7.22465
TCTEX1D4	3.909186
TCTN1	5.572594
TCTN2	6.157236
TCTN3	6.105838
TDG	5.498664
TDGF1	2.220656
TDGF3	2.485368
TDH	3.21776
TDO2	2.300556
TDP1	6.996088
TDP2	8.102672
TDRD1	2.49614
TDRD10	3.330514
TDRD12	6.666318
TDRD3	5.836792
TDRD5	3.45251
TDRD6	2.496574
TDRD7	7.950772
TDRD9	2.474058
TDRG1	3.130168
TDRKH	6.00834
TEAD1	6.425738
TEAD2	7.243204
TEAD3	6.906774
TEAD4	6.727362
TEC	5.217006
TECPR1	4.62856
TECPR2	5.039264
TECR	10.23062
TECR	11.2555
TECRL	2.177302
TECTA	3.041132
TECTB	2.716618
TEDDM1	3.018606
TEF	5.930302
TEK	3.095164
TEKT1	2.933668
TEKT2	4.337066

TEKT3	3.143372
TEKT4	4.986626
TEKT4	3.73583
TEKT5	3.27675
TELO2	5.412798
TENC1	5.367806
TEP1	6.559086
TEPP	4.604252
TERC	7.13222
TERF1	5.883082
TERF1	6.844438
TERF1P2	4.788132
TERF2	7.93659
TERF2IP	6.817506
TERT	4.83599
TES	9.006846
TESC	4.512502
TESK1	7.215972
TESK2	6.60783
TET1	2.66605
TET2	5.249694
TET2	5.904138
TET3	5.332896
TEX10	7.896174
TEX101	3.017896
TEX11	2.638436
TEX12	2.400642
TEX13A	3.742234
TEX13B	3.500916
TEX14	3.100252
TEX15	2.624714
TEX19	3.438692
TEX2	7.64043
TEX261	3.35796
TEX261	8.008242
TEX264	6.205968
TEX28	3.015324
TEX28	3.015324
TEX28	3.30454
TEX9	2.755486
TF	9.168578
TFAM	6.419826
TFAP2A	3.882268

TFAP2A	6.820856
TFAP2B	3.197032
TFAP2C	7.427762
TFAP2D	2.42598
TFAP2E	5.870444
TFAP4	5.4857
TFB1M	5.774324
TFB2M	6.125936
TFCP2	7.63333
TFCP2L1	4.67628
TFDP1	10.233406
TFDP2	8.61452
TFDP3	2.727782
TFE3	6.291086
TFEB	5.329992
TFEC	2.311232
TFF1	5.329322
TFF2	4.442822
TFF3	3.926182
TFG	8.808018
TFIP11	7.915706
TFPI	4.847452
TFPI2	5.152724
TFPT	7.193592
TFR2	4.703852
TFRC	10.74136
TG	3.333648
TGDS	5.850966
TGFA	6.760802
TGFB1	6.031692
TGFB1I1	5.090354
TGFB2	5.823262
TGFB3	3.99733
TGFBI	5.839782
TGFBR1	7.407424
TGFBR2	6.527802
TGFBR3	5.747336
TGFBRAP1	6.60932
TGIF1	6.61459
TGIF2	5.968788
TGIF2LX	2.494278
TGIF2LY	2.496974
TGM1	6.029896

TGM2	6.763304
TGM3	3.644464
TGM4	3.242904
TGM5	5.048982
TGM6	3.923056
TGM7	3.10543
TGOLN2	7.728516
TGS1	7.01533
TH	4.997186
TH1L	8.897084
THADA	6.706412
THAP1	6.315554
THAP10	5.599178
THAP11	6.552008
THAP2	2.963596
THAP3	4.78915
THAP4	5.851318
THAP4	5.851318
THAP5	5.10501
THAP6	4.742996
THAP7	6.208664
THAP8	4.728916
THAP9	4.584526
THBD	4.69317
THBS1	7.637294
THBS2	3.733788
THBS3	5.437434
THBS4	3.924286
THEG	4.12127
THEM4	5.58748
THEM5	3.643676
THEMIS	2.361868
THG1L	7.3526
THNSL1	4.273418
THNSL2	5.770274
THOC1	6.549748
THOC2	7.658164
THOC3	7.950092
THOC3	7.94103
THOC4	9.63762
THOC4	9.671986
THOC5	7.75397
THOC6	7.331656

THOC7	7.692884
THOP1	6.551888
THPO	4.287572
THRA	5.318672
THRAP3	8.794314
THRB	5.017936
THRSP	4.19049
THSD1	4.055792
THSD1P1	3.597952
THSD4	5.358682
THSD7A	5.444726
THSD7B	3.07666
THTPA	5.107196
THUMPD1	6.409604
THUMPD2	5.350958
THUMPD3	7.850468
THY1	3.659212
THYN1	6.36024
TIA1	7.892516
TIAL1	6.176638
TIAM1	6.101648
TIAM2	4.204182
TICAM1	5.268798
TIE1	3.203184
TIFA	5.652708
TIGD1	5.856162
TIGD2	5.318372
TIGD3	5.490804
TIGD4	3.281156
TIGD5	5.917084
TIGD6	4.742628
TIGD7	3.764206
TIGIT	3.261362
TIMD4	2.795728
TIMELESS	7.977014
TIMM10	6.219672
TIMM13	7.93691
TIMM17A	7.8526
TIMM17B	6.8078
TIMM22	8.071468
TIMM23	10.015198
TIMM23	3.37109
TIMM23	9.89101

TIMM44	7.481714
TIMM50	9.068646
TIMM8A	6.063972
TIMM8B	6.887266
TIMM9	5.400542
TIMP1	9.469174
TIMP2	8.519866
TIMP2	4.818348
TIMP3	9.694882
TIMP3	6.798746
TIMP4	5.343236
TINAG	2.441082
TINAGL1	8.073206
TINF2	7.372836
TIPARP	8.855624
TIPIN	7.6396
TIPRL	8.118902
TIRAP	4.84788
TJAP1	6.06195
TJP1	8.226854
TJP2	8.64926
TJP3	7.808218
TK1	9.843774
TK2	6.199144
TKT	9.363284
TKTL1	3.462706
TKTL2	3.36379
TLCD1	6.706964
TLCD2	5.995946
TLE1	7.64874
TLE2	5.920226
TLE3	6.264202
TLE4	4.465826
TLE6	4.43053
TLK1	6.935362
TLK2	7.712468
TLL1	2.582902
TLL2	3.454312
TLN1	7.245914
TLN2	3.974234
TLR1	4.304798
TLR10	2.641908
TLR2	4.451574

TLR3	6.087842
TLR4	2.55888
TLR5	5.706748
TLR6	5.329668
TLR7	2.524638
TLR8	2.418284
TLR9	4.29772
TLX1	3.51854
TLX2	5.871882
TLX3	3.896612
TM2D1	4.735824
TM2D2	6.689166
TM2D3	5.909632
TM4SF1	10.2703
TM4SF18	9.204702
TM4SF19	8.540816
TM4SF20	2.29797
TM4SF4	3.397362
TM4SF5	4.335452
TM6SF1	2.776978
TM6SF2	4.086916
TM7SF2	7.23732
TM7SF3	8.325104
TM7SF4	2.40895
TM9SF1	8.04269
TM9SF2	10.86078
TM9SF3	9.669144
TM9SF4	8.136404
TMBIM1	9.574818
TMBIM4	7.678952
TMBIM6	11.3931
TMC1	2.620156
TMC2	2.509568
TMC3	2.910432
TMC4	8.552642
TMC5	8.724568
TMC6	6.253076
TMC7	6.743522
TMC8	5.210366
TMCC1	7.166132
TMCC2	3.775692
TMCC3	5.789988
TMCO1	8.890638

TMCO2	3.023104
TMCO3	7.741588
TMCO4	6.720772
TMCO5A	2.351708
TMCO6	6.104116
TMCO7	5.597124
TMED1	5.563266
TMED10	9.443422
TMED10P1	5.682432
TMED2	10.7244
TMED3	7.373624
TMED4	8.404438
TMED5	7.223124
TMED6	2.955006
TMED7	5.918416
TMED8	6.395778
TMED9	9.381572
TMEFF1	4.933534
TMEFF2	2.467698
TMEM100	2.480974
TMEM101	5.267952
TMEM102	6.654918
TMEM104	5.651326
TMEM105	5.245732
TMEM106A	5.786122
TMEM106A	5.816854
TMEM106B	7.693424
TMEM106C	8.73275
TMEM107	5.784704
TMEM108	2.799046
TMEM109	9.328206
TMEM11	6.951228
TMEM110	6.141918
TMEM111	6.330784
TMEM111	9.505278
TMEM115	6.391908
TMEM116	6.85306
TMEM117	4.84732
TMEM119	3.744058
TMEM120A	5.935422
TMEM120B	7.183604
TMEM120B	5.20651
TMEM121	6.07886

TMEM123	9.064078
TMEM125	6.59573
TMEM126A	6.270176
TMEM126B	4.868972
TMEM127	7.988918
TMEM128	5.81445
TMEM129	5.67846
TMEM130	4.508728
TMEM131	6.048654
TMEM132A	6.128918
TMEM132B	2.942398
TMEM132C	3.307156
TMEM132D	3.301824
TMEM132E	3.81321
TMEM133	3.50574
TMEM134	7.80745
TMEM135	5.985052
TMEM136	5.02731
TMEM138	7.450758
TMEM139	9.510104
TMEM140	5.569946
TMEM141	6.595612
TMEM143	5.36797
TMEM144	5.526932
TMEM145	4.269078
TMEM146	3.032592
TMEM147	8.006648
TMEM148	3.65307
TMEM149	4.525654
TMEM14A	7.58854
TMEM14B	10.54704
TMEM14C	9.387504
TMEM14E	3.123068
TMEM150A	5.293978
TMEM150B	4.556644
TMEM151A	5.861688
TMEM151B	3.423948
TMEM154	6.076038
TMEM155	2.901702
TMEM155	3.097396
TMEM156	2.894994
TMEM158	6.486686
TMEM159	7.472386

TMEM160	7.01989
TMEM161A	6.162932
TMEM161B	6.392582
TMEM163	2.785334
TMEM164	6.738788
TMEM165	9.8279
TMEM167A	7.106716
TMEM167B	6.95003
TMEM167B	6.95003
TMEM168	5.660368
TMEM169	3.899352
TMEM17	4.199024
TMEM170A	7.836742
TMEM171	4.975284
TMEM173	8.20463
TMEM174	2.4269
TMEM175	5.152484
TMEM176A	2.986398
TMEM176B	4.258778
TMEM177	5.481576
TMEM178	5.232812
TMEM179	4.412798
TMEM179B	8.11736
TMEM18	7.34363
TMEM180	5.43851
TMEM181	6.66073
TMEM182	5.76118
TMEM183A	8.940752
TMEM183B	8.914688
TMEM184A	6.05541
TMEM184B	7.979412
TMEM184C	7.719878
TMEM185A	7.055182
TMEM185B	7.432422
TMEM186	6.582842
TMEM187	5.675656
TMEM188	6.371874
TMEM189	7.421716
TMEM19	7.290804
TMEM190	4.999248
TMEM191A	7.256094
TMEM191A	4.801534
TMEM191A	7.343806

TMEM192	6.132948
TMEM194A	8.517302
TMEM194B	7.249648
TMEM194B	5.890354
TMEM195	2.344388
TMEM196	3.265358
TMEM198	4.59721
TMEM199	7.804312
TMEM2	6.045374
TMEM20	5.05322
TMEM200A	3.179064
TMEM200B	5.99409
TMEM201	5.519536
TMEM202	2.63847
TMEM203	8.132476
TMEM203	8.062948
TMEM204	4.59647
TMEM205	7.575644
TMEM206	4.539874
TMEM207	2.933854
TMEM208	7.146816
TMEM209	7.374638
TMEM211	3.16823
TMEM212	2.871998
TMEM214	8.654258
TMEM215	4.482216
TMEM216	6.54456
TMEM217	3.511712
TMEM218	7.510222
TMEM219	7.02606
TMEM22	4.834362
TMEM220	2.852646
TMEM221	6.060364
TMEM222	7.218594
TMEM223	7.135268
TMEM225	2.390988
TMEM229A	2.519828
TMEM229B	3.120982
TMEM231	3.942006
TMEM232	2.580012
TMEM25	5.41358
TMEM26	2.750324
TMEM27	3.216606

TMEM30A	7.792764
TMEM30B	7.243348
TMEM31	2.967038
TMEM33	7.570792
TMEM35	4.603336
TMEM37	4.98106
TMEM38A	6.423364
TMEM38B	6.597686
TMEM39A	9.349596
TMEM39B	6.139072
TMEM40	7.084718
TMEM41A	9.556604
TMEM41B	6.104122
TMEM42	5.849464
TMEM43	8.970598
TMEM44	6.331236
TMEM45A	9.00164
TMEM45B	8.3565
TMEM47	2.569558
TMEM48	8.417526
TMEM49	10.040666
TMEM5	5.876852
TMEM50A	9.624328
TMEM50B	5.262734
TMEM51	5.845076
TMEM52	4.86621
TMEM53	5.665338
TMEM54	8.466562
TMEM55A	6.134652
TMEM55B	7.247298
TMEM56	5.17338
TMEM57	6.954956
TMEM59	10.07636
TMEM59L	4.762918
TMEM60	6.04959
TMEM61	5.37518
TMEM62	7.894336
TMEM63A	8.151888
TMEM63B	6.792808
TMEM63C	3.210708
TMEM64	6.357448
TMEM65	4.978956
TMEM66	8.022736

TMEM67	5.057594
TMEM68	3.606208
TMEM68	5.443262
TMEM69	8.100236
TMEM70	6.160012
TMEM71	2.457852
TMEM72	4.521724
TMEM74	2.637238
TMEM75	3.313094
TMEM78	2.769408
TMEM79	6.84215
TMEM80	7.148466
TMEM81	4.19689
TMEM82	4.201524
TMEM85	9.98868
TMEM86A	3.78964
TMEM86B	5.555246
TMEM87A	9.057948
TMEM87B	7.321838
TMEM88	4.15616
TMEM89	5.584326
TMEM8A	6.282936
TMEM8B	4.61768
TMEM8C	4.402148
TMEM9	7.060414
TMEM90A	3.170344
TMEM90B	3.275058
TMEM91	4.81782
TMEM92	5.86987
TMEM93	7.676832
TMEM95	3.192936
TMEM97	7.960136
TMEM98	5.149194
TMEM99	6.124186
TMEM9B	7.056038
TMF1	6.718872
TMIE	3.365974
TMIGD1	2.58521
TMIGD2	5.366866
TMLHE	6.639176
TMOD1	7.12591
TMOD2	2.867556
TMOD3	8.731668

TMOD4	3.28133
TMPO	9.131492
TMPPE	5.29902
TMPRSS11A	2.197714
TMPRSS11B	2.308716
TMPRSS11D	2.317004
TMPRSS11E	2.754378
TMPRSS11E	2.643564
TMPRSS11F	2.450274
TMPRSS12	3.109944
TMPRSS13	5.45168
TMPRSS15	2.140852
TMPRSS2	5.722482
TMPRSS3	8.999844
TMPRSS4	7.461042
TMPRSS5	3.326932
TMPRSS6	4.085922
TMPRSS6	3.978602
TMPRSS7	3.017418
TMPRSS9	3.93707
TMSB10	10.67154
TMSB15A	2.451052
TMSB15B	2.40679
TMSB4X	9.899592
TMSB4X	9.635304
TMSB4X	10.78804
TMSB4XP2	6.9739
TMSB4Y	3.454618
TMSL3	11.0104
TMTC1	2.716036
TMTC2	5.364894
TMTC3	5.898216
TMTC4	5.802644
TMUB1	7.267444
TMUB2	6.866234
TMX1	9.230506
TMX2	8.478348
TMX3	4.102246
TMX4	6.05675
TNC	5.63399
TNF	4.014468
TNF	4.014468
TNF	4.014468

TNFAIP1	7.318442
TNFAIP2	7.839516
TNFAIP3	6.62765
TNFAIP6	3.430968
TNFAIP8	4.677876
TNFAIP8L1	4.115732
TNFAIP8L2	3.760744
TNFAIP8L3	3.941228
TNFRSF10A	7.036216
TNFRSF10B	7.66466
TNFRSF10C	3.078674
TNFRSF10D	3.950468
TNFRSF11A	5.366052
TNFRSF11B	3.532528
TNFRSF12A	8.08323
TNFRSF13B	6.021006
TNFRSF13C	4.9916
TNFRSF14	4.656246
TNFRSF17	2.544542
TNFRSF17	2.502398
TNFRSF18	5.763534
TNFRSF19	3.765304
TNFRSF1A	8.355178
TNFRSF1B	4.578924
TNFRSF21	8.457906
TNFRSF25	6.075062
TNFRSF4	5.016186
TNFRSF8	5.03818
TNFRSF9	3.620662
TNFSF10	8.444848
TNFSF11	2.950606
TNFSF12	5.997808
TNFSF13B	3.030368
TNFSF14	4.468466
TNFSF15	5.353218
TNFSF18	2.553438
TNFSF4	2.757504
TNFSF8	3.643742
TNFSF9	6.640908
TNIK	6.359946
TNIP1	7.282314
TNIP2	6.017768
TNIP3	2.396306

TNK1	5.608612
TNK2	6.210042
TNKS	6.335194
TNKS1BP1	7.29873
TNKS2	6.93094
TNMD	2.497944
TNN	3.40689
TNNC1	5.439572
TNNC2	4.241678
TNNI1	4.538104
TNNI2	4.983452
TNNI3	3.891632
TNNI3K	2.29787
TNNT1	7.406176
TNNT2	4.539258
TNNT3	5.011982
TNP1	3.057222
TNP2	3.106702
TNPO1	7.704364
TNPO2	8.50402
TNPO3	8.160602
TNR	2.894928
TNRC18	6.170638
TNRC18	7.280722
TNRC18	7.164002
TNRC18	6.09689
TNRC6A	6.663332
TNRC6B	6.710934
TNRC6C	5.159774
TNS1	4.752504
TNS3	7.18843
TNS4	5.58925
TNXA	5.669566
TNXB	4.984828
TNXB	5.20889
TNXB	5.20889
TOB1	9.589982
TOB2	7.4114
TOB2	9.815052
TOB2	9.815052
TOE1	7.38977
TOLLIP	6.392512
TOM1	7.664796

TOM1L1	7.742542
TOM1L1	4.662648
TOM1L2	5.300314
TOM1L2	7.487326
TOMM20	8.461506
TOMM20L	2.70955
TOMM22	9.688886
TOMM22	7.59082
TOMM34	7.825542
TOMM40	7.25877
TOMM40L	5.35322
TOMM7	6.247094
TOMM70A	7.69095
TOP1	8.321538
TOP1MT	7.319206
TOP2A	8.710636
TOP2B	6.660056
TOP3A	7.552208
TOP3B	6.773874
TOPBP1	7.922836
TOPORS	5.910254
TOR1A	8.23245
TOR1AIP1	8.281644
TOR1AIP1	5.71976
TOR1AIP2	7.86442
TOR1AIP2	9.057026
TOR1B	7.953354
TOR2A	4.94866
TOR3A	6.876654
TOX	5.857992
TOX2	4.461088
TOX3	3.942486
TOX4	8.41144
TP53	6.453936
TP53AIP1	2.504464
TP53BP1	6.657422
TP53BP2	7.034466
TP53I11	5.662944
TP53I13	5.611674
TP53I3	6.46787
TP53INP1	6.87399
TP53INP2	5.52725
TP53RK	6.138866

TP53TG1	3.605524
TP53TG3	2.710708
TP53TG3	2.823484
TP53TG3	2.703288
TP53TG3	2.703288
TP53TG5	3.47135
TP63	5.680818
TP73	5.069428
TPBG	8.454008
TPCN1	6.694162
TPCN2	6.978802
TPD52	5.613854
TPD52L1	9.421058
TPD52L2	8.60862
TPD52L3	2.81337
TPH1	2.537032
TPH2	2.715808
TPI1	8.858834
TPI1	8.591924
TPI1P2	5.364668
TPK1	3.07637
TPM1	6.115424
TPM1	4.798928
TPM2	4.321566
TPM3	5.076136
TPM3	11.03926
TPM4	8.43881
TPMT	6.66434
TPO	3.188282
TPP1	6.617868
TPP2	6.74821
TPPP	4.399906
TPPP2	2.892312
TPPP3	3.7853
TPR	7.035602
TPRA1	7.973858
TPRG1	4.714842
TPRG1L	8.167104
TPRKB	5.549006
TPRN	5.39561
TPRX1	3.109944
TPRXL	7.594038
TPSAB1	4.368124

TPSAB1	4.357556
TPSD1	5.57656
TPSG1	4.342496
TPST1	7.277456
TPST2	6.359582
TPT1	9.093262
TPTE	1.954892
TPTE2	2.217144
TPTE2P3	3.551904
TPX2	9.38057
TRA2A	7.976628
TRA2A	6.605228
TRA2B	9.988576
TRABD	7.260404
TRADD	7.13916
TRAF1	3.808896
TRAF2	7.415958
TRAF3	5.994472
TRAF3IP1	4.716452
TRAF3IP2	7.095508
TRAF3IP3	2.746178
TRAF4	8.218396
TRAF5	2.938378
TRAF6	6.583618
TRAF7	6.804892
TRAFD1	8.2022
TRAIP	5.487902
TRAJ17	2.820234
TRAK1	6.595772
TRAK2	6.279988
TRAM1	9.799588
TRAM1L1	2.592498
TRAM2	6.244888
TRANK1	6.027626
TRAP1	8.752402
TRAPPC1	8.043856
TRAPPC10	6.57571
TRAPPC2	5.773142
TRAPPC2L	6.91889
TRAPPC3	8.65642
TRAPPC4	8.72275
TRAPPC5	8.112992
TRAPPC6A	7.462522

TRAPPC6B	7.393818
TRAPPC8	5.084288
TRAPPC9	7.145426
TRAT1	2.532532
TRAV8-3	2.605974
TRBC1	3.524702
TRBV21OR9-	4.224194
TRBV23OR9-	3.83835
TRDMT1	2.693512
TRDN	2.380556
TREH	3.169192
TREM1	3.884156
TREM2	2.99797
TREML1	3.500484
TREML2	4.714618
TREML3	3.787496
TREML4	3.544592
TRERF1	5.039024
TRERF1	5.212966
TREX2	4.793064
TREX2	5.014288
TRGV3	2.571274
TRH	3.84708
TRHDE	3.422192
TRHR	2.766368
TRIAP1	7.809548
TRIB1	8.191316
TRIB2	5.767594
TRIB3	7.118316
TRIL	4.046884
TRIM10	3.195052
TRIM10	3.86621
TRIM10	3.195052
TRIM11	6.286366
TRIM13	4.765158
TRIM14	8.375888
TRIM15	5.184804
TRIM15	5.184804
TRIM15	5.184804
TRIM16	9.152806
TRIM16L	9.90698
TRIM17	5.37268
TRIM2	6.322596

TRIM21	6.709606
TRIM22	6.078534
TRIM22	3.32521
TRIM23	4.08762
TRIM24	6.956366
TRIM25	8.65311
TRIM26	7.764294
TRIM26	7.934706
TRIM27	7.3787
TRIM27	7.3787
TRIM28	9.784664
TRIM29	9.656722
TRIM3	4.565048
TRIM31	9.425168
TRIM31	9.412148
TRIM31	9.475082
TRIM32	7.437754
TRIM33	6.382862
TRIM35	5.161336
TRIM36	2.964342
TRIM37	7.057654
TRIM38	8.214368
TRIM39	4.973256
TRIM39	4.973256
TRIM39	4.973256
TRIM4	5.378664
TRIM40	3.950524
TRIM40	3.691652
TRIM40	3.691652
TRIM41	6.254554
TRIM42	2.870176
TRIM43	2.524174
TRIM43	2.53788
TRIM43	2.386358
TRIM44	7.299036
TRIM45	4.987782
TRIM46	4.599034
TRIM47	6.57013
TRIM48	2.941516
TRIM49	2.708288
TRIM49	2.959992
TRIM49	3.051658
TRIM49L1	2.66265

TRIM49L1	2.66265
TRIM5	7.551536
TRIM50	4.0266
TRIM52	6.929088
TRIM52	5.927904
TRIM54	4.246722
TRIM55	3.542536
TRIM56	6.849126
TRIM58	3.738682
TRIM59	5.860246
TRIM60	3.740198
TRIM61	4.12423
TRIM62	5.337612
TRIM63	3.054452
TRIM64	2.9898
TRIM64	2.9898
TRIM65	6.403702
TRIM66	5.148878
TRIM67	4.339034
TRIM68	2.64589
TRIM69	4.022632
TRIM6-TRIM	4.410394
TRIM7	4.05033
TRIM71	5.430656
TRIM72	4.283822
TRIM73	4.645878
TRIM73	4.58694
TRIM8	6.728766
TRIM9	3.359422
TRIML1	2.811414
TRIML2	5.267818
TRIO	7.58731
TRIOBP	5.849206
TRIP10	8.401728
TRIP11	4.076616
TRIP11	5.899188
TRIP12	9.057634
TRIP13	8.998702
TRIP4	7.349412
TRIP6	7.360704
TRIT1	7.220942
TRMT1	7.50168
TRMT11	5.812742

TRMT112	10.80418
TRMT12	7.513368
TRMT2A	6.48365
TRMT2B	6.65102
TRMT5	6.365186
TRMT6	7.0056
TRMT61A	5.488368
TRMT61B	6.508774
TRMU	6.382326
TRNAU1AP	7.2275
TRNP1	6.413142
TRNT1	5.768386
TRO	3.796942
TROAP	7.402508
TROVE2	6.921534
TRPA1	2.543388
TRPC1	3.137914
TRPC2	3.28596
TRPC3	2.81889
TRPC4	2.773806
TRPC4AP	7.72593
TRPC5	3.064698
TRPC6	2.528704
TRPC7	3.125278
TRPM1	3.379776
TRPM2	3.765284
TRPM3	3.135956
TRPM4	7.036254
TRPM5	4.512298
TRPM6	3.022656
TRPM7	6.711708
TRPM8	2.918646
TRPS1	8.41527
TRPT1	6.791902
TRPV1	5.624102
TRPV2	3.965492
TRPV3	4.365486
TRPV4	5.399636
TRPV5	3.345954
TRPV6	2.379264
TRRAP	7.376864
TRUB1	6.891772
TRUB2	8.936768

TRY6	3.92043
TRYX3	2.54122
TSC1	6.780934
TSC2	6.359352
TSC22D1	6.925546
TSC22D2	7.172118
TSC22D3	6.694132
TSC22D4	6.040926
TSEN15	7.368826
TSEN2	6.843828
TSEN34	7.72267
TSEN54	6.285652
TSFM	6.99931
TSG101	8.082974
TSGA10	2.344228
TSGA10IP	4.88023
TSGA14	5.133396
TSHB	2.137966
TSHR	2.852396
TSHZ1	3.954494
TSHZ2	3.145724
TSHZ2	3.295504
TSHZ3	3.271818
TSKS	3.860012
TSKU	7.873242
TSLP	2.28633
TSN	7.78904
TSNARE1	5.204716
TSNAX	6.687306
TSNAXIP1	3.033488
TSPAN1	8.409914
TSPAN10	5.05479
TSPAN11	5.084774
TSPAN11	3.651674
TSPAN12	2.778566
TSPAN13	9.554184
TSPAN14	7.613366
TSPAN15	8.814638
TSPAN16	2.577828
TSPAN16	3.481506
TSPAN17	7.324238
TSPAN18	3.692432
TSPAN2	4.41223

TSPAN3	9.60223
TSPAN31	8.46229
TSPAN32	4.40234
TSPAN33	5.507658
TSPAN4	6.291796
TSPAN5	6.50402
TSPAN6	6.960808
TSPAN7	3.087904
TSPAN8	2.14942
TSPAN9	5.236596
TSPO	7.010942
TSPO2	3.876152
TSPY1	3.73749
TSPY2	3.877694
TSPY26P	5.911466
TSPY3	3.411998
TSPY3	3.415662
TSPY3	3.413064
TSPY3	3.465506
TSPY3	3.251176
TSPY3	3.47021
TSPYL1	4.104854
TSPYL1	9.012428
TSPYL2	5.796106
TSPYL4	5.345238
TSPYL5	2.771792
TSPYL6	4.516908
TSR1	7.941742
TSR2	7.068492
TSSC1	7.050982
TSSC4	5.465508
TSSK1B	3.81588
TSSK2	3.811186
TSSK3	4.077622
TSSK4	3.34242
TSSK6	4.229294
TST	8.962358
TSTA3	9.488472
TSTD2	6.770576
TTBK1	4.092546
TTBK2	5.615798
TTC1	6.962992
TTC12	4.955978

TTC13	6.665184
TTC14	5.996584
TTC15	5.61986
TTC16	4.192022
TTC17	7.70755
TTC18	3.492422
TTC19	8.161398
TTC21A	3.449418
TTC21B	3.96357
TTC22	5.33972
TTC23	5.4534
TTC23L	2.704002
TTC24	4.921718
TTC25	2.711796
TTC26	5.007796
TTC27	6.58344
TTC28	3.731034
TTC28-AS	4.042086
TTC29	2.269942
TTC3	6.012608
TTC30A	4.028652
TTC30B	3.510878
TTC31	5.127558
TTC32	3.100798
TTC33	4.689476
TTC35	6.805518
TTC36	4.6408
TTC37	5.771272
TTC38	6.097426
TTC39A	5.828022
TTC39B	6.067976
TTC39C	5.87142
TTC39C	5.510742
TTC4	6.619916
TTC5	5.55166
TTC6	3.540618
TTC6	2.479858
TTC7A	6.423704
TTC7B	4.701488
TTC8	5.358676
TTC9	6.525668
TTC9B	4.726206
TTC9C	7.253082

TTF1	6.02407
TTF2	6.704478
TTI1	6.957572
TTK	6.84788
TTL	6.862458
TTLL1	5.459256
TTLL10	5.341964
TTLL11	4.586946
TTLL12	7.541234
TTLL13	3.09644
TTLL13	2.905412
TTLL2	2.650564
TTLL3	5.620996
TTLL4	7.290088
TTLL5	6.92273
TTLL6	3.042752
TTLL7	2.398462
TTLL8	3.39474
TTLL9	3.022818
TTN	2.739466
TTPA	3.546432
TTPAL	6.63457
TTR	3.284808
TTTY1	3.186128
TTTY1	3.186128
TTTY10	2.551384
TTTY11	2.405382
TTTY12	2.501612
TTTY13	3.158982
TTTY14	4.85449
TTTY2	2.56158
TTTY2	2.56158
TTTY5	3.789606
TTTY6	3.19547
TTTY6	3.19547
TTTY7	2.681424
TTTY7	2.681424
TTTY8	3.079128
TTTY8	3.079128
TTTY9A	2.502354
TTTY9A	2.502354
TTYH1	4.56623
TTYH2	4.526496

TTYH3	6.95187
TUB	4.011602
TUBA1A	4.61865
TUBA1B	9.28036
TUBA1C	10.134022
TUBA3C	4.942334
TUBA3D	4.868676
TUBA3E	3.05711
TUBA4A	8.043754
TUBA4B	4.756264
TUBA8	4.404256
TUBAL3	2.767084
TUBB	11.74212
TUBB	11.74212
TUBB	11.74212
TUBB1	2.882804
TUBB2A	5.619856
TUBB2A	5.619856
TUBB2A	6.032552
TUBB2B	5.292038
TUBB2C	10.81694
TUBB2C	3.937448
TUBB3	6.66601
TUBB4	5.936696
TUBB4	4.24033
TUBB4Q	2.479
TUBB4Q	7.400832
TUBB6	8.56334
TUBB8	6.06578
TUBB8	3.238456
TUBB8	6.04867
TUBBP5	6.998658
TUBD1	6.031454
TUBE1	5.011772
TUBG1	9.98981
TUBG2	6.353834
TUBGCP2	7.05937
TUBGCP3	7.486632
TUBGCP4	7.45895
TUBGCP5	5.747152
TUBGCP6	5.318332
TUFM	9.044294
TUFT1	7.753704

TUG1	9.068184
TULP1	4.229658
TULP2	3.312362
TULP3	7.229388
TULP4	5.595328
TUSC1	5.85253
TUSC2	5.968898
TUSC3	7.905138
TUSC5	3.58493
TUT1	6.177878
TWF1	7.770202
TWF1	7.889604
TWF2	7.367106
TWIST1	5.878374
TWISTNB	6.120392
TWSG1	7.8593
TXK	2.883754
TXLNA	8.255854
TXLNB	2.493922
TXLNG	5.78138
TXN	11.13702
TXN2	6.455328
TXNDC11	5.069418
TXNDC11	7.650364
TXNDC12	9.186182
TXNDC15	7.009764
TXNDC16	4.63655
TXNDC17	6.60643
TXNDC2	3.424056
TXNDC3	2.724528
TXNDC6	3.46484
TXNDC8	2.38799
TXNDC9	6.058014
TXNDC9	2.982738
TXNIP	10.7905
TXNL1	6.541818
TXNL4A	6.584706
TXNL4B	6.888188
TXNRD1	10.35268
TXNRD2	6.149148
TXNRD3	5.289114
TYK2	7.034492
TYMP	5.098174

TYMS	9.367384
TYR	2.658758
TYR	2.498852
TYRO3	5.63959
TYROBP	5.71099
TYRP1	2.644588
TYSND1	6.102572
TYW1	7.062322
TYW1B	5.487954
TYW3	6.272866
U2AF1	9.5094
U2AF1L4	6.211042
U2AF2	8.563708
UACA	5.071944
UAP1	8.044604
UAP1L1	5.431694
UBA1	8.420386
UBA2	8.322178
UBA3	8.80722
UBA5	7.21424
UBA52	10.59284
UBA6	7.111668
UBA7	6.236326
UBAC1	7.784588
UBAC2	7.77555
UBAP1	8.655456
UBAP2	8.276996
UBAP2L	9.282716
UBASH3A	3.081562
UBASH3B	7.723998
UBB	11.45842
UBC	12.41108
UBD	3.04074
UBD	3.060354
UBE2A	7.23835
UBE2B	6.811158
UBE2C	8.04536
UBE2CBP	3.885058
UBE2D1	5.04096
UBE2D2	9.068182
UBE2D3	7.554972
UBE2D4	6.795698
UBE2DNL	4.788958

UBE2E1	7.883752
UBE2E2	6.982228
UBE2E3	7.422492
UBE2E3	5.449328
UBE2F	6.354684
UBE2G1	9.067426
UBE2G2	7.321102
UBE2H	9.44798
UBE2I	6.15963
UBE2J1	7.305954
UBE2J2	6.455386
UBE2K	9.783014
UBE2K	4.223558
UBE2L3	10.27166
UBE2L3	7.586508
UBE2L6	7.7537
UBE2M	8.185466
UBE2MP1	9.078846
UBE2N	6.948962
UBE2NL	4.433924
UBE2O	5.757968
UBE2Q1	8.168628
UBE2Q2	6.210486
UBE2Q2P1	3.601944
UBE2Q2P3	5.063806
UBE2Q2P3	5.307616
UBE2Q2P3	5.928014
UBE2R2	8.017274
UBE2S	7.804346
UBE2S	7.448858
UBE2T	8.626206
UBE2U	2.111736
UBE2V2	7.403966
UBE2W	7.77515
UBE2Z	8.859038
UBE3A	6.879752
UBE3B	7.42152
UBE3C	8.45455
UBE4A	8.557592
UBE4B	7.651504
UBFD1	7.386882
UBIAD1	6.958088
UBL3	6.2525

UBL4A	6.542428
UBL4B	2.781602
UBL5	7.798096
UBL7	7.405722
UBLCP1	6.734872
UBN1	7.237254
UBN2	5.478576
UBP1	7.33272
UBQLN1	9.36906
UBQLN2	7.45888
UBQLN3	3.138558
UBQLN4	7.552562
UBQLN4	5.70412
UBQLNL	2.971372
UBR1	6.33856
UBR2	8.038496
UBR3	5.161494
UBR4	8.404012
UBR5	8.726586
UBR7	7.15066
UBTD1	6.41327
UBTD2	6.72936
UBTF	7.977456
UBTFL1	2.211726
UBTFL1	2.25722
UBTFL1	2.25722
UBTFL1	2.25722
UBTFL1	2.25722
UBTFL1	2.282548
UBXN1	7.139994
UBXN10	2.77308
UBXN11	6.283438
UBXN2A	6.647182
UBXN2B	6.76203
UBXN4	8.279072
UBXN4	5.437104
UBXN6	7.013358
UBXN7	8.6886
UBXN8	3.858858
UCA1	11.49134
UCHL1	4.884306
UCHL3	8.548962
UCHL5	6.325292

UCK1	8.346408
UCK2	9.573314
UCKL1	6.209234
UCMA	3.617698
UCN	5.973232
UCN2	4.363316
UCN3	3.484682
UCP1	2.992892
UCP2	4.39147
UCP3	4.85985
UEVLD	6.833932
UFC1	8.824
UFD1L	8.786412
UFM1	7.284676
UFSP1	5.851868
UFSP2	4.805968
UGCG	7.485286
UGDH	7.756498
UGGT1	8.5838
UGGT2	5.615816
UGP2	8.479298
UGP2	4.36147
UGT1A1	5.962966
UGT2A1	2.225068
UGT2A3	2.503154
UGT2A3	2.105498
UGT2B10	2.333202
UGT2B10	2.240852
UGT2B15	2.400648
UGT2B15	2.400648
UGT2B17	2.171264
UGT2B28	2.192978
UGT2B4	2.553566
UGT2B7	2.319468
UGT2B7	3.036702
UGT3A1	2.57503
UGT3A2	3.306158
UGT8	2.84015
UHMK1	8.992944
UHRF1	7.556914
UHRF1BP1	6.332124
UHRF1BP1L	5.19394
UHRF2	7.499604

UIMC1	6.890582
UIMC1	6.610278
UIMC1	6.890582
ULBP1	4.223114
ULBP2	4.55782
ULBP3	4.923252
ULK1	6.021562
ULK2	5.38502
ULK3	6.217566
ULK4	3.015216
ULK4	3.15497
UMOD	4.139238
UMODL1	3.69166
UMPS	8.76128
UNC119	6.314672
UNC119B	7.070712
UNC13A	3.123794
UNC13B	4.158422
UNC13C	2.602942
UNC13D	5.043922
UNC45A	6.537964
UNC45B	3.233776
UNC50	5.981732
UNC5A	5.322212
UNC5B	5.835046
UNC5C	3.00958
UNC5CL	3.457444
UNC5D	3.736836
UNC80	3.786542
UNC80	2.880888
UNC93A	3.158364
UNC93B1	7.760948
UNC93B1	7.604998
UNC93B1	7.67618
UNCX	6.699826
UNG	7.565044
UNK	6.547622
UNK	3.550722
UNKL	5.157278
UNKL	5.130566
UNQ5830	3.521274
UNQ6975	2.237972
UNQ9370	3.05154

UPB1	3.845432
UPF0639	3.655884
UPF1	7.854862
UPF2	6.096198
UPF3A	8.414822
UPF3B	4.33178
UPK1A	3.514352
UPK1A	4.000862
UPK1B	3.173712
UPK2	4.591628
UPK3A	4.072548
UPK3B	7.228322
UPP1	6.187402
UPP2	3.174168
UPRT	4.627882
UQCC	6.75421
UQCR10	8.328434
UQCR11	6.2487
UQCRB	5.39246
UQCRC1	8.277678
UQCRC2	10.54064
UQCRFS1	6.16251
UQCRFS1	9.41404
UQCRH	5.83116
UQCRH	9.350012
UQCRQ	8.132318
URB1	6.984636
URB2	6.474192
URGCP	6.677042
URM1	7.823838
UROC1	3.569794
UROD	7.768556
UROS	9.223222
USE1	7.624708
USF1	8.120332
USF2	7.823026
USH1C	3.552662
USH1G	4.380614
USH2A	2.705056
USHBP1	4.17846
USMG5	8.071314
USMG5	8.684008
USO1	6.952526

USP1	8.194382
USP10	9.848954
USP11	8.383326
USP12	6.927764
USP13	6.245352
USP14	9.084796
USP15	5.965134
USP16	5.674114
USP17	4.874346
USP17	4.874346
USP17	4.874346
USP17	4.874346
USP17	4.874346
USP17	4.874346
USP17	4.905508
USP17	4.874346
USP17L2	3.976904
USP17L2	3.662732
USP17L2	3.662732
USP17L2	4.02327
USP17L2	4.033764
USP17L6P	4.756186
USP17L6P	4.593844
USP18	8.85119
USP18	10.055048
USP19	6.80676
USP2	3.628532
USP20	5.115668
USP21	7.002258
USP22	8.483396
USP24	7.005422
USP25	6.655278
USP26	2.269624
USP27X	4.055134
USP28	7.222934
USP29	2.828312
USP3	7.100546
USP30	5.69989
USP31	7.30707
USP32	7.118964
USP33	7.475646
USP34	7.091254
USP35	4.723082

USP36	5.928266
USP37	6.11825
USP38	7.023154
USP39	8.224996
USP4	7.662718
USP40	6.902066
USP42	6.785394
USP43	5.620446
USP44	3.282302
USP45	4.913482
USP46	5.91765
USP47	5.79772
USP48	6.794856
USP49	4.438314
USP5	8.751256
USP50	2.346576
USP51	3.737736
USP53	6.585082
USP54	5.56878
USP6	3.093898
USP6NL	6.053018
USP6NL	5.973838
USP7	8.575018
USP8	5.967296
USP9X	8.165272
USP9Y	2.81512
USPL1	7.076346
UST	4.664494
UTF1	6.27084
UTP11L	7.457378
UTP14A	8.683024
UTP15	6.55309
UTP18	7.365924
UTP20	6.12657
UTP23	6.965882
UTP3	6.863642
UTP6	9.205548
UTRN	6.956706
UTS2	2.205902
UTS2D	2.35533
UTS2R	4.420196
UTY	2.64169
UVRAG	6.934388

UXS1	7.693022
UXT	6.9096
VAC14	6.596994
VAC14	4.394094
VAMP1	5.966202
VAMP2	6.59555
VAMP3	8.135672
VAMP4	5.097702
VAMP5	6.288638
VAMP7	7.224106
VAMP7	7.224106
VAMP8	9.80713
VANGL1	5.54721
VANGL2	5.76623
VAPA	7.643134
VAPB	7.858652
VARS	7.04514
VARS	7.04514
VARS2	5.572342
VARS2	5.745178
VASH1	4.562774
VASH2	3.621114
VASN	5.624564
VASP	7.536768
VAT1	9.127532
VAT1L	3.250584
VAV1	3.75908
VAV2	6.32286
VAV3	3.443572
VAX1	3.796664
VAX2	6.016538
VBP1	6.291798
VCAM1	2.93855
VCAN	2.681602
VCL	6.463944
VCP	9.990562
VCPIP1	7.858186
VCX	3.761888
VCX2	4.096286
VCX3A	3.800758
VCX3B	3.711096
VCY	4.158224
VCY	4.158224

VDAC1	7.92933
VDAC2	6.111068
VDAC2	10.23932
VDAC3	10.7755
VDR	5.759496
VEGFA	7.12732
VEGFB	7.1726
VEGFC	6.431842
VENTX	4.92509
VEPH1	4.319088
VEZF1	6.905708
VEZT	5.970548
VGF	4.990364
VGLL1	10.5414
VGLL2	2.9979
VGLL3	3.095992
VGLL4	6.91688
VHL	5.824912
VHL	7.84357
VHLL	2.923512
VIL1	3.191102
VILL	5.353232
VIM	3.643734
VIP	2.456396
VIPAR	7.23539
VIPR1	5.36173
VIPR2	3.124848
VIT	2.737772
VKORC1	8.691238
VKORC1L1	8.576984
VKORC1L1	4.5475
VLDLR	3.64816
VLDLR	2.603124
VMA21	6.66599
VMAC	4.90628
VMO1	3.684356
VN1R1	2.697356
VN1R2	3.031094
VN1R3	3.105588
VN1R4	2.12314
VN1R5	2.529534
VNN1	3.10109
VNN2	2.543458

VNN3	2.554034
VOPP1	8.901288
VOPP1	8.9017
VPRBP	7.306196
VPREB1	2.764246
VPREB3	4.682008
VPS11	7.136518
VPS13A	5.077378
VPS13B	5.967584
VPS13C	5.814014
VPS13D	6.393548
VPS18	6.764874
VPS24	8.764944
VPS25	7.691458
VPS26A	8.140792
VPS26B	6.307354
VPS28	7.856634
VPS29	7.267266
VPS33A	7.426064
VPS33B	6.382148
VPS35	8.89098
VPS36	6.244858
VPS37A	6.34365
VPS37B	7.301648
VPS37C	6.51101
VPS37D	5.81174
VPS39	7.786842
VPS41	7.914606
VPS45	7.7759
VPS4A	7.31867
VPS4B	6.82787
VPS52	8.798854
VPS52	8.798854
VPS52	8.795322
VPS53	6.791258
VPS53	6.893856
VPS54	5.0675
VPS72	7.864248
VPS8	6.60033
VRK1	7.319416
VRK2	6.71499
VRK3	7.200524
VRTN	2.915614

VSIG1	2.581592
VSIG10	6.152064
VSIG10L	6.005492
VSIG2	4.839846
VSIG4	2.86388
VSIG7	4.973736
VSIG8	4.429102
VSNL1	2.641782
VSTM1	2.77237
VSTM2A	3.10975
VSTM2B	6.23926
VSTM2L	5.207196
VSX1	3.42607
VSX2	4.678732
VTA1	7.539662
VTCN1	6.6416
VTI1A	5.60614
VTI1B	5.917264
VTN	4.19132
VTRNA1-1	2.714356
VTRNA1-2	3.244874
VTRNA1-3	3.405128
VWA1	5.913918
VWA2	4.084946
VWA3A	3.091946
VWA3B	2.777894
VWA5A	3.003274
VWA5B1	4.11669
VWA5B2	3.944922
VWC2	5.68295
VWC2L	2.57913
VWCE	4.309826
VWDE	6.058408
VWDE	6.454642
VWF	3.462414
WAC	8.810618
WAPAL	7.379832
WARS	10.661604
WARS2	5.11072
WAS	4.548024
WASF1	4.311668
WASF2	7.584228
WASF3	4.465824

WASH1	7.15532
WASH1	6.84722
WASH2P	6.815126
WASH3P	6.760282
WASH3P	6.794576
WASH7P	6.807856
WASL	6.9335
WBP1	8.39754
WBP1	8.195002
WBP1	8.221882
WBP11	8.674476
WBP11P1	4.012824
WBP2	7.485678
WBP2NL	3.468714
WBP4	5.491504
WBP5	4.07054
WBSCR16	8.07368
WBSCR17	3.45745
WBSCR17	3.568658
WBSCR22	9.259882
WBSCR27	6.014496
WBSCR28	3.963744
WDFY1	7.36288
WDFY2	5.396436
WDFY3	7.039964
WDFY4	3.406614
WDFY4	4.140828
WDHD1	7.19216
WDR1	10.21
WDR11	6.351208
WDR12	8.10141
WDR13	6.10526
WDR16	3.046756
WDR17	2.195296
WDR18	7.260284
WDR19	4.33195
WDR20	5.191936
WDR24	5.725326
WDR25	5.38805
WDR26	7.547892
WDR27	4.667928
WDR3	5.779844
WDR31	6.12832

WDR33	7.950626
WDR33	4.43893
WDR34	8.140286
WDR35	3.92315
WDR36	6.509472
WDR37	6.197924
WDR38	4.359792
WDR4	7.215216
WDR41	7.525058
WDR43	7.715066
WDR44	6.0843
WDR45	6.647338
WDR45L	8.622472
WDR46	7.464796
WDR46	7.464796
WDR47	4.494776
WDR48	7.060196
WDR49	2.67322
WDR5	7.95883
WDR52	3.620888
WDR52	3.750872
WDR53	7.751998
WDR54	6.060162
WDR55	7.108128
WDR59	7.26582
WDR5B	6.37219
WDR6	7.549674
WDR60	6.388188
WDR61	5.82242
WDR62	6.158008
WDR63	2.646068
WDR64	2.308542
WDR65	3.548534
WDR66	3.769134
WDR67	6.403618
WDR69	3.474154
WDR7	4.567836
WDR70	7.299466
WDR72	2.424424
WDR73	6.091832
WDR74	9.104256
WDR75	7.76335
WDR76	8.074656

WDR77	8.444984
WDR78	2.89033
WDR8	5.657712
WDR81	5.183984
WDR82	9.22092
WDR83	6.892182
WDR85	5.473618
WDR86	4.967582
WDR87	3.846354
WDR88	2.976832
WDR89	6.335506
WDR90	5.248966
WDR91	5.197538
WDR92	6.371402
WDR93	3.100388
WDSUB1	5.204112
WDTC1	6.391686
WDYHV1	7.251876
WEE1	6.544232
WEE1	6.39851
WEE1	6.818058
WFDC1	3.889934
WFDC10A	2.809846
WFDC10B	3.479984
WFDC11	2.4063
WFDC12	4.456592
WFDC13	3.750296
WFDC2	5.195752
WFDC3	3.731766
WFDC5	4.403126
WFDC6	3.35149
WFDC8	2.437098
WFDC9	2.328884
WFIKKN1	5.20542
WFIKKN2	4.57351
WFS1	6.525528
WHAMM	4.736422
WHAMML1	4.126504
WHSC1	6.146424
WHSC1L1	7.359786
WHSC2	6.87119
WIBG	6.662544
WIF1	2.488536

WIPF1	3.76551
WIPF2	6.277372
WIPF3	3.903278
WIPI1	7.61491
WIPI2	7.9314
WISP1	4.194556
WISP2	5.690846
WISP3	5.235514
WIZ	5.430622
WLS	7.84679
WNK1	8.799618
WNK1	5.446558
WNK2	5.68126
WNK3	2.512374
WNK4	4.57768
WNT1	5.48178
WNT10A	6.177294
WNT10B	4.962492
WNT11	4.35699
WNT16	3.642792
WNT2	2.69508
WNT2B	3.688672
WNT3	4.86566
WNT3A	4.324214
WNT4	4.688836
WNT5A	2.463266
WNT5B	4.95757
WNT6	5.157612
WNT7A	5.24773
WNT7B	6.231182
WNT8A	3.623536
WNT8B	3.313096
WNT9A	6.548484
WNT9B	5.531454
WRAP53	6.300138
WRB	7.492084
WRN	5.223924
WRNIP1	7.048048
WSB1	7.98225
WSB2	8.912678
WSCD1	4.14441
WSCD2	3.86056
WT1	5.498944

WT1-AS	3.86128
WTAP	6.633354
WTAP	9.369768
WTIP	5.680184
WWC1	7.035646
WWC2	6.302368
WWC3	4.613712
WWOX	5.95415
WWP1	7.87186
WWP2	6.27652
WWTR1	7.942826
XAB2	7.847656
XAF1	8.299478
XAGE1A	3.20796
XAGE1A	3.20796
XAGE1A	3.20796
XAGE1A	3.20796
XAGE1A	3.20796
XAGE2	3.051646
XAGE2	3.051646
XAGE3	4.022364
XAGE-4	3.606344
XAGE5	2.655284
XBP1	9.643272
XCL1	4.286578
XCL2	2.565238
XCR1	4.096444
XDH	6.434592
XG	2.801848
XIAP	7.585806
XIRP1	3.631304
XIRP2	2.390902
XK	5.681838
XKR3	2.212196
XKR4	4.285382
XKR5	3.2908
XKR6	3.501018
XKR7	4.155652
XKR8	5.59277
XKR9	2.392158
XKRX	3.711316
XKRY	2.299404
XKRY	2.299404

XPA	7.075134
XPC	6.242308
XPNPEP1	7.969742
XPNPEP2	3.018006
XPNPEP3	7.06796
XPO1	9.45975
XPO4	6.819852
XPO5	8.39578
XPO6	9.87704
XPO7	8.0457
XPOT	8.58007
XPR1	7.292642
XRCC1	7.290204
XRCC2	7.496864
XRCC3	5.852846
XRCC4	5.330986
XRCC5	9.522088
XRCC6	10.57064
XRCC6BP1	5.39623
XRN1	6.958808
XRN2	7.18115
XRRA1	4.962712
XYLB	5.635654
XYLT1	4.88294
XYLT2	5.888126
YAF2	6.104884
YAP1	8.191224
YARS	9.006858
YARS2	5.698556
YBX1	10.84344
YBX1P2	8.525868
YBX2	6.082196
YDJC	7.455634
YEATS2	7.669254
YEATS4	7.02013
YES1	7.853378
YIF1A	8.29393
YIF1B	6.188442
YIF1B	6.096628
YIPF1	6.177864
YIPF2	7.08817
YIPF3	8.430706
YIPF4	7.048996

YIPF4	7.841914
YIPF5	8.424856
YIPF6	7.136616
YIPF7	2.346476
YJEFN3	6.07813
YKT6	9.456698
YLPM1	6.833942
YME1L1	10.57868
YOD1	5.73747
YPEL1	4.66138
YPEL2	4.673538
YPEL3	6.778738
YPEL4	3.64761
YPEL5	6.563966
YPLR6490	2.90281
YRDC	8.010752
YSK4	2.420298
YTHDC1	7.727424
YTHDC2	5.223068
YTHDF1	8.493174
YTHDF2	7.30246
YTHDF3	8.154696
YWHAB	9.67545
YWHAE	11.10524
YWHAE	11.19092
YWHAG	7.355498
YWHAH	9.293774
YWHAQ	10.76118
YWHAQP8	6.671472
YWHAZ	11.05946
YY1	8.370846
YY1AP1	7.64244
YY2	4.656598
ZACN	3.125946
ZADH2	6.141526
ZAK	5.793296
ZAN	3.453124
ZAP70	4.029924
ZAR1	5.085062
ZAR1L	2.830174
ZBBX	2.339022
ZBED1	7.063452
ZBED1	7.063452

ZBED2	3.916914
ZBED3	5.739312
ZBED4	6.640246
ZBED5	6.388666
ZBP1	3.883678
ZBTB1	6.498776
ZBTB10	6.230916
ZBTB11	4.98732
ZBTB11	5.71417
ZBTB12	5.516276
ZBTB12	5.516276
ZBTB16	4.117556
ZBTB17	5.387248
ZBTB2	6.146406
ZBTB20	4.668806
ZBTB22	5.584002
ZBTB22	5.538082
ZBTB24	6.171428
ZBTB25	5.110666
ZBTB26	5.917088
ZBTB3	5.147848
ZBTB32	4.164856
ZBTB33	5.53942
ZBTB34	10.245016
ZBTB34	6.794926
ZBTB34	7.380904
ZBTB34	6.401658
ZBTB37	5.058732
ZBTB38	8.35679
ZBTB38	7.522794
ZBTB39	5.786698
ZBTB4	6.103628
ZBTB40	5.614084
ZBTB41	6.251122
ZBTB42	5.091914
ZBTB43	7.01263
ZBTB44	6.433776
ZBTB45	5.389868
ZBTB46	5.417106
ZBTB47	5.496582
ZBTB48	4.611152
ZBTB49	4.904708
ZBTB5	6.156712

ZBTB6	5.92614
ZBTB7A	6.768188
ZBTB7B	6.352192
ZBTB7C	4.72351
ZBTB8B	5.1977
ZBTB8OS	5.48594
ZBTB9	6.162738
ZC3H10	4.025428
ZC3H11A	7.955284
ZC3H12A	6.688944
ZC3H12B	2.5399
ZC3H12C	5.552972
ZC3H12D	5.499292
ZC3H13	6.694126
ZC3H14	6.347838
ZC3H15	7.447752
ZC3H18	7.687792
ZC3H3	7.285208
ZC3H4	6.365906
ZC3H6	4.463672
ZC3H7A	7.62838
ZC3H7B	7.13332
ZC3H8	5.931638
ZC3HAV1	9.723696
ZC3HAV1L	3.01206
ZC3HC1	6.020314
ZC4H2	3.041752
ZCCHC10	4.709812
ZCCHC11	5.860796
ZCCHC12	3.384974
ZCCHC13	3.081586
ZCCHC14	5.459358
ZCCHC16	2.804174
ZCCHC17	7.469668
ZCCHC18	2.3842
ZCCHC2	5.571844
ZCCHC2	3.880506
ZCCHC24	5.02753
ZCCHC3	6.105868
ZCCHC4	5.468928
ZCCHC5	2.7407
ZCCHC6	6.79395
ZCCHC7	5.397562

ZCCHC8	7.050104
ZCCHC9	5.941238
ZCRB1	8.269448
ZCWPW1	5.223628
ZCWPW2	2.401198
ZDBF2	5.622688
ZDHHC1	3.619698
ZDHHC11	4.693196
ZDHHC12	7.413502
ZDHHC13	8.061502
ZDHHC14	4.44161
ZDHHC15	2.46273
ZDHHC16	7.645274
ZDHHC17	4.850494
ZDHHC18	6.888632
ZDHHC19	3.89894
ZDHHC2	5.527606
ZDHHC20	7.159298
ZDHHC21	6.199134
ZDHHC22	3.139766
ZDHHC23	6.09691
ZDHHC24	6.358278
ZDHHC3	6.038314
ZDHHC3	7.907264
ZDHHC4	8.14729
ZDHHC5	9.008768
ZDHHC6	7.099948
ZDHHC7	8.40498
ZDHHC8	4.764532
ZDHHC8P1	4.841382
ZDHHC9	9.03637
ZEB1	2.807048
ZEB2	2.981462
ZER1	6.610644
ZFAND1	8.191292
ZFAND2A	8.766254
ZFAND2B	6.541852
ZFAND3	7.20184
ZFAND5	9.48709
ZFAND6	8.387006
ZFAT	5.137598
ZFC3H1	5.917868
ZFHX2	3.557708

ZFHX2	3.929722
ZFHX3	5.722936
ZFHX4	3.023076
ZFP1	5.518182
ZFP106	7.380824
ZFP112	4.081976
ZFP14	2.699434
ZFP161	5.656716
ZFP2	2.5931
ZFP28	2.699804
ZFP3	4.208382
ZFP30	4.162458
ZFP36	5.852544
ZFP36L1	8.839906
ZFP36L2	7.561082
ZFP37	2.76652
ZFP41	5.765268
ZFP42	2.927004
ZFP57	4.633516
ZFP62	6.182474
ZFP64	6.618632
ZFP82	3.382524
ZFP90	5.435936
ZFP91-CNTF	6.413176
ZFP92	3.382704
ZFPL1	7.645244
ZFPM1	5.336002
ZFPM2	2.460498
ZFR	8.335792
ZFR2	4.674888
ZFX	6.854318
ZFY	2.828372
ZFYVE1	6.268922
ZFYVE16	4.500522
ZFYVE19	6.601326
ZFYVE20	6.663714
ZFYVE21	6.238718
ZFYVE26	6.25063
ZFYVE27	5.459848
ZFYVE28	4.096398
ZFYVE9	3.409988
ZG16	3.185456
ZG16	3.20052

ZG16B	6.133522
ZGPAT	6.290034
ZHX1	6.243858
ZHX2	6.887648
ZHX3	4.17149
ZIC1	3.440534
ZIC2	5.880908
ZIC3	3.933984
ZIC4	2.869982
ZIC5	5.316646
ZIK1	3.02714
ZIM3	2.580202
ZKSCAN1	6.909152
ZKSCAN2	4.38317
ZKSCAN3	3.976828
ZKSCAN4	4.244574
ZKSCAN5	6.026712
ZMAT1	2.184246
ZMAT2	8.329242
ZMAT3	6.279138
ZMAT4	3.704502
ZMAT5	5.778734
ZMIZ1	5.807544
ZMIZ2	6.502992
ZMPSTE24	8.536882
ZMYM1	6.601682
ZMYM2	8.067502
ZMYM3	5.847498
ZMYM4	7.364334
ZMYM5	5.520174
ZMYM6	5.787152
ZMYND10	4.291506
ZMYND11	7.312062
ZMYND12	3.145794
ZMYND15	4.767676
ZMYND17	3.388138
ZMYND19	7.155096
ZMYND8	7.837324
ZNF10	2.63977
ZNF100	2.33219
ZNF101	6.824632
ZNF107	4.927434
ZNF114	3.322756

ZNF117	3.545708
ZNF12	6.213518
ZNF121	6.448152
ZNF124	5.246796
ZNF131	7.41524
ZNF132	3.376562
ZNF133	4.93046
ZNF134	6.273572
ZNF135	3.824872
ZNF136	3.841208
ZNF137P	2.866132
ZNF138	4.145368
ZNF14	3.338148
ZNF140	5.040772
ZNF141	5.042698
ZNF142	5.75945
ZNF143	5.736032
ZNF146	6.455022
ZNF148	7.447112
ZNF154	3.609842
ZNF155	4.650544
ZNF157	3.656712
ZNF16	5.158824
ZNF160	3.566668
ZNF165	6.321974
ZNF167	3.188496
ZNF169	3.76596
ZNF17	4.982248
ZNF174	5.449902
ZNF175	4.44509
ZNF177	3.17563
ZNF18	4.887892
ZNF180	5.348438
ZNF181	3.786184
ZNF182	4.038444
ZNF184	4.404258
ZNF185	5.731406
ZNF187	4.342912
ZNF189	5.125768
ZNF19	4.511632
ZNF192	5.88211
ZNF193	5.022086
ZNF195	5.037328

ZNF197	3.247916
ZNF2	4.008818
ZNF20	2.735532
ZNF200	5.550266
ZNF202	5.154656
ZNF204P	2.217552
ZNF205	5.90405
ZNF207	9.476182
ZNF208	2.38221
ZNF211	4.384534
ZNF212	7.293278
ZNF213	5.608386
ZNF214	2.189596
ZNF215	2.262106
ZNF217	8.57345
ZNF219	5.488408
ZNF22	5.286784
ZNF221	2.767898
ZNF222	3.808918
ZNF223	4.471214
ZNF224	4.447784
ZNF225	3.88026
ZNF226	4.06582
ZNF227	5.57374
ZNF229	4.989638
ZNF23	4.272874
ZNF230	2.820358
ZNF232	3.771022
ZNF233	3.1061
ZNF234	4.900166
ZNF235	3.47723
ZNF236	4.843302
ZNF238	5.4148
ZNF239	3.418994
ZNF24	6.327036
ZNF248	4.591282
ZNF25	3.8265
ZNF250	5.785898
ZNF251	7.196288
ZNF252	5.88113
ZNF253	2.580016
ZNF253	2.943736
ZNF254	4.391548

ZNF256	4.356466
ZNF257	2.0837
ZNF259	7.769984
ZNF26	4.924142
ZNF260	5.605226
ZNF263	6.08203
ZNF264	7.28431
ZNF266	6.079342
ZNF267	5.908582
ZNF268	4.504678
ZNF271	3.952362
ZNF273	4.695674
ZNF274	6.454094
ZNF275	5.724368
ZNF276	5.617108
ZNF277	5.17644
ZNF277	2.776956
ZNF28	3.799032
ZNF280A	2.763056
ZNF280B	3.169492
ZNF280C	5.716944
ZNF280D	5.205124
ZNF281	6.541826
ZNF282	6.75733
ZNF283	5.513494
ZNF284	4.201328
ZNF285	3.080532
ZNF286A	5.89039
ZNF286B	4.374902
ZNF287	2.634342
ZNF292	6.522116
ZNF295	6.290124
ZNF296	5.634924
ZNF29P	2.740118
ZNF3	6.620602
ZNF30	2.728152
ZNF300	5.102574
ZNF300P1	2.20441
ZNF302	5.935024
ZNF304	5.884754
ZNF311	3.591904
ZNF311	3.591904
ZNF317	6.59024

ZNF318	5.763002
ZNF319	4.774562
ZNF32	5.056904
ZNF320	2.639424
ZNF321	2.67529
ZNF322A	5.49894
ZNF322A	5.268662
ZNF323	3.930684
ZNF324	5.819106
ZNF324B	5.876568
ZNF326	5.55379
ZNF329	3.060642
ZNF330	6.804028
ZNF331	4.698858
ZNF333	4.439052
ZNF334	2.45028
ZNF335	6.123116
ZNF337	4.911878
ZNF33A	5.730292
ZNF33B	4.839926
ZNF34	5.041642
ZNF341	5.239896
ZNF343	5.354174
ZNF345	2.169792
ZNF346	6.93111
ZNF347	2.363532
ZNF35	5.541086
ZNF350	3.813444
ZNF354A	4.34971
ZNF354B	3.574932
ZNF354C	4.218106
ZNF358	7.21783
ZNF362	6.8745
ZNF365	3.959856
ZNF366	3.489778
ZNF367	6.858036
ZNF37A	4.277054
ZNF37BP	5.185064
ZNF382	2.855654
ZNF383	5.411736
ZNF384	4.643222
ZNF384	8.04925
ZNF385A	6.618264

ZNF385B	3.07642
ZNF385C	4.55762
ZNF385D	2.678622
ZNF391	3.081686
ZNF394	6.333918
ZNF395	5.713192
ZNF396	2.671204
ZNF397	4.574076
ZNF398	6.13707
ZNF404	2.52289
ZNF407	3.949468
ZNF408	5.095968
ZNF41	4.268556
ZNF410	7.487258
ZNF414	6.327202
ZNF415	2.79824
ZNF416	5.872562
ZNF417	6.486598
ZNF418	3.54799
ZNF419	5.36571
ZNF420	3.154168
ZNF423	3.587092
ZNF425	5.937592
ZNF426	6.0891
ZNF428	5.070228
ZNF429	2.149436
ZNF43	2.319762
ZNF430	3.316766
ZNF431	3.742158
ZNF432	2.997558
ZNF433	2.923012
ZNF434	3.10137
ZNF434	5.493042
ZNF436	6.856954
ZNF438	4.035672
ZNF439	3.195514
ZNF44	2.83235
ZNF440	3.226398
ZNF441	2.569606
ZNF442	3.507674
ZNF443	2.727918
ZNF444	5.897682
ZNF445	5.945552

ZNF446	5.531382
ZNF449	4.190378
ZNF45	5.080706
ZNF451	6.144434
ZNF454	3.050562
ZNF460	8.877302
ZNF461	4.25671
ZNF462	7.557512
ZNF467	5.250544
ZNF468	3.680382
ZNF469	5.15494
ZNF470	2.484958
ZNF471	2.165306
ZNF473	6.029286
ZNF474	2.73872
ZNF479	2.53123
ZNF479	2.751782
ZNF479	2.343198
ZNF48	5.698692
ZNF480	5.151966
ZNF483	2.805382
ZNF484	6.068302
ZNF485	5.324622
ZNF486	2.66564
ZNF487P	4.941988
ZNF488	5.649178
ZNF490	5.516452
ZNF491	2.690636
ZNF492	2.636764
ZNF493	2.485914
ZNF496	5.950588
ZNF497	5.265806
ZNF498	6.211586
ZNF500	5.055506
ZNF501	3.039206
ZNF502	2.4275
ZNF503	3.348322
ZNF503	5.957708
ZNF506	3.359076
ZNF507	6.234012
ZNF510	5.415238
ZNF511	6.678494
ZNF512	7.039186

ZNF512B	5.887482
ZNF513	6.060314
ZNF514	4.736156
ZNF516	4.772706
ZNF517	4.906614
ZNF518A	4.992738
ZNF518B	4.821812
ZNF519	3.073884
ZNF521	2.88924
ZNF524	5.565562
ZNF525	9.108562
ZNF526	6.230534
ZNF527	3.534402
ZNF528	2.907668
ZNF529	4.894806
ZNF530	4.361166
ZNF532	4.979544
ZNF534	2.77866
ZNF536	2.553696
ZNF540	2.183454
ZNF540	3.741372
ZNF541	3.796286
ZNF542	2.481402
ZNF543	6.074802
ZNF544	6.430488
ZNF546	2.474564
ZNF547	3.586354
ZNF548	5.361924
ZNF549	4.321814
ZNF550	5.777104
ZNF551	4.936114
ZNF552	9.104662
ZNF552	5.299396
ZNF554	4.525206
ZNF555	4.249154
ZNF556	2.965188
ZNF557	5.60038
ZNF558	5.514948
ZNF559	5.66918
ZNF56	2.289062
ZNF560	2.35055
ZNF561	4.552024
ZNF562	6.172262

ZNF563	2.513044
ZNF564	5.54641
ZNF565	3.346164
ZNF566	4.283002
ZNF567	3.033138
ZNF568	2.18494
ZNF569	2.57453
ZNF57	5.507968
ZNF570	2.745756
ZNF571	2.926386
ZNF572	4.328536
ZNF573	2.737216
ZNF574	5.9053
ZNF575	5.21287
ZNF576	5.47327
ZNF577	2.630964
ZNF578	2.16119
ZNF579	6.07144
ZNF580	6.197944
ZNF581	6.375556
ZNF582	2.457452
ZNF583	2.643778
ZNF584	5.877292
ZNF585A	2.977798
ZNF585B	5.30462
ZNF586	5.91801
ZNF587	7.624102
ZNF587	6.641116
ZNF589	5.129778
ZNF592	6.178324
ZNF593	6.641884
ZNF594	5.048312
ZNF595	3.882556
ZNF596	2.58369
ZNF597	2.465012
ZNF598	5.960976
ZNF599	3.082424
ZNF600	3.464194
ZNF605	2.92856
ZNF606	3.847478
ZNF606	5.143016
ZNF607	3.817756
ZNF608	5.022874

ZNF609	6.960484
ZNF610	2.740176
ZNF611	4.736712
ZNF613	3.054968
ZNF614	4.576486
ZNF615	2.830056
ZNF616	2.468824
ZNF616	2.290738
ZNF618	5.5628
ZNF618	4.127898
ZNF619	4.912842
ZNF620	5.009306
ZNF621	5.948034
ZNF622	8.107604
ZNF623	7.884256
ZNF624	4.021414
ZNF625	3.571308
ZNF625	2.787186
ZNF626	2.828014
ZNF627	6.271826
ZNF628	5.28242
ZNF629	5.17673
ZNF630	3.316184
ZNF638	6.267986
ZNF639	5.890768
ZNF641	4.863844
ZNF642	3.498872
ZNF643	3.829502
ZNF644	5.067996
ZNF645	2.74686
ZNF646	5.647064
ZNF648	3.093362
ZNF649	2.627704
ZNF652	6.094888
ZNF653	5.681292
ZNF654	4.390156
ZNF655	4.755288
ZNF658	3.562498
ZNF658	3.560108
ZNF658	3.482266
ZNF660	2.448346
ZNF662	3.583908
ZNF663	2.501738

ZNF664	7.357706
ZNF665	2.185988
ZNF667	3.129196
ZNF668	3.938724
ZNF668	5.387924
ZNF669	5.060632
ZNF66P	3.769922
ZNF670	4.652318
ZNF671	4.665586
ZNF672	6.301114
ZNF673	5.432224
ZNF674	2.868058
ZNF675	3.350282
ZNF676	2.180398
ZNF677	2.638378
ZNF678	2.944438
ZNF678	3.19923
ZNF679	2.674534
ZNF680	4.705176
ZNF681	2.575694
ZNF682	2.271134
ZNF683	4.288084
ZNF684	3.411442
ZNF687	5.923122
ZNF688	5.152576
ZNF689	6.264468
ZNF69	3.885002
ZNF691	5.165924
ZNF692	6.377804
ZNF695	5.051312
ZNF696	4.797178
ZNF697	4.82217
ZNF699	3.193662
ZNF7	6.835358
ZNF70	5.987518
ZNF700	5.882588
ZNF701	4.741856
ZNF702P	2.51525
ZNF703	5.416372
ZNF704	5.803484
ZNF705A	2.763792
ZNF705D	2.560128
ZNF706	6.443638

ZNF707	6.1698
ZNF708	2.904496
ZNF709	2.42711
ZNF71	4.343778
ZNF710	6.812682
ZNF711	2.734274
ZNF713	4.301592
ZNF714	4.48295
ZNF716	2.736838
ZNF717	6.304034
ZNF720	6.881742
ZNF721	4.89469
ZNF724P	3.184964
ZNF726	2.545176
ZNF729	2.185954
ZNF730	2.43388
ZNF732	2.279384
ZNF737	2.20223
ZNF738	3.950424
ZNF74	5.676644
ZNF740	7.683854
ZNF746	7.715772
ZNF747	6.941138
ZNF749	5.223772
ZNF750	4.063494
ZNF75A	5.712708
ZNF75A	4.12384
ZNF75D	5.454794
ZNF76	6.501002
ZNF761	4.812456
ZNF763	2.313656
ZNF764	5.833876
ZNF765	6.115172
ZNF765	7.405904
ZNF766	5.483614
ZNF767	6.510836
ZNF768	7.707404
ZNF77	4.878966
ZNF770	2.798616
ZNF770	6.774764
ZNF771	5.047816
ZNF772	4.588794
ZNF773	5.539822

ZNF774	4.927678
ZNF775	5.981202
ZNF776	6.407286
ZNF777	6.102834
ZNF778	5.58156
ZNF778	7.691102
ZNF780A	4.75226
ZNF780B	5.325468
ZNF781	2.43204
ZNF782	4.579362
ZNF783	5.540622
ZNF784	6.342304
ZNF785	6.257264
ZNF786	5.797402
ZNF787	7.665408
ZNF788	2.52117
ZNF789	5.189078
ZNF79	5.840898
ZNF790	3.024246
ZNF791	6.276814
ZNF792	3.460496
ZNF793	3.333398
ZNF799	3.731842
ZNF8	7.190842
ZNF80	2.698578
ZNF800	5.601778
ZNF804A	2.63858
ZNF804B	2.929696
ZNF805	6.167066
ZNF808	6.248344
ZNF81	3.74448
ZNF813	2.64532
ZNF814	6.721058
ZNF816	2.485804
ZNF818P	2.518594
ZNF821	4.208728
ZNF823	4.561578
ZNF826P	2.96852
ZNF827	6.056096
ZNF828	6.384626
ZNF829	2.45311
ZNF83	3.517978
ZNF830	6.902298

ZNF831	3.820534
ZNF833P	3.047554
ZNF835	5.063866
ZNF836	5.013958
ZNF837	4.964332
ZNF839	5.854358
ZNF84	4.16491
ZNF841	5.940854
ZNF843	4.430014
ZNF844	2.646082
ZNF845	4.983124
ZNF846	2.389054
ZNF847P	2.038648
ZNF85	2.875354
ZNF850	3.18111
ZNF852	3.340782
ZNF852	5.438246
ZNF860	2.831272
ZNF861P	3.231784
ZNF862	5.57585
ZNF865	5.247628
ZNF876P	2.51881
ZNF879	3.10064
ZNF880	2.916328
ZNF90	2.832846
ZNF90	8.984722
ZNF91	3.710296
ZNF92	4.254572
ZNF93	2.424106
ZNF98	2.562328
ZNF99	2.11217
ZNFX1	8.712902
ZNHIT1	4.315014
ZNHIT2	5.377352
ZNHIT3	7.746212
ZNHIT6	6.122752
ZNRD1	4.773414
ZNRD1	6.611632
ZNRD1-AS	3.684444
ZNRF1	6.183522
ZNRF2	7.780262
ZNRF3	5.340576
ZNRF4	3.892976

ZP1	3.8027
ZP2	2.844804
ZP3	4.19796
ZP4	2.827224
ZPBP	2.591832
ZPBP2	2.548188
ZPLD1	3.042136
ZRANB1	9.19476
ZRANB2	7.702094
ZRANB3	4.151276
ZRSR2	6.375378
ZSCAN1	5.085804
ZSCAN10	4.830942
ZSCAN12	3.608858
ZSCAN16	5.12082
ZSCAN18	4.296632
ZSCAN2	3.938436
ZSCAN20	5.288328
ZSCAN21	5.853858
ZSCAN22	5.011798
ZSCAN23	2.423844
ZSCAN29	7.155056
ZSCAN30	3.072642
ZSCAN4	2.639564
ZSCAN5A	6.031332
ZSCAN5B	2.935396
ZSCAN5C	5.933734
ZSWIM1	6.7237
ZSWIM2	2.30612
ZSWIM3	4.268506
ZSWIM4	6.020928
ZSWIM5	4.514016
ZSWIM6	6.429616
ZUFSP	4.3043
ZW10	7.171618
ZWILCH	7.41842
ZWINT	7.19912
ZXDA	7.223698
ZXDB	6.080156
ZXDC	7.024302
ZXDC	5.271318
ZYG11A	6.686874
ZYG11B	6.914388

ZYX	8.400086
ZZEF1	3.483942
ZZEF1	6.481394
ZZZ3	6.975542
