# Supplementary figures and images for: Systems Analysis of Insulin and IGF1 Receptors Networks in Breast Cancer Cells Identifies Commonalities and Divergences in Expression Patterns
Source: Front Endocrinol (Lausanne). 2020 Jul 7;11:435. doi: 10.3389/fendo.2020.00435 (PMC7359857; doi:10.3389/fendo.2020.00435)

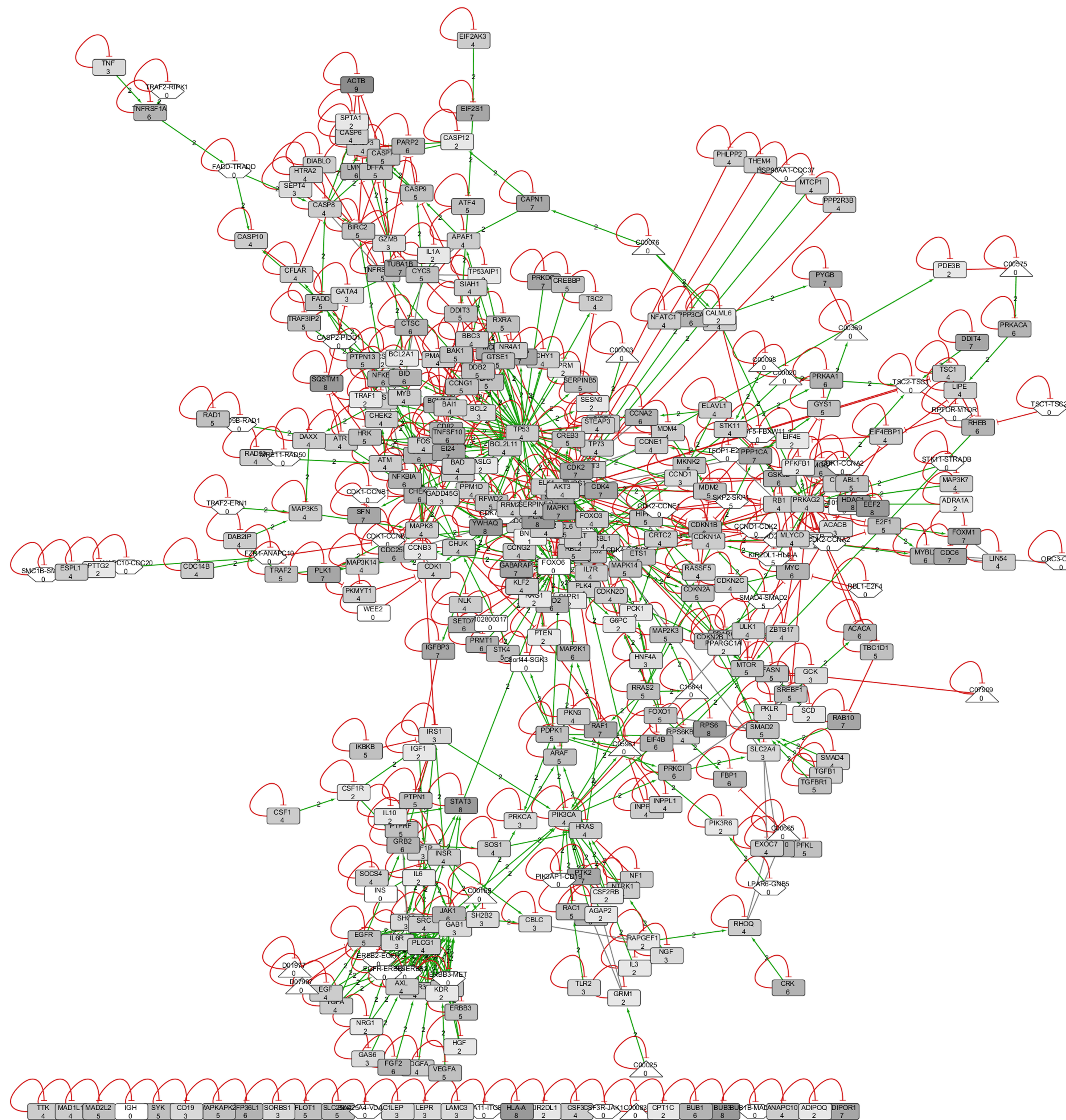

Supplement: Supplementary Data Sheet 2 — Complete network file containing 385 nodes and edges from nine KEGG pathways with normalized expression values from the gene expression file (22,148 genes). The file (CYS) can be opened in cytoscape (https://cytoscape.org/download.html). A pdf file is provided. [file Data_Sheet_2.PDF]
