# Supplementary material for: Systems Analysis of Insulin and IGF1 Receptors Networks in Breast Cancer Cells Identifies Commonalities and Divergences in Expression Patterns
Source: Front Endocrinol (Lausanne). 2020 Jul 7;11:435. doi: 10.3389/fendo.2020.00435 (PMC7359857; doi:10.3389/fendo.2020.00435)
Supplement: Supplementary Data Sheet 3 — Unprocessed original scans for all of the figures containing blots. [file Data_Sheet_3.PDF]

Systems analysis of insulin and IGF1 receptors networks in breast cancer cells identifies commonalities and divergences in expression patterns

Rive Sarfstein, Adva Yeheskel, Tali Sinai-Livne, Metsada Pasmanik-Chor and Haim Werner

SUPPLEMENTARY INFORMATION

The supplementary figures presented here correspond to the unprocessed original scans for all of the figures containing blots. The supplementary figure numbers correspond to the numbers in the manuscript. Squares denote lanes shown in the manuscript figures. Additional information is presented in the legends to figures in the manuscript.

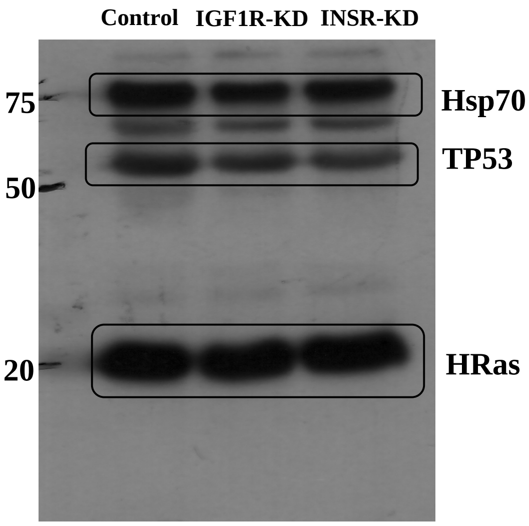

Figures 3A and 3C

Control

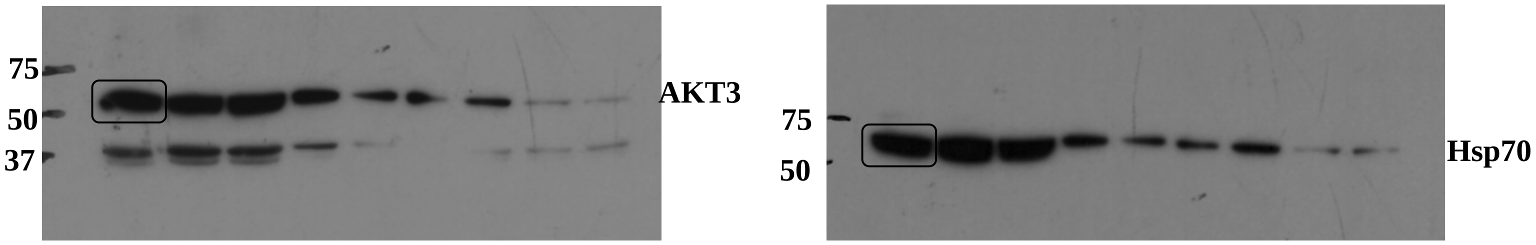

IGF1R-KD

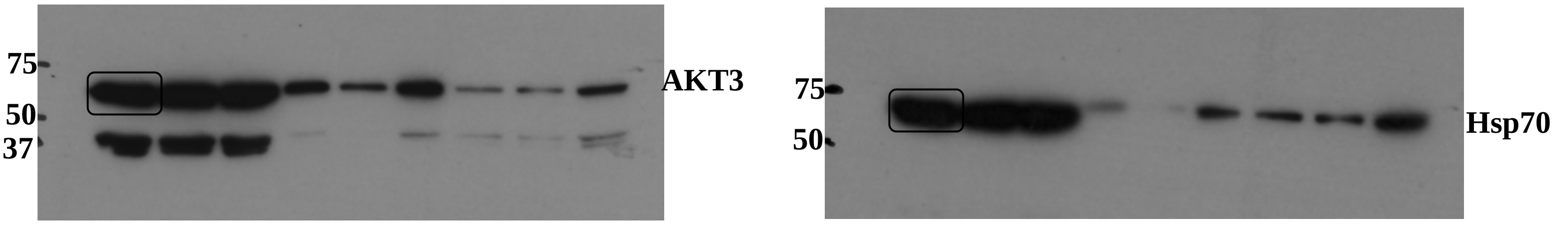

INSR-KD

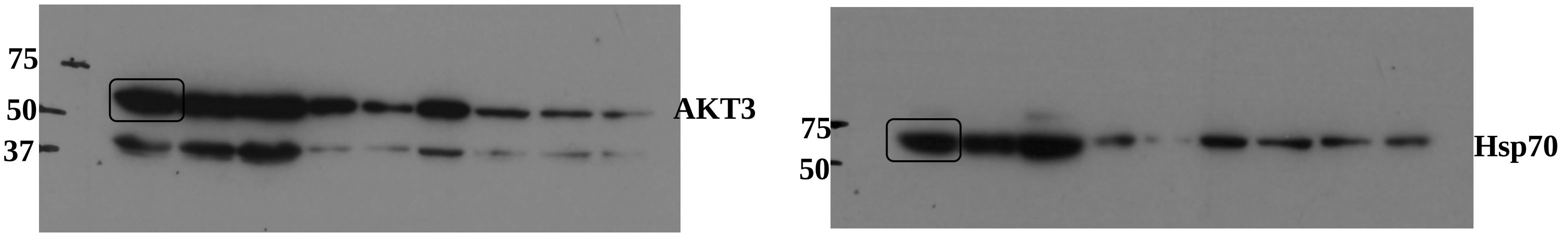

Figure 3E

Control

50
37
25
20
15

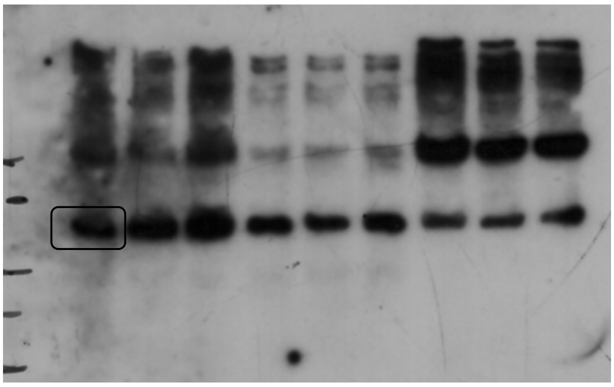

Casp3

250
150
100
75
50

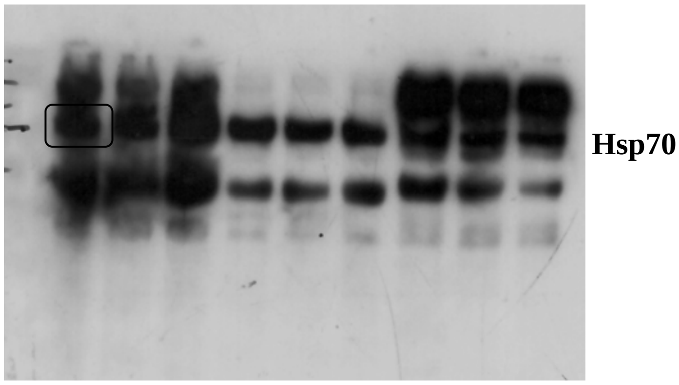

Hsp70

IGF1R-KD

50
37
25
20
15

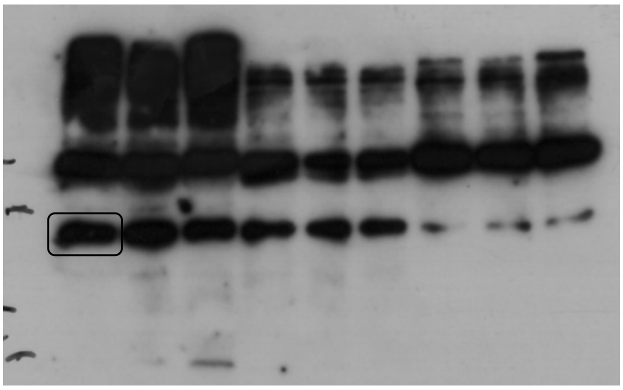

Casp3

75
50

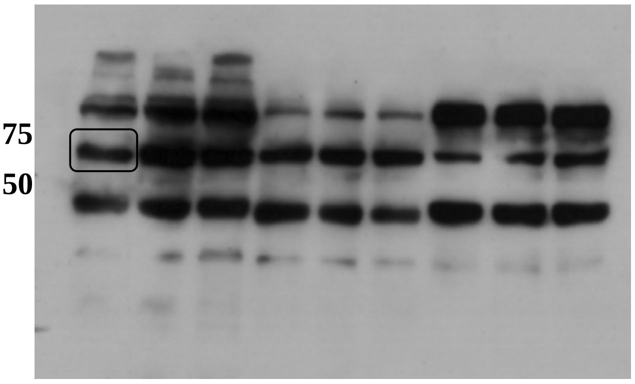

Hsp70

INSR-KD

50
37
25
20
15

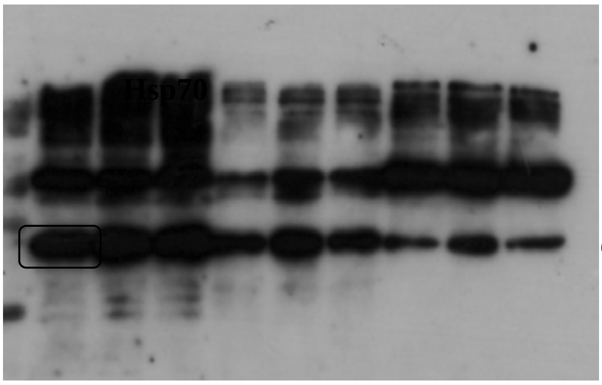

Casp3

75
50

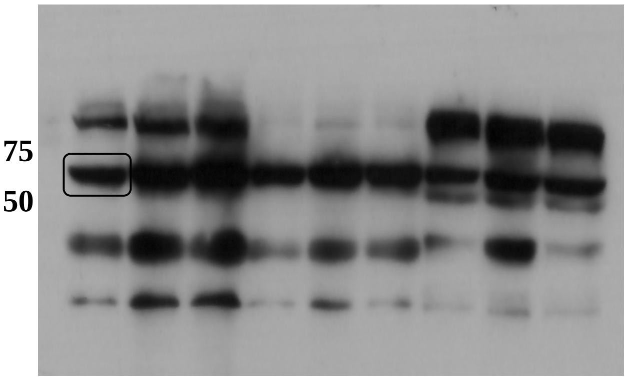

Hsp70

Figures 3G

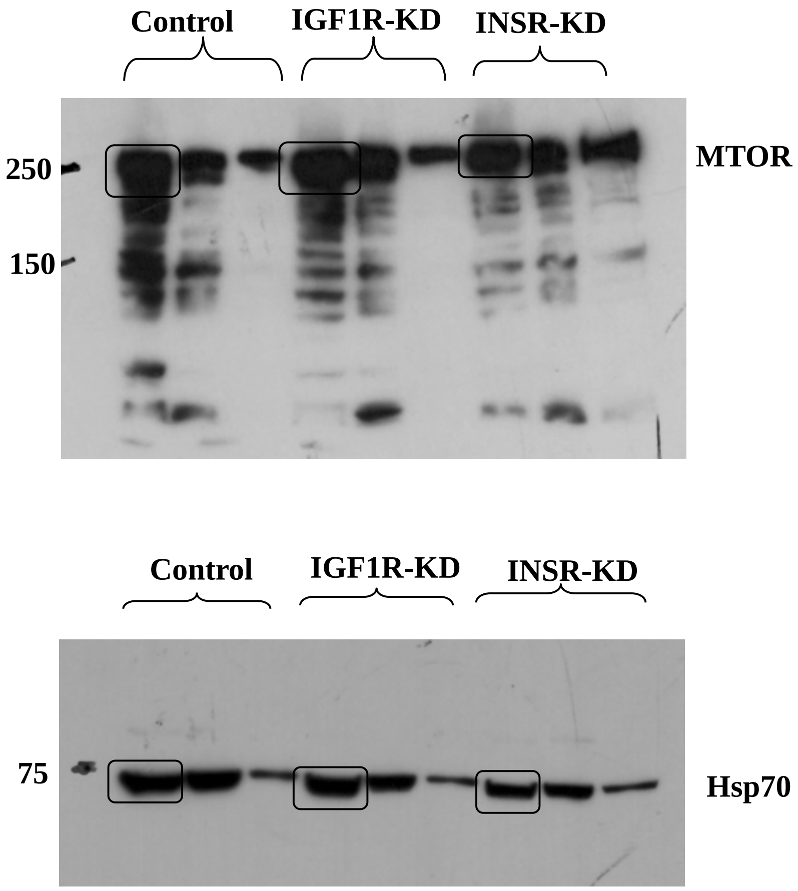
Control
IGF1R-KD
INSR-KD
250
150
MTOR
Control
IGF1R-KD
INSR-KD
75
Hsp70

Figure 3I

Control

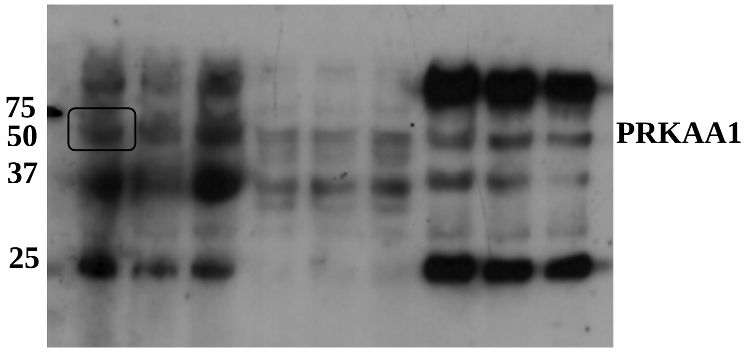

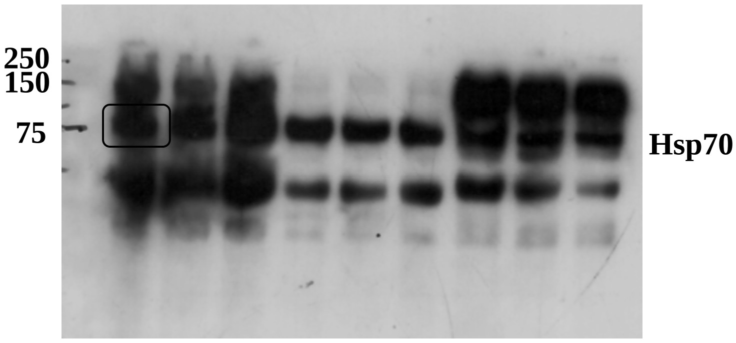

IGF1R-KD

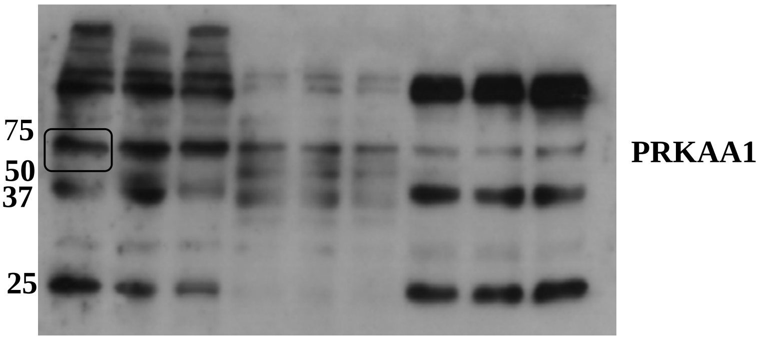

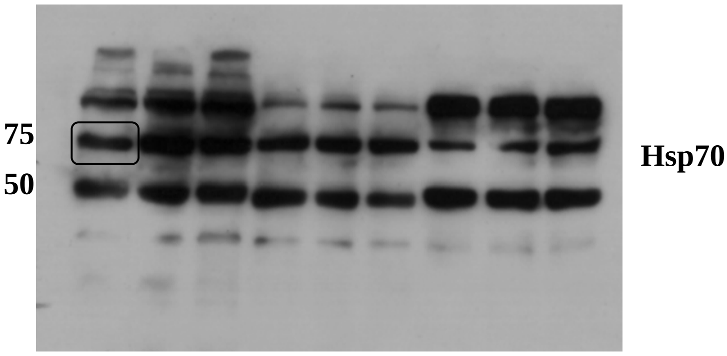

INSR-KD

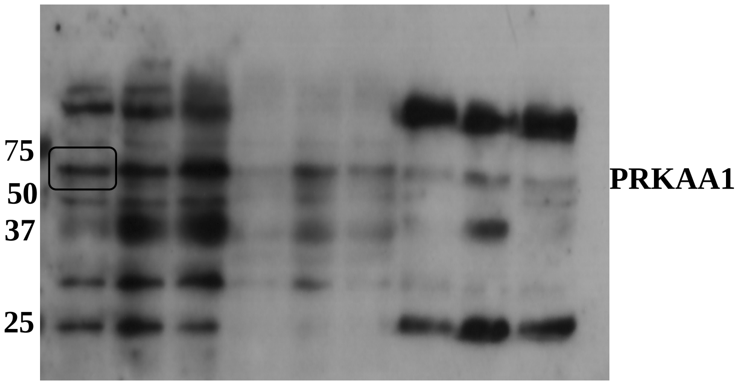

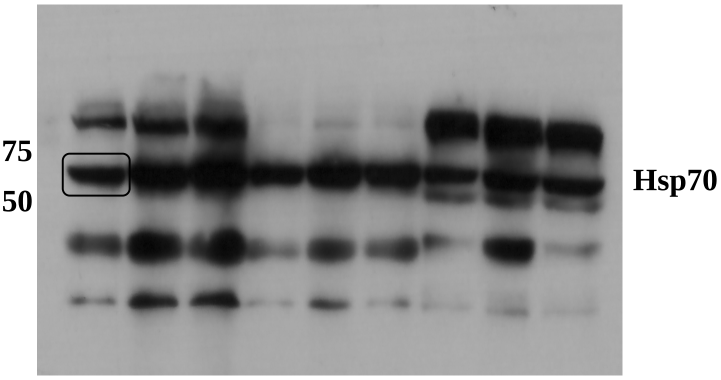

Figure 3K

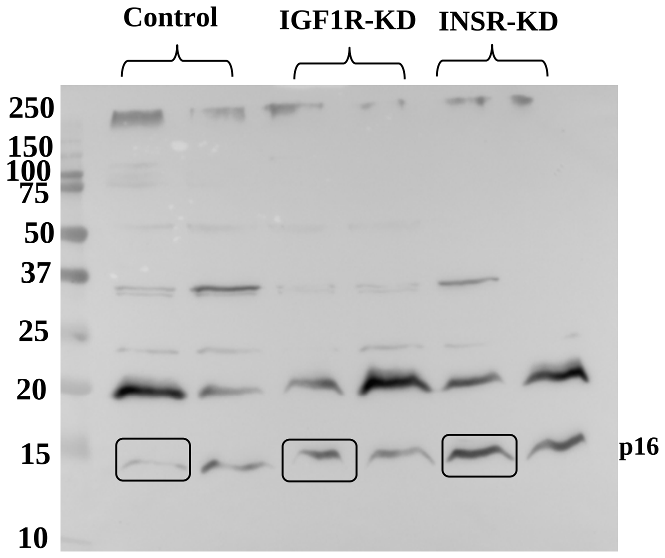
Control
IGF1R-KD
INSR-KD
250
150
100
75
50
37
25
20
15
10
p16

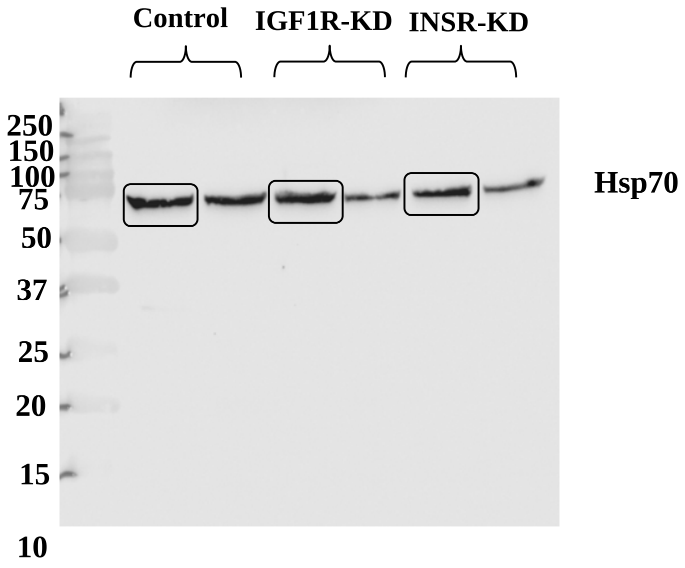
Control
IGF1R-KD
INSR-KD
250
150
100
75
50
37
25
20
15
10
Hsp70

Figure 3M

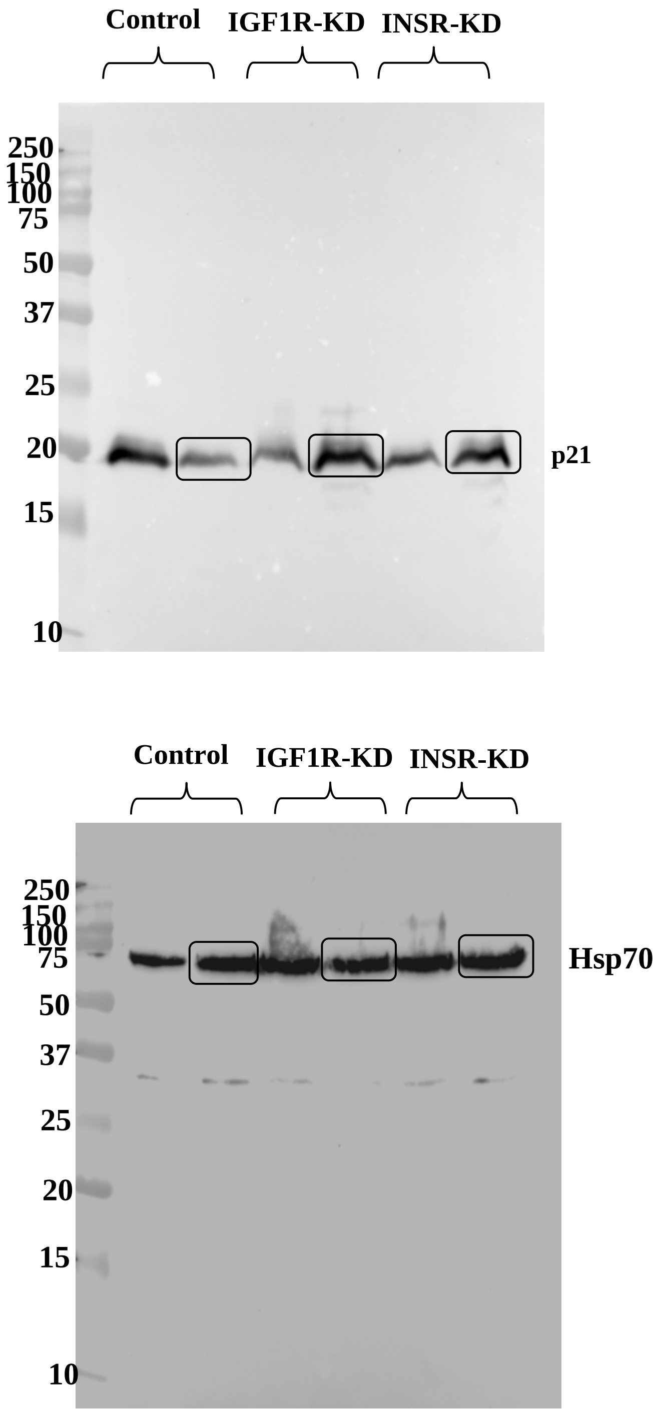
Control
IGF1R-KD
INSR-KD
250
150
100
75
50
37
25
20
15
10
p21
Control
IGF1R-KD
INSR-KD
250
150
100
75
50
37
25
20
15
10
Hsp70

Figure 3O

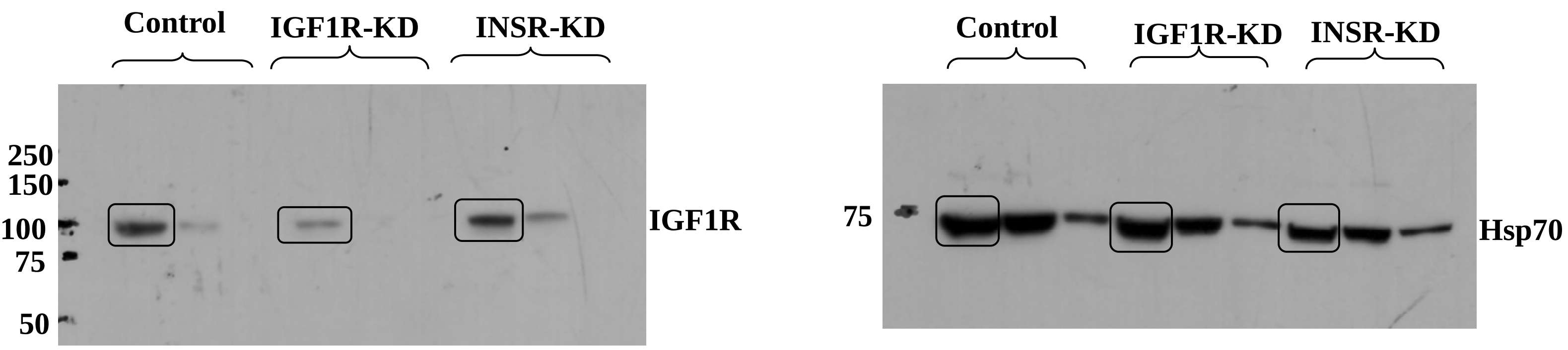

Figure 4A

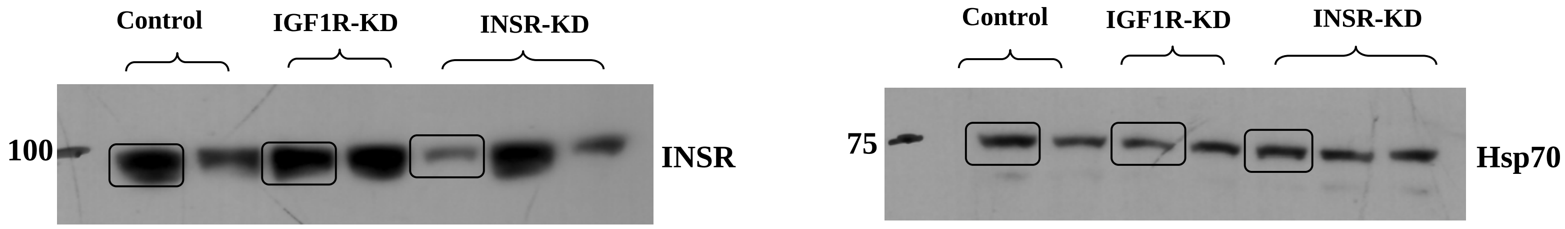

Figure 4B

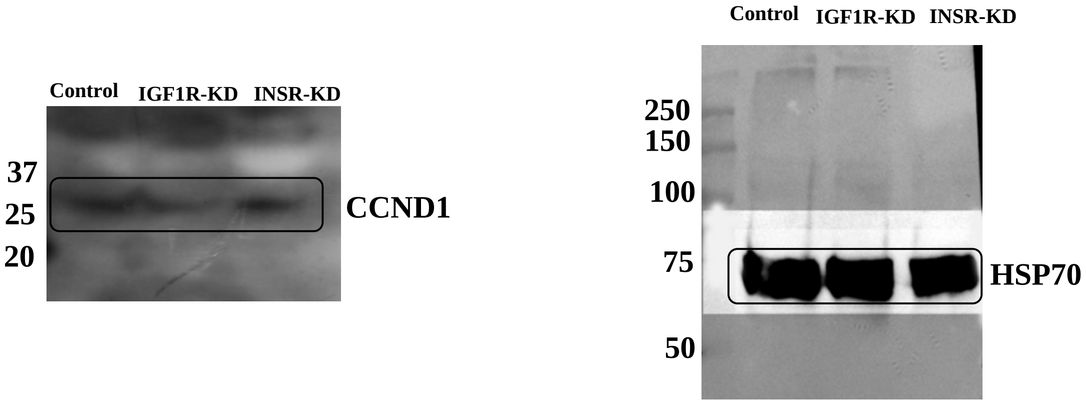
Control
IGF1R-KD
INSR-KD
37
25
20
CCND1
Control
IGF1R-KD
INSR-KD
250
150
100
75
50
HSP70

Figure 5A

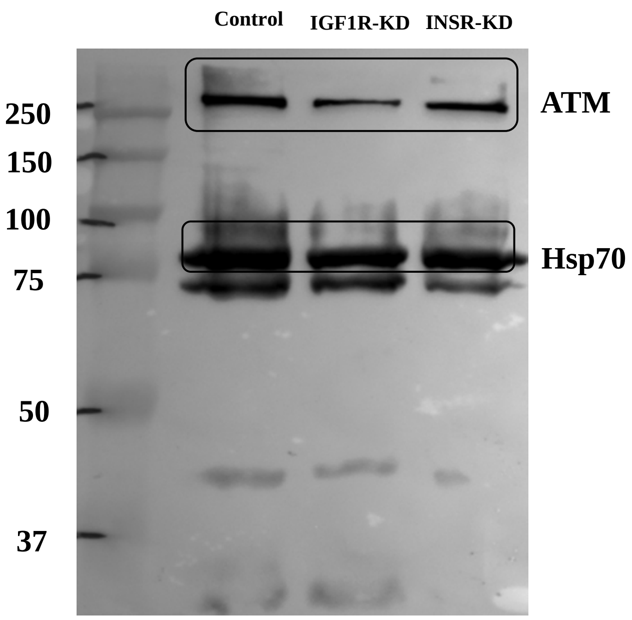

Figure 5D

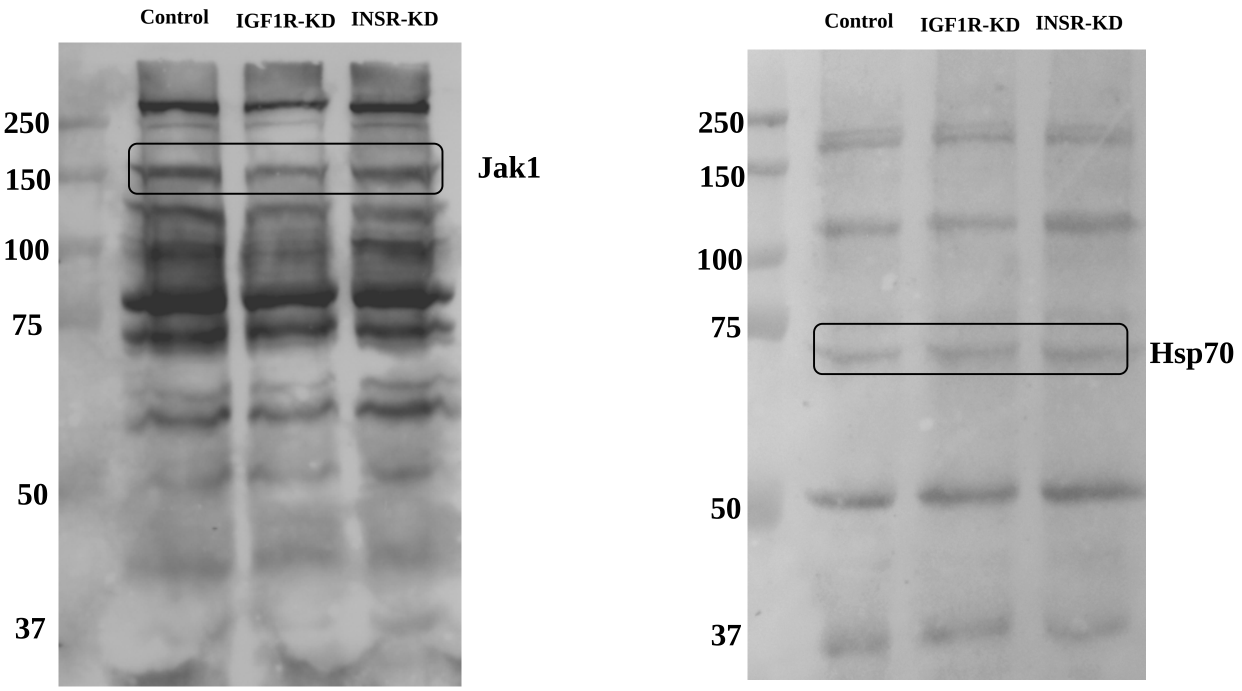
Control
IGF1R-KD
INSR-KD
250
150
100
75
50
37
Jak1
Control
IGF1R-KD
INSR-KD
250
150
100
75
50
37
Hsp70

Figure 5G

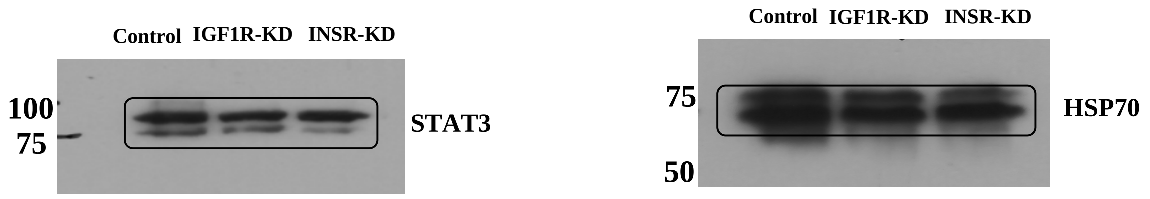
Control
IGF1R-KD
INSR-KD
100
75
STAT3
Control
IGF1R-KD
INSR-KD
75
50
HSP70

Figure 5J

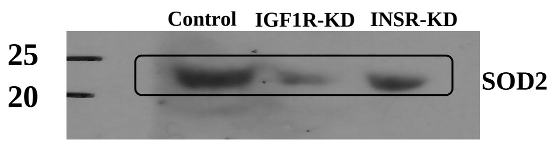
Control IGF1R-KD INSR-KD
25
20
SOD2

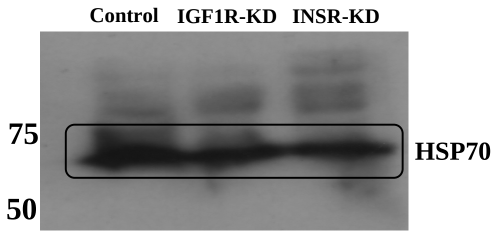
Control IGF1R-KD INSR-KD
75
50
HSP70

Figure 5N

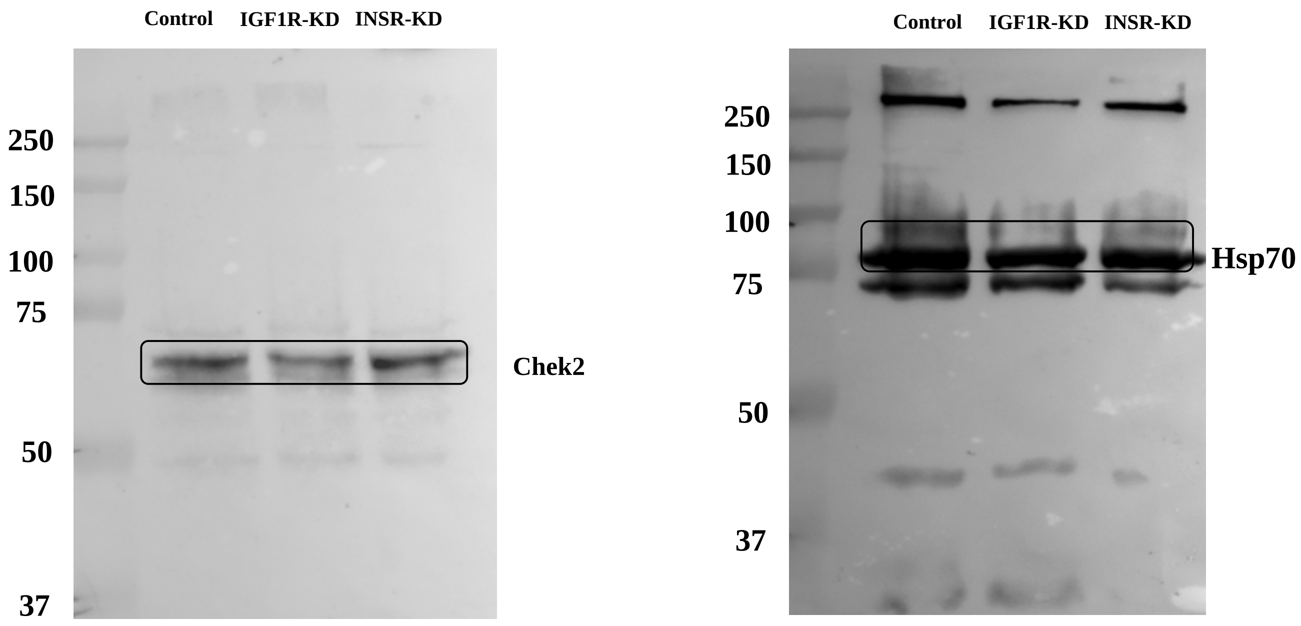
Control
IGF1R-KD
INSR-KD
250
150
100
75
50
37
Chek2
Control
IGF1R-KD
INSR-KD
250
150
100
75
50
37
Hsp70

Figure 5Q

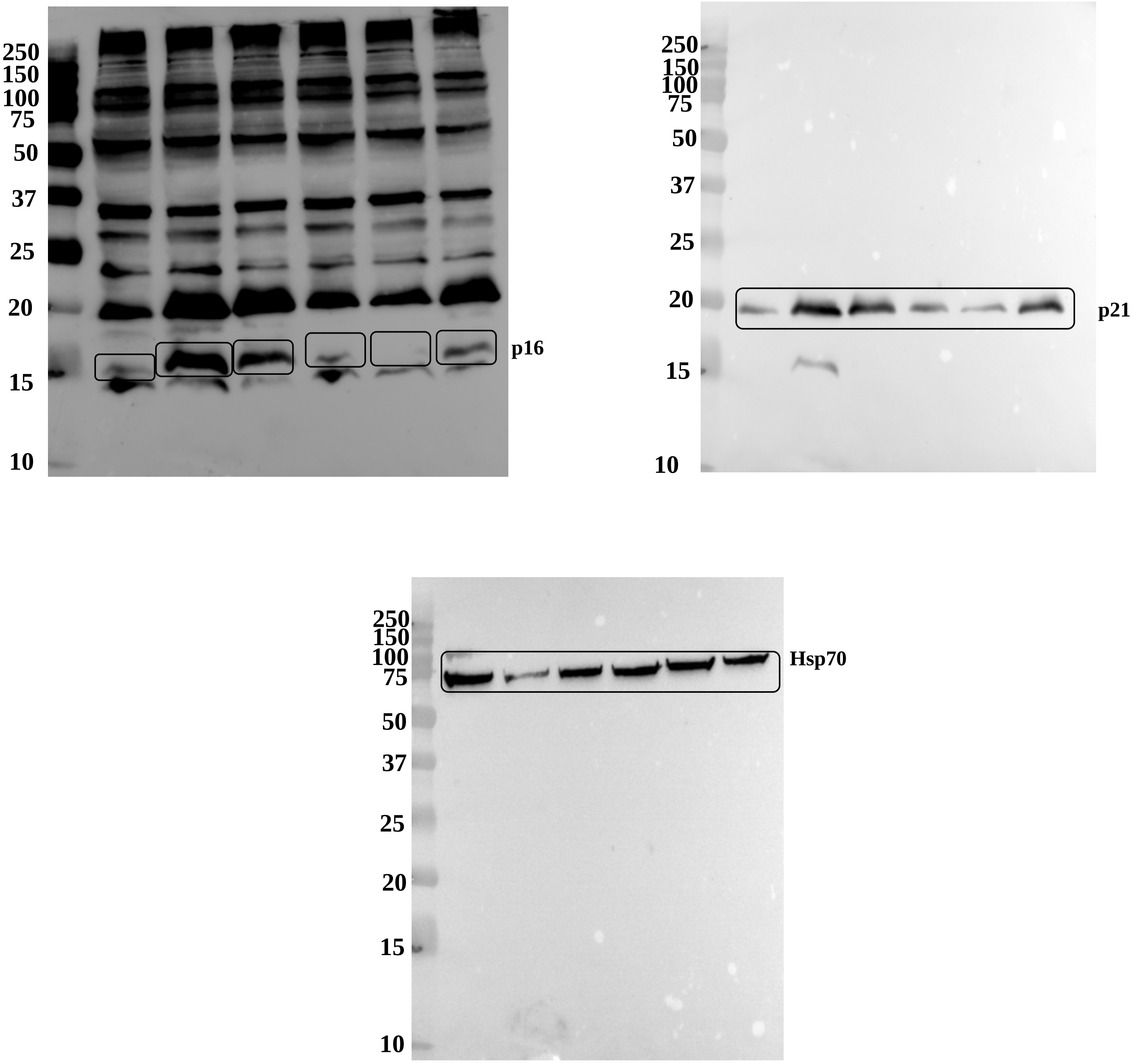

Figure 7C
